# Levofloxacin reposition-based design: synthesis, biological evaluation of new levofloxacin derivatives targeting topoisomerase II beta polymerase as promising anticancer agents, molecular docking, and physicochemical characterization†

**DOI:** 10.1039/d4ra03975k

**Published:** 2024-09-03

**Authors:** Zeinab Mahmoud, Mohamed M. Ismail, Mona Kamel, Amira Youssef

**Affiliations:** a Pharmaceutical Organic Chemistry Department, Faculty of Pharmacy, Cairo University 11561 Cairo Egypt; b Pharmaceutical Organic Chemistry Department, College of Pharmaceutical Sciences and Drug Manufacturing, Misr University for Science and Technology P. O. Box 77 Giza Egypt amira.youssef@must.edu.eg +201285266644

## Abstract

Repositioning of already approved medications through repurposing or re-profiling for new medical uses after certain structural modifications is a novel approach in drug discovery. Fluoroquinolone antibiotics are one of the cardinal agents investigated for potential anticancer activities. In this work, levofloxacin was repositioned for anticancer activities. A series of levofloxacin-based compounds were designed and synthesized through the derivatization of levofloxacin's carboxylic acid functionality. The newly synthesized compounds were screened for cytotoxic activities against breast, liver, and leukemia cancer cell lines. Their effect on normal cells was also investigated. The target compounds were evaluated for their proliferative inhibitory activity toward topoisomerase II beta polymerization. Compound 5 showed higher inhibitory activity against a breast cancer cell line (MCF-7) with IC_50_ = 1.4 μM and lesser side effects on a normal breast cell line (MCF-10a) with IC_50_ = 30.40 μM than reference drugs. The best activity against a liver cancer cell line (Hep3B) was exhibited by compounds 3c, 4b, 5, 7, 8, 13a and 13c with IC_50_ values ranging from 0.43 to 8.79 μM. Regarding the effect of compounds 5 and 13a on a leukemia cancer cell line (L-SR), their IC_50_ values were 0.96 and 3.12 μM, respectively. Compounds 3c and 5 showed Topo2β inhibitory effects on Hep3B cells (81.33% and 83.73%, respectively), which was better than levofloxacin and etoposide as reference drugs. Cytometry cell cycle analysis revealed that compounds 3c and 5 arrested the cell cycle at the S phase (37.56% and 39.09%, respectively). Compounds 3c and 5 exhibited an elevation in active caspase-3 levels by 4.9 and 4.5 folds, respectively. Molecular modeling simulation of compounds 3c and 5 demonstrated energy scores (−29.77 and −20.46 kcal mol^−1^, respectively) more than those of the reference drugs as they interact with the most essential amino acids required for good affinity towards human topoisomerase II beta enzyme (PDB ID: 3QX3). Physicochemical characteristics of the most promising cytotoxic compounds 3c and 5 were investigated and compared to etoposide and levofloxacin as reference drugs. However, they showed high gastrointestinal absorption and could not penetrate the blood–brain barrier.

## Abstract 2

Repositioning of already approved medications through repurposing or re-profiling for new medical uses after certain structural modifications is a novel approach in drug discovery.

## Graphical abstract

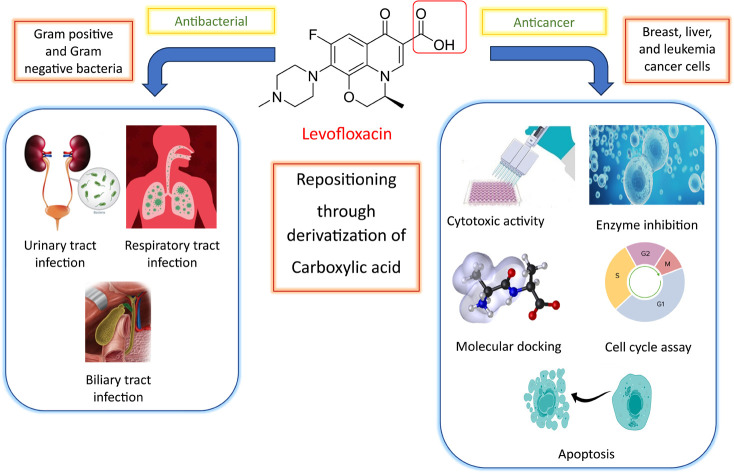

## 1. Introduction

**Fig. 1:**
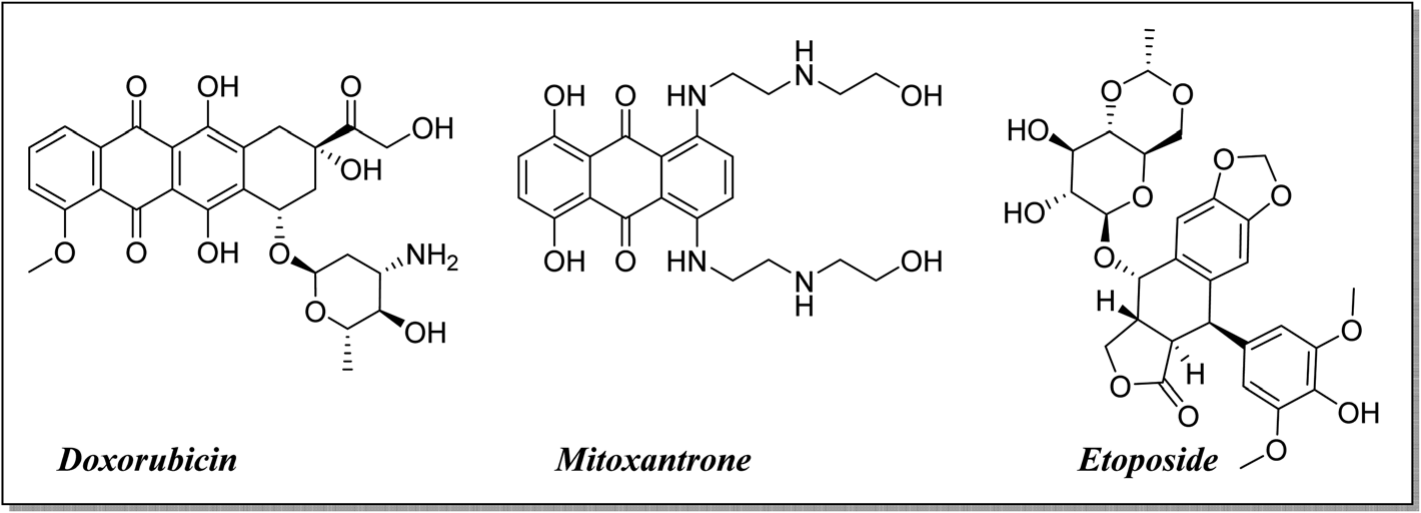
Significant topoisomerase II inhibitors.

The high costs coupled with the increased attrition rate in developing new and efficacious anti-cancer agents for clinical use necessitate alternative thinking.^1–4^ There is increasing interest towards the repositioning of existing drugs and reevaluating them for new biological activities.^5–8^ It makes the repositioning of fluoroquinolones as antitumor agents justifiable by their apoptotic potential, anti-proliferative activity and induced cell cycle arrest in addition to their use for decades as antimicrobial agents with different derivatives.^9–14^ Fluoroquinolones exhibit the required features in cancer treatment, such as lower incidence of resistance development, reduced toxicity, diminished tendency for the development of drug-induced secondary tumors, and higher potency than other topoisomerase II inhibitors^15–18^ (Fig. 1).

Moreover, fluoroquinolone derivatives are characterized by their ease and flexibility of synthesis using different procedures and building blocks to afford several useful chemical structures.^15^ It was reported that replacement of the C-3 carboxylic group of fluoroquinolone antibacterial analogs with a heterocyclic ring can yield novel antitumor analogs. More specifically, the replacement of the carboxylic group of fluoroquinolones with a heterocyclic ring, hydrazones, or Mannich bases furnished a promising approach for developing novel anticancer agents.^10,17,19,20^ The aim of this study was to design and synthesize new levofloxacin derivatives with improved biological activities.

**Fig. 2:**
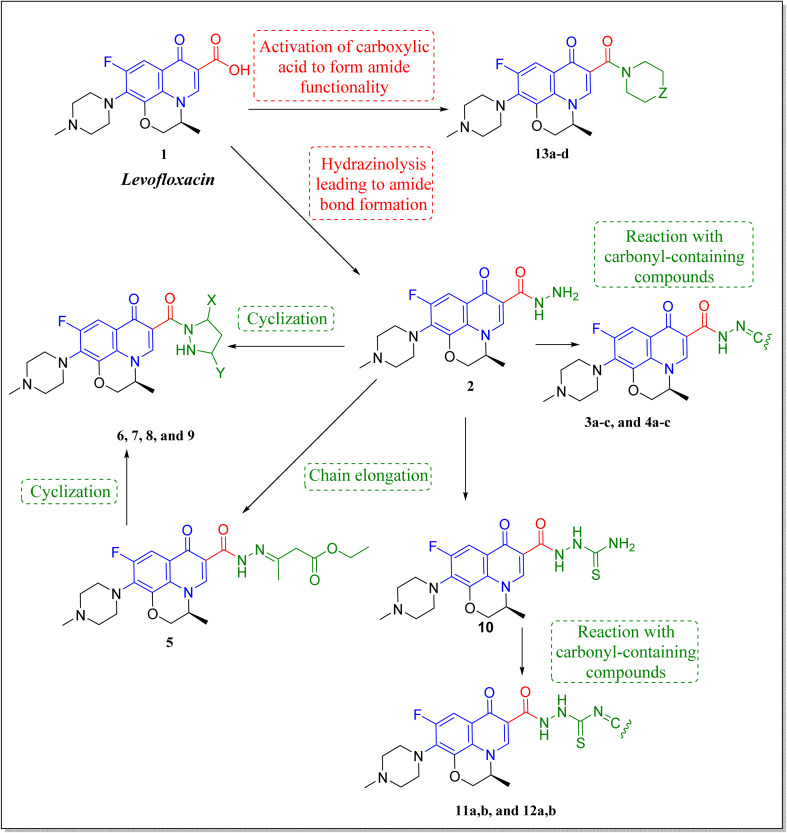
Rational strategy for repositioning levofloxacin towards anticancer activity.

Levofloxacin is a well-known, widely used synthetic fluoroquinolone antibacterial agent. Moreover, many research studies highlighted its biological effect as an anticancer agent,^21–24^ through the inhibition of human topoisomerase II,^25–28^ involving topoisomerase II beta.^28^ It was concluded that the activity of levofloxacin on Topo2β is more potent than that of Topo2α, as it interacted with the TRP931 amino acid residue with a binding energy of −8.06 on Topo2α, while the binding with Topo2β showed interaction with 2 amino acid residues (SER480 and ASP479) with a better binding energy of −11.78. Moreover, levofloxacin was investigated for cell cycle analysis showing cell cycle arrest primarily in the S and G2/M phases of progression with a significant increase in caspase-3 levels.^29–32^ Structural features required for the anticancer activity of levofloxacin have been well defined.^33^ Most of the required chemical modifications to reposition levofloxacin from its antibacterial activity into its anticancer one were at its carboxylic group functionality.^15,25,33–36^ Based on the illustrated findings, this study aimed to design levofloxacin-based selective mammalian topoisomerase II beta (Topo2β) enzyme inhibitors as potent anticancer agents according to the denoted tactic in Fig. 2.

## 2. Results and discussion

### 2.1. Chemistry

**Scheme 1:**
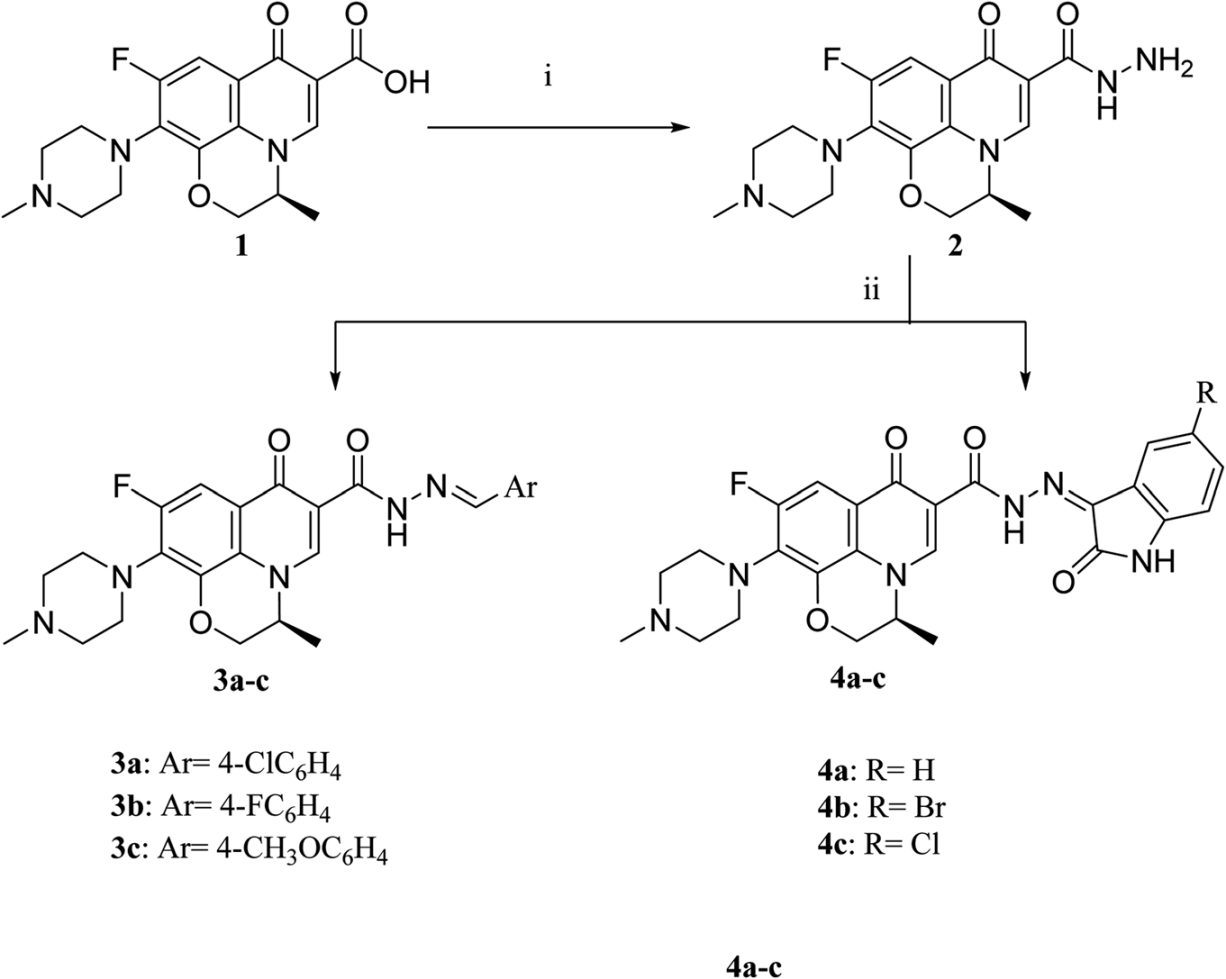
Preparation of levofloxacin derivatives 3a–c and 4a–c. Reagents and conditions: (i) 80% NH_2_NH_2_, reflux 24 h; (ii) appropriate carbonyl-containing compounds, absolute ethanol, glacial acetic acid, reflux 6 h.

**Scheme 2:**
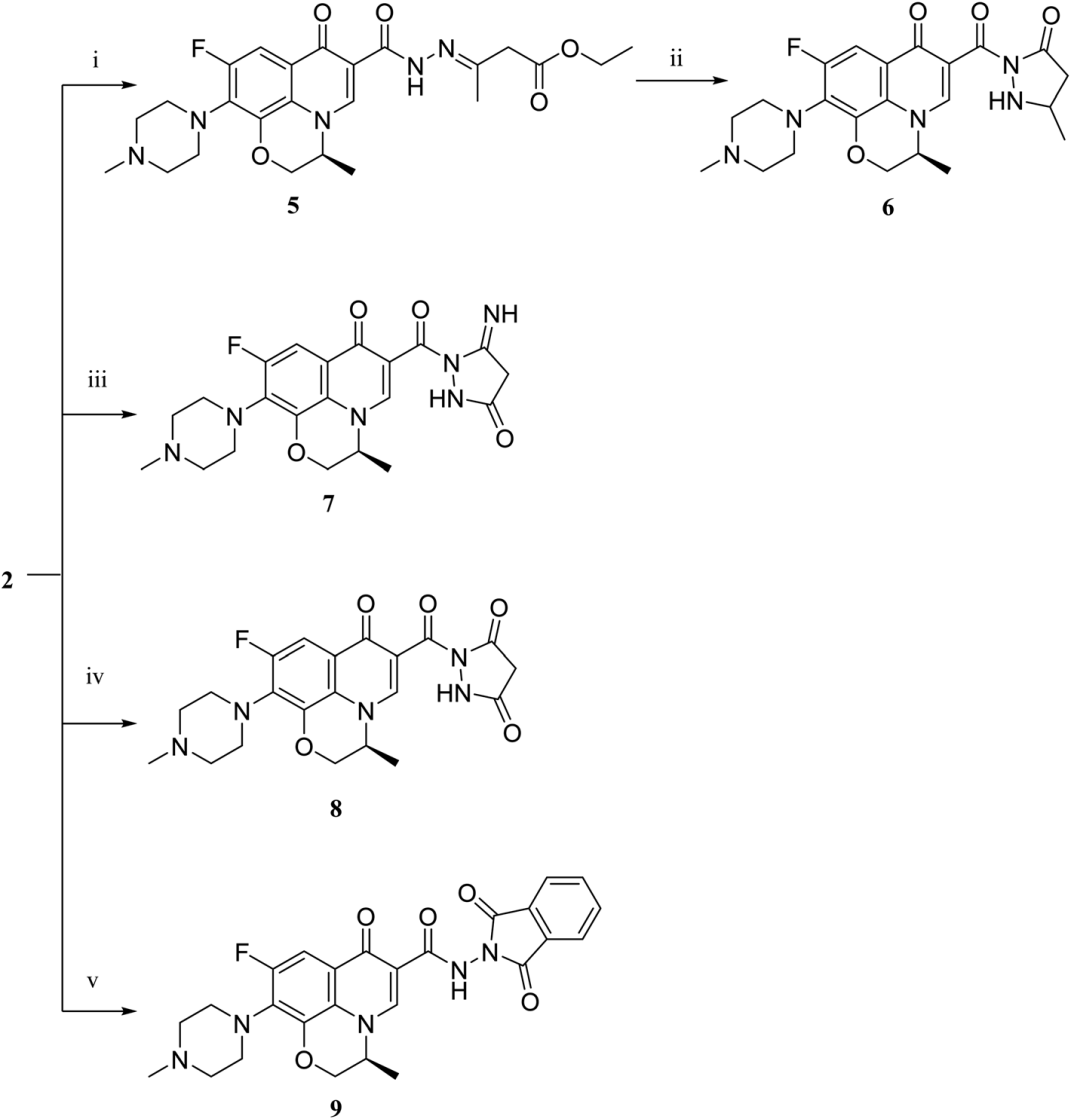
Preparation of levofloxacin derivatives 5, 6, 7, 8 and 9. Reagents and conditions: (i) ethyl acetoacetate, absolute ethanol, reflux 12 h; (ii) reflux 90 h; (iii) ethyl cyanoacetate, absolute ethanol, piperidine, reflux 90 h; (iv) diethyl malonate, absolute ethanol, sodium ethoxide, reflux 7 h; (v) phthalic anhydride, absolute ethanol, glacial acetic acid, reflux 4 h.

**Scheme 3:**
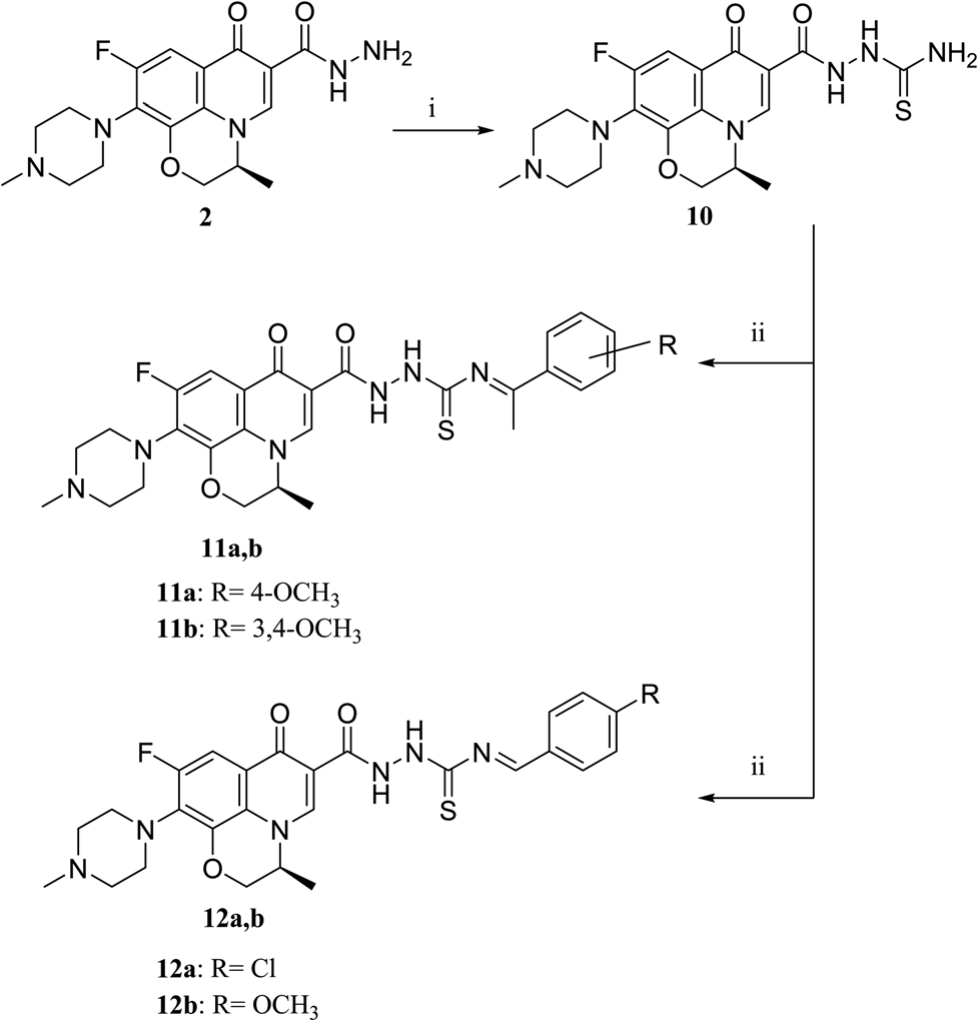
Preparation of levofloxacin derivatives 10, 11a,b and 12a,b. Reagents and conditions: (i) KSCN, absolute ethanol, conc. HCl, reflux 8 h; (ii) appropriate carbonyl-containing compounds, absolute ethanol, sodium acetate, reflux 12 h.

The deigned levofloxacin-based compounds were synthesized according to four schemes. In Scheme 1, levofloxacin 1 undergoes two main reactions. The hydrazinolysis of levofloxacin with hydrazine hydrate (80%) afforded the levofloxacin hydrazide 2 according to the reported procedure.^35^ Compound 2 is considered as the cornerstone for synthesizing all new members in Schemes 1–3. All the newly synthesized compounds were elucidated by ^1^H NMR, ^13^C NMR and IR spectroscopic techniques, and are in agreement with the rational correspondence of the proposed structures. Accordingly, refluxing compound 2 with different carbonyl-containing compounds in absolute ethanol in the presence of glacial acetic acid afforded the *N*′-benzylidinequinoline-6-carbohydrazide series 3 and 4. It was reported that fluoroquinolone Schiff's bases could be obtained by simple condensation of fluoroquinolone hydrazides with different carbonyl compounds in absolute ethanol in the presence of glacial acetic acid under reflux.^37,38^ Compounds 3a–c and 4a–c were prepared *via* the conventional pathway by reacting levofloxacin hydrazide 2 with the appropriate carbonyl-containing compounds namely, 4-chlorobenzaldehyde, 4-fluorobenzaldehyde and anisaldehyde to give 3a–c, and isatin, 5-bromoisatin and 5-chloroisatin to afford 4a–c, respectively. Interestingly, tracking the appearance and disappearance with the accurate integration of amino groups (NH) and (NH_2_) was so helpful in identifying the newly synthesized compounds. The D_2_O-influenced disappearance of the (NH) singlet peak in the range of *δ* 13.37–10.64 ppm corresponding to only one proton integration in addition to the drastic increase in the aromatic protons' integration confirmed the success of the reactions of this pathway. Compounds 4a–c showed two D_2_O exchangeable singlet signals around *δ* 10.77–13.85 ppm corresponding to two NH protons and exhibited aromatic protons in the range of *δ* 6.86–7.60 ppm in ^1^H NMR spectroscopy. The ^13^C NMR spectrum displayed the appearance of three peaks in the range of *δ* 174.6–159.1 ppm.

Literature review showed that the related cyclization reactions of hydrazides with ethyl acetoacetate have been performed by heating under reflux in absolute ethanol,^39–41^ or in glacial acetic acid.^42,43^ In contrast, in Scheme 2, the reaction of compound 2 with ethyl acetoacetate ester afforded time-dependent. More clearly, levofloxacin hydrazide 2 reacted with ethyl acetoacetate in absolute ethanol under reflux for 12 hours and the reaction was monitored by TLC. The spectral data of the obtained compound 5 revealed that it is the open uncyclized product. However, upon extending the reaction time, a change in color was observed. This continuous change in color encouraged us to continue the reaction up to 90 hours under reflux to afford the cyclized pyrazolidone ring compound 6. The spectral data of both compounds 5 and 6 were in accordance with the predicted assumption. The ^1^H NMR spectrum of compound 5 revealed the presence of the triplet signal at *δ* 1.22 ppm corresponding to the methyl group of OCH_2_C*H*_3_ and the quartet signal of the two protons at *δ* 4.12 ppm related to the presence of OC*H*_2_CH_3_. Meanwhile, the signal of the two protons at *δ* 3.26 ppm corresponding to N=C(CH_3_)C*H*_2_ and a singlet signal corresponding to three protons at *δ* 2.02 confirm the presence of N=C(C*H*_3_)CH_2_. On the contrary, the ^1^H NMR spectrum of compound 6 showed the expected signals for the two CH_2_ groups of the pyrazolidone ring protons at *δ* 1.84 and 1.89 ppm, and the multiplet signal of three protons in the range of *δ* 2.02–2.09 ppm corresponding to the presence of CH_3_ of pyrazolidone ring. The ^1^H NMR spectra showed two D_2_O exchangeable singlet signals at *δ* 10.62 and 10.65 corresponding to the tautomeric OH, while the NH proton of pyrazolidone ring appeared at *δ* 3.26 ppm. Furthermore, refluxing the hydrazide derivative 2 with either ethyl cyanoacetate or diethyl malonate afforded the cyclic amides 7 and 8, sequentially. Compound 7 was prepared through the reaction of levofloxacin hydrazide 2 with ethyl cyanoacetate and a few drops of piperidine in absolute ethanol, following the reported methods.^42^ The ^1^H NMR spectrum of compound 7 revealed the presence of a multiplet signal of two protons in the range of *δ* 4.13–4.18 ppm corresponding to the CH_2_ protons of pyrazolidone ring, and two D_2_O exchangeable singlet signals in the range of *δ* 10.60–11.86 ppm due to two NH protons. The respective levofloxacin pyrazolidine-3,5-dione 8 was prepared *via* refluxing the hydrazide derivative 2 with diethyl malonate in the presence of sodium ethoxide, following the reported steps.^42^ Compound 8^1^H NMR spectrum exhibited a multiplet signal corresponding to two protons of CH_2_ of pyrazolidone ring in the range of *δ* 4.54–4.57 ppm and the D_2_O exchangeable proton corresponding to the signal of NH proton at *δ* 10.62 ppm. The ^13^C NMR spectrum revealed the presence of the 4 carbonyl groups in the range of *δ* 154.4–174.2 ppm. Reviewing the literature revealed that the reaction of hydrazides with phthalic anhydride is achieved under different conditions, namely, fusion,^44^ or in the presence of anhydrous sodium acetate to give the cyclized ring structure.^42^ Adopting these reported procedures failed to give the target compound 9. Refluxing phthalic anhydride with compound 2 for 5 hours in the presence of glacial acetic acid yielded compound 9 as a straightforward product, according to the illustrated procedures.^38,45^^1^H NMR spectrum revealed the presence of a D_2_O exchangeable singlet signal at *δ* 11.67 ppm due to two NH protons, and multiplet signals corresponding to the aromatic protons in the range of *δ* 7.48–7.60 ppm and *δ* 7.93–7.96 ppm. The appearance of four carbonyl carbons in the range of *δ* 165.6–174.2 ppm in the ^13^C NMR spectrum provided evidence. The ^1^H NMR spectrum revealed the presence of a D_2_O exchangeable singlet signal at *δ* 11.67 ppm due to two NH protons, and multiplet signals corresponding to the aromatic protons in the range of *δ* 7.48–7.60 ppm and *δ* 7.93–7.96 ppm, respectively. The appearance of four carbonyl carbons in the range of *δ* 165.6–174.2 ppm in the ^13^C NMR spectrum confirmed the formation of compound 9. The aim of the reactions of Scheme 3 was to bring about the chain elongation strategy for the design. In accordance, refluxing compound 2 with potassium thiocyanate afforded the hydrazinecarbothioamide derivative 10, according to the following procedure.^42^ The ^1^H NMR spectrum of compound 10 revealed the presence of a singlet signal equivalent to four D_2_O exchangeable protons at *δ* 7.89, 9.40, 11.20 and 11.39 ppm corresponding to two NH and NH_2_ protons. In the ^13^C NMR spectrum, the thiocarbonyl carbon appeared at *δ* 174.2 ppm. The condensation reaction of compound 10 with different carbonyl-containing compounds pulled off the production of the thioamide derivatives 11a,b and 12a,b according to the reported procedure.^46^ Series 11 and 12 IR spectrum showed the two NH absorption bands in the range of *ν* 3523–3419 cm^−1^. Additionally, the ^1^H NMR spectrum of compounds 11a and 11b revealed the presence of singlet signals of the two D_2_O exchangeable protons in the range of *δ* 9.38–13.20 ppm, and the presence of a singlet signal of the N=CH proton at *δ* 8.42 and 8.35 ppm, respectively. Moreover, the ^1^H NMR spectrum of compounds 12a and 12b revealed the presence of a singlet signal of the N=C–CH_3_ proton at *δ* 2.35 and 1.85 ppm, sequentially, and the doublet signals corresponding to the aromatic protons in the range of *δ* 7.00–7.81 ppm. However, the ^13^C NMR spectrum of series 11 and only compound 12b showed the presence of a carbon monosulphide atom in the range of *δ* 174.4–188.5 ppm. Unfortunately, the poor solubility of compound 12a hindered running any ^13^C NMR experiment.

**Scheme 4:**
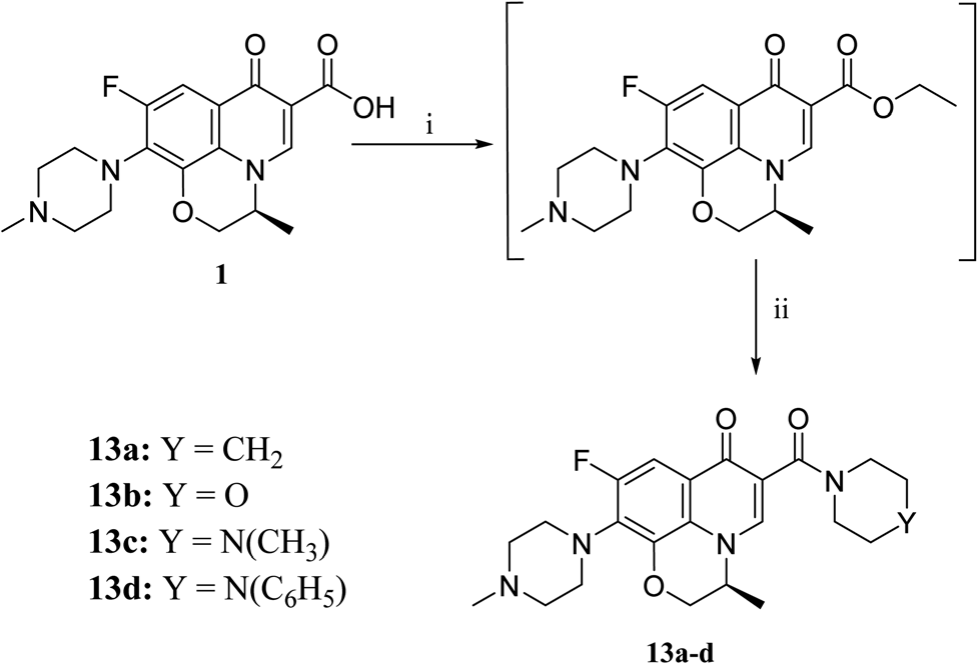
Preparation of levofloxacin derivatives 13a–d. Reagents and conditions: (i) ClCOOEt, triethylamine (TEA), methanol, 0–5 °C, stirring 30 min; (ii) the appropriate secondary amine, 0 °C, srirring 12 h.

The activation of levofloxacin carboxylic acid functionality by esterification was another tactic for amide bond synthesis. The synthesis of methyl levofloxacin ester was reported^47^*via* heating under reflux levofloxacin in methanol in the presence of few drops of concentrated sulphuric acid for about 7–8 hours. To obtain the corresponding amide, aromatic amines were then added in the same round flask with continuous stirring and the reaction was continued to be refluxed for about 2–3 hours. Adopting this procedure in this study failed to afford the target compounds. In Scheme 4, levofloxacin 1 was reacted with ethyl chloroformate in the presence of triethylamine in methanol at 0 °C to give the unseparated intermediate ester, which was directly reacted with the appropriate secondary amines to afford series 13a–d, following the reported method.^48^^1^H NMR spectra showed the aliphatic hydrogen signals of piperazine and azinanylcarbonyl protons in the aliphatic region in the range of *δ* 2.31–3.86 ppm. The ^1^H NMR spectra of compound 13c revealed the appearance of the specific signal corresponding to the methyl group at *δ* 2.31 ppm, while the spectrum of compound 13d showed the appearance of 5 aromatic protons of the *N*-phenyl group in the range of *δ* 6.92–6.99 ppm.

### 2.2. Cytotoxic activity discussion

**Fig. 3:**
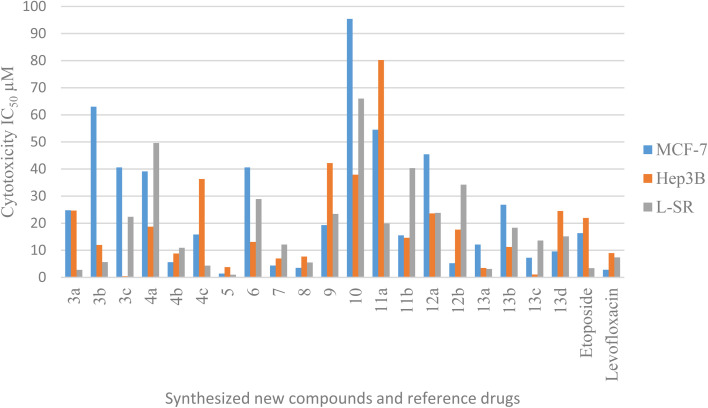
Graphical bar presentation of IC_50_ for the target compounds against MCF-7, Hep3B and L-SR cell lines using MTT assay.

All the test compounds were evaluated for their cytotoxic activity against human breast cancer (MCF-7), human liver cancer (Hep3B) and leukemia (L-SR) cell lines. The new compounds showed promising cytotoxic activity compared with etoposide and levofloxacin as reference drugs. The *in vitro* antitumor activities of compounds 3a–c, 4a–c, 5, 6, 7, 8, 9, 10, 11a,b, 12a,b and 13a–d against the three cell lines were evaluated by an MTT assay. The drug concentration required to inhibit the cell growth by 50% (IC_50_) was evaluated, and the results are displayed and represented graphically (Tables 1, 2 and Fig. 3). For comparison, the IC_50_ values of reference drugs, etoposide and levofloxacin, are displayed. The addition of aldehydic derivatives to compound 2 results in compounds 3a–c exhibiting low cytotoxic activity against the breast cancer cell line MCF-7. Compounds 3a and 3b (4-chlorobenzaldehyde and 4-fluorobenzaldehyde), respectively, displayed high cytotoxic activity against leukemia (L-SR), as compound 3c (4-methoxybenzaldehyde derivative) showed high cytotoxic activity against the liver cancer cell line with an IC_50_ value of 0.43 μM. The addition of isatin derivatives to compound 2 displayed low to moderate cytotoxic activity against selected cancer cell lines. Compounds 4b (5-bromoisatin) exhibited high cytotoxic activity against the liver cancer cell line Hep3B with an IC_50_ value of 8.79 μM in comparison to reference drugs. The open uncyclized compound 5 showed excellent cytotoxic activity against MCF-7, Hep3B and L-SR cell lines (IC_50_ values of 1.4 μM, 3.77 μM, and 0.96 μM, correspondingly). Furthermore, the cyclized pyrazolidone ring compound 6 exhibited low cytotoxic activity against all cancer cell lines. The cyclized iminopyrazolidone and pyrazolidine-3,5-dione compounds 7 and 8, respectively, showed a moderate cytotoxic activity against MCF-7 (IC_50_ values of 4.37 and 3.51 μM) and excellent cytotoxic activity against Hep3B (IC_50_ values of 6.99 and 7.69 μM) compared with the reference drugs. The addition of ethyl cyanoacetate and diethyl malonate to compound 2 results in compounds 9 and 10, respectively, exhibiting low cytotoxic activity against all cancer cells. The addition of carbonyl-containing compounds to compound 10 results in compounds 11a,b and 12a, which exhibited low to moderate cytotoxic activity against cancer cell lines. Levofloxacin amide compounds 13a–d exhibited high to moderate cytotoxic activity against most cancer cell lines in comparison to etoposide and levofloxacin as the reference drugs.

**Table 1:** *In vitro* IC_50_ of the target compounds 3a–c, 4a–c, 5–10, 11a-b, 12a-b, and 13a–d against MCF-7, Hep3B and L-SR cell lines

Compound no.	Cytotoxicity IC_50_ (μM)		
	MCF-7	Hep3B	L-SR
3a	24.77 ± 1.61	24.67 ± 0.81	2.75 ± 0.07
3b	62.99 ± 2.8	11.95 ± 0.39	5.64 ± 0.27
3c	40.59 ± 3.17	0.43 ± 0.01	22.35 ± 1.34
4a	39.1 ± 1.81	18.7 ± 0.87	49.6 ± 2.99
4b	5.59 ± 0.26	8.79 ± 0.41	10.9 ± 0.66
4c	15.8 ± 0.76	36.3 ± 1.68	4.32 ± 0.26
5	1.4 ± 0.06	3.77 ± 0.17	0.96 ± 0.06
6	40.6 ± 1.88	13.1 ± 0.61	28.9 ± 1.75
7	4.37 ± 0.2	6.99 ± 0.32	12.1 ± 0.73
8	3.51 ± 0.16	7.69 ± 0.36	5.49 ± 0.33
9	19.3 ± 0.9	42.2 ± 1.96	23.4 ± 1.41
10	95.4 ± 4.42	37.9 ± 1.76	66.0 ± 3.98
11a	54.5 ± 2.52	80.2 ± 3.71	19.9 ± 1.2
11b	15.5 ± 0.72	14.6 ± 0.67	40.3 ± 2.43
12a	45.4 ± 2.1	23.6 ± 1.09	23.8 ± 1.43
12b	5.21 ± 0.24	17.6 ± 0.81	34.2 ± 2.06
13a	12.1 ± 0.56	3.46 ± 0.16	3.12 ± 0.19
13b	26.8 ± 1.24	11.2 ± 0.52	18.3 ± 1.11
13c	7.26 ± 0.34	1.04 ± 0.05	13.6 ± 0.82
13d	9.59 ± 0.44	24.5 ± 1.14	15.1 ± 0.91
Etoposide	16.32 ± 0.72	21.93 ± 0.72	3.38 ± 0.13
Levofloxacin	2.79 ± 0.16	8.94 ± 0.29	7.38 ± 0.31

**Table 2:** Percentage of Topo2β polymerase inhibition for compound 5 against the human breast cancer cell line MCF-7

Compound no.	MCF-7 (μM)	Topo2β% inhibition
5	1.40	78.30
Etoposide	16.32	63.18
Levofloxacin	2.79	59.80

In conclusion, compound 5 showed high inhibitory activity against the MCF-7 cell line (IC_50_ = 1.4 μM; *cf.* etoposide IC_50_ value = 16.32 μM and levofloxacin IC_50_ value = 2.79 μM). Furthermore, compounds 3c, 4b, 5, 7, 8, 13a and 13c against the Hep3B cell line exhibited quite promising cytotoxic activity with IC_50_ values ranging from 0.43 to 8.79 μM in comparison to etoposide, and levofloxacin 21.93 and 8.94 μM, sequentially. Regarding the effect of compounds 5 and 13a against the L-SR cell lines, their IC_50_ values were 0.96 and 3.12 μM, respectively (*cf.* etoposide IC_50_ value = 3.38 μM and levofloxacin IC_50_ value = 7.38 μM).

### 2.3. Topoisomerase II beta (Topo2β) polymerase inhibition assay

This assay was employed to predict the inhibitory effects of novel compounds on Topo2β. Etoposide and levofloxacin were used as reference drugs. The percentage inhibition on topoisomerase II beta polymerization of the most active compounds against cancer cell lines was measured. Accordingly, compound 5 with good cytotoxic activity against human breast cancer cell line (MCF-7) was tested for inhibiting Topo2β polymerization using etoposide and levofloxacin as reference drugs. Compound 5 has proven to be a Topo2β polymerase inhibitor with 78.30% inhibition against MCF-7, in comparison with 63.18% and 59.80% for etoposide and levofloxacin, sequentially (Table 2). In the case of compounds that revealed excellent cytotoxic activity against the human liver cancer cell line Hep3B, a Topo2β polymerization inhibition assay was carried out for representative compounds 3c, 5, 7, 13a and 13c. Compounds 3c and 5 showed 81.33% and 83.73% inhibition, respectively against the human liver cancer cell line Hep3B, much more potent than etoposide and levofloxacin (73.22% and 73.59%, correspondingly) (Table 3). In addition, compounds 5 has the highest cytotoxic activity against leukemia cell line (L-SR). It showed Topo2β inhibition against leukemia cell line (L-SR) with 80.29% (*cf.* 69.85% and 70.40%) for etoposide and levofloxacin, respectively (Table 4). The selected compounds 3c and 5 with outstanding Topo2β polymerization inhibitory activity have the following features: 4-methoxyphenyl and opened uncyclized levofloxacin hydrazide. Furthermore, they exhibited remarkable inhibitory activity as well as great potencies against Hep3B. Therefore, the liver cancer cell line Hep3B was selected for further assays.

**Table 3:** Percentage of Topo2β polymerase inhibition for 3c, 5, 7, 13a and 13c against the human liver cancer cell line Hep3B

Compound no.	Hep3B (μM)	Topo2β % inhibition
3c	0.43	81.33
5	3.77	83.73
7	6.99	71.92
13a	3.46	68.77
13c	1.04	70.93
Etoposide	21.93	73.22
Levofloxacin	8.94	73.59

**Table 4:** Percentage of Topo2β polymerase inhibition for 5 against the leukemia cell line L-SR

Compound no.	L-SR (μM)	Topo2β% inhibition
5	0.96	80.29
13a	3.12	69.38
Etoposide	3.38	69.85
Levofloxacin	7.38	70.40

### 2.4. Cell cycle analysis

**Fig. 4:**
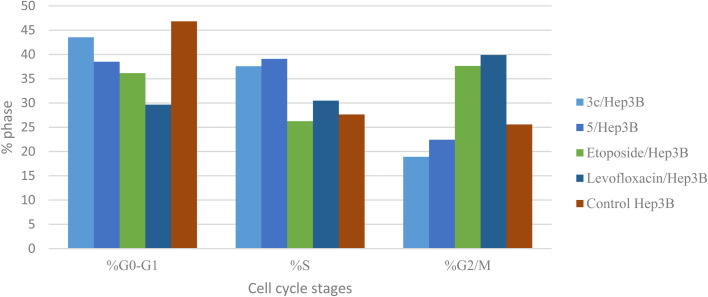
Graphical representation of the cell cycle analysis for compounds 3c, 5, control, etoposide, and levofloxacin against the human liver cancer cell line Hep3B.

**Fig. 5:**
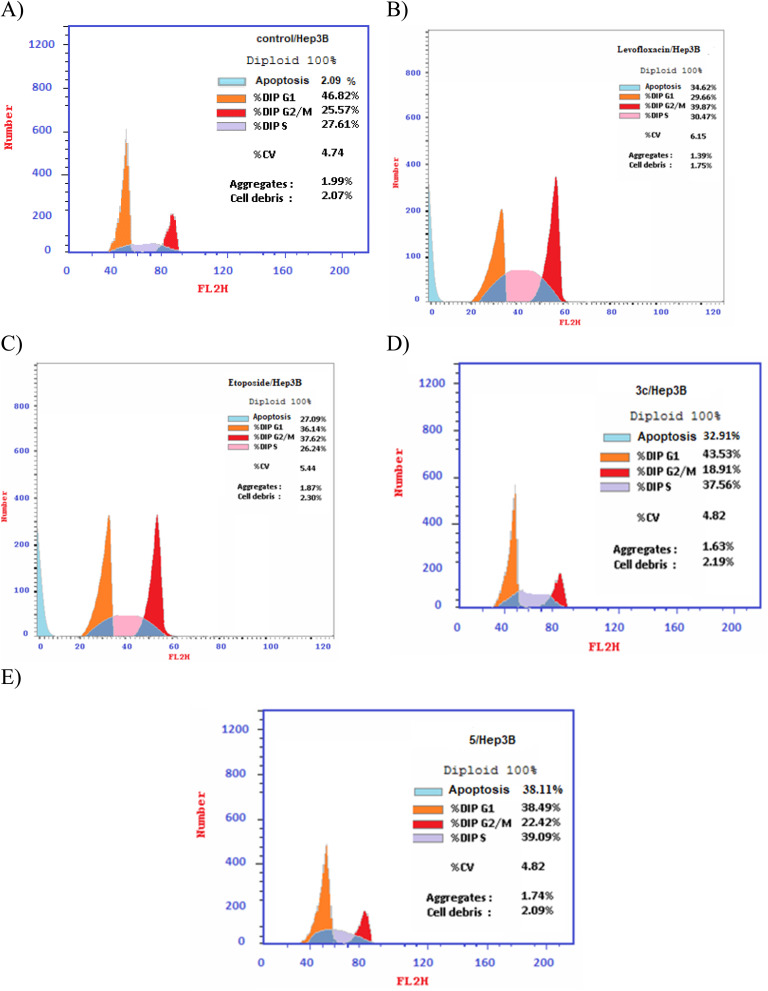
DNA-ploidy flow cytometric analysis for (A) control, (B) levofloxacin, (C) etoposide, (D) compound 3c, and (E) compound 5 against the human liver cancer cell line Hep3B.

Different fluoroquinolone molecules, involving levofloxacin, were found to disturb the normal function of eukaryotic DNA topoisomerases either by stabilization of DNA cleavable complexes to induce the formation of DNA breaks (S phase) or through intercalation of DNA that results in the inhibition of the cell division (G2/M phase) and induction of apoptosis.^49–53^ Interestingly, it was informed that there was a significant increase in the percentage of apoptotic cells primarily at the S and G2/M phases for etoposide^54,55^ and levofloxacin derivatives on Hep3B cells after the modification of the carboxylic acid group of levofloxacin.^25,33^ Accordingly, the cell cycle analysis for the most promising compounds 3c and 5 against the human liver cancer cell line Hep3B was studied, as the carboxylic acid group of levofloxacin was modified, yielding *n*′-benzylidinequinoline-6-carbohydrazide and an open-chained compound respectively. The IC_50_ value of positive controls (etoposide and levofloxacin) used in this assay is 21.93 and 8.94 μM, respectively. Before treatment of Hep3b cells with the targeted compounds, it was noticed that the high percentages of cell cycle arrest of levofloxacin (39.87%) and etoposide (37.62%) were observed at the G2/M phases. Furthermore, the inhibition by etoposide and levofloxacin at the S phase was 26.24% and 30.47%, respectively. After treatment with compounds 3c and 5 with IC_50_ = 0.43 and 3.77 μM, sequentially, there was a significant increase in the percentage of apoptotic cells at the S phase to 37.56% and 39.09%, compared to etoposide (26.24%) and levofloxacin (30.47%). At the G2/M phase, it was showed that the percentages of apoptotic cells after treatment with compounds 3c and 5 decreased (18.91% and 22.42%, respectively), in comparison to etoposide and levofloxacin as reference drugs (37.62% and 39.87%). Only a significant increase in the percentage of apoptotic cells at the G0–G1 phase from 36.14% for etoposide and 29.66% for levofloxacin to 43.53% was detected upon treatment with compound 3c, but it could not inhibit the cell cycle through arresting at the G0–G1 phase as its percentage is less than the negative control Hep3B percentage (46.82%). Hence, it can be concluded that compounds 3c and 5 inhibit the cell cycle proliferation through arresting the cell cycle at the S phase and inducing the cell death (Table 5, Fig. 4 and 5).

**Table 5:** Cell cycle distribution of compounds 3c, 5, control, etoposide, and levofloxacin in Hep3B cells

Compound no.	IC_50_	DNA content		
		%G0–G1	%S	%G2/M
3c/Hep3B	0.43	43.53	**37.56**	18.91
5/Hep3B	7.7	38.49	**39.09**	22.42
Etoposide/Hep3B	21.93	36.14	26.24	**37.62**
Levofloxacin/Hep3B	8.94	29.66	30.47	**39.87**
Control Hep3B	—	46.82	27.61	25.57

### 2.5. Annexin V-FITC/propidium iodide (PI) staining assay

**Fig. 6:**
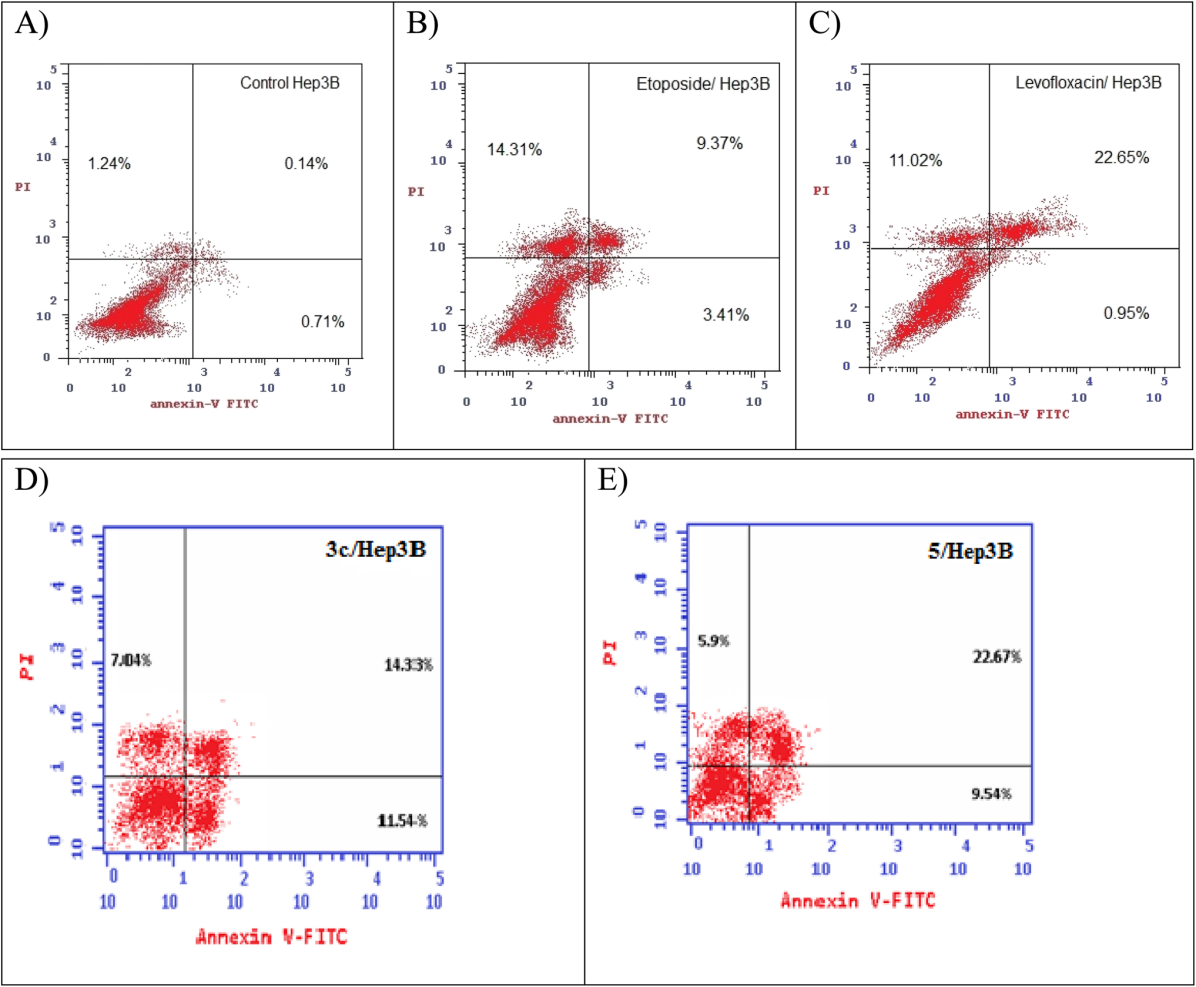
Representative dot plots of the liver cancer cell line Hep3B treated with (A) control, (B) etoposide, (C) levofloxacin, (D) compound 3c, and (E) compound 5 adopting flow cytometric analysis after Annexin V/propidium iodide (PI) staining.

**Fig. 7:**
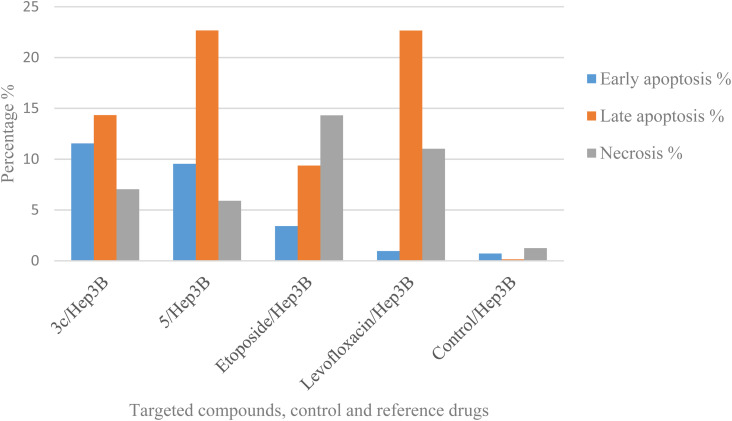
Graphical representation of the percentage of apoptosis and necrosis of compounds 3c, 5, control, etoposide, and levofloxacin in the liver cancer cell line Hep3B.

Using Annexin V fluorescein isothiocyanate (FITC)-conjugate (Annexin V-FITC) is a standard procedure to investigate the apoptosis progression.^56,57^ A biometric flow cytometric analysis was performed for compounds 3c and 5 after treatment of the liver cancer cell line Hep3B with the tested compounds at their detected IC_50_ to ensure their ability to cause apoptosis. As shown in Fig. 6, quadrant plots were divided into four parts: Q1 lower left = total cells, Q2 lower right = early apoptotic cells, Q3 upper right = late apoptotic cells, and Q4 upper left = necrotic cells. The results from these plots showed the best significant rise in the percentage of apoptotic cells (summation of Q2 and Q3) by 25.87 and 32.21 for compounds 3c and 5 respectively compared to control, etoposide, and levofloxacin with apoptotic percentages of 0.85 and 23.60, respectively as illustrated in Table 6, Fig. 6 and 7.

**Table 6:** Effect of compounds 3c, 5, control, etoposide and levofloxacin on apoptosis and necrosis

Compound no.	Total %	Early apoptosis %	Late apoptosis %	Necrosis %
3c/Hep3B	32.91	11.54	14.33	7.04
5/Hep3B	38.11	9.54	22.67	5.9
Etoposide/Hep3B	27.09	3.41	9.37	14.31
Levofloxacin/Hep3B	34.62	0.95	22.65	11.02
Cont. Hep3B	2.09	0.71	0.14	1.24

### 2.6. Caspase-3 analysis

**Fig. 8:**
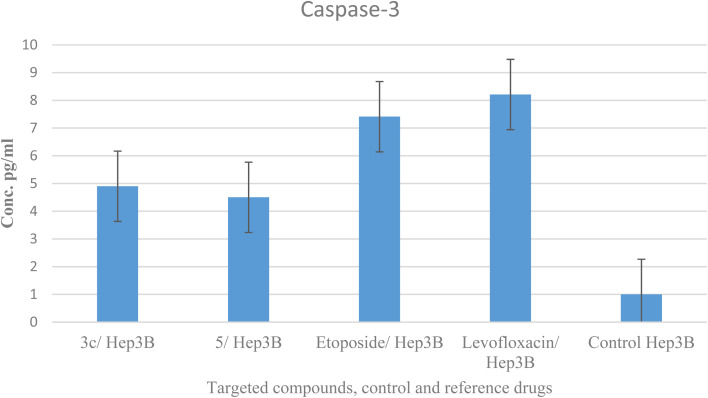
Graphical representation of compounds 3c, 5, control Hep3B and reference drugs on caspase-3.

Caspases are crucial mediators of apoptosis. Among them, caspase-3 is considered as a key effector caspase in inducing cell apoptosis and serves as a marker of programmed cell death.^58^ Compounds 3c and 5 were tested for their effect on caspase-3 levels in the most sensitive liver cancer cell line Hep3B. The treatment of Hep3B cells with compounds 3c and 5 showed elevation in caspase-3 levels by 4.9 and 4.5 folds, respectively in comparison to values 5.5 and 4.7 and one-fold for etoposide, levofloxacin, and control, sequentially. These results supported cell cycle analysis and Annexin V studies and suggest that compounds 3c and 5 induced apoptosis through activation of caspase-3 (Table 7 and Fig. 8).

**Table 7:** Effect of compounds 3c, 5, control, etoposide, and levofloxacin on caspase-3 levels in the liver cancer cell line Hep3B

Compound no.	Caspase-3	
	Conc. Pg mL^−1^	Folds
3c/Hep3B	407.3 ± 7.55	4.9
5/Hep3B	375.8 ± 16.6	4.5
Etoposide/Hep3B	460 ± 11.3	5.5
Levofloxacin/Hep3B	391.7 ± 5.86	4.7
Control Hep3B	55.28 ± 3.25	1

### 2.7. Effect on normal human cell lines

One of the serious problems of cancer chemotherapy is the undesired damage of normal cells. This is attributed to the undistinguishing cytotoxicity of anticancer drugs.^59^ Representative target compounds that were most active against the MCF-7, Hep3B and L-SR cell lines and causing the highest Topo2β percentage inhibition were selected. The following normal cell lines were selected: normal breast MCF-10a, normal liver THLE2 and normal lymphocyte PCS-800-011. Etoposide and levofloxacin were selected as reference drugs. Five representative compounds were evaluated for their toxicity towards the normal liver cell line THLE2, using etoposide and levofloxacin as reference drugs. In addition, only compound 5 was evaluated for its effect on the normal breast cell line MCF-10a, and compounds 5 and 7 were evaluated for their toxic effects on the normal lymphocyte cell line PCS-800-011 *via* an MTT assay, and the IC_50_ doses of all the representative target compounds against normal cell lines selected were calculated. In accordance, compound 5 (IC_50_ = 30.40 μM) has lesser side effects on the normal breast cell line MCF-10a than those of the reference drugs. Regarding the normal liver cell line THLE2, compounds 5 and 7 exhibited IC_50_ = 40.56 μM and 68.52 μM, respectively in comparison with 35.20 and 29.95 for etoposide and levofloxacin, sequentially. Finally, for the normal lymphocyte cell line PCS-800-011, compound 7 (IC_50_ = 32.06 μM) exhibited IC_50_ values exceeding those of levofloxacin, and the results are provided in Table 8.

**Table 8:** Effects of compounds 3c, 5, 7, 13a and 13c on normal human cell lines

Compound no.	Cytotoxicity IC_50_ (μM)		
	THLE2	PCS-800-011	MCF-10a
3c	19.98 ± 0.76	NA	NA
5	40.56 ± 2.28	19.68 ± 1.03	30.40 ± 1.66
7	68.52 ± 3.86	32.06 ± 1.68	NA
13a	29.93 ± 1.68	NA	NA
13c	24.02 ± 1.35	NA	NA
Etoposide	35.20 ± 2.19	37.06 ± 1.79	23.88 ± 1.55
Levofloxacin	29.95 ± 1.52	20.23 ± 0.85	22.25 ± 1.41

### 2.8. Molecular modeling study

**Fig. 9:**
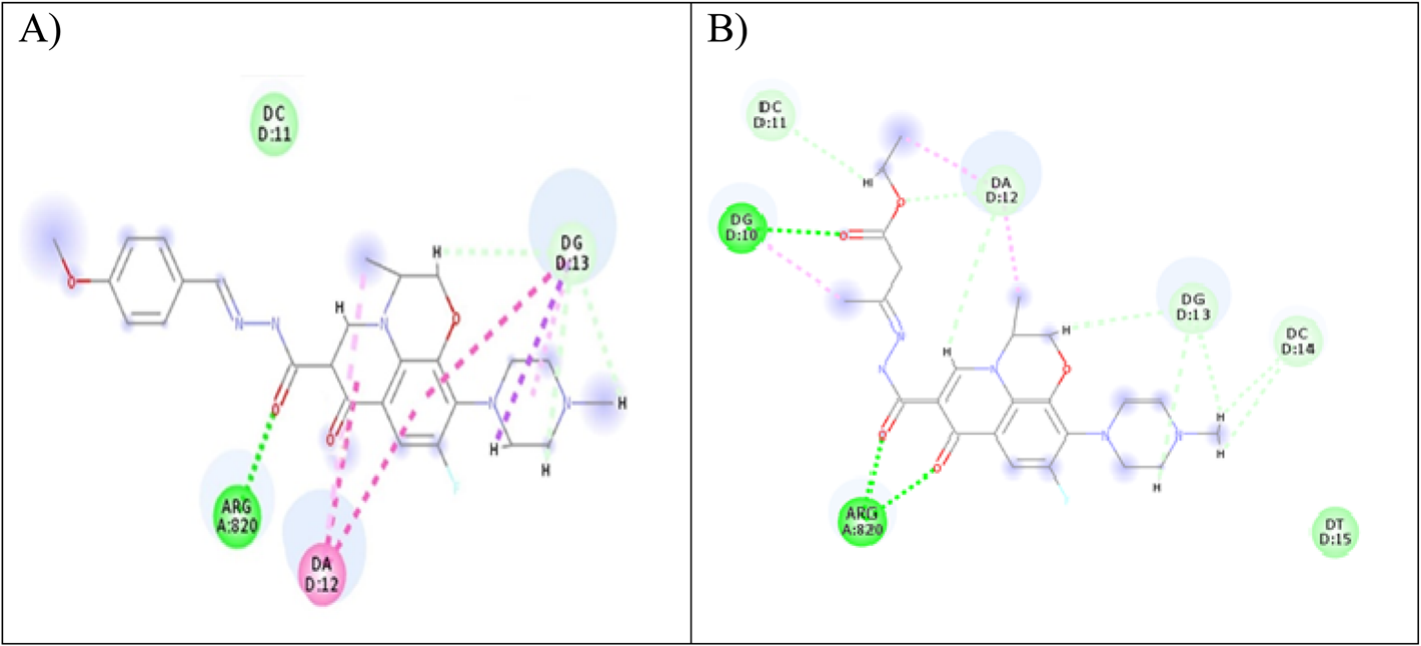
(A) Two-dimensional interaction of compound 3c at Topo2β binding sites (PDB code: 3QX3). (B) Two-dimensional interaction of compound 5 in Topo2β binding sites (PDB code: 3QX3).

Computer-aided drug design is an important tool in designing selective and potent inhibitors.^60^ Docking study can be used to illustrate the molecular interaction of novel compounds at the protein–ligand interface.^61^ A molecular modeling simulation study was conducted to investigate the probable binding modes of the most active compound 5 on Topo2β enzyme of breast, 3c and 5 on Topo2β enzyme of liver cancer cell lines and compound 5 on Topo2β enzyme of leukemia cancer cell lines. In this study, we selected PDB ID: 3QX3^62^ human topoisomerase II beta in complex with DNA and etoposide. DNA chains were retained from the active site. The molecular docking setup was validated by carrying out the docking of etoposide and fluoroquinolones in Topo2β, as reported in a previous study.^28^ It was reported that ASP479, SER480, ARG820, ARG503, LYS456 and GLN778 amino acid residues of Topo2β are involved in binding with fluoroquinolones. Etoposide demonstrated energy score (*S*) = −9.38 kcal mol^−1^ and showed interaction with DNA nucleotides: DG10, DG11, DA12 and DC11 in Topo2β complex, which is essential for binding affinities.^28^ Levofloxacin demonstrated energy score (*S*) = −11.78 kcal mol^−1^ and showed a hydrogen-bond interaction with ASP479 and SER480 in Topo2β complex.^28^ The results obtained from docking imitation are presented in Fig. 9.

Compound 3c demonstrated energy score (*S*) = −29.77 kcal mol^−1^, as it showed one conventional hydrogen bond interaction with ARG820, three carbon–hydrogen bond interactions with DG13, and two pi-alkyl bonds with DG13 and DA12. Furthermore, compound 5 exhibited energy score (*S*) = −20.46 kcal mol^−1^, as it showed three conventional hydrogen bonds, one with DG10 and two with ARG820, three carbon–hydrogen bonds, one with DC11 and two with DA12, and two pi-alkyl bonds with DA12.

### 2.9. Physicochemical properties

**Fig. 10:**
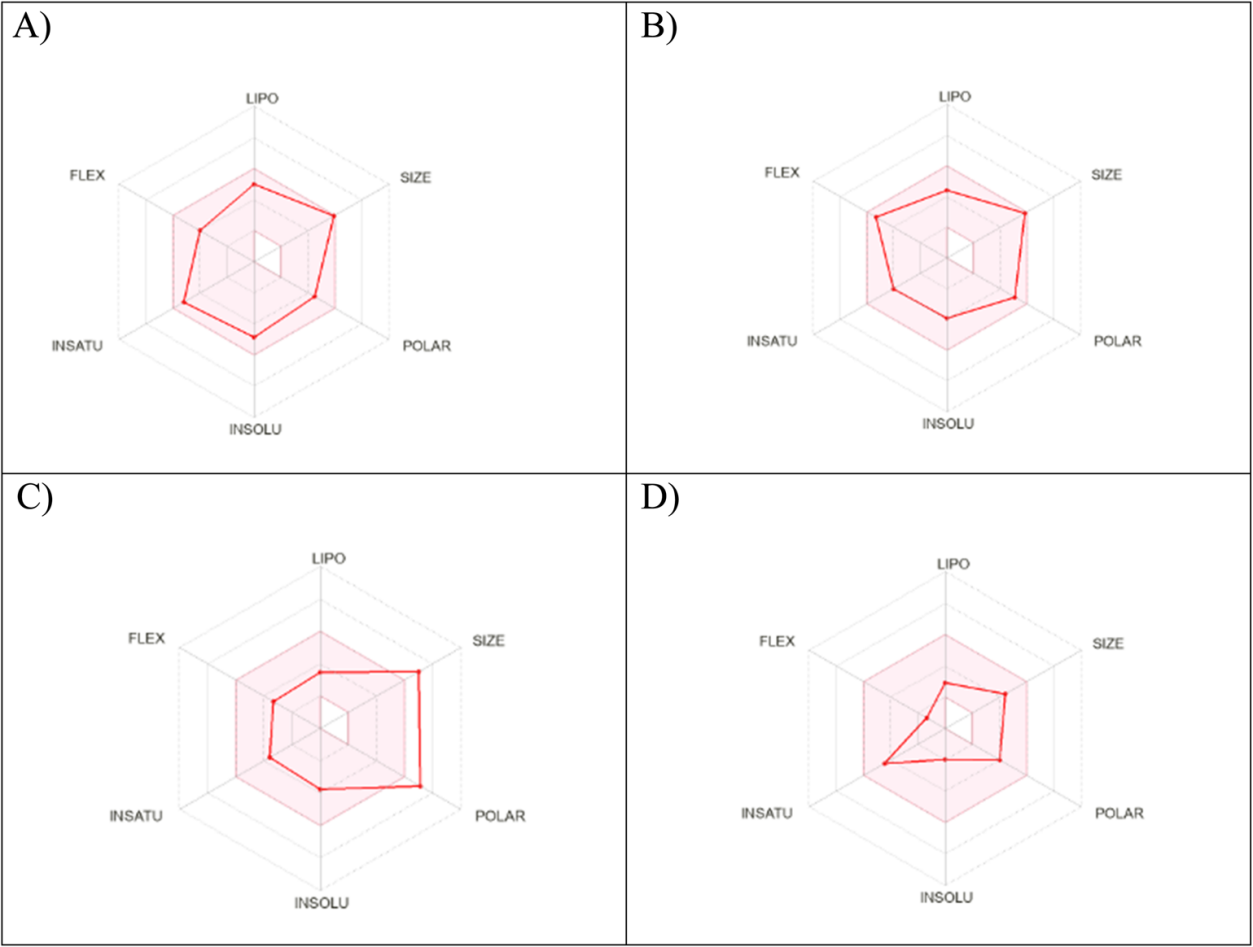
Radar chart showing six physicochemical properties of the tested (A) compound 3c, (B) compound 5, (C) etoposide and (D) levofloxacin.

**Fig. 11:**
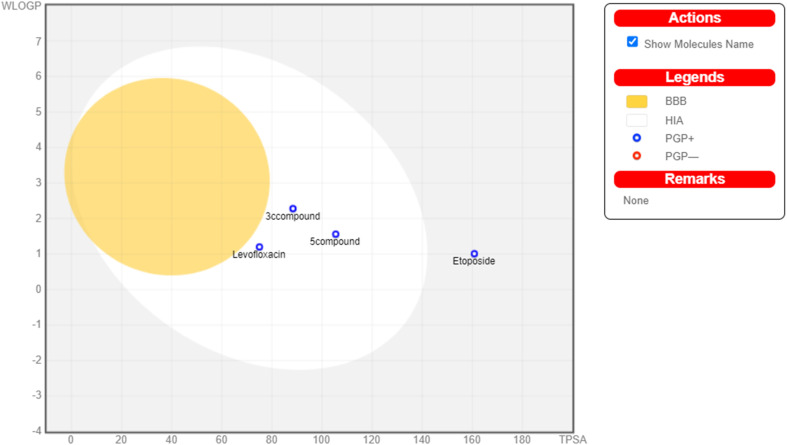
Boiled egg model of the tested compounds 3c, 5, etoposide and levofloxacin.

The physicochemical characteristics of the most promising cytotoxic compounds 3c and 5 on various tested cell lines were investigated compared to etoposide and levofloxacin as reference drugs. The results of the ADME revealed six physicochemical properties: lipophilicity, size, polarity, solubility, saturation, and flexibility. Compounds 3c, 5 and levofloxacin showed no deviation in all physicochemical properties, while etoposide showed a deviation in size and polarity (Fig. 10). Other physicochemical parameters include topological polar surface area, pan assay interference structures, lipophilicity petameter WLOGP, number of rotatable bonds, number of hydrogen bond acceptors, number of hydrogen bond donors, gastrointestinal absorption blood–brain barrier permeability and drug likeness (Table 9). In addition, compounds 3c, 5, etoposide and levofloxacin were good substrates for P-glycoprotein. Furthermore, compounds 3c, 5 and levofloxacin showed high GI absorption as they were located in the boiled egg white, but etoposide showed low GI absorption. Finally, compounds 3c, 5, levofloxacin and etoposide are located outside the boiled egg yolk, so they cannot penetrate the blood–brain barrier (Fig. 11).

**Table 9:** Pharmacokinetic profiles of the investigated compounds 3c, 5, etoposide and levofloxacin

Compound no.	TPSA^a^	PAINS^b^	WLOGP^c^	NRB^d^	HBD^e^	HBA^f^	GI abs.^g^	BBB perm.^h^	Lipinski^i^
3c	88.40	0	2.28	6	1	7	High	No	0 Violation
5	105.47	0	1.56	8	1	8	High	No	0 Violation
Etoposide	160.83	0	1.01	5	3	13	Low	No	0 Violation
Levofloxacin	75.01	0	1.20	2	1	6	High	No	0 Violation

a Topological polar surface rea.

b Pan assay interference structures.

c lipophilicity petameter WLOGP.

d Number of rotatable bonds.

e Number of hydrogen bond acceptors.

f Number of hydrogen bond donor.

g Gastrointestinal absorption.

h Blood–brain barrier permeability.

i Lipinski (drug likeness).

## 3. Experimental

### 3.1. Chemistry

The melting points were determined using a Stuart apparatus, and the values given are uncorrected. IR spectra were recorded using a Shimadzu IR 4000s spectrophotometer (KBr, cm^−1^) at the College of Pharmaceutical Sciences and Drug Manufacturing, Misr University for Science and Technology, Egypt. ^1^H NMR spectra were recorded using a Bruker 400 MHz (Bruker Corp., Billerica, MA, USA) spectrophotometer, Faculty of Pharmacy, Cairo University, Cairo, Egypt and the Centre of Drug Discovery Research & Development, Faculty of Pharmacy, Ain shams University, and a Jeol 500 MHz spectrophotometer at the National Research Center. Chemical shifts were recorded in ppm on a *δ* scale, coupling constants (*J*) were given in Hz and peak multiplicities are designed as follows: s, singlet; d, doublet; t, triplet; m, multiplet. ^13^C NMR spectra were recorded using a Bruker 100 MHz spectrophotometer, Faculty of Pharmacy, Cairo University and the Centre of Drug Discovery Research & Development, Faculty of Pharmacy, Ain shams University, and a Jeol 500 MHz spectrophotometer at the National Research Center. Chemical shifts were recorded in ppm on a *δ* scale. Microanalyses for C, H, and N were carried out at the Regional Center for Mycology and Biotechnology, Faculty of Pharmacy, Al-Azhar University. C, H, and N analysis values were accepted within the range of ± 0.4 of the calculated percentages. The reactions' progress was monitored by TLC using aluminum sheets precoated with UV fluorescent silica gel (MERCK 60F 254), and spots were visualized using a UV Lamp. The used eluting system was methanol : hexane with a ratio of 9.5/0.5 respectively. Scientific microwave MAS-II was used under 300 W microwave irradiation, operating at 80 °C. The evaluation of the cytotoxic activity was performed at VACSERA, Giza, Egypt, using MCF-7, Hep3B and leukemia SR cell lines. Enzyme assay, cell cycle analysis, apoptosis and caspase-3 assay were carried out at VACSERA, Giza, Egypt using an SEB870Hu 96 Tests Enzyme-linked Immunosorbent Assay Kit for Topoisomerase II Beta. The purity of compound 5 was detected by HPLC at the Lambda max of it. Levofloxacin was obtained from Amoun Pharmaceutical Industries Company. Compound 2 was prepared according to the reported procedure,^35^ while the levofloxacin ester intermediate was prepared according to similar reported method.^48^

#### 3.1.1. General procedure of preparation of compounds 3a–c

A mixture of compound 2 (0.375 g, 0.001 mol), substituted carbonyl-containing compounds (0.001 mol) and 3–4 drops of glacial acetic acid in absolute ethanol (20 mL) was heated under reflux for 2–6 h. After cooling, the precipitated solid was filtered, washed with cold ethanol, and crystallized from ethanol to give compounds 3a–c.

##### 3.1.1.1. *N*′-(4-Chlorobenzylidene)-9-fluoro-3-methyl-10-(4-methylpiperazin-1-yl)-7-oxo-2,3-dihydro-7*H*-[1,4]oxazino[2,3,4-*ij*]quinoline-6-carbohydrazide (3a)

Yield, 50.2%; mp = 260–262 °C; IR (KBr) *ν* = 3446 (NH), 3005 (CH aromatic), 2935 (CH aliphatic), 1668, 1640 (2C=O), 1618 (C=N) cm^−1^. ^1^H NMR (DMSO-d_6_) ppm *δ*: 1.45 (d, 3H, *J* = 6.4 Hz, CH_3_ of oxazine ring), 2.23 (s, 3H, CH_3_ of piperazine ring), 2.44 (t, 4H, *J* = 4 Hz, 2CH_2_ of piperazine ring), 3.27 (t, 4H, *J* = 4 Hz, 2CH_2_ of piperazine ring), 4.37 (d, 1H, *J* = 10.4 Hz, CH_2_ of oxazine ring), 4.55 (d, 1H, *J* = 10.8 Hz, CH_2_ of oxazine ring), 4.89–4.91 (m, 1H, CH of oxazine ring), 7.52 (d, 2H, *J* = 7.6 Hz, Ar–H), 7.54 (d, 1H, Ar–H), 7.76 (d, 2H, *J* = 8 Hz, 2Ar–H), 8.41 (s, 1H, N=CH), 8.89 (s, 1H, Ar–H), 13.24 (s, 1H, NH, D_2_O exchangeable). ^13^C NMR (DMSO-d_6_) *δ*: 174.5, 161.6, 154.6, 150.1, 146.9, 145.9, 140.8, 134.9, 133.8, 131.8, 130.9, 129.3, 126.9, 109.6, 103.8, 68.6, 55.8, 54.8, 50.6, 46.5, 18.4. Calcd for C_25_H_25_ClFN_5_O_3_ (497.96): C, 60.30; H, 5.06; N, 14.06. Found: C, 60.47; H, 5.28; N, 14.32.

##### 3.1.1.2. 9-Fluoro-*N*′-(4-fluorobenzylidene)-3-methyl-10-(4-methylpiperazin-1-yl)-7-oxo-2,3-dihydro-7*H*-[1,4]oxazino[2,3,4-*ij*]quinoline-6-carbohydrazide (3b)

Yield, 62.3%; mp = 266–268 °C; IR (KBr) *ν* = 3446 (NH), 3041 (CH aromatic), 2964 (CH aliphatic), 1675 (C=O), 1600 (C=N) cm^−1^. ^1^H NMR (DMSO-d_6_) ppm *δ*: 1.45 (d, 3H, *J* = 6.7 Hz, CH_3_ of oxazine ring), 2.22 (s, 3H, CH_3_ of piperazine ring), 2.42 (t, 4H, *J* = 4 Hz, 2CH_2_ of piperazine ring), 3.26 (t, 4H, *J* = 5 Hz, 2CH_2_ of piperazine ring), 4.37 (d, 1H, *J* = 9.6 Hz, CH_2_ of oxazine ring), 4.56 (d, 1H, *J* = 11.4 Hz, CH_2_ of oxazine ring), 4.90–4.92 (m, 1H, CH of oxazine ring), 7.28–7.32 (m, 2H, 2Ar–H), 7.53 (d, 1H, *J* = 12.5 Hz, Ar–H), 7.79–7.82 (m, 2H, 2Ar–H), 8.41 (s, 1H, N=CH), 8.88 (s, 1H, Ar–H), 13.21 (s, 1H, NH, D_2_O exchangeable).^13^C NMR (DMSO-d_6_) *δ*: 174.5, 164.8, 162.3, 161.4, 157.0, 154.5, 147.1, 145.9, 140.6, 131.5, 129.8, 124.7, 121.8, 116.2, 103.8, 68.5, 55.8, 54.7, 50.6, 46.5, 18.4. Calcd for C_25_H_25_F_2_N_5_O_3_ (481.50): C, 62.36; H, 5.23; N, 14.55. Found: C, 62.59; H, 5.35; N, 14.81.

##### 3.1.1.3. 9-Fluoro-3-methyl-10-(4-methylpiperazin-1-yl)-*N*′-(4-methoxybenzylidene)-7-oxo-2,3-dihydro-7*H*-[1,4]oxazino[2,3,4-*ij*]quinoline-6-carbohydrazide (3c)

Yield, 44.6%; mp = 245–247 °C; IR (KBr) *ν* = 3446 (NH), 3050 (CH aromatic), 2933 (CH aliphatic), 1668, 1643 (2C=O), 1533 (C=N) cm^−1^. ^1^H NMR (DMSO-d_6_) ppm *δ*: 1.45 (d, 3H, *J* = 6.7 Hz, CH_3_ of oxazine ring), 2.23 (s, 3H, CH_3_ of piperazine ring), 2.43 (t, 4H, *J* = 4 Hz, 2CH_2_ of piperazine ring), 3.26 (t, 4H, *J* = 5 Hz, 2CH_2_ of piperazine ring), 3.81 (s, 3H, OCH_3_), 4.36 (d, 1H, *J* = 9.5 Hz, CH_2_ of oxazine ring), 4.55 (d, 1H, *J* = 11.4 Hz, CH_2_ of oxazine ring), 4.90–4.93 (m, 1H, CH of oxazine ring), 7.02 (d, 2H, *J* = 8.8 Hz, 2Ar–H), 7.55 (d, 1H, *J* = 12.5 Hz, Ar–H), 7.69–7.80 (m, 2H, 2Ar–H), 8.34 (s, 1H, N=CH), 8.88 (s, 1H, Ar–H), 13.13 (s, 1H, NH, D_2_O exchangeable). ^13^C NMR (DMSO-d_6_) *δ*: 174.5, 161.2, 157.0, 154.6, 148.0, 145.8, 140.6, 131.6, 129.3, 127.4, 124.8, 121.9, 114.8, 109.8, 103.8, 68.6, 55.8, 54.7, 50.61, 50.57, 46.5, 18.4. Calcd for C_26_H_28_FN_5_O_4_ (493.53): C, 63.27; H, 5.72; N, 14.19. Found: C, 63.04; H, 5.91; N, 14.47.

#### 3.1.2. General procedure of preparation of compounds 4a–c

Compound 2 (0.15 g, 0.0004 mol), each of isatin or its 5-halosubstituted derivatives (0.0004 mol) and 3–4 drops of glacial acetic acid in absolute ethanol (20 mL) were mixed. The reaction mixture was refluxed for 6 h. The solution was cooled to room temperature and the precipitated solid was filtered and crystallized from ethanol to yield compounds 4a–c.

##### 3.1.2.1. 9-Fluoro-3-methyl-10-(4-methylpiperazin-1-yl)-7-oxo-*N*′-(2-oxoindolin-3-ylidene)-2,3-dihydro-7*H*-[1,4]oxazino[2,3,4-*ij*]quinoline-6-carbohydrazide (4a)

Yield, 89.2%; mp <300 °C; IR (KBr) *ν* = 3429 (NH), 3080 (CH aromatic), 2930 (CH aliphatic), 1732, 1678, 1649 (3C=O), 1618 (C=N) cm^−1^. ^1^H NMR (DMSO-d_6_) ppm *δ*: 1.47 (d, 3H, *J* = 9 Hz, CH_3_ of oxazine ring), 2.24 (s, 3H, CH_3_ of piperazine ring), 2.44 (t, 4H, *J* = 4 Hz, 2CH_2_ of piperazine ring), 3.27 (t, 4H, *J* = 4 Hz, 2CH_2_ of piperazine ring), 4.39 (d, 1H, *J* = 9.7 Hz, CH_2_ of oxazine ring), 4.56 (d, 1H, *J* = 10.6 Hz, CH_2_ of oxazine ring), 4.89–4.94 (m, 1H, CH of oxazine ring), 6.90–7.58 (m, 4H, 4Ar–H), 8.43 (d, 1H, *J* = 7.7 Hz, Ar–H), 9.00 (s, 1H, Ar–H), 10.77, 13.77 (s, 2H, 2NH, D_2_O exchangeable). ^13^C NMR (DMSO-d_6_) *δ*: 165.4, 163.0, 161.3, 146.5, 144.1, 142.9, 140.5, 138.2, 133.1, 131.8, 126.3, 124.6, 122.6, 121.2, 111.2, 109.3, 104.4, 104.1, 68.5, 55.7, 55.1, 50.4, 46.4, 18.2. Calcd for C_26_H_25_FN_6_O_4_ (504.51): C, 61.90; H, 4.99; N, 16.66. Found: C, 61.73; H, 5.12; N, 16.80.

##### 3.1.2.2. *N*′-(5-Bromo-2-oxoindolin-3-ylidene)-9-fluoro-3-methyl-10-(4-methylpiperazin-1-yl)-7-oxo-2,3-dihydro-7*H*-[1,4]oxazino[2,3,4-*ij*]quinoline-6-carbohydrazide (4b)

Yield, 72.8%; mp <300 °C; IR (KBr) *ν* = 3446 (NH), 3080 (CH aromatic), 2933 (CH aliphatic), 1732, 1720, 1641 (3C=O), 1612 (C=N) cm^−1^. ^1^H NMR (DMSO-d_6_) ppm *δ*: 1.47 (d, 3H, *J* = 6.5 Hz, CH_3_ of oxazine ring), 2.23 (s, 3H, CH_3_ of piperazine ring), 2.44 (t, 4H, *J* = 4 Hz, 2CH_2_ of piperazine ring), 3.27 (t, 4H, *J* = 4 Hz, 2CH_2_ of piperazine ring), 4.40 (d, 1H, *J* = 8.9 Hz, CH_2_ of oxazine ring), 4.56 (d, 1H, *J* = 10.6 Hz, CH_2_ of oxazine ring), 4.93 (m, 1H, CH of oxazine ring), 6.86–6.90 (m, 1H, Ar–H), 7.51 (d, 1H, *J* = 11 Hz, Ar–H), 7.60 (d, 1H, *J* = 8.9 Hz, Ar–H), 8.64 (s, 1H, Ar–H), 9.01 (s, 1H, Ar–H), 10.90, 13.83 (s, 2H, 2NH, D_2_O exchangeable). ^13^C NMR (DMSO-d_6_) *δ*: 174.6, 165.2, 163.4, 148.8, 146.8, 145.0, 143.2, 140.6, 137.7, 137.3, 135.3, 127.1, 124.8, 117.5, 114.4, 113.2, 108.9, 104.2, 68.6, 55.7, 55.2, 50.4, 46.4, 18.3. Calcd for C_26_H_24_BrFN_6_O_4_ (583.41): C, 53.53; H, 4.15; N, 14.41. Found: C, 53.72; H, 4.39; N, 14.67.

##### 3.1.2.3. *N*′-(5-Chloro-2-oxoindolin-3-ylidene)-9-fluoro-3-methyl-10-(4 methylpiperazin-1-yl)-7-oxo-2,3-dihydro-7*H*-[1,4]oxazino[2,3,4-*ij*]quinoline-6-carbohydrazide (4c)

Yield, 69.6%; mp <300 °C; IR (KBr) *ν* = 3444 (NH), 3050 (CH aromatic), 2930 (CH aliphatic), 1732, 1722, 1680 (3C=O), 1612 (C=N) cm^−1^. ^1^H NMR (DMSO-d_6_) ppm *δ*: 1.47 (d, 3H, *J* = 6.6 Hz, CH_3_ of oxazine ring), 2.25 (s, 3H, CH_3_ of piperazine ring), 2.46 (t, 4H, *J* = 4 Hz, 2CH_2_ of piperazine ring), 3.28 (t, 4H, *J* = 4 Hz, 2CH_2_ of piperazine ring), 4.39 (d, 1H, *J* = 9.2 Hz, CH_2_ of oxazine ring), 4.56 (d, 1H, *J* = 10.6 Hz, CH_2_ of oxazine ring), 4.90–4.95 (m, 1H, CH of oxazine ring), 6.91–6.96 (m, 1H, Ar–H), 7.38 (d, 1H, *J* = 8.3 Hz, Ar–H), 7.48–7.53 (m, 1H, Ar–H), 8.52 (s, 1H, Ar–H), 9.02 (s, 1H, Ar–H), 10.90, 13.85 (s, 2H, 2NH, D_2_O exchangeable). ^13^C NMR (DMSO-d_6_) *δ*: 170.1, 163.1, 159.1, 149.5, 144.9, 143.0, 142.1, 140.7, 137.3, 132.4, 129.0, 126.6, 125.73, 124.6, 112.5, 108.9, 108.0, 104.0, 68.6, 55.7, 55.2, 50.4, 46.3, 18.3. Calcd for C_26_H_24_ClFN_6_O_4_ (538.96): C, 57.94; H, 4.49; N, 15.59. Found: C, 57.80; H, 4.63; N, 15.73.

#### 3.1.3. General procedure of preparation of compounds 5 and 6

A mixture of compound 2 (0.375 g, 0.001 mol) and ethyl acetoacetate (0.17 mL, 0.001 mol) in absolute ethanol (20 mL) was heated under reflux for 12 h and monitored by TLC. The reaction mixture was evaporated. The product was obtained and crystallized from acetonitrile to yield compound 5. Upon continuing refluxing the above reaction to complete 90 h, compound 6 was formed. The reaction mixture was then evaporated, poured on crushed ice, and filtered. The precipitate was crystallized from dichloromethane to afford compound 6.

##### 3.1.3.1. Ethyl 3-(2-(9-fluoro-3-methyl-10-(4-methylpiperazin-1-yl)-7-oxo-2,3-dihydro-7*H*-[1,4]oxazino[2,3,4-*ij*]quinoline-6-carbonyl)hydrazineylidene)butanoate (5)

Yield, 36.9%; mp = 100–102 °C; IR (KBr) *ν* = 3420 (NH), 3040 (CH aromatic), 2960 (CH aliphatic), 1740, 1670, 1660 (3C=O), 1606 (C=N) cm^−1^. ^1^H NMR (DMSO-d_6_) ppm *δ*: 1.22 (t, 3H, *J* = 7.2 Hz, aliphatic OCH_2_CH_3_), 1.43 (d, 3H, *J* = 6.4 Hz, CH_3_ of oxazine ring), 2.02 (s, 3H, N=C–CH_3_), 2.22 (s, 3H, CH_3_ of piperazine ring), 2.42 (t, 4H, *J* = 4 Hz, 2CH_2_ of piperazine ring), 3.25–3.26 (m, 5H, *J* = 4.4 Hz, N=C–CH_2_ + 2CH_2_ of piperazine ring), 3.35 (s, 1H, CH_2_ of piperazine overlapped), 4.12 (q, 2H, *J* = 7.2 Hz, OCH_2_), 4.36 (d, 1H, *J* = 9.6 Hz, CH_2_ of oxazine ring), 4.55 (d, 1H, *J* = 12.8 Hz, CH_2_ of oxazine ring), 4.87–4.89 (m, 1H, CH of oxazine ring), 7.51 (d, 1H, *J* = 6 Hz, Ar–H), 8.87 (s, 1H, Ar–H), 12.91 (s, 1H, NH, D_2_O exchangeable). ^13^C NMR (DMSO-d_6_) *δ*: 174.7, 170.2, 161.2, 154.5, 150.3, 145.7, 140.5, 131.6, 124.7, 121.8, 109.8, 103.9, 68.5, 61.0, 55.7, 54.8, 50.5, 46.4, 44.1, 18.3, 16.9, 14.5. Calcd for C_24_H_30_FN_5_O_5_ (487.53): C, 59.13; H, 6.20; N, 14.37. Found: C, 59.41; H, 6.12; N, 14.50. HPLC purity = 96.87%.

##### 3.1.3.2. 9-Fluoro-3-methyl-6-((3-methyl-5-oxo-4,5-dihydro-1*H*-pyrazol-1-yl)carbonyl)-10-(4-methylpiperazin-1-yl)-2,3-dihydro-7*H*-[1,4]oxazino[2,3,4-*ij*]quinolin-7-one (6)

Yield, 38.3%; mp = 178–180 °C; IR (KBr) *ν* = 3419 (NH), 3055 (CH aromatic), 2978 (CH aliphatic), 1720, 1654 (2C=O) cm^−1^. ^1^H NMR (DMSO-d_6_) ppm *δ*: 1.42 (d, 3H, *J* = 8.8 Hz, CH_3_ of oxazine ring), 1.84, 1.89 (2s, 2H, CH_2_ tautomeric of pyrazolidone ring), 2.02–2.09 (m, 4H, 1H + 3H of CH_3_ of pyrazolidone ring), 2.23 (s, 3H, CH_3_ of piperazine ring), 2.44 (t, 4H, *J* = 4 Hz, 2CH_2_ of piperazine ring), 3.28 (t, 4H, *J* = 4 Hz, 2CH_2_ of piperazine ring), 4.34 (d, 1H, *J* = 6 Hz, CH_2_ of oxazine ring), 4.54 (d, 1H, *J* = 7.2 Hz, CH_2_ of oxazine ring), 4.84–4.87 (m, 1H, CH of oxazine ring), 7.55 (d, 1H, *J* = 9.6 Hz, Ar–H), 8.77 (s, 1H, Ar–H), 10.62, 10.65 (2s, 1H, OH tautomeric, D_2_O exchangeable). ^13^C NMR (DMSO-d_6_) *δ*: 178.3, 174.3, 164.2, 158.9, 154.5, 144.8, 140.6, 131.4, 124.8, 110.2, 68.5, 55.7, 54.5, 50.5, 46.5, 37.0, 23.1, 19.7, 18.4, 11.5. Calcd for C_22_H_24_FN_5_O_4_ (443.48): C, 59.58; H, 5.91; N, 15.79. Found: C, 59.80; H, 6.13; N, 16.03.

#### 3.1.4. General procedure of preparation of compound 7

A mixture of compound 2 (0.375 g, 0.001 mol), ethyl cyanoacetate (0.09 mL, 0.001 mol) and 2–3 drops of piperidine in absolute ethanol (20 mL) was refluxed for 90 h. The solvent was evaporated, and the resulting product was washed with ethanol and crystalized from methanol to give compound 7.

##### 3.1.4.1 9-Fluoro-6-((5-imino-3-oxopyrazolidine-1-yl)carbonyl)-3-methyl-10-(4-methylpiperazin-1-yl)-2,3-dihydro-7*H*-[1,4]oxazino[2,3,4-*ij*]quinolin-7-one (7)

Yield, 49.7%; mp = 101–103 °C; IR (KBr) *ν* = 3419, 3392 (2NH), 3018 (CH aromatic), 2980 (CH aliphatic), 1745, 1654, 1620 (3C=O) cm^−1^. ^1^H NMR (DMSO-d_6_) ppm *δ*: 1.43 (d, 3H, *J* = 5.6 Hz, CH_3_ of oxazine ring), 2.84 (s, 3H, CH_3_ of piperazine ring), 3.37–3.46 (m, 6H, 3CH_2_ of piperazine ring), 3.83 (s, 1H, CH_2_ of piperazine ring), 3.99 (s, 1H, CH_2_ of piperazine ring), 4.13–4.18 (m, 2H, CH_2_ of pyrazolidone ring), 4.36 (d, 1H, *J* = 9.2 Hz, CH_2_ of oxazine ring), 4.54 (d, 1H, *J* = 6.8 Hz, CH_2_ of oxazine ring), 4.85–4.86 (m, 1H, CH of oxazine ring), 7.49 (d, *J* = 12.8 Hz, 1H, Ar–H), 8.75–8.80 (m, 1H, Ar–H), 10.60, 11.86 (2 s, 1H, NH/OH, D_2_O exchangeable). ^13^C NMR (DMSO-d_6_) *δ*: 174.1, 166.4, 164.2, 162.3, 160.1, 144.8, 140.7, 124.6.4, 118.2, 116.1, 110.0, 103.8, 68.6, 62.4, 56.5, 54.8, 49.1, 44.5, 26.8, 18.3. Calcd for C_21_H_23_FN_6_O_4_ (442.45): C, 57.01; H, 5.24; N, 18.99. Found: C, 57.23; H, 5.40; N, 19.17.

#### 3.1.5. General procedure of preparation of compound 8

To a solution of compound 2 (0.225 g, 0.0006 mol) in a sodium ethoxide solution (0.04 g sodium in 20 mL absolute ethanol), diethyl malonate (0.14 g, 0.0009 mol) was added and heated under reflux for 8 h. The reaction mixture was cooled, and the formed precipitate was filtered, washed with water, dried, and crystallized from ethanol to yield compound 8.

##### 3.1.5.1 9-Fluoro-6-((3,5-dioxopyrazolidine-1-yl)carbonyl)-3-methyl-10-(4-methylpiperazin-1-yl)-2,3-dihydro-7*H*-[1,4]oxazino[2,3,4-*ij*]quinolin-7-one (8)

Yield, 37.6%; mp = 228–230 °C; IR (KBr) *ν* = 3419 (NH), 3005 (CH aromatic), 2960 (CH aliphatic), 1740, 1710, 1653, 1620 (4C=O) cm^−1^. ^1^H NMR (DMSO-d_6_) ppm *δ*: 1.41 (d, 3H, *J* = 6.7 Hz, CH_3_ of oxazine ring), 2.23 (s, 3H, CH_3_ of piperazine ring), 2.44 (t, 4H, *J* = 4.3 Hz, 2CH_2_ of piperazine ring), 3.25 (t, 4H, *J* = 4.4 Hz, 2CH_2_ of piperazine ring), 4.33–4.35 (m, 1H, CH of oxazine ring), 4.52–4.54 (m, 1H, CH_2_ of oxazine ring), 4.54–4.57 (m, 2H, CH_2_ of pyrazolidine ring), 4.82–4.87 (m, 1H, CH of oxazine ring), 7.50 (d, 1H, *J* = 12.7 Hz, Ar–H), 8.76 (s, 1H, Ar–H), 10.62 (s, 1H, NH, D_2_O exchangeable).^13^C NMR (DMSO-d_6_) *δ*: 174.2, 164.2, 156.9, 154.4, 144.8, 140.6, 131.4, 124.7, 122.1, 110.0, 103.9, 103.7, 68.5, 60.2, 55.8, 54.5, 50.6, 46.5, 18.4. Calcd for C_21_H_22_FN_5_O_5_ (443.44): C, 56.88; H, 5.00; N, 15.79. Found: C, 57.09; H, 5.14; N, 15.83. MS (*m*/*z*, %): 443.34 [M^+^, 47.50], 79.28 [100.00].

#### 3.1.6. General procedure of preparation of compound 9

A mixture of compound 2 (0.375 g, 0.001 mol), phthalic anhydride (0.15 g, 0.001 mol) and 2–3 drops of glacial acetic acid in absolute ethanol (15 mL) was heated under reflux for 5 h. The reaction mixture was concentrated and allowed to cool. The formed precipitate was filtered, washed with acetonitrile to yield compound 9.

##### 3.1.6.1 9-Fluoro-3-methyl-10-(4-methylpiperazin-1-yl)-7-oxo-*N*-(1,3-dioxoisoindolin-2-yl)-2,3-dihydro-7*H*-[1,4]oxazino[2,3,4-*ij*]quinoline-6-carboxamide (9)

Yield, 61.3%; mp = 138–140 °C; IR (KBr) *ν* = 3431 (NH), 3032 (CH aromatic), 2980 (CH aliphatic), 1735, 1654, 1620 (3C=O) cm^−1^. ^1^H NMR (DMSO-d_6_) ppm *δ*: 1.43 (d, 3H, *J* = 12.4 Hz, CH_3_ of oxazine ring), 2.64 (s, 3H, CH_3_ of piperazine ring), 3.00 (t, 4H, *J* = 4 Hz, 2CH_2_ of piperazine ring), 3.41 (s, 4H, *J* = 4 Hz, 2CH_2_ of piperazine ring), 4.38 (d, 1H, *J* = 11.6 Hz, CH_2_ of oxazine ring), 4.55 (d, 1H, *J* = 11.6 Hz, CH_2_ of oxazine ring), 4.85–4.88 (m, 1H, CH of oxazine ring), 7.48–7.60 (m, 3H, 3Ar–H), 7.93–7.99 (m, 1H, Ar–H), 8.14 (m, 1H, Ar–H), 8.77 (s, 1H, Ar–H), 10.61, 11.68 (2s, 1H, NH, D_2_O exchangeable). ^13^C NMR (DMSO-d_6_) *δ*: 174.2, 172.7, 168.9, 165.6, 144.8, 135.6, 135.1, 133.1, 132.7, 130.9, 130.4, 124.7, 124.1, 122.6, 108.5, 103.9, 68.7, 56.6, 54.9, 48.8, 44.3, 18.9. Calcd for C_26_H_24_FN_5_O_5_ (505.51): C, 61.78; H, 4.79; N, 13.85. Found: C, 61.52; H, 4.95; N, 14.09. MS (*m*/*z*, %): 505.75 [M^+^, 62.61], 189.33 [100.00].

#### 3.1.7. General procedure of preparation of compound 10

A mixture of compound 2 (0.2 g, 0.0005 mol), potassium thiocyanate (0.1 g, 0.001 mol) and concentrated hydrochloric acid (0.18 mL) in absolute ethanol (15 mL) was heated under reflux for 6 h. The obtained precipitate was filtered, dried, and washed with cold ethanol to give compound 10.

##### 3.1.7.1 2-[(9-Fluoro-3-methyl-10-(4-methylpiperazin-1-yl)-7-oxo-2,3-dihydro-7*H*-[1,4]oxazino[2,3,4-*ij*]quinoline-6-carbonyl)]hydrazin-1-carbothioamide (10)

Yield, 64.4%; mp >300 °C; IR (KBr) *ν* = 3444, 3419 (2NH), 3385 (NH_2_), 3050 (CH aromatic), 2960 (CH aliphatic), 1670 (C=O) cm^−1^. ^1^H NMR (DMSO-d_6_) ppm *δ*: 1.44 (d, 3H, *J* = 4 Hz, CH_3_ of oxazine ring), 2.80 (s, 3H, CH_3_ of piperazine ring), 3.15 (t, 2H, *J* = 4 Hz, CH_2_ of piperazine), 3.43–3.64 (m, 6H, 3CH_2_ of piperazine ring), 4.38 (d, 1H, *J* = 9.6 Hz, CH_2_ of oxazine ring), 4.58 (d, 1H, *J* = 10.6 Hz, CH_2_ of oxazine ring), 4.90–4.92 (m, 1H, CH of oxazine ring), 7.57 (d, 1H, *J* = 12.2 Hz, Ar–H), 8.83 (s, 1H, Ar–H), 7.89, 9.40, 11.20, 11.39 (4 broad s, 4H, NH/NH_2_, D_2_O exchangeable). ^13^C NMR (DMSO-d_6_) *δ*: 174.2, 156.7, 154.2, 145.7, 140.9, 130.0, 124.6, 122.8, 109.7, 104.0, 68.8, 56.5, 54.6, 53.5, 47.6, 42.8, 18.5. Calcd for C_19_H_23_FN_6_O_3_S (434.49): C, 52.52; H, 5.34; N, 19.34. Found: C, 52.79; H, 5.61; N, 19.12.

#### 3.1.8. General procedure of preparation of compound 11a,b

A mixture of compound 2 (0.15 g, 0.0004 mol), either 4-chlorobenzaldehyde or 4-methoxy benzaldehyde (0.0004 mol) and anhydrous sodium acetate (0.03 g, 0.0004 mol) in absolute ethanol (15 mL) was heated under reflux for 24 h. The obtained precipitate was filtered, dried, and washed with ethanol to afford compounds 11a,b.

##### 3.1.8.1. *N*-(4-Chlorobenzylidene)-2-[(9-fluoro-3-methyl-10-(4-methylpiperazin-1-yl)-7-oxo-2,3-dihydro-7*H*-[1,4]oxazino[2,3,4-*ij*]quinoline-6-carbonyl)]hydrazine-1-carbothioamide (11a)

Yield, 76.3%; mp >300 °C; IR (KBr) *ν* = 3446, 3431 (2NH), 3082 (CH aromatic), 2991 (CH aliphatic), 1678, 1647 (2C=O), 1608 (C=N) cm^−1^. ^1^H NMR (DMSO-d_6_) ppm *δ*: 1.45 (d, 3H, *J* = 6.4 Hz, CH_3_ of oxazine ring), 2.80 (s, 3H, CH_3_ of piperazine ring), 3.18 (broad s, 2H, CH_2_ of piperazine ring), 3.35 (s, 2H, CH_2_ of piperazine overlapped), 3.56 (t, 4H, *J* = 4 Hz, 2CH_2_ of piperazine ring), 4.40 (d, 1H, *J* = 9.2 Hz, CH_2_ of oxazine ring), 4.59 (d, 1H, *J* = 10.4 Hz, CH_2_ of oxazine ring), 4.91–4.95 (m, 1H, CH of oxazine ring), 7.52 (d, 2H, *J* = 8.8 Hz, 2Ar–H), 7.57 (d, 1H, *J* = 12 Hz, Ar–H), 7.77 (d, 2H, *J* = 8.8 Hz, 2Ar–H), 8.42 (s, 1H, N=CH), 8.92 (s, 1H, Ar–H), 11.32, 13.20 (s, 2H, 2NH, D_2_O exchangeable). ^13^C NMR (DMSO-d_6_) *δ*: 174.4, 161.5, 156.8, 154.3, 147.1, 146.0, 141.0, 134.9, 133.7, 129.3, 129.3, 124.7, 122.7, 109.75, 104.0, 103.8, 68.8, 54.8, 53.6, 47.6, 42.9, 18.4. Calcd for C_26_H_26_ClFN_6_O_3_S (557.04): C, 56.06; H, 4.70; N, 15.09. Found: C, 56.30; H, 4.87; N, 15.26.

##### 3.1.8.2. 2-[(9-Fluoro-3-methyl-10-(4-methylpiperazin-1-yl)-7-oxo-2,3-dihydro-7*H*-[1,4]oxazino[2,3,4-*ij*]quinoline-6-carbonyl)]*N*-(4-methoxybenzylidene)hydrazine-1-carbothioamide (11b)

Yield, 36.2%; mp >300 °C; IR (KBr) *ν* = 3523, 3446 (2NH), 3030 (CH aromatic), 2989 (CH aliphatic), 1668, 1650 (2C=O), 1604 (C=N) cm^−1^. ^1^H NMR (DMSO-d_6_) ppm *δ*: 1.45 (d, 3H, *J* = 8.8 Hz, CH_3_ of oxazine ring), 2.27 (s, 3H, CH_3_ of piperazine ring), 3.29 (t, 5H, CH_2_ of piperazine ring), 3.35 (s, 3H, CH_2_ of piperazine overlapped), 3.82 (s, 3H, OCH_3_), 4.37 (d, 1H, *J* = 10 Hz, CH_2_ of oxazine ring), 4.56 (d, 1H, *J* = 12.4 Hz, CH_2_ of oxazine ring), 4.91–4.93 (m, 1H, CH of oxazine ring), 7.03 (d, 2H, *J* = 9.6 Hz, 2Ar–H), 7.55 (d, 1H, *J* = 10 Hz, Ar–H), 7.71 (d, 2H, *J* = 11.6 Hz, Ar–H), 8.35 (s, 1H, N=CH), 8.89 (s, 1H, Ar–H), 13.13 (s, 1H, NH, D_2_O exchangeable). ^13^C NMR (DMSO-d_6_) *δ*: 174.5, 161.3, 161.3, 154.6, 148.1, 145.8, 140.7, 140.7, 131.6, 129.3, 127.4, 124.8, 121.9, 121.86, 114.8, 109.8, 103.8, 68.6, 55.8, 54.7, 50.4, 46.4, 18.4. Calcd for C_27_H_29_FN_6_O_4_S (552.63): C, 58.68; H, 5.29; N, 15.21. Found: C, 58.92; H, 5.46; N, 15.39.

#### 3.1.9. General procedure of preparation of compound 12a,b

Compound 2 (0.15 g, 0.0004 mol) was mixed with the appropriate substituted acetophenone derivative (0.0004 mol) and anhydrous sodium acetate (0.03 g, 0.0004 mol) in absolute ethanol (15 mL). The reaction mixture was heated under reflux for 24 h. The obtained precipitate was filtered, dried, and washed with ethanol to give compounds 12a,b.

##### 3.1.9.1. 2-[(9-Fluoro-3-methyl-10-(4-methylpiperazin-1-yl)-7-oxo-2,3-dihydro-7*H*-[1,4]oxazino[2,3,4-*ij*]quinoline-6-carbonyl)]-*N*-(1-(4-methoxyphenyl)ethylidene)hydrazine-1-carbothioamide (12a)

Yield, 75%; mp >300 °C; IR (KBr) *ν* = 3444, 3419 (2NH), 3043 (CH aromatic), 2987 (CH aliphatic), 1660, 1640 (2C=O), 1597 (C=N) cm^−1^. ^1^H NMR (DMSO-d_6_) ppm *δ*: 1.45 (d, 3H, *J* = 6.8 Hz, CH_3_ of oxazine ring), 2.35 (s, 3H, N=C^_^CH_3_), 2.67 (s, 3H, CH_3_ of piperazine ring), 3.11 (broad s, 3H, CH_2_ of piperazine ring), 3.35 (s, 2H, CH_2_ of piperazine overlapped), 3.52 (broad s, 3H, CH_2_ of piperazine ring), 3.81 (s, 3H, OCH_3_), 4.39 (d, 1H, *J* = 9.6 Hz, CH_2_ of oxazine ring), 4.58 (d, 1H, *J* = 10.8 Hz, CH_2_ of oxazine ring), 4.95–4.96 (m, 1H, CH of oxazine ring), 7.00 (d, 2H, *J* = 8.8 Hz, 2Ar–H), 7.62–7.65 (m, 1H, Ar–H), 7.81 (d, 2H, *J* = 8.8 Hz, 2Ar–H), 8.94 (s, 1H, CH Ar), 10.42, 13.10 (s, 2H, 2NH, D_2_O exchangeable). Calcd for C_28_H_31_FN_6_O_4_S (566.65): C, 59.35; H, 5.51; N, 14.83. Found: C, 59.47; H, 5.62; N, 15.04.

##### 3.1.9.2. *N*-(1-(3,4-Dimethoxyphenyl)ethylidene)-2-[(9-fluoro-3-methyl-10-(4-methylpiperazin-1-yl)-7-oxo-2,3-dihydro-7*H*-[1,4]oxazino[2,3,4-*ij*]quinoline-6-carbonyl)]hydrazine-1-carbothioamide (12b)

Yield, 71.2%; mp = 222–224 °C; IR (KBr) *ν* = 3523, 3446 (2NH), 3080 (CH aromatic), 2990 (CH aliphatic), 1668, 1650 (2C=O) cm^−1^. ^1^H NMR (DMSO-d_6_) ppm *δ*: 1.42 (d, 3H, *J* = 6.8 Hz, CH_3_ of oxazine ring), 1.85 (s, 3H, N=C^_^CH_3_), 2.22 (s, 3H, CH_3_ of piperazine ring), 2.43 (t, 4H, *J* = 12.8 Hz, 2CH_2_ of piperazine ring), 3.26–3.27 (m, 6H, 2OCH_3_), 3.81 (t, 4H, *J* = 4 Hz, 2CH_2_ of piperazine ring), 4.35 (d, 1H, *J* = 10.4 Hz, CH_2_ of oxazine ring), 4.54 (d, 1H, *J* = 10.8 Hz, CH_2_ of oxazine ring), 4.87–4.88 (m, 1H, CH of oxazine ring), 7.00 (d, 1H, *J* = 8.4 Hz, Ar–H), 7.37–7.43 (m, 1H, Ar–H), 7.50 (s, 1H, Ar–H), 7.54 (d, 1H, *J* = 5.6 Hz, Ar–H), 8.78 (s, 1H, Ar–H), 9.38, 11.25 (s, 2H, 2NH, D_2_O exchangeable). ^13^C NMR (DMSO-d_6_) *δ*: 188.5, 179.8, 175.9, 168.6, 165.8, 162.1, 156.7, 154.5, 152.9, 140.6, 135.4, 124.7, 121.6, 120.5, 114.8, 111.3, 104.3, 103.5, 68.6, 55.7, 54.6, 50.6, 50.5, 46.5, 23.6, 18.4. Calcd for C_29_H_33_FN_6_O_5_S (596.68): C, 58.38; H, 5.57; N, 14.08. Found: C, 58.50; H, 5.69; N, 14.31.

#### 3.1.10. General procedure of preparation of compound 13a–d

Ethyl chloroformate (0.095 mL, 0.001 mol) and triethylamine (0.167 mL, 0.0012 mol) were added to levofloxacin (0.361 g, 0.001 mol) in 10 mL of methanol. The reaction mixture was stirred for about 30 min in an ice bath. The appropriate secondary amine (0.0015 mol) was added with continuous stirring for further 12 h. The reaction solvent was evaporated. The formed precipitate was obtained and boiled in petroleum ether to afford compounds 13a–d.

##### 3.1.10.1. 9-Fluoro-3-methyl-10-(4-methylpiperazin-1-yl)-6-((piperidin-1-yl)carbonyl)-2,3-dihydro-7*H*-[1,4]oxazino[2,3,4-*ij*]quinolin-7-one (13a)

Yield, 39.7%; mp = 238–240 °C; IR (KBr) *ν* = 3080 (CH aromatic), 2935 (CH aliphatic), 1718, 1620 (2C=O) cm^−1^. ^1^H NMR (DMSO-d_6_) ppm *δ*: 1.57 (d, 3H, *J* = 4 Hz, CH_3_ of oxazine ring), 1.87 (s, 3H, CH_3_ of piperazine ring), 2.54 (s, 6H, 3CH_2_ of piperidine ring), 3.13 (broad s, 2H, CH_2_ of piperazine ring), 3.33–3.40 (m, 6H, 3CH_2_ of piperazine ring), 3.87 (s, 2H, CH_2_ of oxazine ring), 4.37–4.43 (m, 1H, CH of oxazine ring), 4.58 (broad s, 4H, 2CH_2_ of piperidine ring), 7.60 (d, 1H, *J* = 8 Hz, Ar–H), 8.60 (s, 1H, Ar–H). ^13^C NMR (DMSO-d_6_) *δ*: 177.1, 172.9, 167.4, 156.8, 155.1, 145.4, 144.8, 139.5, 133.2, 124.8, 120.4, 109.5, 107.9, 105.0, 68.3, 55.6, 54.9, 50.6, 46.4, 44.7, 22.7, 18.4. Calcd for C_23_H_29_FN_4_O_3_ (428.51): C, 64.47; H, 6.82; N, 13.08. Found: C, 64.70; H, 6.95; N, 13.31.

##### 3.1.10.2. 9-Fluoro-3-methyl-10-(4-methylpiperazin-1-yl)-6-((morpholin-4-yl)carbonyl)-2,3-dihydro-7*H*-[1,4]oxazino[2,3,4-*ij*]quinolin-7-one (13b)

Yield, 46.5%; mp = 235–237 °C; IR (KBr) *ν* = 3041 (CH aromatic), 2935 (CH aliphatic), 1720, 1624 (2C=O) cm^−1^. ^1^H NMR (DMSO-d_6_) ppm *δ*: 1.58 (d, 3H, CH_3_ of oxazine ring), 2.36 (s, 3H, CH_3_ of piperazine ring), 2.56 (s, 6H, 3CH_2_ of piperazine ring), 3.34–3.40 (m, 8H, 4CH_2_ of morpholine ring), 3.86 (s, 2H, CH_2_ of piperazine ring), 4.37–4.56 (m, 3H, CH_2_ of oxazine ring + CH of oxazine ring), 7.59 (d, 1H, *J* = 12 Hz, Ar–H), 8.57 (s, 1H, Ar–H). ^13^C NMR (DMSO-d_6_) *δ*: 177.1, 172.8, 167.1, 166.2, 157.0, 155.1, 145.3, 144.7, 139.5, 133.2, 124.8, 120.4, 107.8, 105.2, 68.3, 55.7, 55.6, 50.58, 50.5, 46.4, 18.4. Calcd for C_22_H_27_FN_4_O_4_ (430.48): C, 61.38; H, 6.32; N, 13.02. Found: C, 61.60; H, 6.46; N, 13.29.

##### 3.1.10.3. 9-Fluoro-3-methyl-10-(4-methylpiperazin-1-yl)-6-((4-methylpiperazin-1-yl)carbonyl)-2,3-dihydro-7*H*-[1,4]oxazino[2,3,4-*ij*]quinolin-7-one (13c)

Yield, 45.1%; mp = 239–241 °C; IR (KBr) *ν* = 3080 (CH aromatic), 2968 (CH aliphatic), 1718, 1620 (2C=O) cm^−1^. ^1^H NMR (DMSO-d_6_) ppm *δ*: 1.58 (d, 3H, *J* = 4 Hz, CH_3_ of oxazine ring), 2.31 (s, 3H, CH_3_ of piperazine ring), 2.36 (s, 3H, CH_3_ of piperazine ring), 2.49–2.60 (m, 8H, 4CH_2_ of piperidine ring), 3.33–3.41 (m, 8H, 4CH_2_ of piperazine ring), 3.87 (s, 2H, CH_2_ of oxazine ring), 4.32–4.43 (m, 1H, CH of oxazine ring), 7.61 (d, 1H, *J* = 8 Hz, Ar–H), 8.58 (s, 1H, Ar–H). ^13^C NMR (DMSO-d_6_) *δ*: 177.1, 167.2, 144.8, 139.7, 133.2, 124.8, 123.9, 120.4, 109.6, 107.8, 105.0, 68.3, 55.7, 54.8, 54.0, 53.3, 52.0, 50.6, 46.1, 18.4. Calcd for C_23_H_30_FN_5_O_3_ (443.52): C, 62.29; H, 6.82; N, 15.79. Found: C, 62.58; H, 7.10; N, 16.05.

##### 3.1.10.4. 9-Fluoro-3-methyl-10-(4-methylpiperazin-1-yl)-6-((4-phenylpiperazin-1-yl)carbonyl)-2,3-dihydro-7*H*-[1,4]oxazino[2,3,4-*ij*]quinolin-7-one (13d)

Yield, 31.7%; mp = 246–248 °C; IR (KBr) *ν* = 3030 (CH aromatic), 2966 (CH aliphatic), 1718, 1620 (2C=O) cm^−1^. ^1^H NMR (DMSO-d_6_) ppm *δ*: 1.57 (d, 3H, *J* = 16 Hz, CH_3_ of oxazine ring), 2.37 (s, 3H, CH_3_ of piperazine ring), 3.19–3.28 (m, 8H, 4CH_2_ of piperidine ring), 3.37–3.42 (m, 8H, 4CH_2_ of piperazine ring), 3.89 (s, 2H, CH_2_ of oxazine ring), 4.02–4.55 (m, 1H, CH of oxazine ring), 6.92–6.99 (m, 5H, 5CH Ar), 7.65 (d, 1H, *J* = 8 Hz, Ar–H), 8.61 (s, 1H, Ar–H). ^13^C NMR (DMSO-d_6_) *δ*: 177.2, 167.3, 157.1, 151.2, 145.4, 144.7, 142.3, 139.5, 129.3, 124.8, 120.5, 117.2, 116.6, 108.0, 105.2, 68.2, 55.7, 55.6, 50.6, 49.2, 46.4, 45.2, 18.4. Calcd for C_28_H_32_FN_5_O_3_ (505.59): C, 66.52; H, 6.38; N, 13.85. Found: C, 66.41; H, 6.52; N, 14.11.

### 3.2. Cytotoxic activity

The newly synthesized compounds were evaluated for their *in vitro* growth inhibition of three human tumor cell lines representing different tumor types, namely, breast adenocarcinoma (MCF-7), liver hepatocellular carcinoma (Hep3B) and leukemia-SR. Etoposide and levofloxacin were taken as reference drugs, and an MTT assay was applied. Moreover, the toxic effect of representative target compounds was evaluated on normal breast (MCF10a), normal liver (THLE2) and normal lymphocyte (PCS-800-011) cell lines using an MTT assay. The *in vitro* IC_50_ antitumor activity for the newly synthesized compounds was established in VACSERA, Egypt. The breast tumor cell line (MCF-7), hepatic tumor cell line (Hep3B) and leukemia-SR cell line were obtained from American Type Culture Collection. The cells were cultured using Dulbecco's modified Eagle's medium (DMEM) (Invitrogen/Life Technologies) supplemented with 10% fetal bovine serum (FBS) (Hyclone), 10 μg mL^−1^ of insulin (Sigma), and 1% penicillin–streptomycin. All chemicals and reagents were purchased from Sigma-Aldrich or Invitrogen. For 24 hours before to the MTT experiment, plate cells (densities of 1.2–1.8 × 10 000 cells per well) were layered in a 96-well plate containing 100 μL of the tested chemical per well and 100 μL of full growth media.

#### 3.2.1. Cell culture protocol

The culture medium was transferred to a centrifuge tube. The cell layer was rinsed with 0.25% (w/v) Trypsin 0.53 mM EDTA solution to remove all traces of serum, which contained Trypsin inhibitor. Then 2.0 to 3.0 mL of Trypsin EDTA solution was added to the flask, and the cells were observed using an inverted microscope until the cell layer became dispersed (usually within 5–15 min). After that, 6.0 to 8.0 mL of complete growth medium was added and the cells were aspirated by gently pipetting. The cell suspension was transferred to the centrifuge tube with the medium and cells from step 1 and centrifuged at approximately 125 g for 5–10 min. The supernatant was discarded. The cell pellet was re-suspended in a fresh growth medium. Appropriate aliquots of the cell suspension were added to new culture vessels. The cultures were incubated at 37 °C for 24 h. After treatment of cells with the serial concentrations of the compound to be tested, incubation was carried out for 48 h at 37 °C, and then the plates were examined using an inverted microscope and processed for the MTT assay.

#### 3.2.2. MTT assay protocol

The MTT method of monitoring *in vitro* cytotoxicity is well suited for use with multiwell plates.^63^ For the best results, the cells in the log phase of growth should be employed and the final cell number should not exceed 106 cells per cm^2^. Each test should include a blank containing a complete medium without cells. Cultures from the incubator were transferred into a laminar flow hood or other sterile work area. Each vial of MTT [M-5655] was reconstituted for use with 3 mL of medium or balanced salt solution without phenol red and serum. Reconstituted MTT was added in an amount equal to 10% of the culture medium volume. Cultures were returned to the incubator for 2–4 h depending on the cell type and maximum cell density. An incubation period of 2 h is generally adequate but may be lengthened for low cell densities or cells with lower metabolic activity. The incubation times should be consistent when making comparisons. After the incubation period, the cultures were removed from the incubator, and the resulting formazan crystals were dissolved by adding an amount of MTT solubilization solution [M-8910] equal to the original culture medium volume and gently mixed in a gyratory shaker to enhance dissolution. Occasionally, especially in dense cultures, pipetting up and down may be required to completely dissolve the MTT formazan crystals. The absorbance was spectrophotometrically measured at a wavelength of 570 nm. The background absorbance of multiwell plates was measured at 690 nm and subtracted from the 450 nm measurement. Tests performed in multiwell plates might be read using an appropriate type of plate reader or the contents of individual wells might be transferred to appropriate size cuvettes for spectrophotometric measurement.

### 3.3. *In vitro* topoisomerase II beta polymerase inhibition assay

This assay employs a quantitative sandwich enzyme immunoassay technique. Target compounds were selected to be evaluated against topoisomerase II [MBS#942146], using a human DNA topoisomerase II beta (Topo2β) ELISA kit according to the manufacturer's instructions. Antibody specific for Topo2β was pre-coated onto a microplate. Standards and samples were pipetted into the wells and any Topo2β present was bound by the immobilized antibody. After removing any unbound substances, a biotin-conjugated antibody specific for Topo2β was added to the wells. After washing, avidin-conjugated Horseradish Peroxidase (HP) was added to the wells. Following washing to remove any unbound avidin–enzyme reagent, a substrate solution was added to the wells and color develops in proportion to the amount of Topo2β bound in the initial step. The color development was stopped, and the intensity of the color was measured. The minimum detectable dose of human Topo2β is typically less than 4.69 pg mL^−1^. The sensitivity of this assay or lower limit of detection (LLD) is defined as the lowest protein concentration that could be differentiated from zero. The mean OD value of 20 replicates of the zero standard was determined and added to their three standard deviations.^64^

### 3.4. Cell cycle analysis by propidium iodide (PI) staining

An apoptosis study was performed at the Cell Culture Unit, VACSERA, Cairo, Egypt. These studies were employed against compounds 3c and 5 to investigate their cytotoxic effect on the most sensitive liver hepatocellular carcinoma (Hep3B) cell line. The treated cells were washed twice with ice-cold phosphate-buffered saline (PBS), centrifuged and then fixed in ice-cold 70% (v/v) ethanol to prevent clustering of cells during fixation. Subsequently, cells were washed with PBS, resuspended with 0.1 mg mL^−1^ RNaseA, stained using 50 μg mL^−1^ propidium iodide (PI) and incubated for 40 min at 37 °C. Then 3 mL of PBS was added, and the cells were centrifuged to remove the supernatant. The cells were analyzed by flow cytometry using a FACSort Calibur (Becton Dickinson Biosciences, CA, USA). Finally, the Cell Quest software was used to determine the cell cycle distribution.^65,66^

### 3.5. Annexin-V-FITC apoptosis assay protocol

An Annexin-V-FITC apoptosis assay was used to determine apoptosis and necrosis. The cells were incubated with Annexin-V-FITC, centrifuged and then resuspended in 500 μL of 1× binding buffer. Then 5 μL of Annexin-V-FITC and 5 μL of propidium iodide (PI) were added, and the cells were then trypsinized and washed once with serum-containing media before incubation with Annexin-V-FITC at room temperature for 5 min in the dark. A FACSort Calibur flow cytometer (Becton Dickinson Biosciences) was used to analyze apoptosis.^56,57^

### 3.6. Caspase-3 enzyme assay

Caspase-3 is one of the crucial mediators of programmed cell death (apoptosis) as it is essential for the formation of apoptotic bodies.^67–70^ Active caspase-3 level was measured in pg ml^−1^ protein using an Invitrogen ELISA kit (catalog KHO1091). The following procedures were performed. First, 100 μL of the standard diluent buffer was added to the zero standard wells. Then, 100 μL of standards and controls or diluted samples were added to the appropriate microtitre wells. Cover wells were covered and incubated for 2 h at room temperature. The solution was decanted from wells. Then 100 μL of active caspase-3 detection antibody solution was pipetted into each well. The plate was covered and incubated for 1 h at room temperature. After that, 100 μL of Anti-Rabbit IgG HP working solution was added to each well. The wells were covered with a plate cover and incubated for 30 min at room temperature. Then, 100 μL of stabilized chromogen was added to each well. The liquid in the wells will begin to turn blue, and the wells were incubated for 30 min at room temperature. A stop solution was added to each well. The solution in the wells should change from blue to yellow. The plate was read within 2 h after adding the stop solution. Curve fitting software was used to generate the standard curve.

### 3.7. Molecular modeling study

All docking studies were performed using Discovery Studio 2016. PDB (code: 3QX3) was chosen as a template for the modeling study of targeted compounds bound to DNA topoisomerase II beta binding sites.^71^ The crystal structure was obtained from the RCSB protein data bank. Generally, the protein structure was prepared and followed by adding hydrogen bonds. The prepared molecules were minimized and docked into the protein structure using the CDOCKER protocol. The following steps were used for the present docking study using the Discovery Studio software:

(1) Loading of Topo2β complex: the 3D protein structure of the target Topo2β enzyme (3QX3) complexed with its ligand with a resolution of 2.3 Å was downloaded from the RCSB protein data bank as a PDB file.

(2) Preparation of the protein: the PDB file was opened in Discovery Studio. Water molecules were retained, and the native ligand was deleted. Protonation step is prepared from protocol/macromolecule/prepare protein. Identify the binding site from tools/define and edit binding site/define receptor then script/select ligand.

(3) Preparation of the test set: the test set molecules were drawn in 2D structures using the ChemBioDraw Ultra 11.0 software and saved as an MDL file. These molecules were opened on Discovery Studio. Energy minimization of targeted compounds was run from tools/full minimization. From tools/apply force field.

(4) Docking of the test set: from protocol/macromolecule/docking using CDOCKER protocol.

(5) Analysis of docking result: after running the protocol, a report about 10 different conformers of each molecule was obtained and then the best conformer was selected according to the – CDOCKER energy. The binding mode and interaction of the tested compounds were visualized at the binding sites.

### 3.8. Physicochemical properties

The SwissADME web tool was used to estimate the physicochemical and pharmacokinetic characteristics *in silico* (Swissadme. Available online: https://www.swissadme.ch/).

## 4. Conclusion

To sum up, a group of structurally related levofloxacin compounds have been synthesized. Their cytotoxic activities against the three cell lines, namely, MCF-7, Hep3B and L-SR were investigated by an MTT method. Compounds 3c, 4b, 5, 7, 8, 13a and 13c possessed the highest cytotoxic activity against the Hep3b cancer cell line. Nevertheless, compounds 3c, 5 and 13a showed the highest cytotoxic activity against the L-SR cancer cell line, while compounds 3c and 7 exhibited the best cytotoxic activity against the MCF-7 cancer cell line. The inhibition of Topo2β polymerase enzyme was also investigated. Compounds 3c and 7 showed the best Topo2β inhibition activity, compared to etoposide and levofloxacin as reference drugs. An Annexin-V-FITC apoptosis assay protocol was used to investigate the cell cycle apoptosis profile. Compound 3c and compound 5 showed cell cycle arrest at the S phase. Compounds 3c and 5 showed elevation in caspase-3 levels. The effects on normal cell lines were evaluated in comparison to etoposide and levofloxacin. Compound 5 manifested lesser side effects on the normal breast cell line MCF-10a and on the normal liver cell line THLE2.

## Data availability

The data supporting this article have been included as part of the ESI.†

## Conflicts of interest

There are no conflicts to declare.

## Supplementary Material

RA-014-D4RA03975K-s001
