# Supplementary material for: Levofloxacin reposition-based design: synthesis, biological evaluation of new levofloxacin derivatives targeting topoisomerase II beta polymerase as promising anticancer agents, molecular docking, and physicochemical characterization
Source: RSC Adv. 2024 Sep 3;14(38):28098–119. doi: 10.1039/d4ra03975k (PMC11369887; doi:10.1039/d4ra03975k)

Supplementary Information (SI) for RSC Advances.
This journal is © The Royal Society of Chemistry 2024

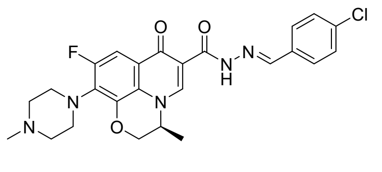

3a IR

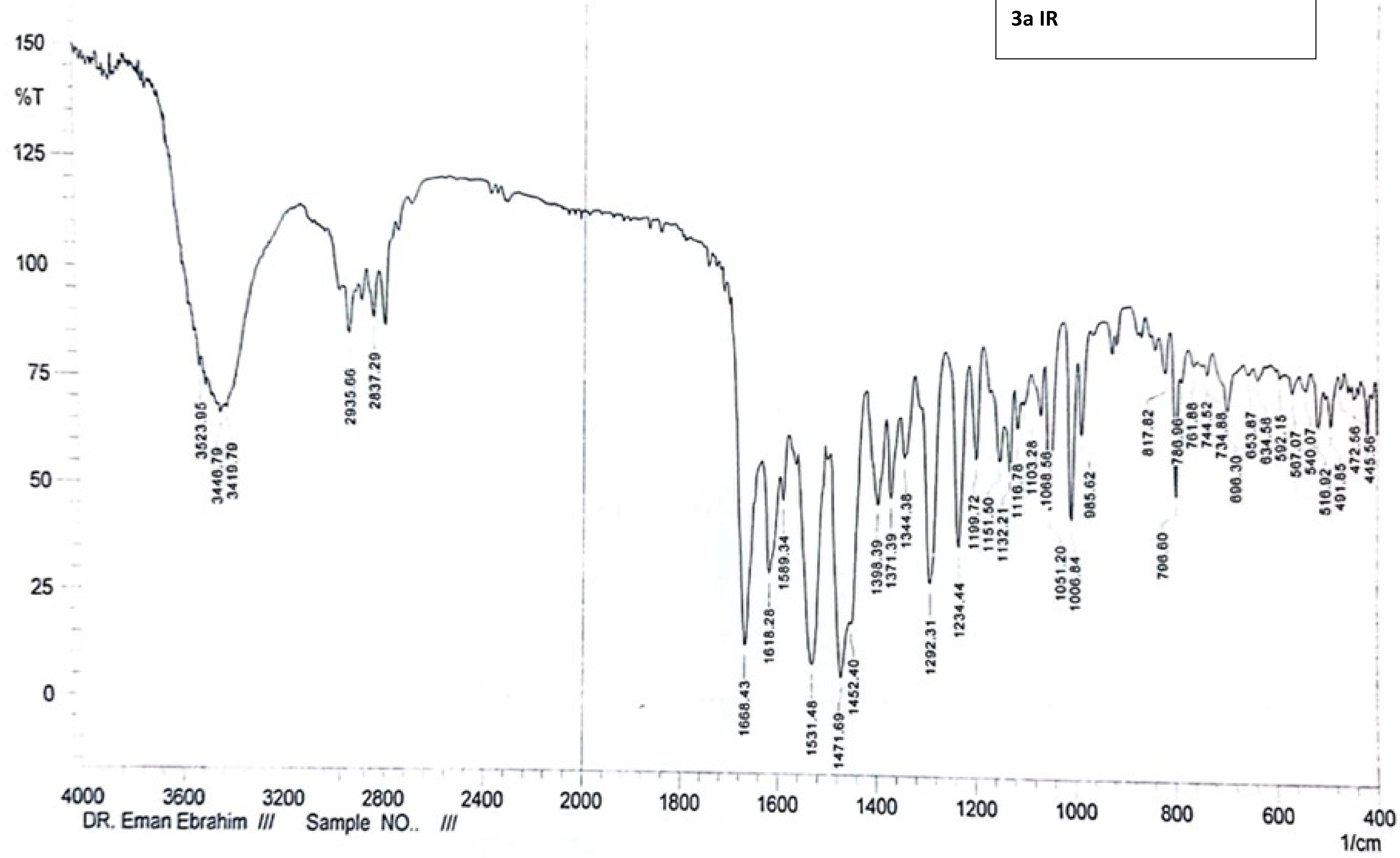

Comment:
DR. Eman Ebrahim /// Sample NO.. ///

**Prepared By : Dr.Ahmed A.EL-Wakeel , Ph.D.

Date/Time; 4/30/2010 3:03:02 AM
No. of Scans; 10
Resolution; 4 [1/cm]
Apodization; Happ-Genzel
User; Heinrich

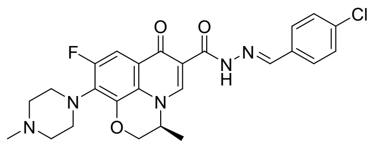

3a NMR

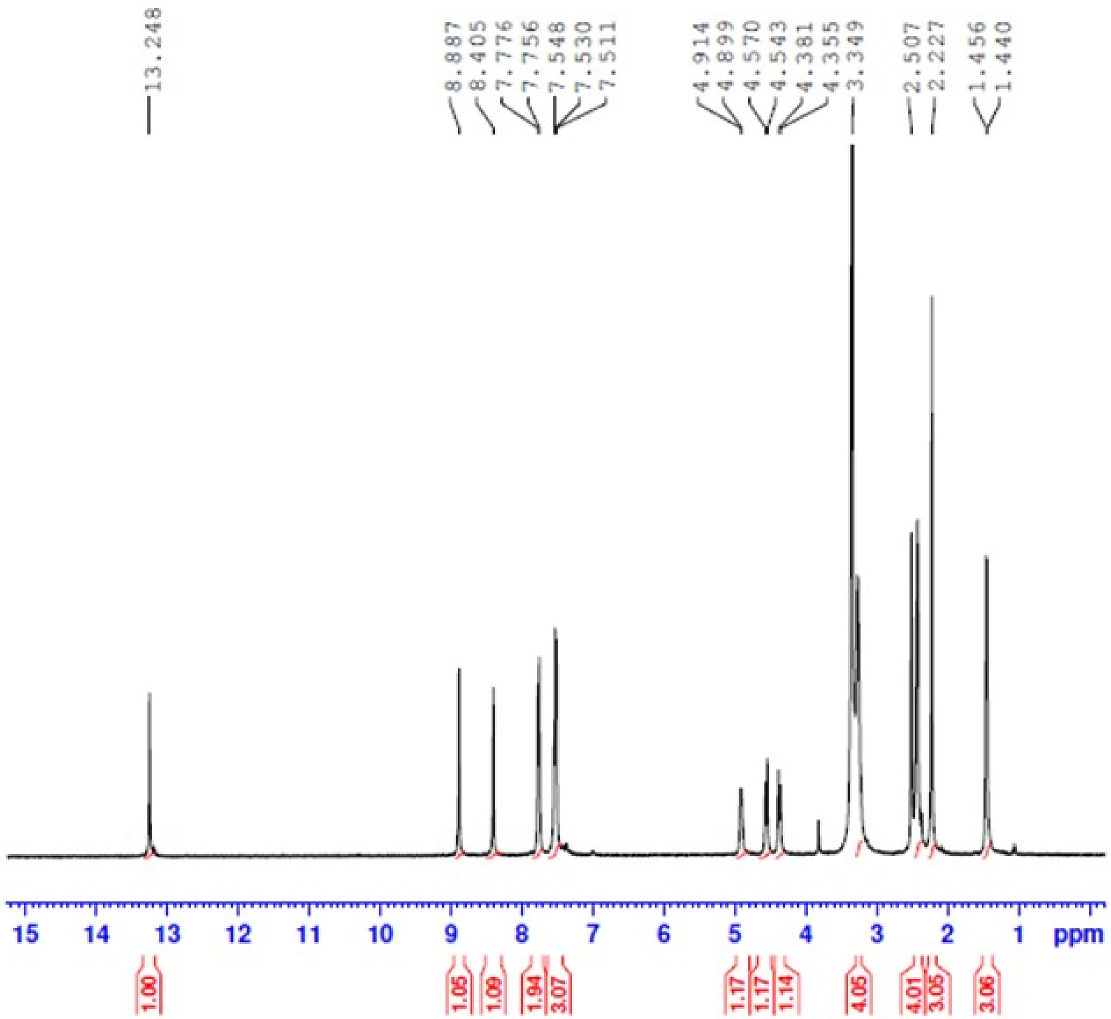

BRUKER

```
Current Data Parameters
NAME          amira-youssef-       4cl
EXPNO                    1
PROCNO                   1

F2 - Acquisition Parameters
Date_             20211212
Time                 16.31
INSTRUM              spect
PROBHD   5 mm PABBO BB/
PULPROG               zg30
TD                   65536
SOLVENT               DMSO
NS                      20
DS                       2
SWH               8012.820 Hz
FIDRES            0.122266 Hz
AQ               4.0894465 sec
RG                  205.37
DW                  62.400 usec
DE                    6.50 usec
TE                   300.0 K
D1              1.00000000 sec
TD0                      1

======= CHANNEL f1 =======
SFO1           400.1524711 MHz
NUC1                    1H
P1                   12.00 usec
PLW1           18.00000000 W

F2 - Processing parameters
SI                   65536
SF             400.1500000 MHz
WDW                     EM
SSB      0
LB                    0.30 Hz
GB       0
PC                    1.00
```

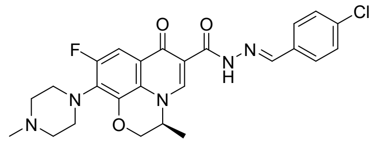

3a D_2O

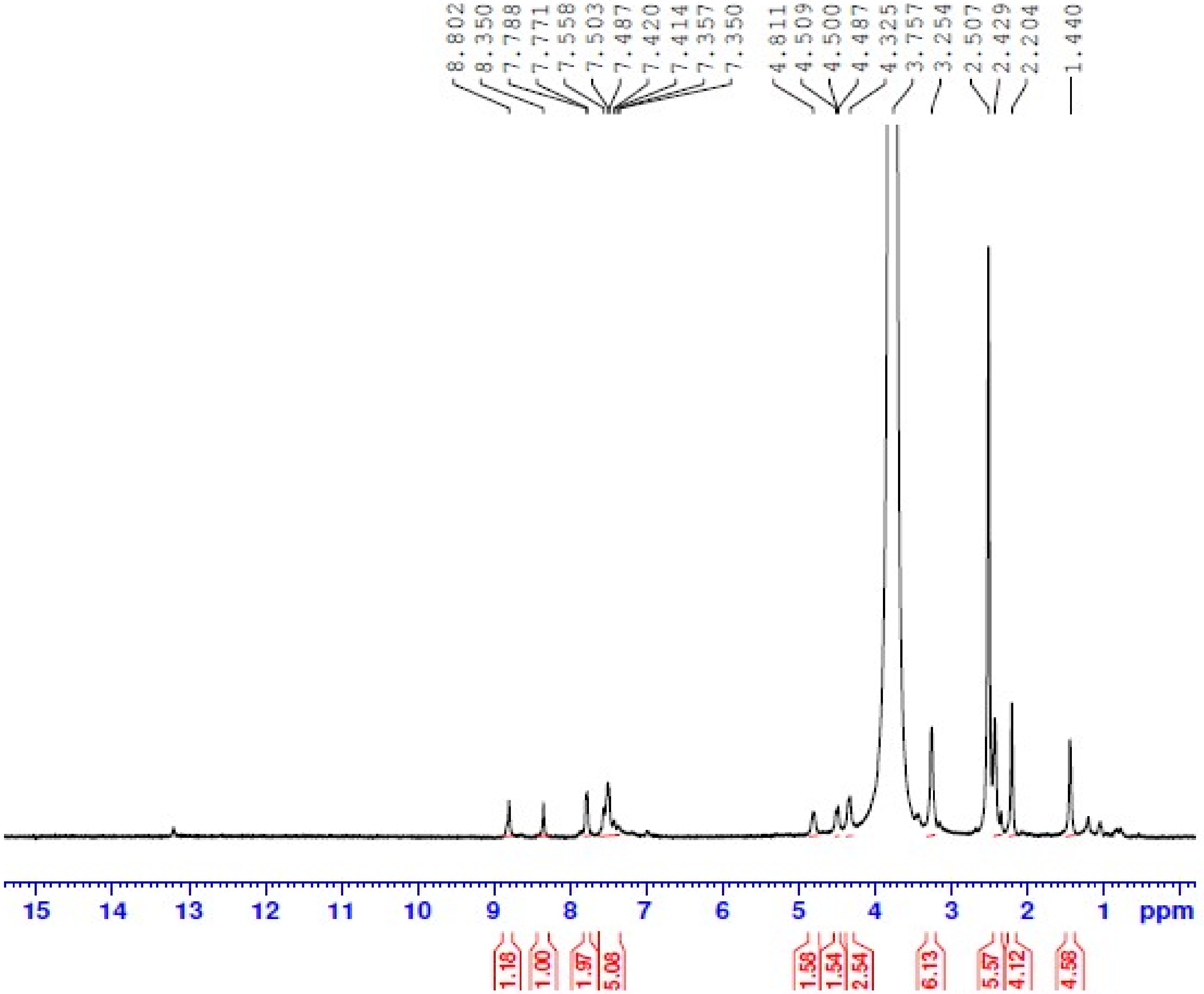

BRUKER

```
Current Data Parameters
NAME      amira-youssef-chalc-4cl-d2o
EXPNO                  1
PROCNO                 1

F2 - Acquisition Parameters
Date_           20211226
Time               12.07
INSTRUM            spect
PROBHD   5 mm PABBO BB/
PULPROG             zg30
TD                 65536
SOLVENT             DMSO
NS                   128
DS                     2
SWH             8012.820 Hz
FIDRES          0.122266 Hz
AQ             4.0894465 sec
RG                164.32
DW                62.400 usec
DE                  6.50 usec
TE                 300.0 K
D1            1.00000000 sec
TD0                    1

======== CHANNEL f1 ========
SFO1         400.1524711 MHz
NUC1                  1H
P1                 12.00 usec
PLW1         18.00000000 W

F2 - Processing parameters
SI                 65536
SF           400.1500000 MHz
WDW                   EM
SSB      0
LB                  0.30 Hz
GB       0
PC                  1.00
```

Amira Youssef_C_4-Cl

Microanalytical Unit - FOPCU - NMR laboratory
www.pharma.cu.edu.eg dir-mau.fopcu@pharma.cu.edu.eg

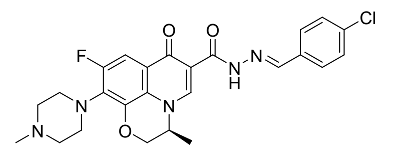

3a ^{13}C

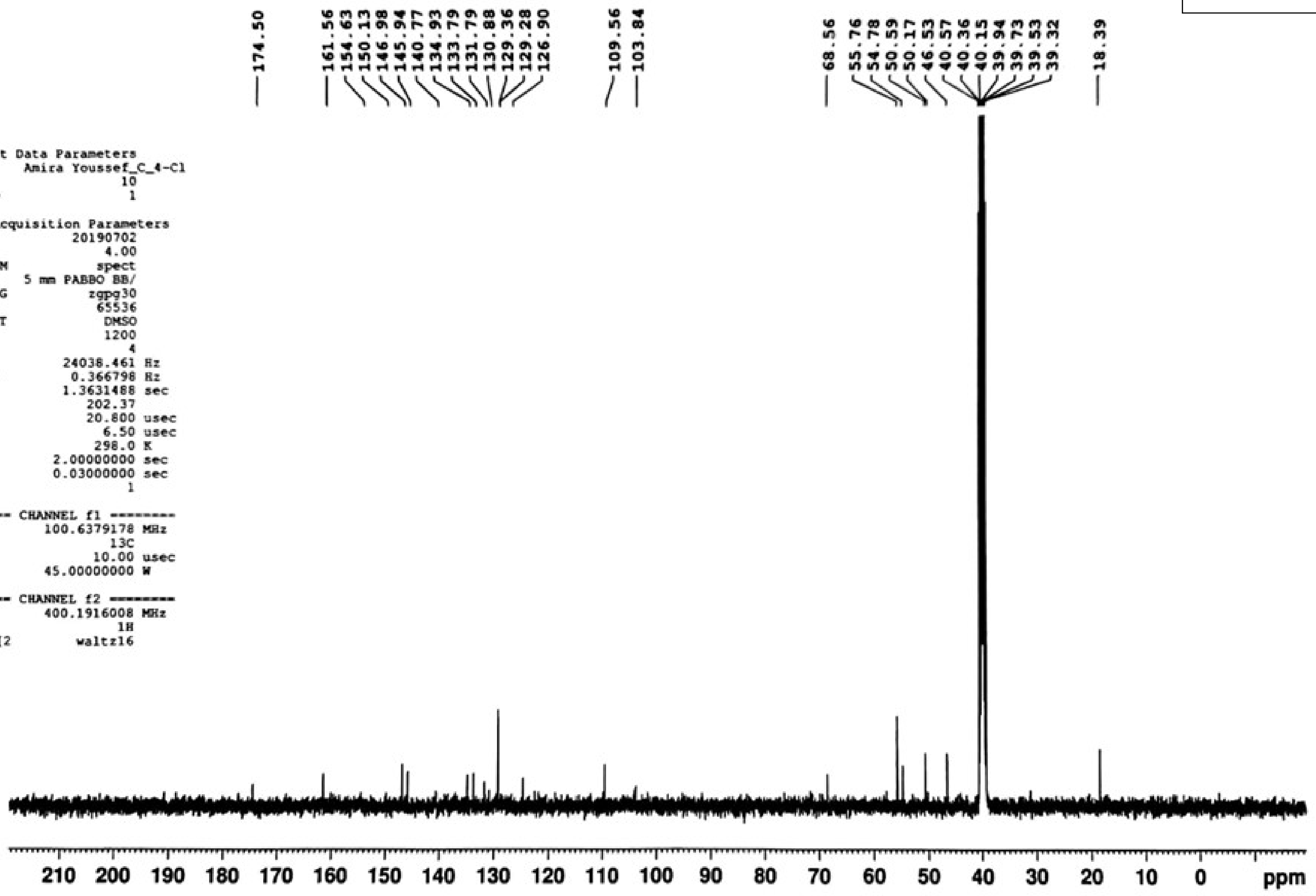


```
Current Data Parameters
NAME       Amira Youssef_C_4-Cl
EXPNO                     10
PROCNO                     1

F2 - Acquisition Parameters
Date_              20190702
Time                   4.00
INSTRUM               spect
PROBHD   5 mm PABBO BB/
PULPROG             zgpg30
TD                   65536
SOLVENT               DMSO
NS                    1200
DS                       4
SWH              24038.461 Hz
FIDRES            0.366798 Hz
AQ               1.3631488 sec
RG                  202.37
DW                  20.800 usec
DE                    6.50 usec
TE                   298.0 K
D1              2.00000000 sec
D11             0.03000000 sec
TD0                      1

======== CHANNEL f1 ========
SFO1          100.6379178 MHz
NUC1                  13C
P1                  10.00 usec
PLW1          45.00000000 W

======== CHANNEL f2 ========
SFO2          400.1916008 MHz
NUC2                   1H
CPDPRG[2           waltz16
```

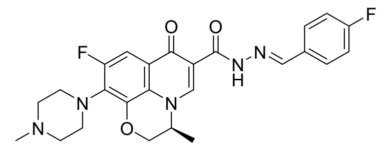

3b IR

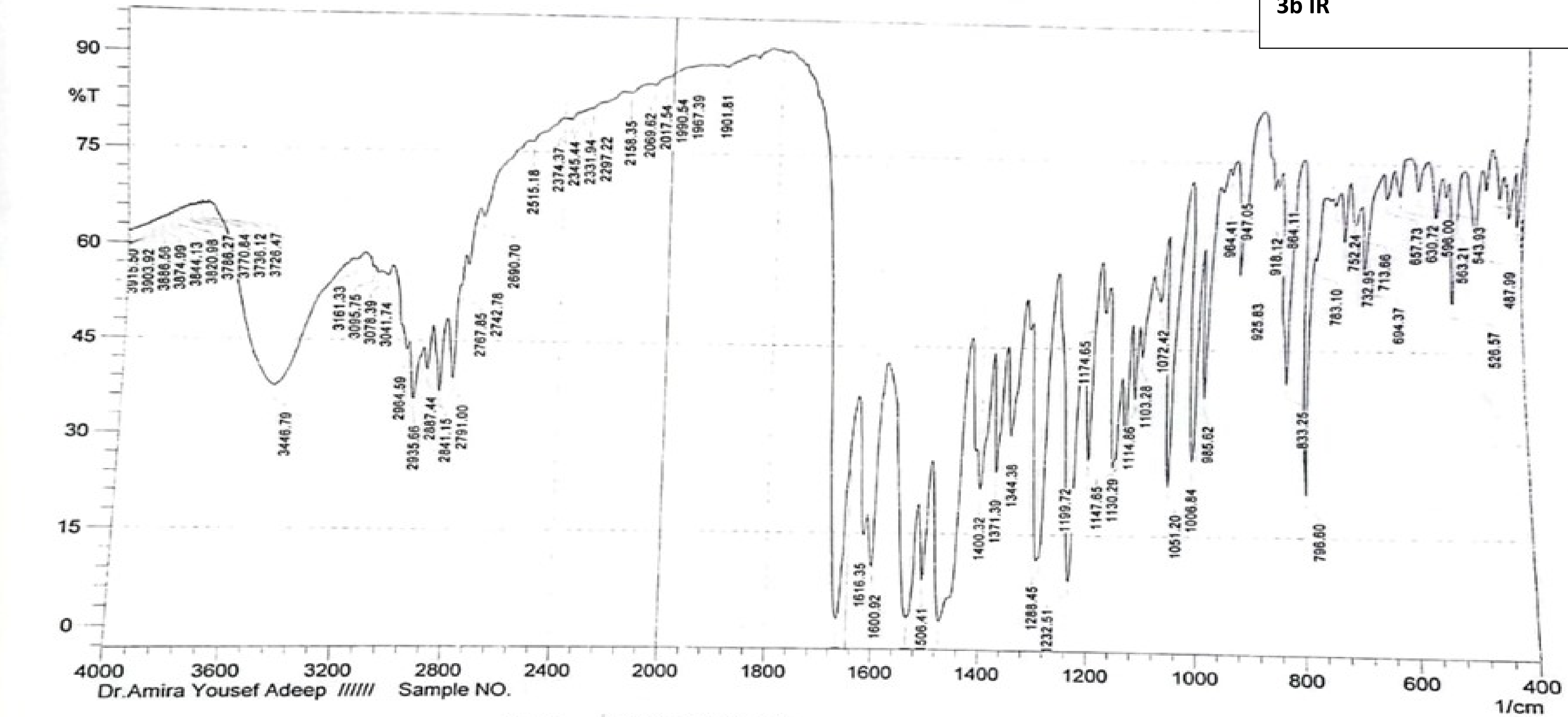

Comment;
Dr.Amira Yousef Adeep ////// Sample NO. (P-F

**Prepared By : Dr.Ahmed A.EL-Wakeel , Ph.D.

Date/Time; 4/29/2010 11:18:05 PM
No. of Scans; 10
Resolution; 4 [1/cm]
Apodization; Happ-Genzel
User: Heinrich

Amira Yousef_H_P-F

Microanalytical Unit - FOPCU - NMR laboratory
www.pharma.cu.edu.eg dir-mau.fopcu@pharma.cu.edu.eg

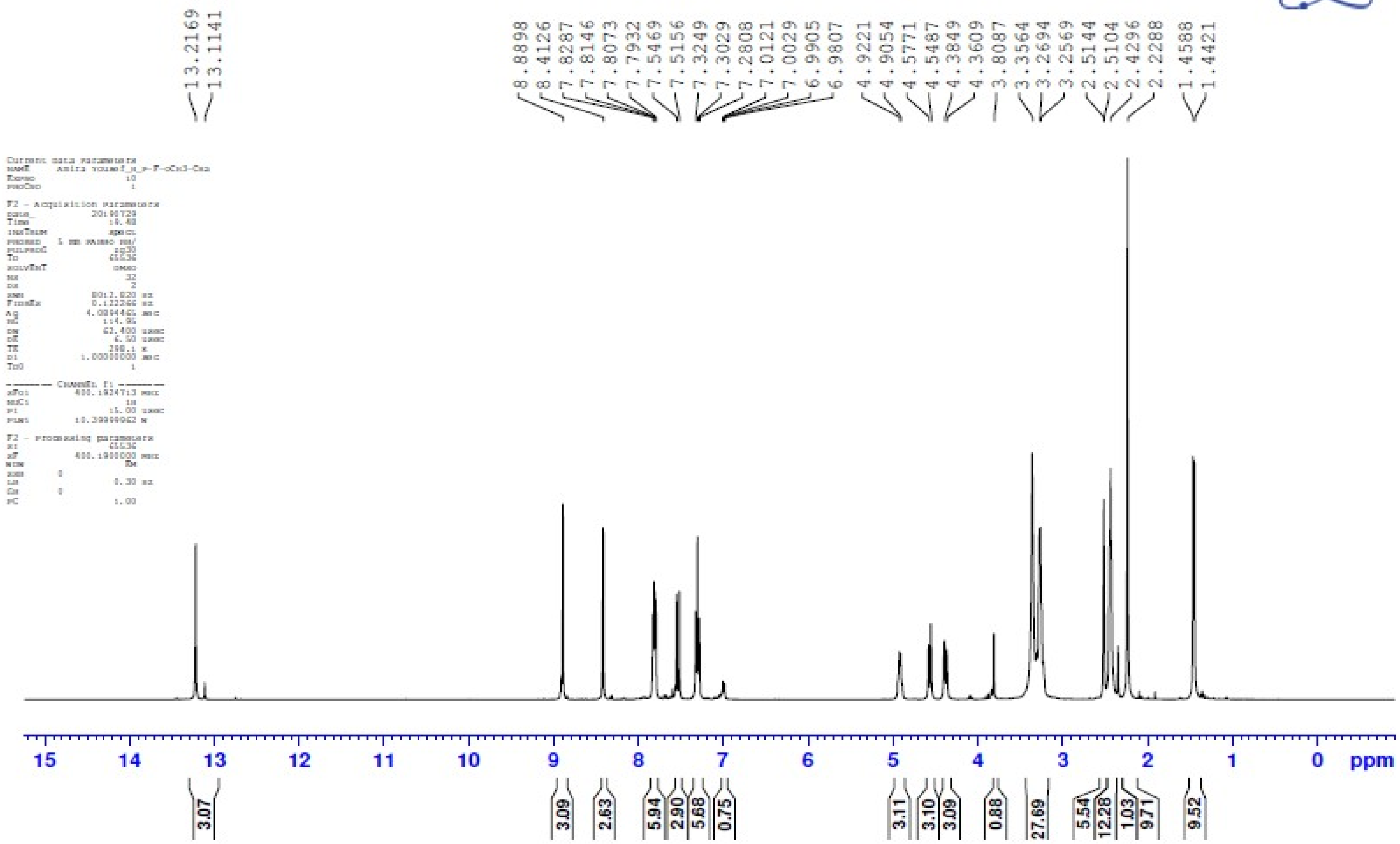

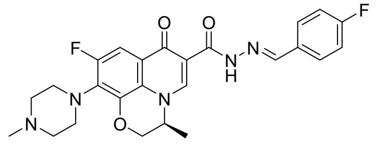

3b NMR

Amira Yousef_C_P-F-

Microanalytical Unit - FOPCU - NMR laboratory
www.pharma.cu.edu.eg dir-mau.fopcu@pharma.cu.edu.eg

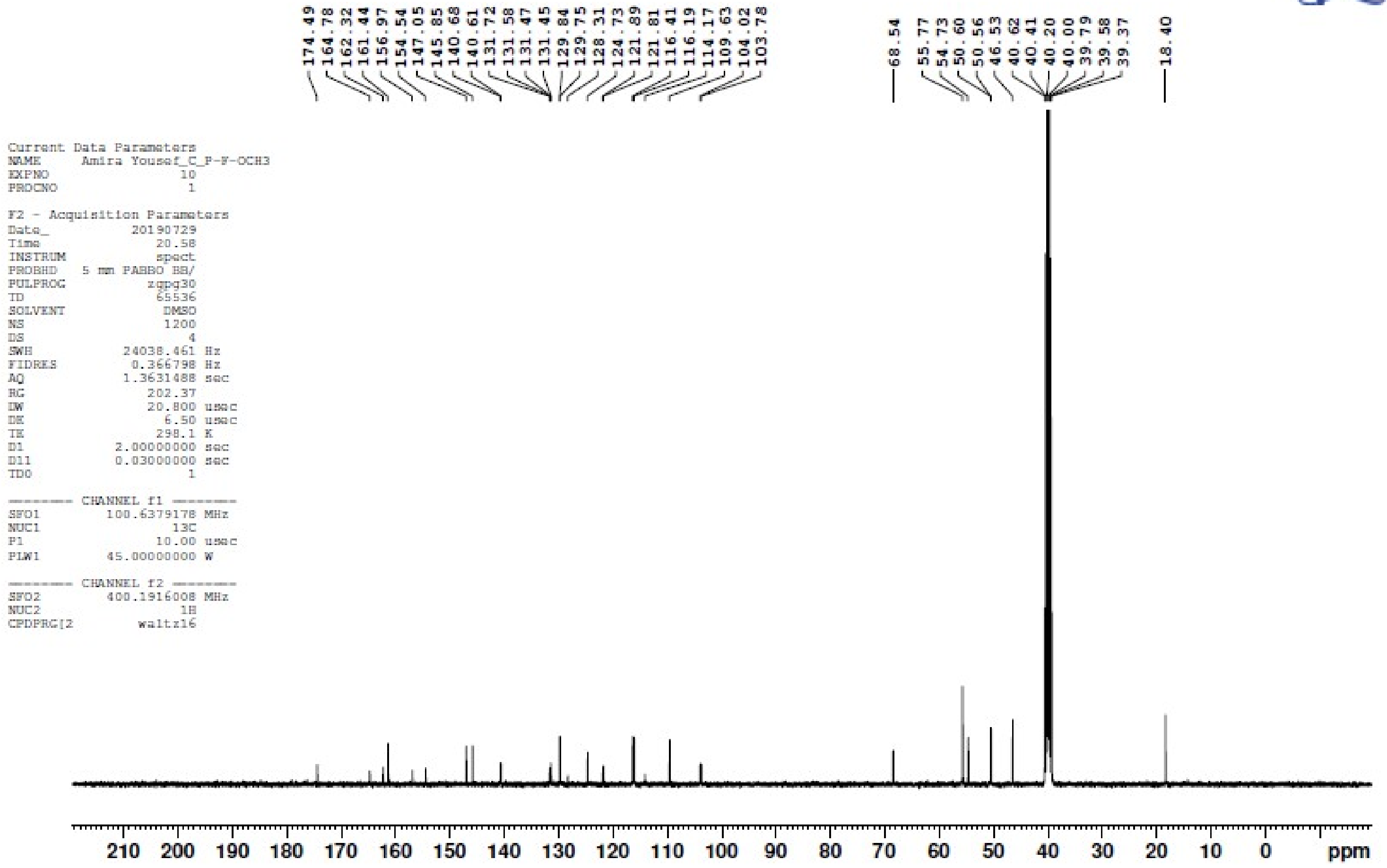

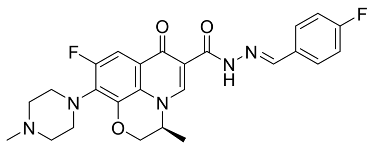

3b ^{13}C

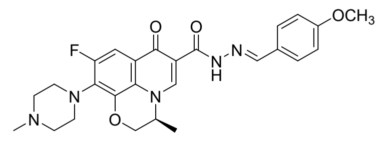

3c IR

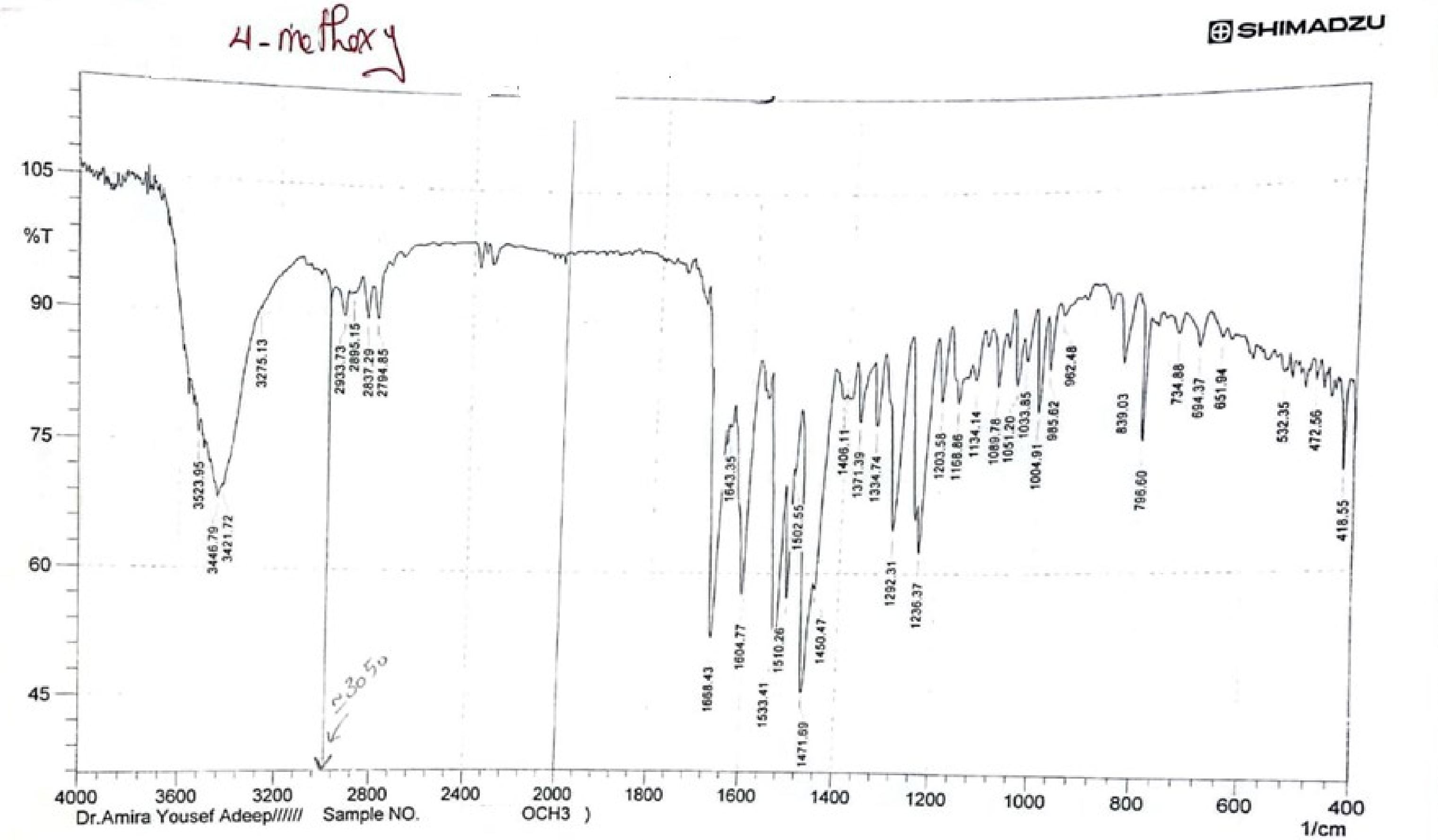

Comment;
Dr.Amira Yousef Adeep////// Sample NO.
OCH3)
**Prepared By : Dr.Ahmed A.EL-Wakeel , Ph.D.

Date/Time; 1/21/2019 12:05:34 PM
No. of Scans; 10
Resolution; 4 [1/cm]
Apodization; Happ-Genzel
User; Heinrich

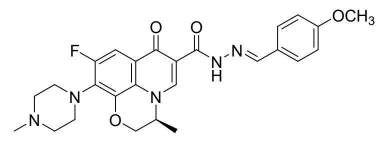
OCH3
F
O
O
N
H
N
N
N
O
N

3c NMR

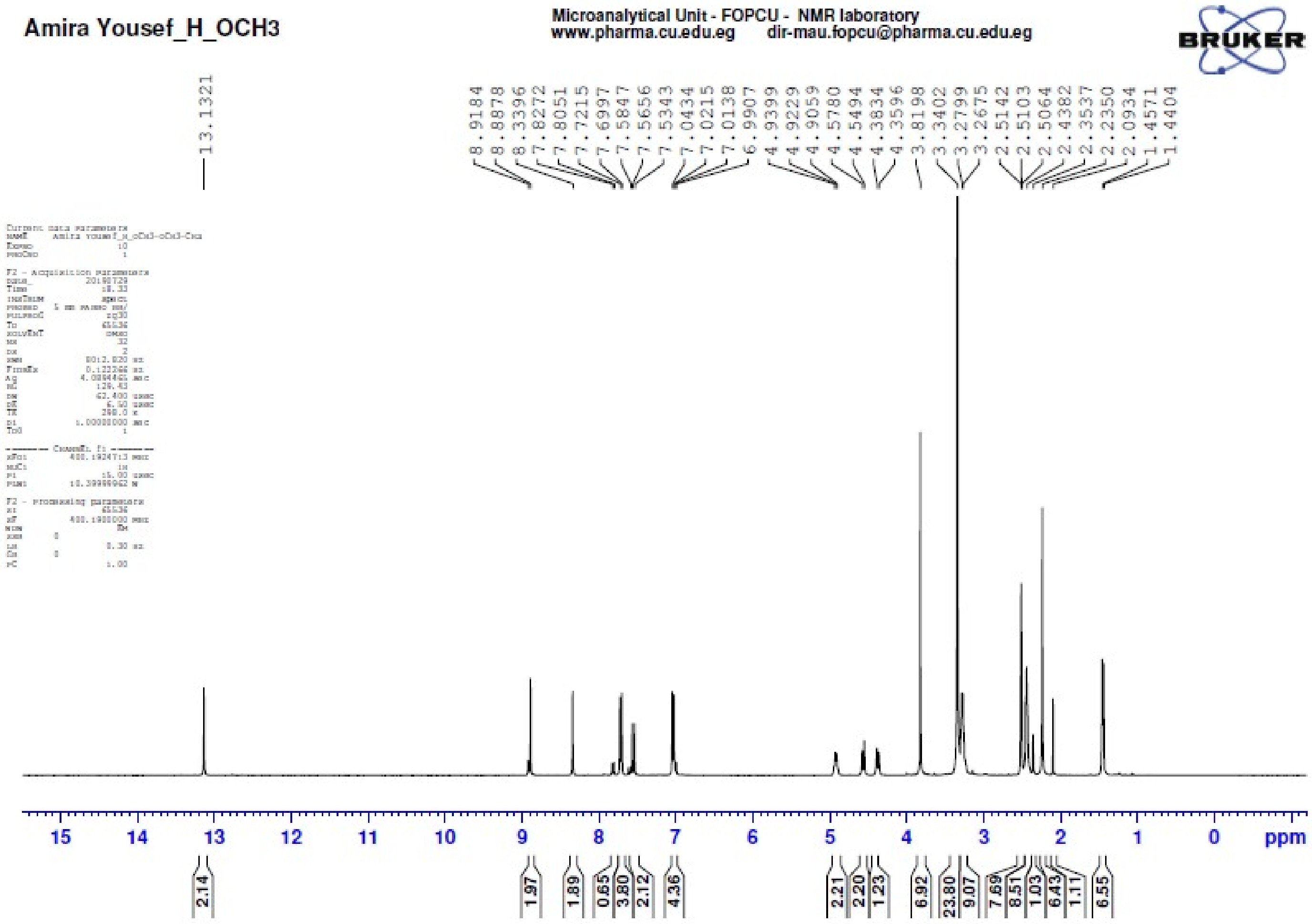
Amira Yousef_H_OCH3
Microanalytical Unit - FOPCU - NMR laboratory
www.pharma.cu.edu.eg dir-mau.fopcu@pharma.cu.edu.eg
BRUKER
13.1321
8.9184
8.8878
8.3396
7.8272
7.8051
7.7215
7.6997
7.5847
7.5656
7.5343
7.0434
7.0215
7.0138
6.9907
4.9399
4.9229
4.9059
4.5780
4.5494
4.3834
4.3596
3.8198
3.3402
3.2799
3.2675
2.5142
2.5103
2.5064
2.4382
2.3537
2.2350
2.0934
1.4571
1.4404
15 14 13 12 11 10 9 8 7 6 5 4 3 2 1 0 ppm
2.14
1.97
1.89
0.65
3.80
2.12
4.36
2.21
2.20
1.23
6.92
23.80
9.07
7.69
8.51
1.03
6.43
1.11
6.55

Amira Yousef_C_OCH3-

Microanalytical Unit - FOPCU - NMR laboratory
www.pharma.cu.edu.eg dir-mau.fopcu@pharma.cu.edu.eg

BRUKER

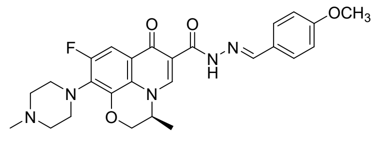

3c ^{13}C

```
Current Data Parameters
NAME      Amira Yousef_C_OCH3-OCH
EXPNO                10
PROCNO                1

F2 - Acquisition Parameters
Date_          20190729
Time              19.43
INSTRUM           spect
PROBHD   5 mm PABBO BB/
PULPROG         zgpg30
TD                65536
SOLVENT            DMSO
NS                 1200
DS                    4
SWH           24038.461 Hz
FIDRES         0.366798 Hz
AQ            1.3631488 sec
RG               202.37
DW               20.800 usec
DE                 6.50 usec
TE                298.0 K
D1           2.00000000 sec
D11          0.03000000 sec
TD0                   1

======== CHANNEL f1 ========
SFO1        100.6379178 MHz
NUC1                13C
P1                10.00 usec
PLW1        45.00000000 W

======== CHANNEL f2 ========
SFO2        400.1916008 MHz
NUC2                 1H
CPDPRG[2          waltz16
```

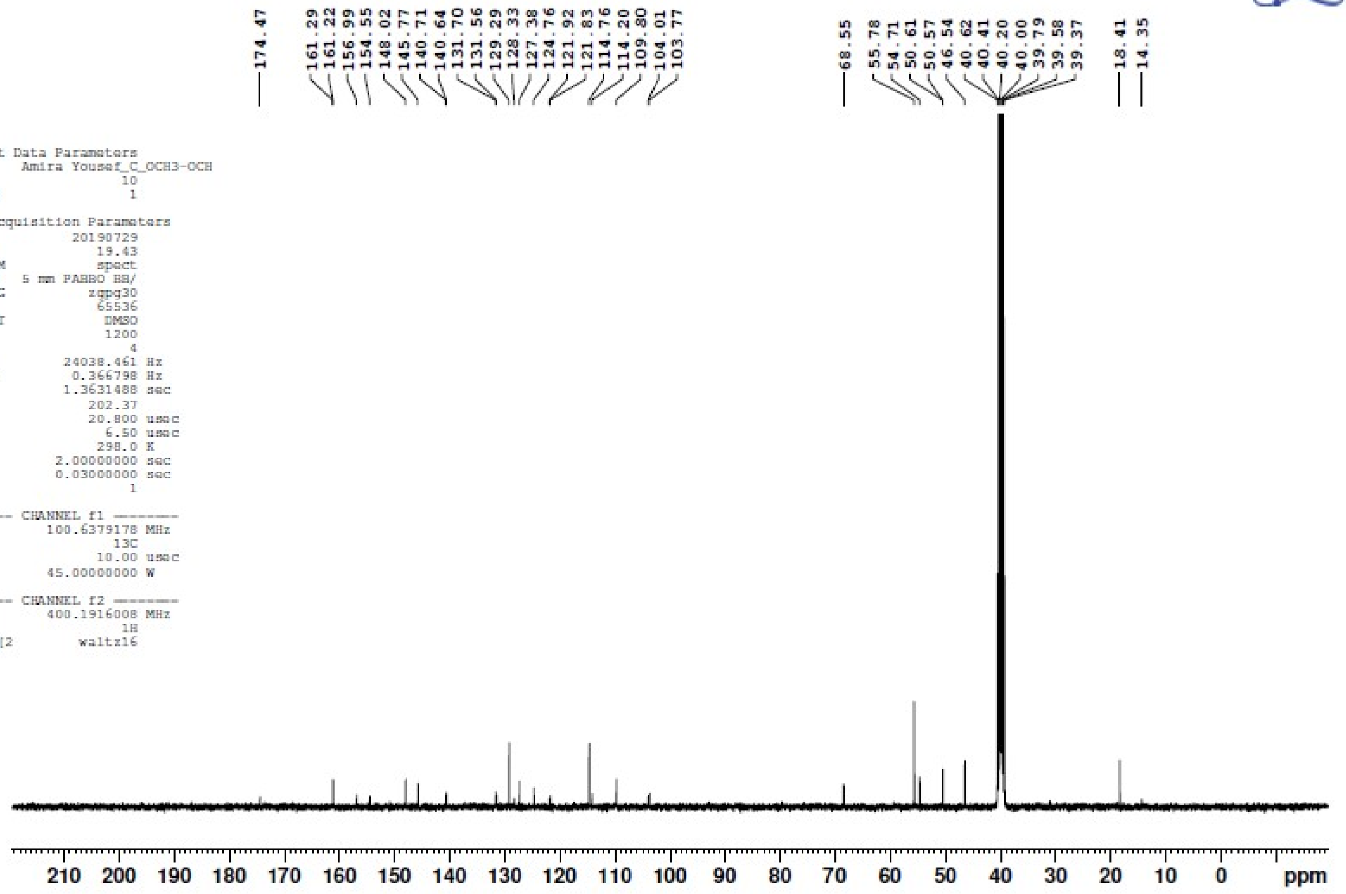

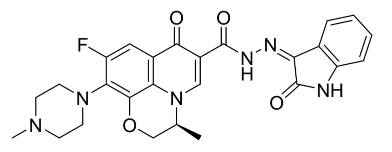

4a IR

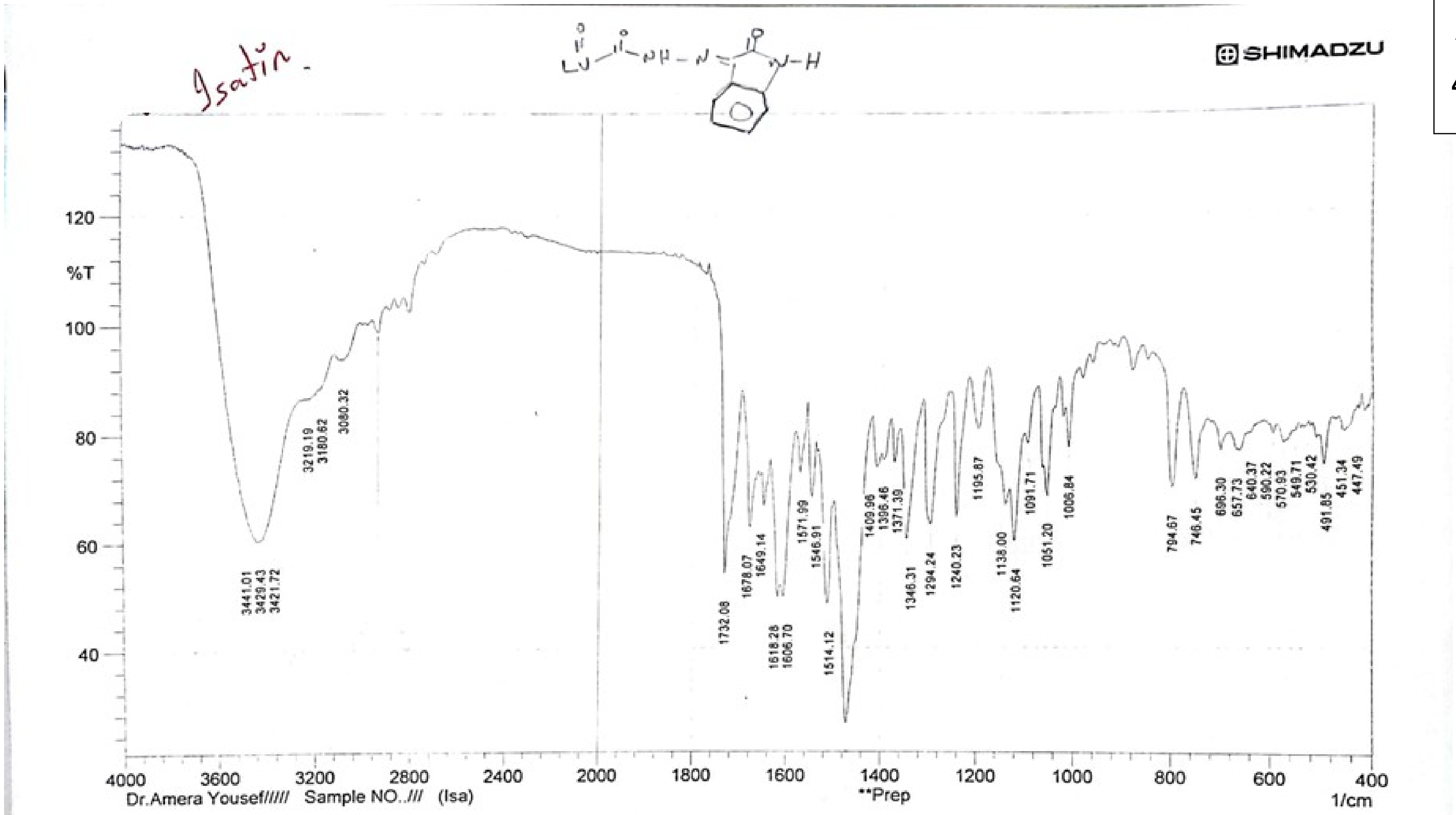

Comment;
Dr.Amera Yousef///// Sample NO../// (Isa)
**Prepared By : Dr.Ahmed A.EL-Wakeel , Ph.D.

Date/Time; 4/29/2010 11:32:51 PM
No. of Scans; 10
Resolution; 4 [1/cm]
Apodization; Happ-Genzel
User; Heinrich

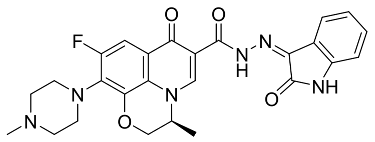

4a NMR

Amira Youssef_H_Isatin

Microanalytical Unit - FOPCU - NMR laboratory
www.pharma.cu.edu.eg dir-mau.fopcu@pharma.cu.edu.eg

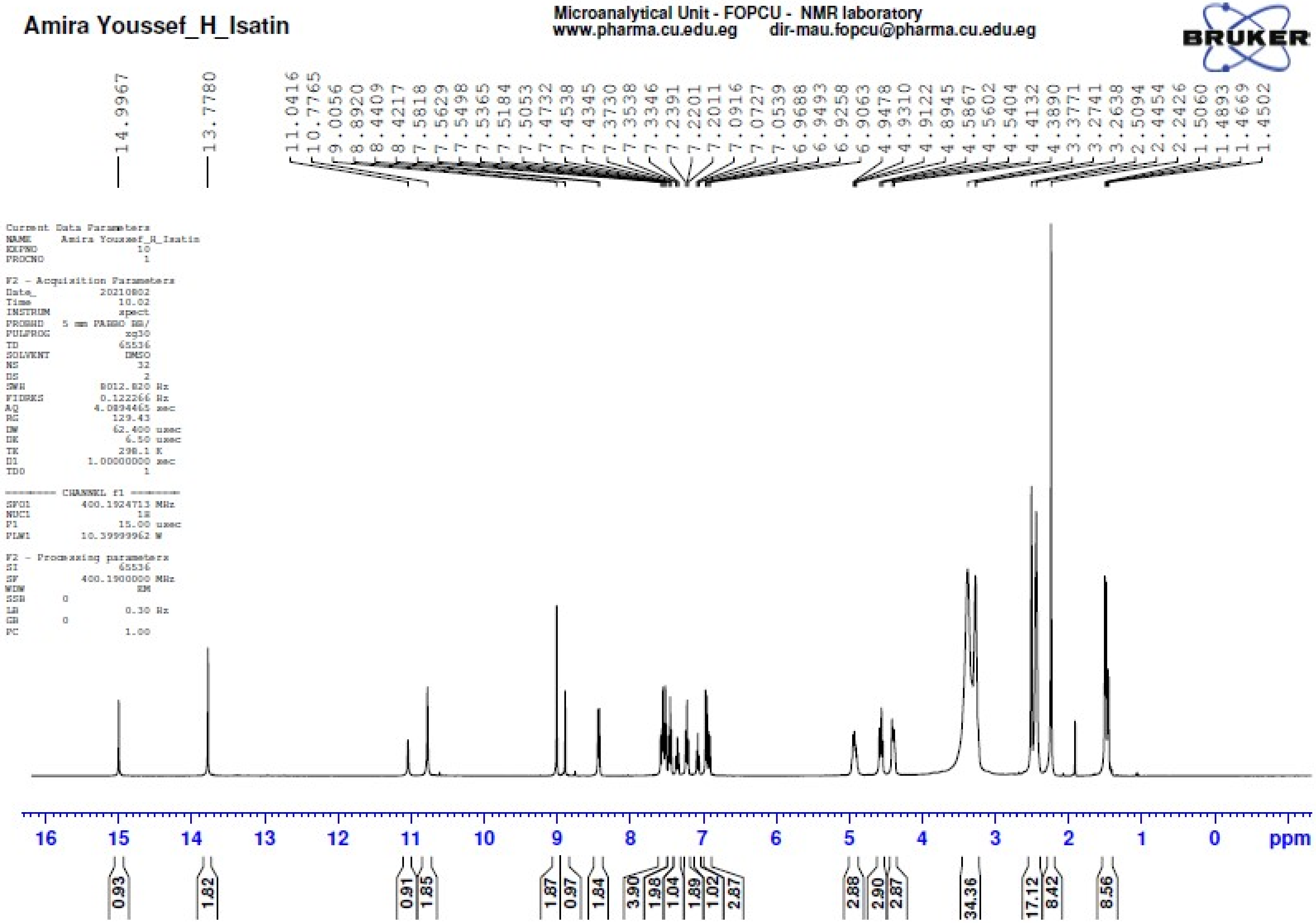

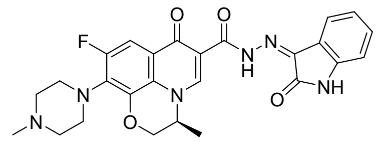

4a ^{13}C

Amira Youssef_C_Isatin

Microanalytical Unit - FOPCU - NMR laboratory
www.pharma.cu.edu.eg dir-mau.fopcu@pharma.cu.edu.eg

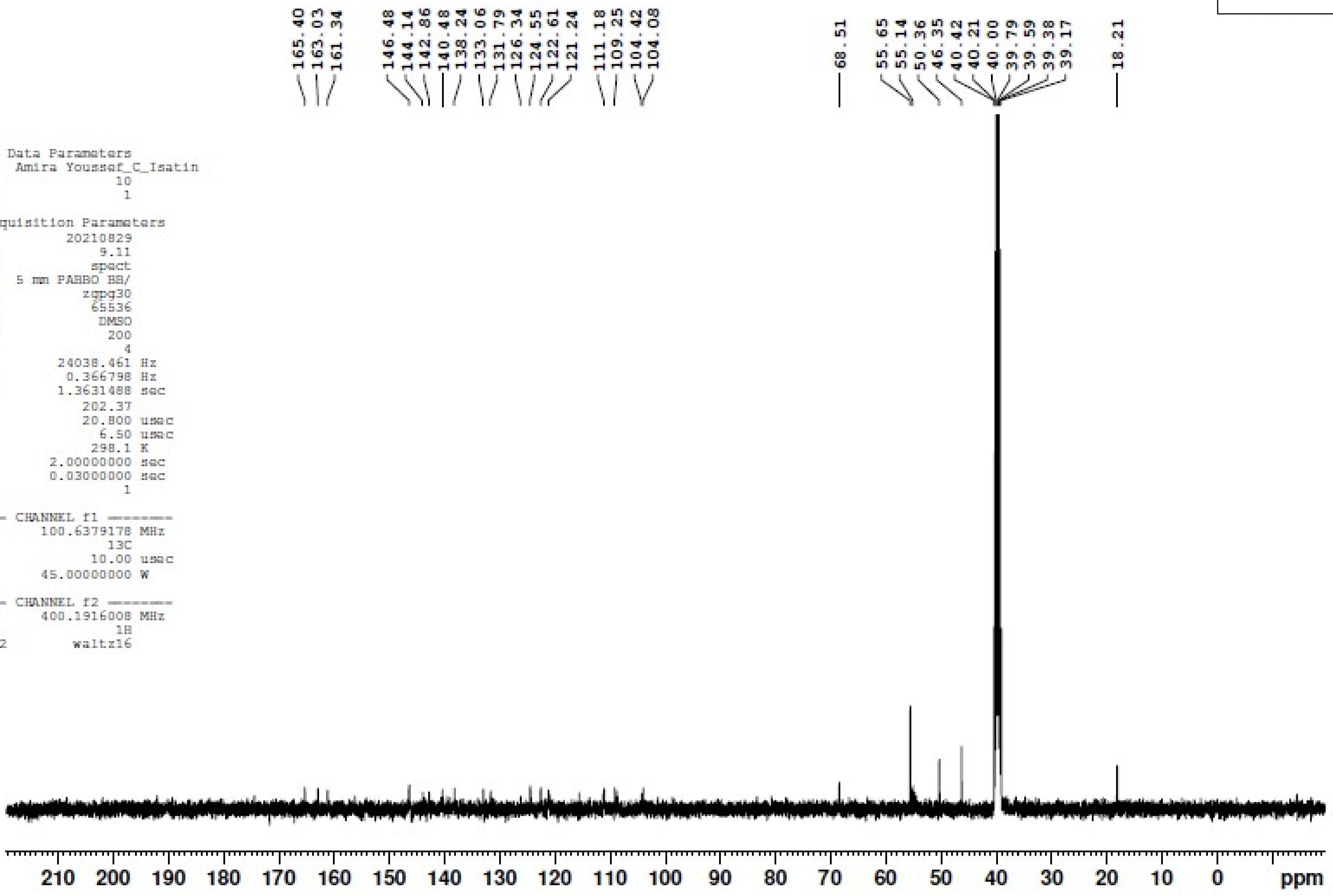

Current Data Parameters
NAME Amira Youssef_C_Isatin
EXPNO 10
PROCNO 1

F2 - Acquisition Parameters
Date_ 20210829
Time 9.11
INSTRUM spect
PROBHD 5 mm PABBO BB/
PULPROG zgpg30
TD 65536
SOLVENT DMSO
NS 200
DS 4
SWH 24038.461 Hz
FIDRES 0.366798 Hz
AQ 1.3631488 sec
RG 202.37
DW 20.800 usec
DE 6.50 usec
TE 298.1 K
D1 2.00000000 sec
D11 0.03000000 sec
TD0 1

======== CHANNEL f1 ========
SFO1 100.6379178 MHz
NUC1 13C
P1 10.00 usec
PLW1 45.00000000 W

======== CHANNEL f2 ========
SFO2 400.1916008 MHz
NUC2 1H
CPDPRG[2 waltz16

Amira Youssef_H_Isatin_D2O

Microanalytical Unit - FOPCU - NMR laboratory
www.pharma.cu.edu.eg dir-mau.fopcu@pharma.cu.edu.eg

BRUKER

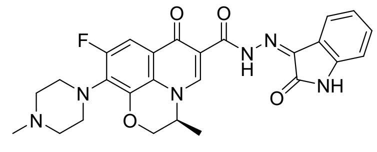

4a D_2O

```
Current Data Parameters
NAME      Amira Youssef_H_Isatin_D2O
EXPNO                    10
PROCNO                    1

F2 - Acquisition Parameters
Date_              20210915
Time                  11.58
INSTRUM               spect
PROBHD   5 mm PABBO BB/
PULPROG                zg30
TD                    65536
SOLVENT                DMSO
NS                       32
DS                        2
SWH             8012.820 Hz
FIDRES          0.122266 Hz
AQ             4.0894465 sec
RG                    32.12
DW               62.400 usec
DE                 6.50 usec
TE                298.0 K
D1           1.00000000 sec
TD0                       1

======== CHANNEL f1 ========
SFO1         400.1924713 MHz
NUC1                     1H
P1                15.00 usec
PLW1        10.39999962 W

F2 - Processing parameters
SI                    65536
SF          400.1900000 MHz
WDW                      EM
SSB       0
LB                  0.30 Hz
GB        0
PC                     1.00
```

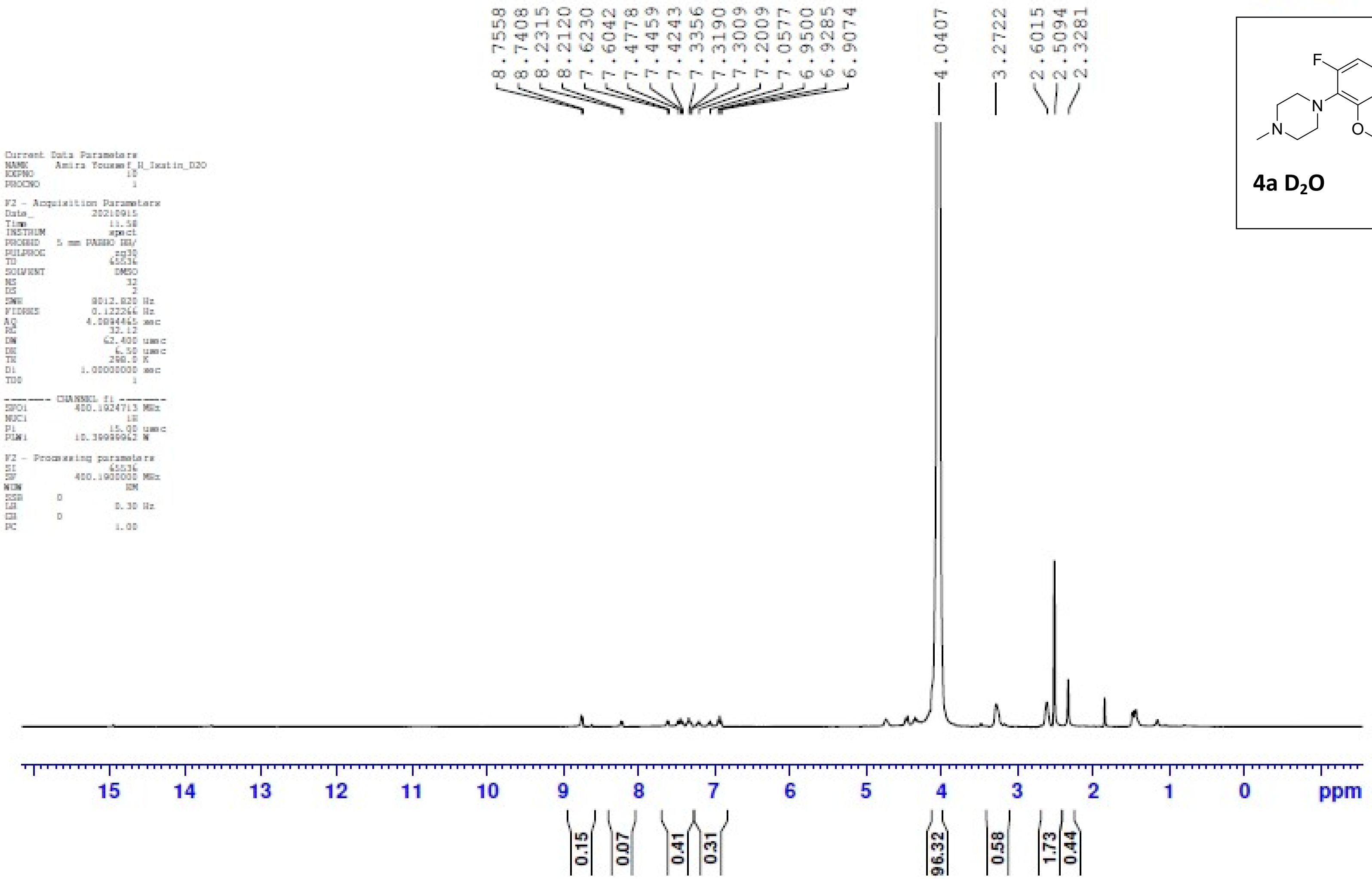

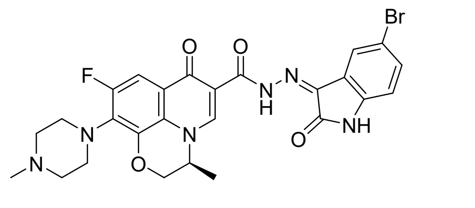

4b IR

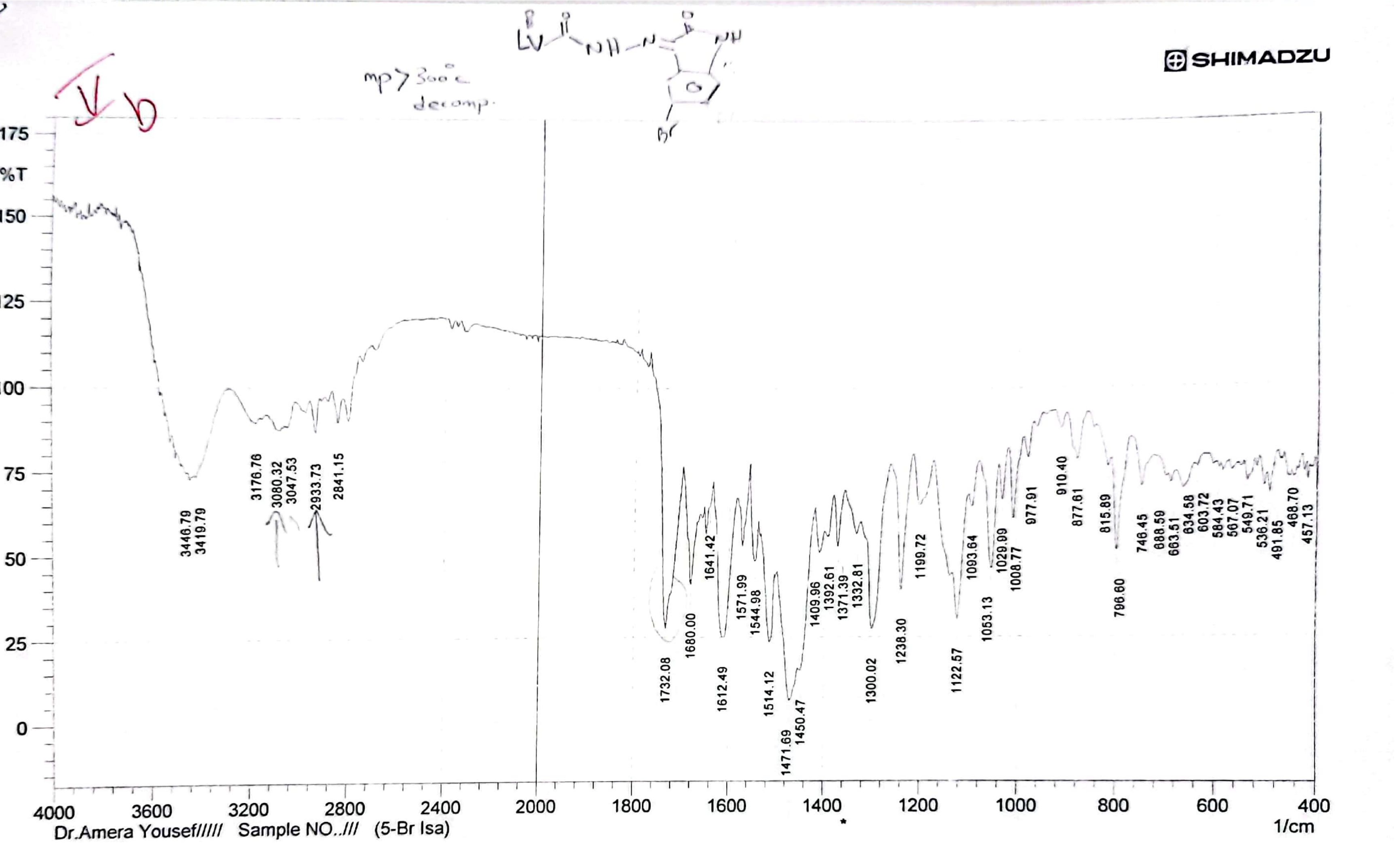

mment;
.Amera Yousef///// Sample NO../// (5-Br Isa)
Prepared By : Dr.Ahmed A.EL-Wakeel , Ph.D.

Date/Time; 4/30/2010 2:39:10 AM
No. of Scans; 10
Resolution; 4 [1/cm]

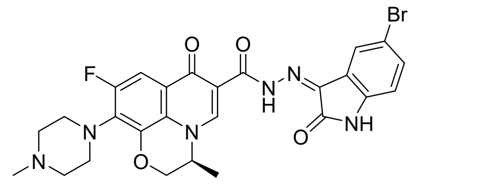

4b NMR

Amira Youssef_H_5-Br-Isa

Microanalytical Unit - FOPCU - NMR laboratory
www.pharma.cu.edu.eg dir-mau.fopcu@pharma.cu.edu.eg

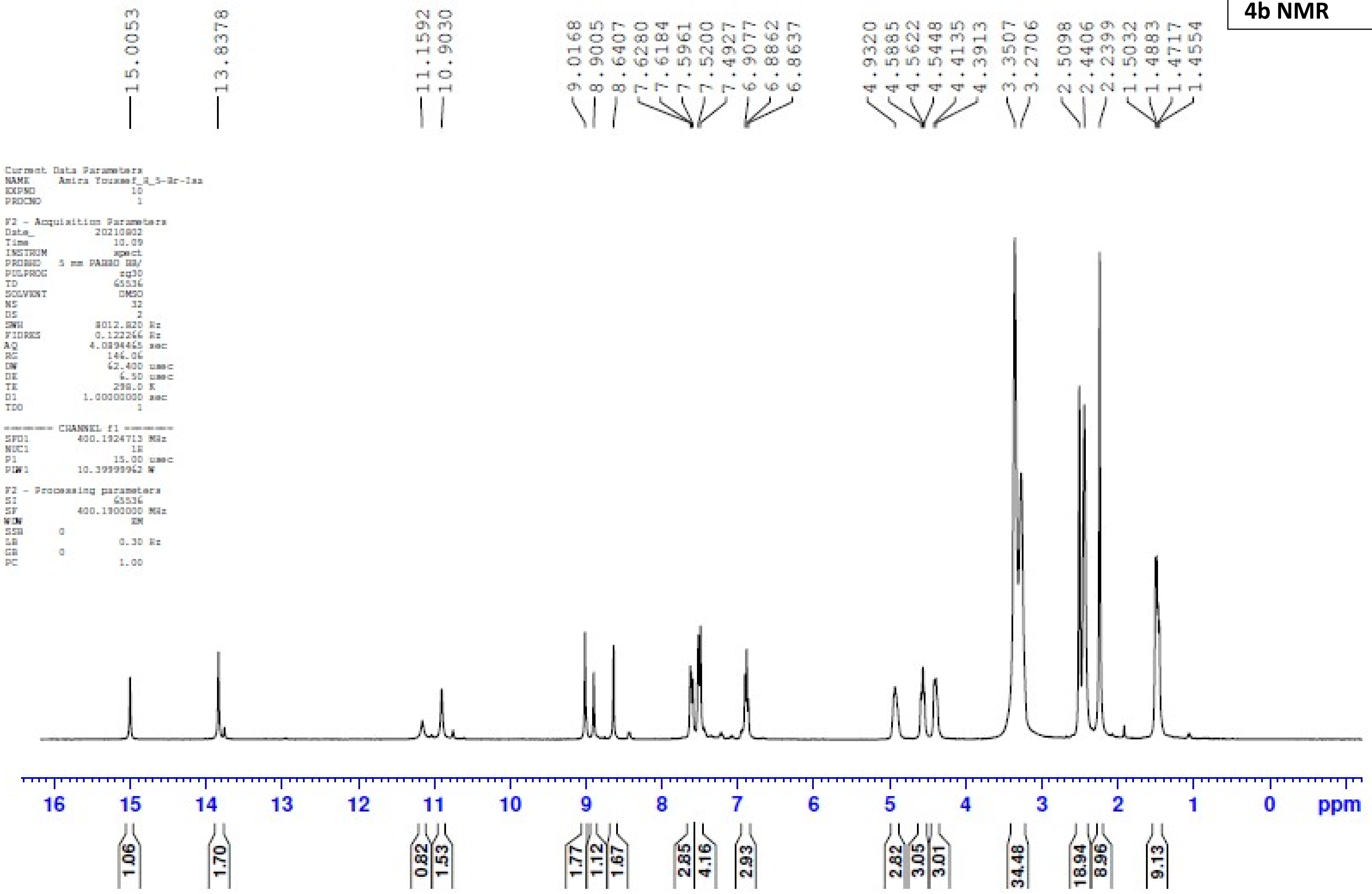

Amira Youssef_H_5-Br-Isa_D2O

Microanalytical Unit - FOPCU - NMR laboratory
www.pharma.cu.edu.eg dir-mau.fopcu@pharma.cu.edu.eg

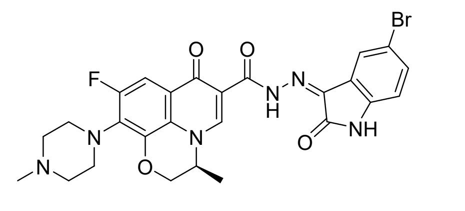

4b D_2O

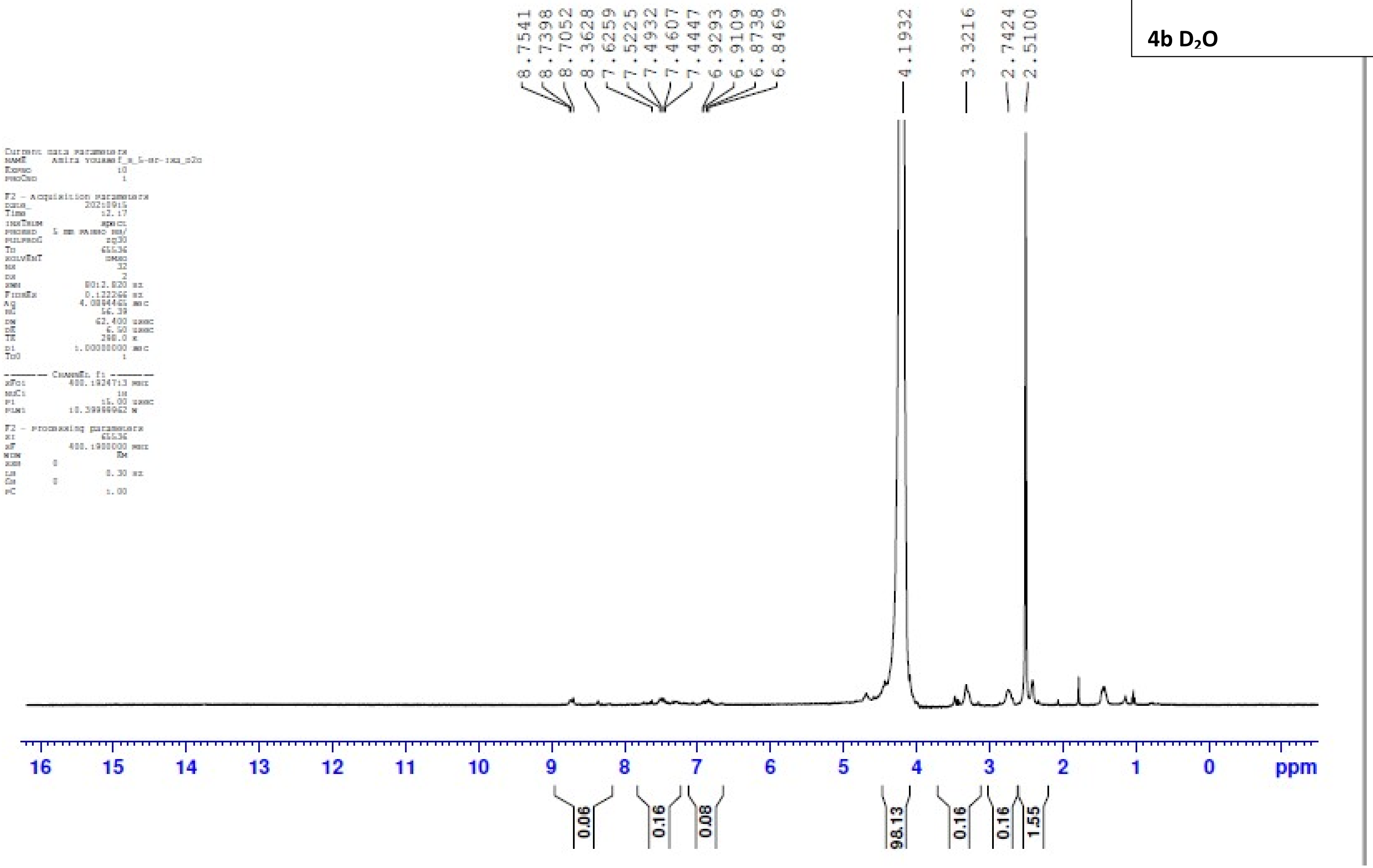

Amira Youssef_C_5-Br-Isa

Microanalytical Unit - FOPCU - NMR laboratory
www.pharma.cu.edu.eg dir-mau.fopcu@pharma.cu.edu.eg

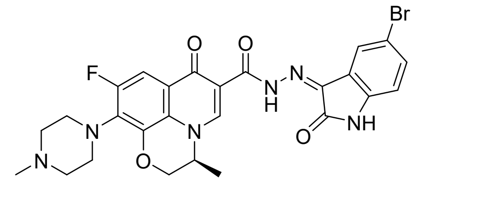

4b ^{13}C

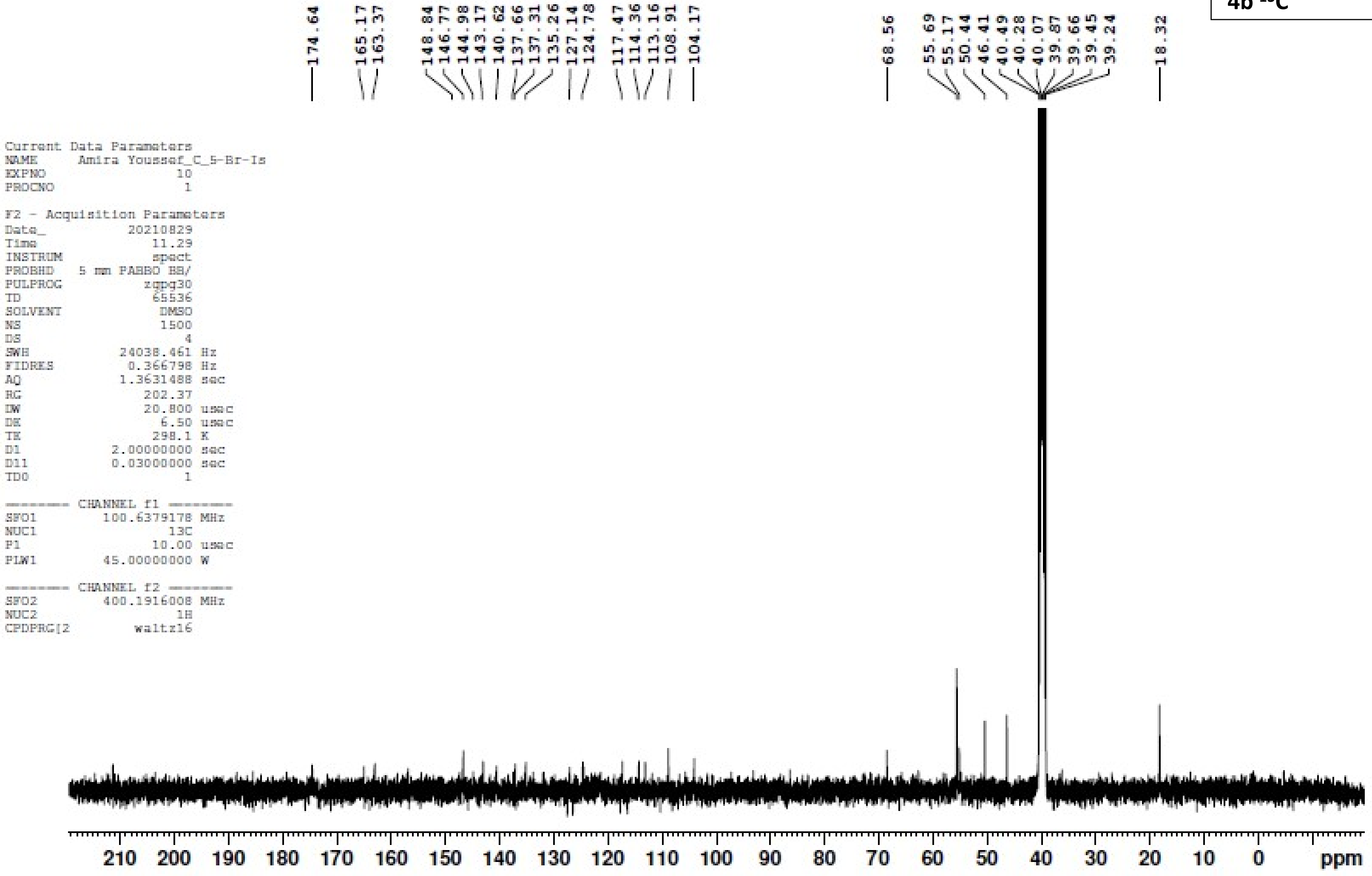

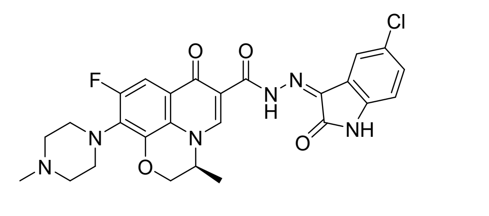

4c IR

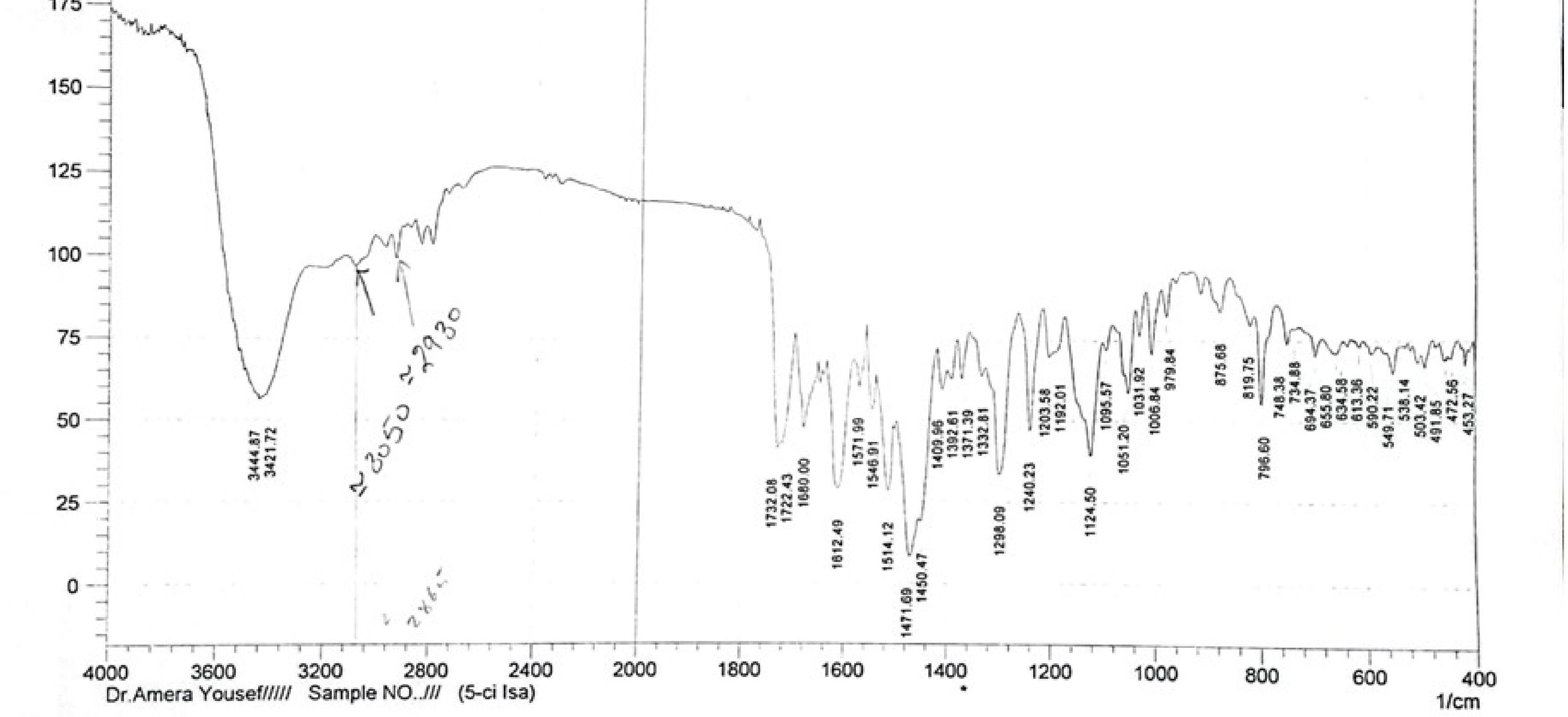

Comment;
Dr.Amera Yousef///// Sample NO../// (5-ci Isa)
**Prepared By : Dr.Ahmed A.EL-Wakeel , Ph.D.

Date/Time; 4/30/2010 1:54:48 AM
No. of Scans; 10
Resolution; 4 [1/cm]
Apodization; Happ-Genzel
User; Heinrich

Amira Youssef_H_5-Cl-Isa

Microanalytical Unit - FOPCU - NMR laboratory
www.pharma.cu.edu.eg dir-mau.fopcu@pharma.cu.edu.eg

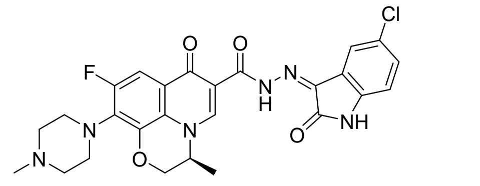

4c NMR

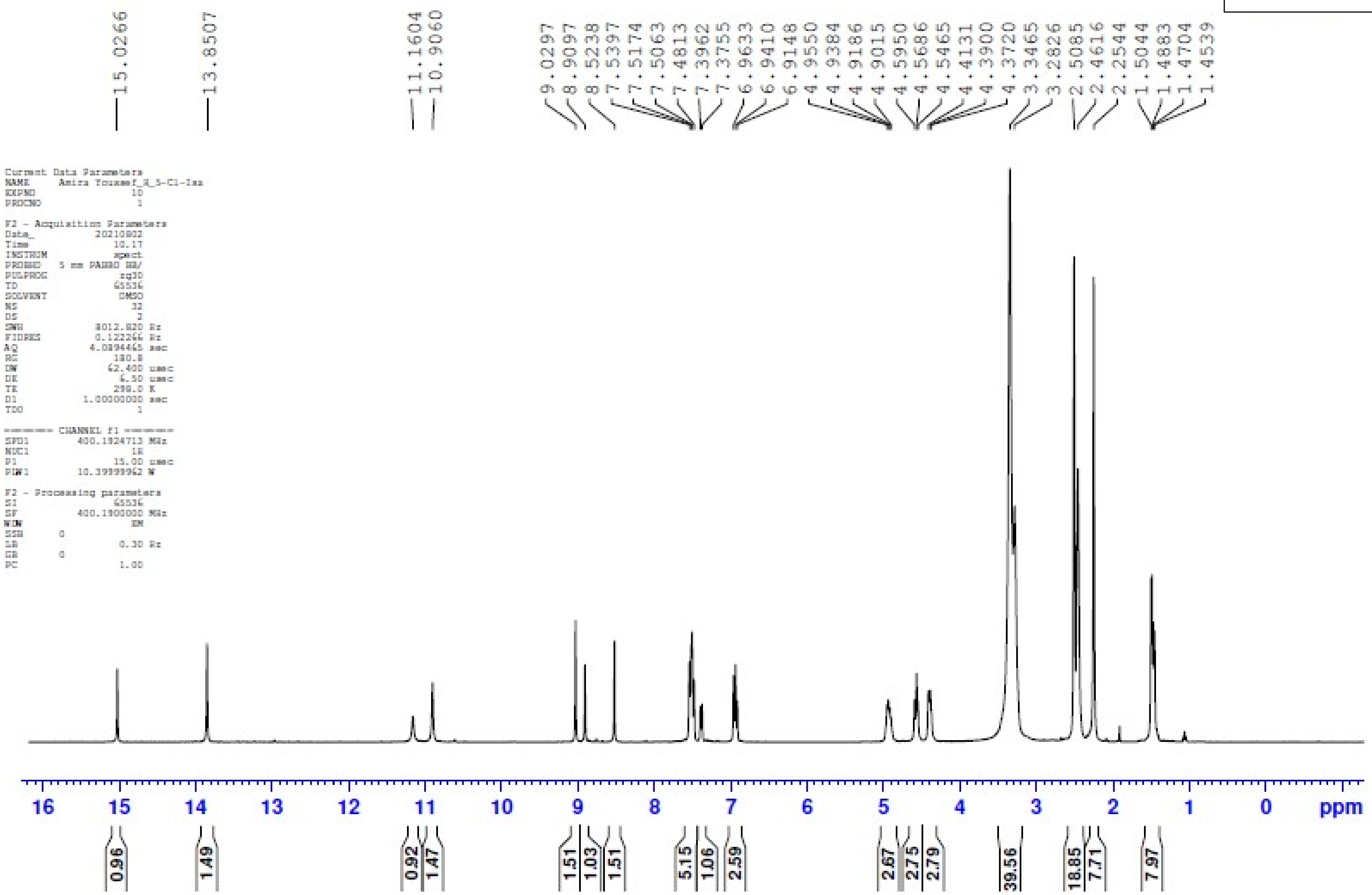

Amira Youssef_H_5-Cl-Isa_D2O

Microanalytical Unit - FOPCU - NMR laboratory
www.pharma.cu.edu.eg dir-mau.fopcu@pharma.cu.edu.eg

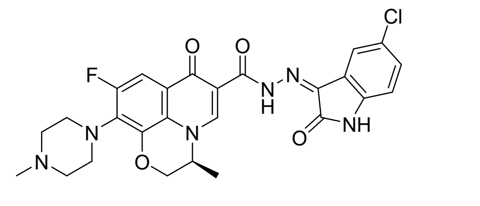

4c D_2O

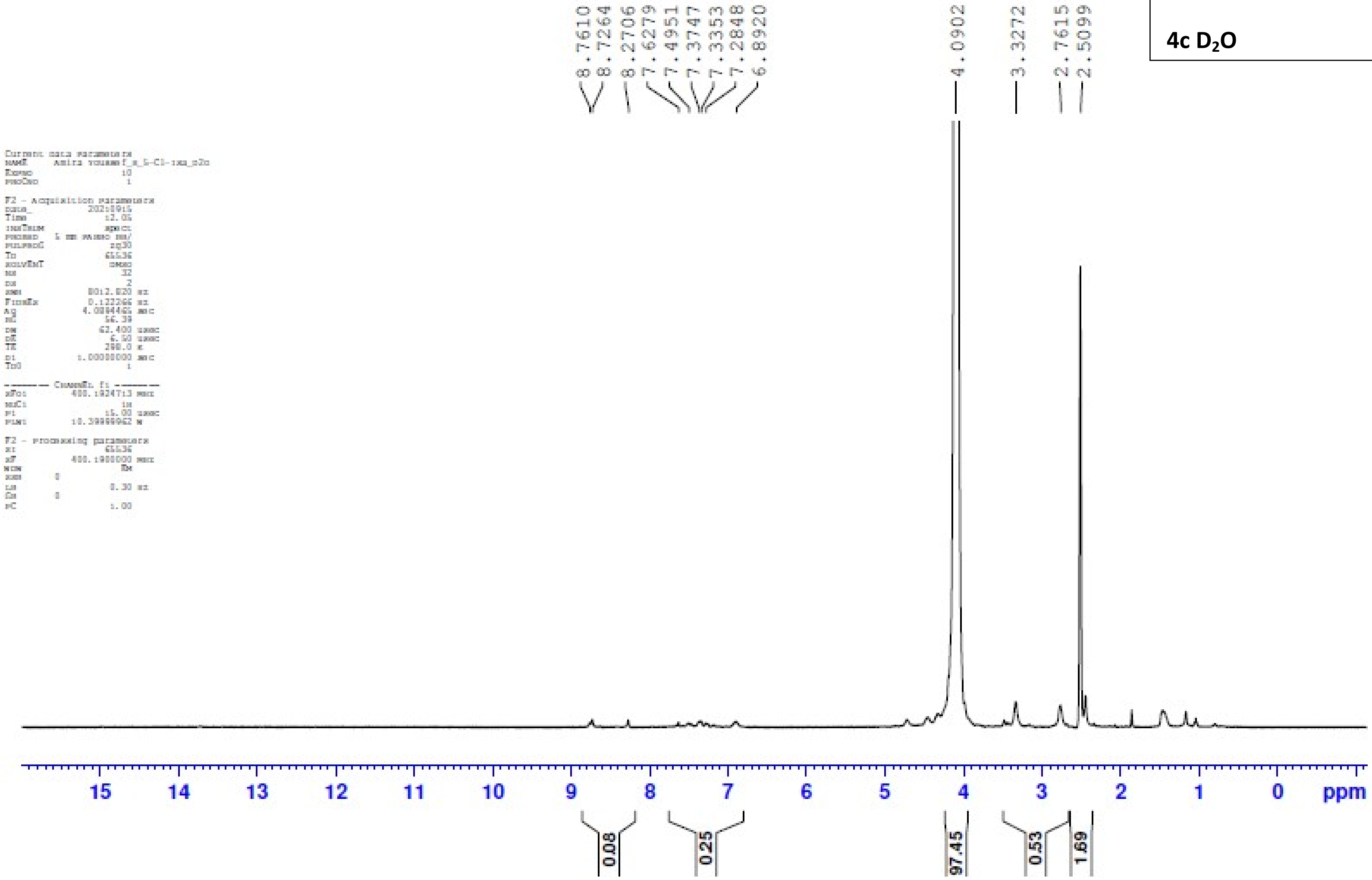

Amira Youssef_C_5-Cl-Isa

Microanalytical Unit - FOPCU - NMR laboratory
www.pharma.cu.edu.eg dir-mau.fopcu@pharma.cu.edu.eg

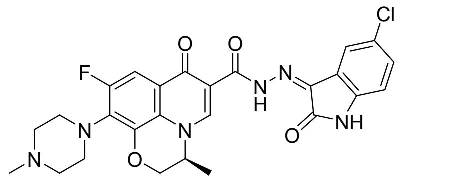

4c ^{13}C

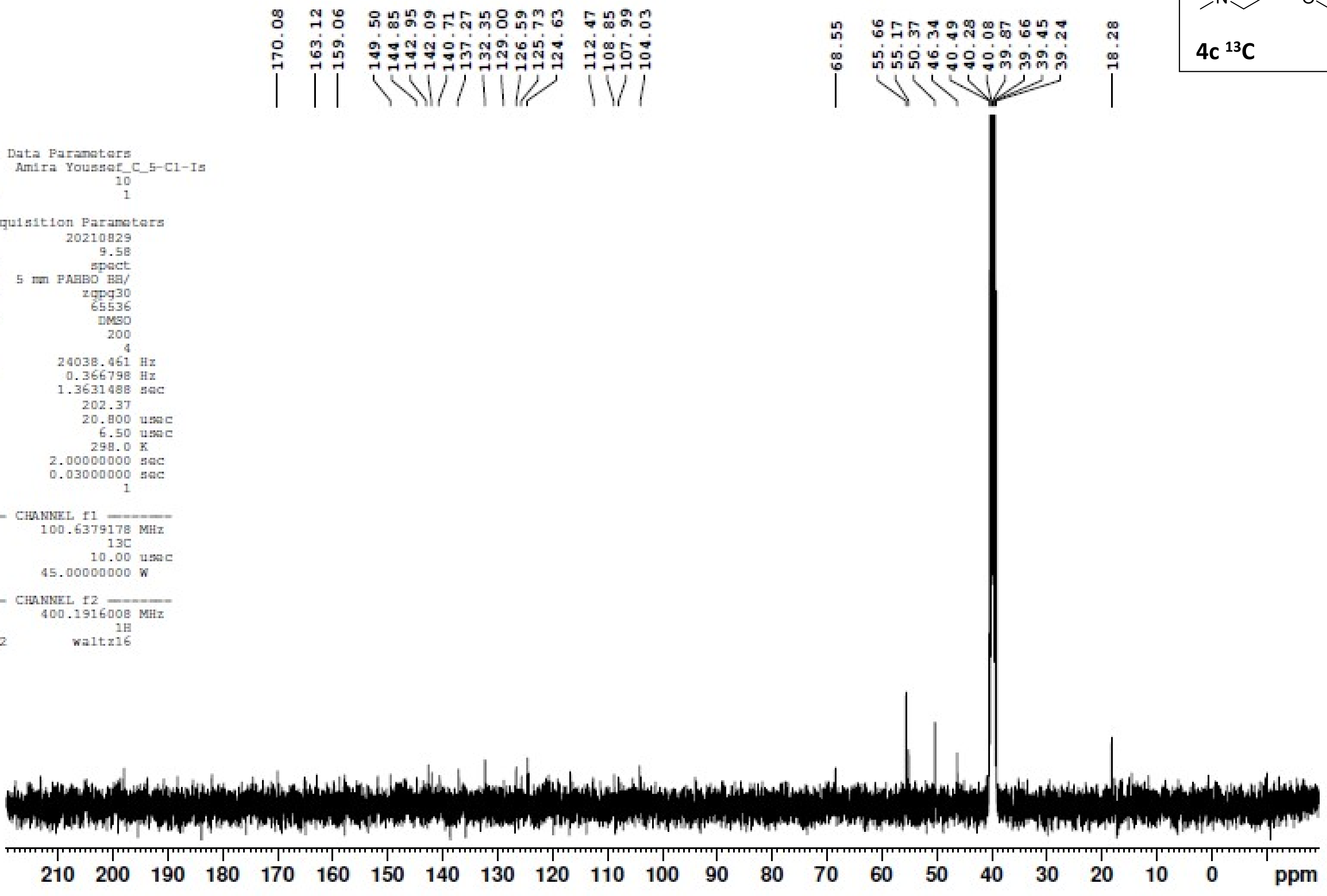


```
Current Data Parameters
NAME     Amira Youssef_C_5-Cl-Is
EXPNO                     10
PROCNO                     1

F2 - Acquisition Parameters
Date_               20210829
Time                    9.58
INSTRUM                spect
PROBHD   5 mm PABBO BB/
PULPROG              zgpg30
TD                     65536
SOLVENT                 DMSO
NS                       200
DS                         4
SWH                24038.461 Hz
FIDRES              0.366798 Hz
AQ                 1.3631488 sec
RG                    202.37
DW                    20.800 usec
DE                      6.50 usec
TE                     298.0 K
D1                2.00000000 sec
D11               0.03000000 sec
TD0                        1

======== CHANNEL f1 ========
SFO1          100.6379178 MHz
NUC1                   13C
P1                   10.00 usec
PLW1           45.00000000 W

======== CHANNEL f2 ========
SFO2          400.1916008 MHz
NUC2                    1H
CPDPRG[2           waltz16
```

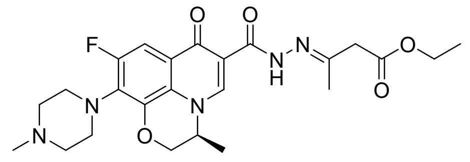

5 IR

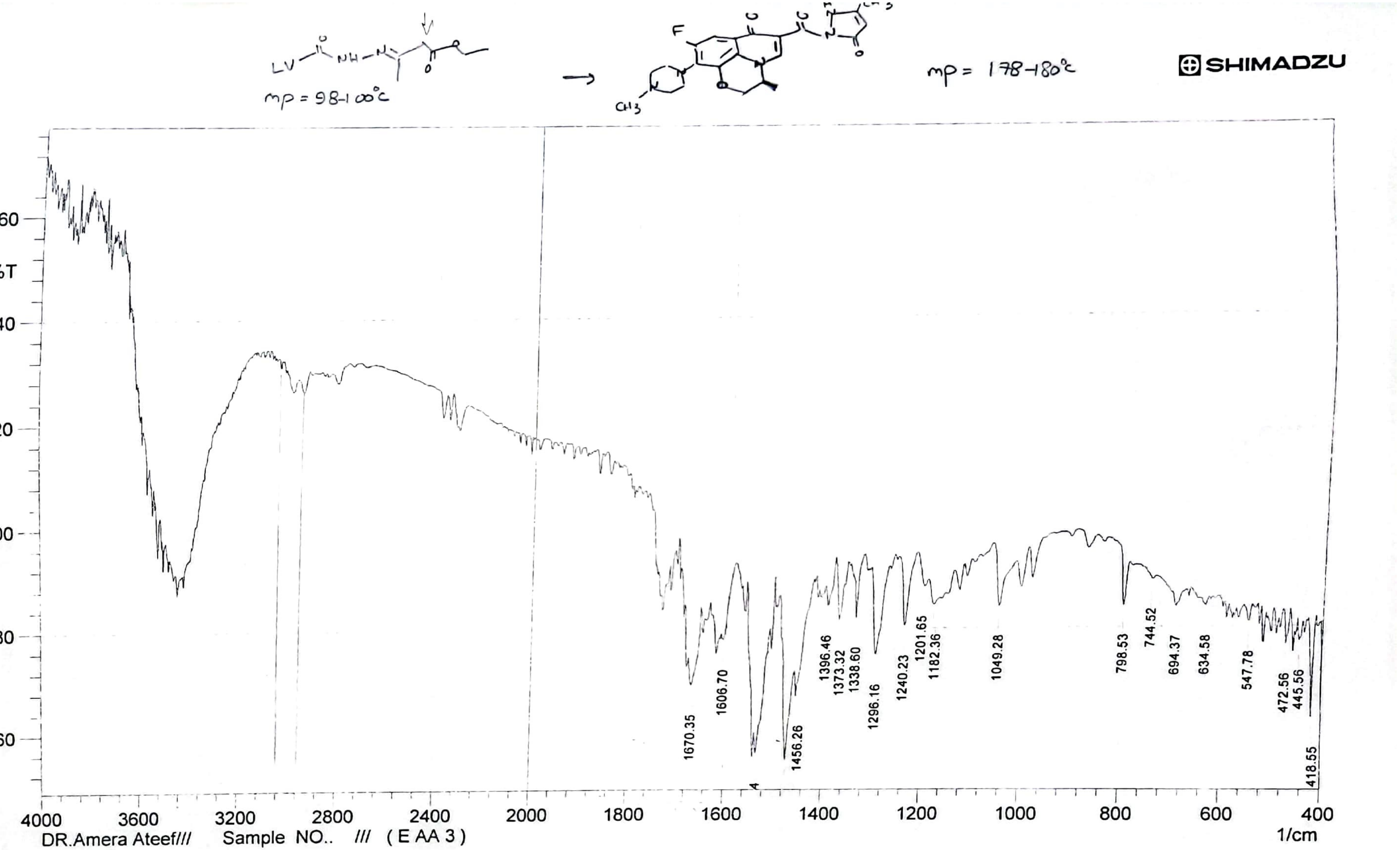

ment;

Date/Time; 4/30/2010 5:19:59 AM

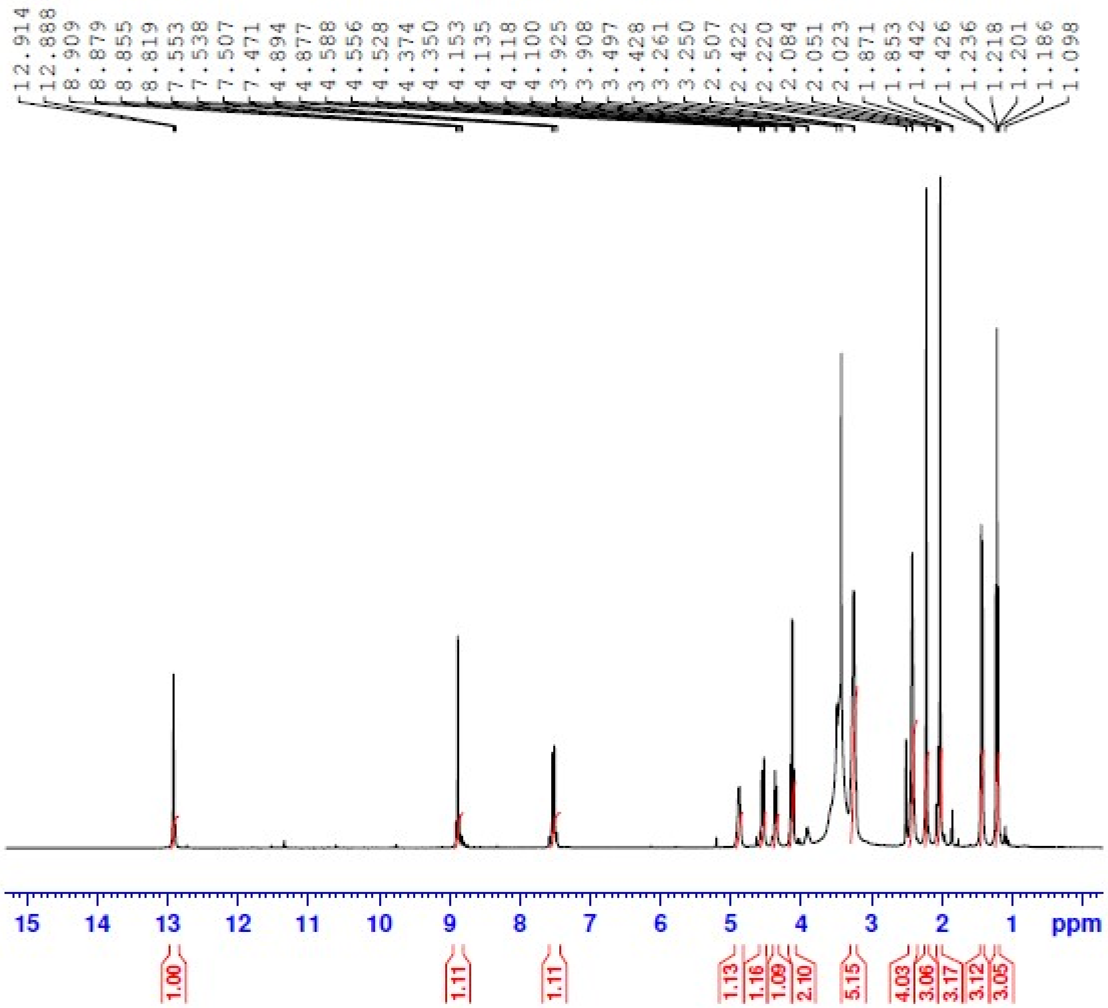

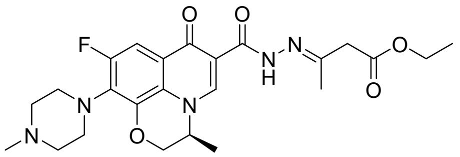

5 NMR

```
Current Data Parameters
NAME      amira-youssef-EAA
EXPNO                     1
PROCNO                    1

F2 - Acquisition Parameters
Date_             20211125
Time                 14.51
INSTRUM              spect
PROBHD   5 mm PABBO BB/
PULPROG               zg30
TD                   65536
SOLVENT               DMSO
NS                      26
DS                       2
SWH              8012.820 Hz
FIDRES           0.122266 Hz
AQ              4.0894465 sec
RG                  205.37
DW                  62.400 usec
DE                    6.50 usec
TE                   300.0 K
D1              1.00000000 sec
TD0                      1

======== CHANNEL f1 ========
SFO1           400.1524711 MHz
NUC1                    1H
P1                   12.00 usec
PLW1           18.00000000 W

F2 - Processing parameters
SI                   65536
SF             400.1500000 MHz
WDW                     EM
SSB      0
LB                    0.30 Hz
GB       0
PC                    1.00
```

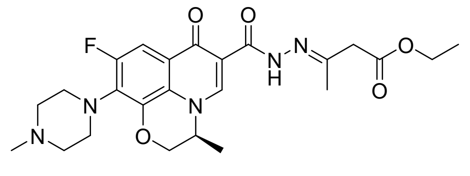

5 D_2O

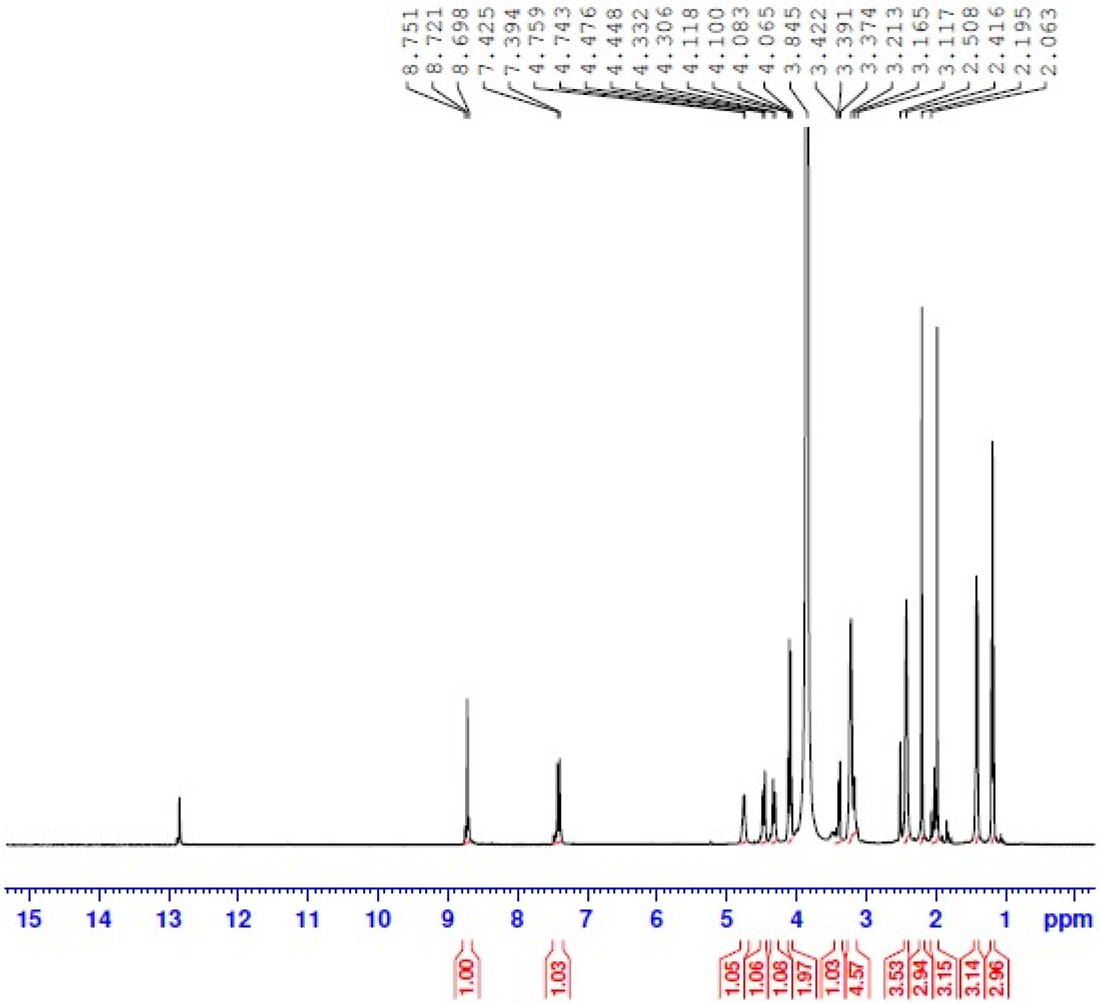


```
Current Data Parameters
NAME      amira-youssef-EAA-d2o
EXPNO                     1
PROCNO                    1

F2 - Acquisition Parameters
Date_              20211212
Time                  15.56
INSTRUM               spect
PROBHD    5 mm PABBO BB/
PULPROG                zg30
TD                    65536
SOLVENT                DMSO
NS                       16
DS                        2
SWH                8012.820 Hz
FIDRES             0.122266 Hz
AQ                4.0894465 sec
RG                   117.48
DW                   62.400 usec
DE                     6.50 usec
TE                    300.0 K
D1               1.00000000 sec
TD0                       1

======== CHANNEL f1 ========
SFO1            400.1524711 MHz
NUC1                     1H
P1                    12.00 usec
PLW1            18.00000000 W

F2 - Processing parameters
SI                    65536
SF              400.1500000 MHz
WDW                      EM
SSB       0
LB                     0.30 Hz
GB        0
PC                     1.00
```

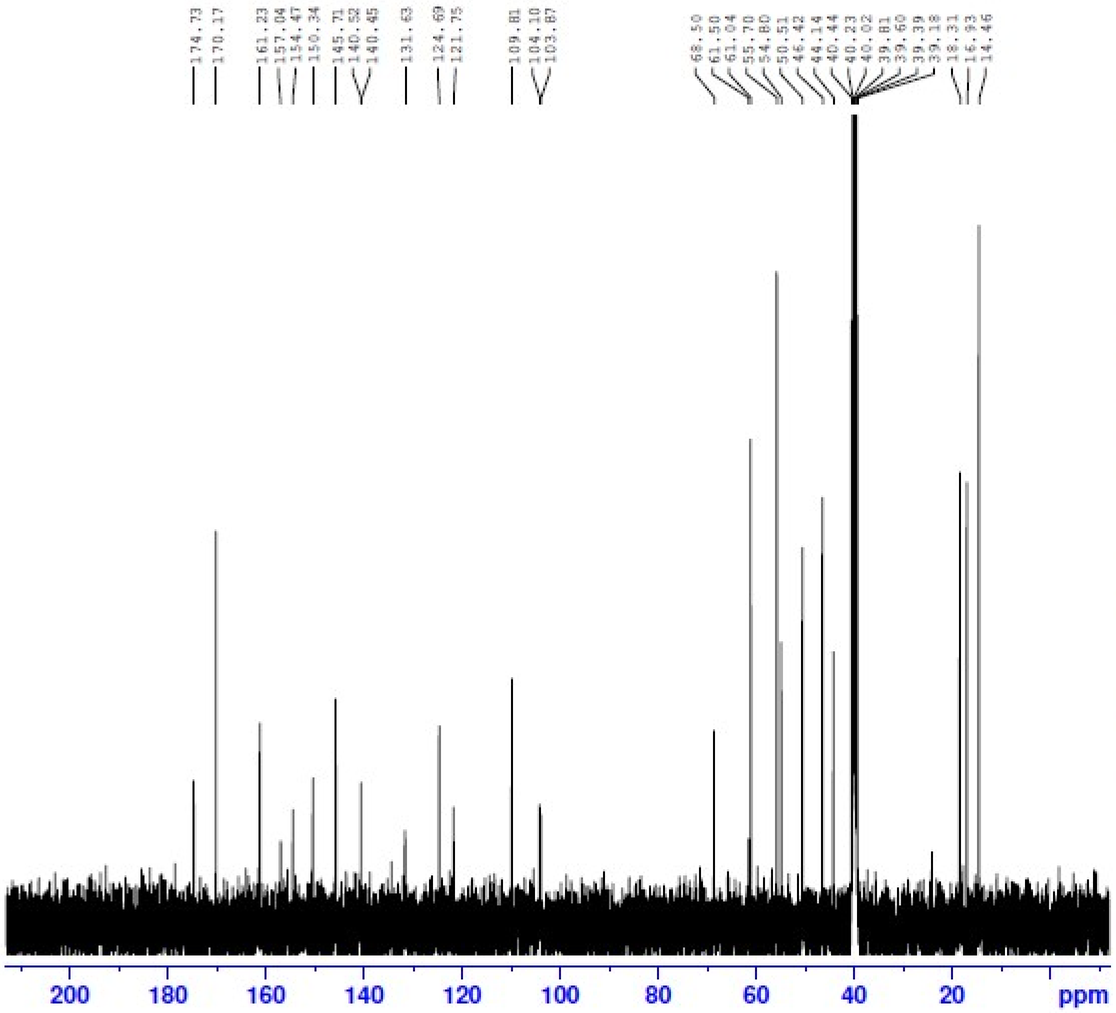

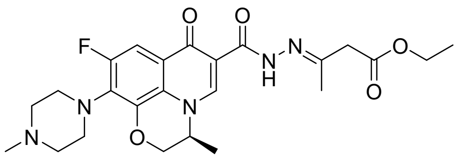

5 ^{13}C

```
Current Data Parameters
NAME       amira-youssef-EAA
EXPNO                      2
PROCNO                     1

F2 - Acquisition Parameters
Date_             20211212
Time                 13.57
INSTRUM              spect
PROBHD     5 mm PABBO BB/
PULPROG             zgpg30
TD                   65536
SOLVENT               DMSO
NS                     643
DS                       4
SWH              24038.461 Hz
FIDRES            0.366798 Hz
AQ               1.3631488 sec
RG                  205.37
DW                  20.800 usec
DE                    6.50 usec
TE                   300.0 K
D1              2.00000000 sec
D11             0.03000000 sec
TD0                      1

======== CHANNEL f1 ========
SFO1        100.6278588 MHz
NUC1                    13C
P1                    10.00 usec
PLW1         47.00000000 W

======== CHANNEL f2 ========
SFO2        400.1516006 MHz
NUC2                     1H
CPDPRG[2            waltz16
PCPD2                 90.00 usec
PLW2         18.00000000 W
PLW12         0.34722000 W
PLW13         0.28125000 W

F2 - Processing parameters
SI                    32768
SF              100.6177975 MHz
WDW                      EM
SSB      0
LB                     1.00 Hz
GB       0
PC                     1.40
```

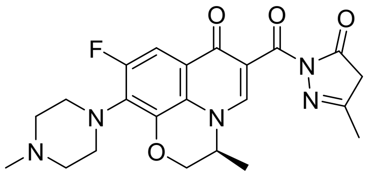
F
O
O
O
N
N
N
N
N
O

6 IR

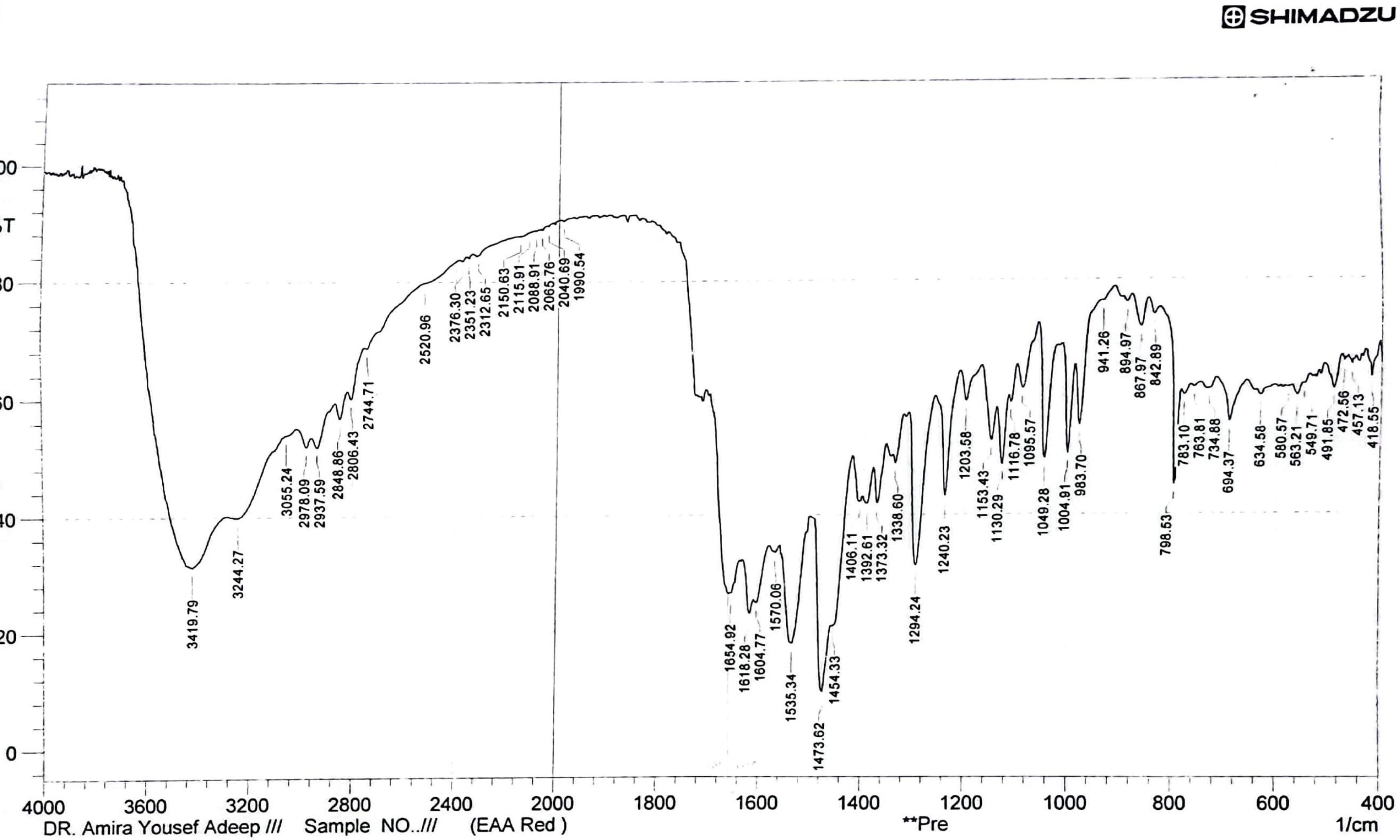
SHIMADZU
%T
3419.79
3244.27
3055.24
2978.09
2937.59
2848.86
2806.43
2744.71
2520.96
2376.30
2351.23
2312.65
2150.63
2115.91
2088.91
2065.76
2040.69
1990.54
1654.92
1618.28
1604.77
1570.06
1535.34
1473.62
1454.33
1406.11
1392.61
1373.32
1338.60
1294.24
1240.23
1203.58
1153.43
1130.29
1116.78
1095.57
1049.28
1004.91
983.70
941.26
894.97
867.97
842.89
798.53
783.10
763.81
734.88
694.37
634.58
580.57
563.21
549.71
491.85
472.56
457.13
418.55
4000
3600
3200
2800
2400
2000
1800
1600
1400
1200
1000
800
600
400
DR. Amira Yousef Adeep /// Sample NO../// (EAA Red)
**Pre
1/cm

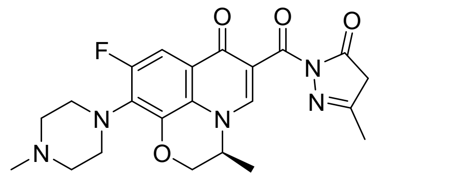

6 NMR

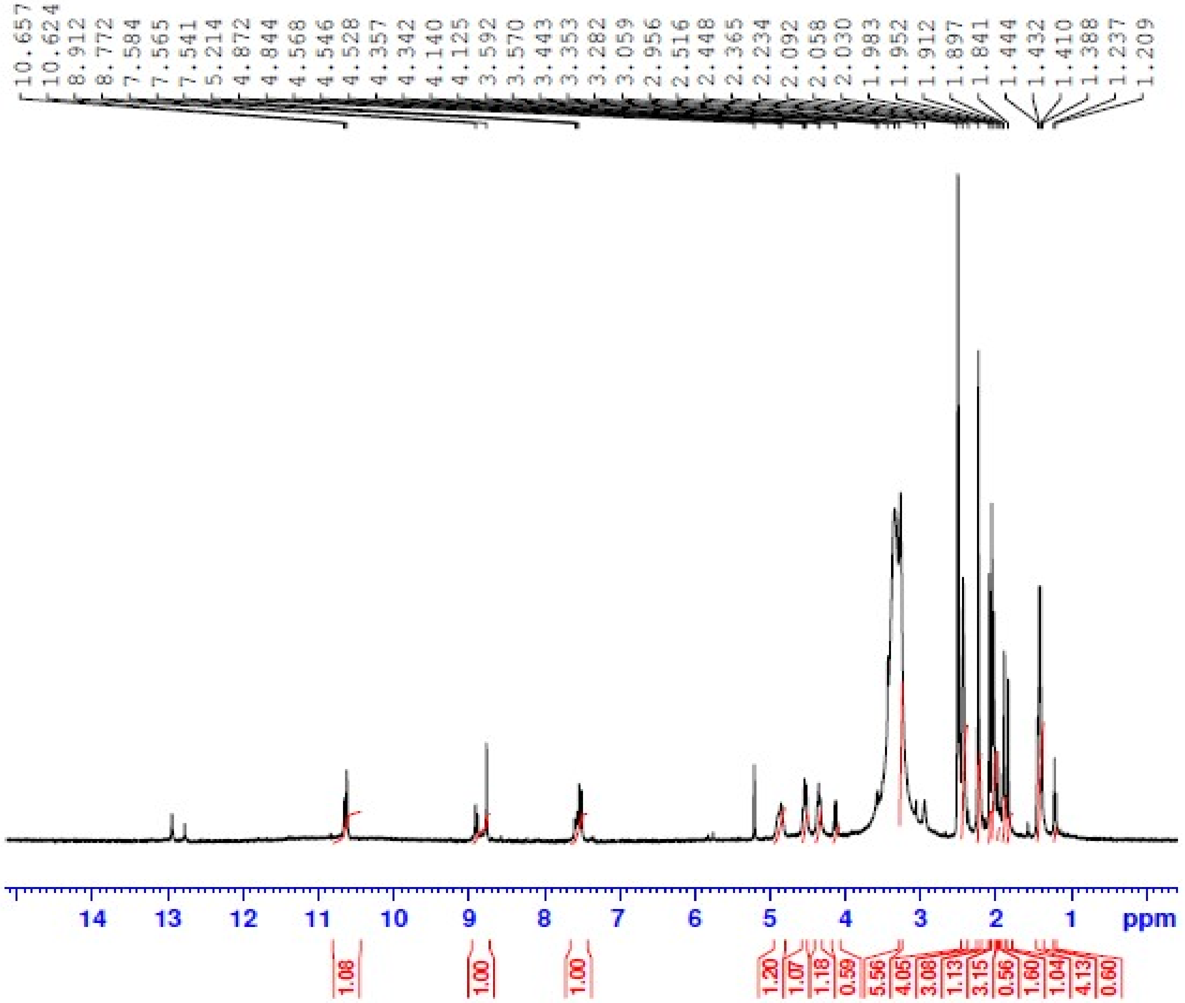

Current Data Parameters
NAME amira-youssef-KAA-red
EXPNO 1
PROCNO 1

F2 - Acquisition Parameters
Date_ 20220119
Time 16.29
INSTRUM spect
PROBHD 5 mm PABBO BB/
PULPROG zg30
TD 65536
SOLVENT DMSO
NS 32
DS 2
SWH 8012.820 Hz
FIDRES 0.122266 Hz
AQ 4.0894465 sec
RG 31.47
DW 62.400 usec
DE 6.50 usec
TE 300.0 K
D1 1.00000000 sec
TD0 1

======== CHANNEL f1 ========
SFO1 400.1524711 MHz
NUC1 1H
P1 12.00 usec
PLW1 18.00000000 W

F2 - Processing parameters
SI 65536
SF 400.1500000 MHz
WDW EM
SSB 0
LB 0.30 Hz
GB 0
PC 1.00

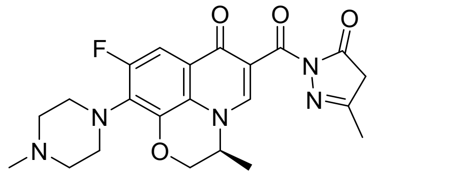

6 D_2O

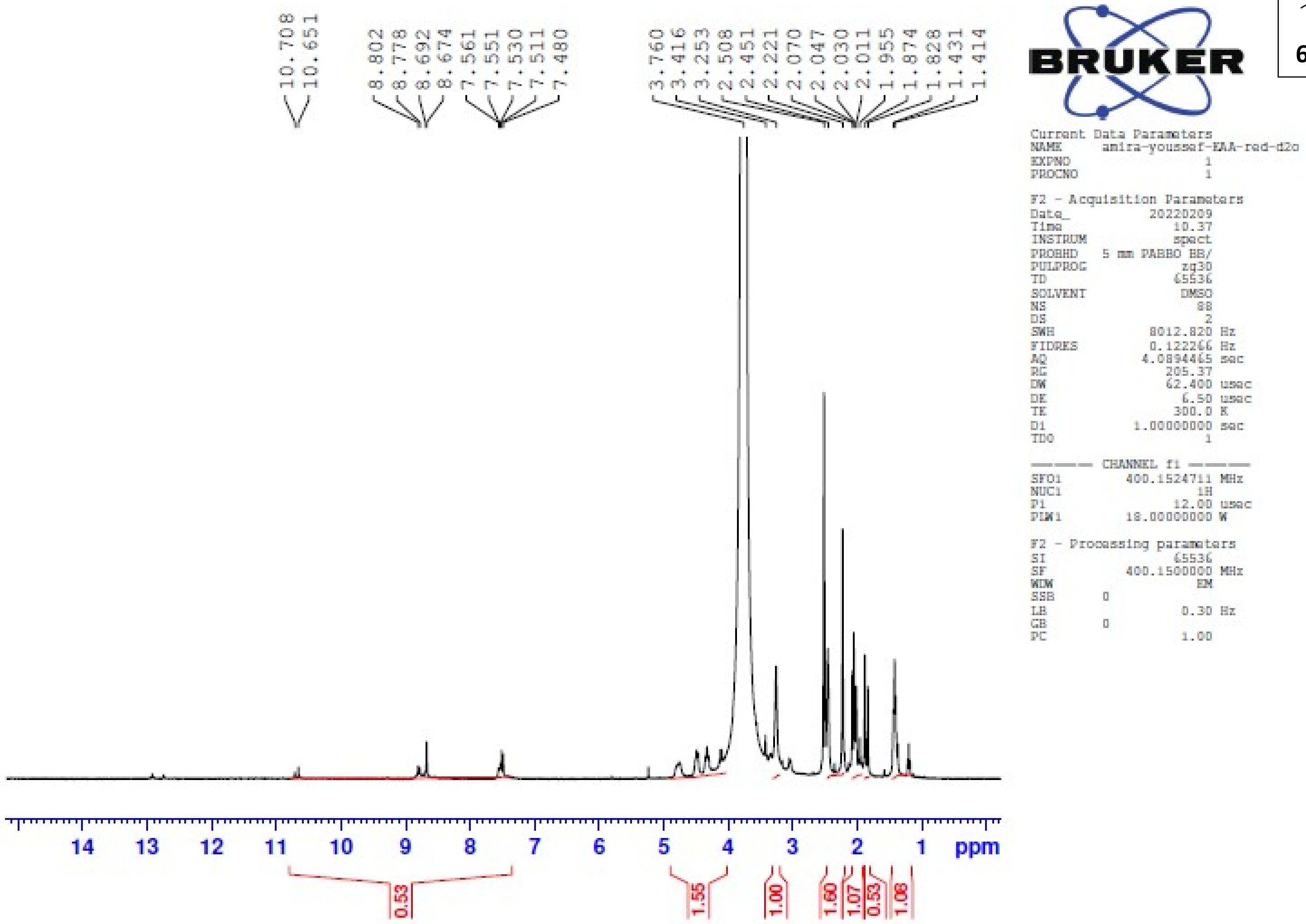
10.708
10.651
8.802
8.778
8.692
8.674
7.561
7.551
7.530
7.511
7.480
3.760
3.416
3.253
2.508
2.451
2.221
2.070
2.047
2.030
2.011
1.955
1.874
1.828
1.431
1.414
BRUKER
Current Data Parameters
NAME amira-youssef-KAA-red-d2o
EXPNO 1
PROCNO 1
F2 - Acquisition Parameters
Date_ 20220209
Time 10.37
INSTRUM spect
PROBHD 5 mm PABBO BB/
PULPROG zg30
TD 65536
SOLVENT DMSO
NS 88
DS 2
SWH 8012.820 Hz
FIDRES 0.122266 Hz
AQ 4.0894465 sec
RG 205.37
DW 62.400 usec
DE 6.50 usec
TE 300.0 K
D1 1.00000000 sec
TD0 1
CHANNEL f1
SFO1 400.1524711 MHz
NUC1 1H
P1 12.00 usec
PLW1 18.00000000 W
F2 - Processing parameters
SI 65536
SF 400.1500000 MHz
WDW EM
SSB 0
LB 0.30 Hz
GB 0
PC 1.00
14
13
12
11
10
9
8
7
6
5
4
3
2
1
ppm
0.53
1.55
1.00
1.60
1.07
0.53
1.08

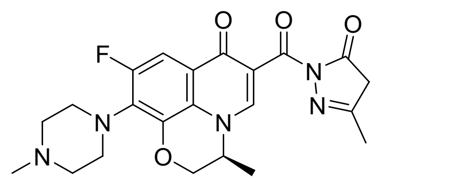

6 ^{13}C

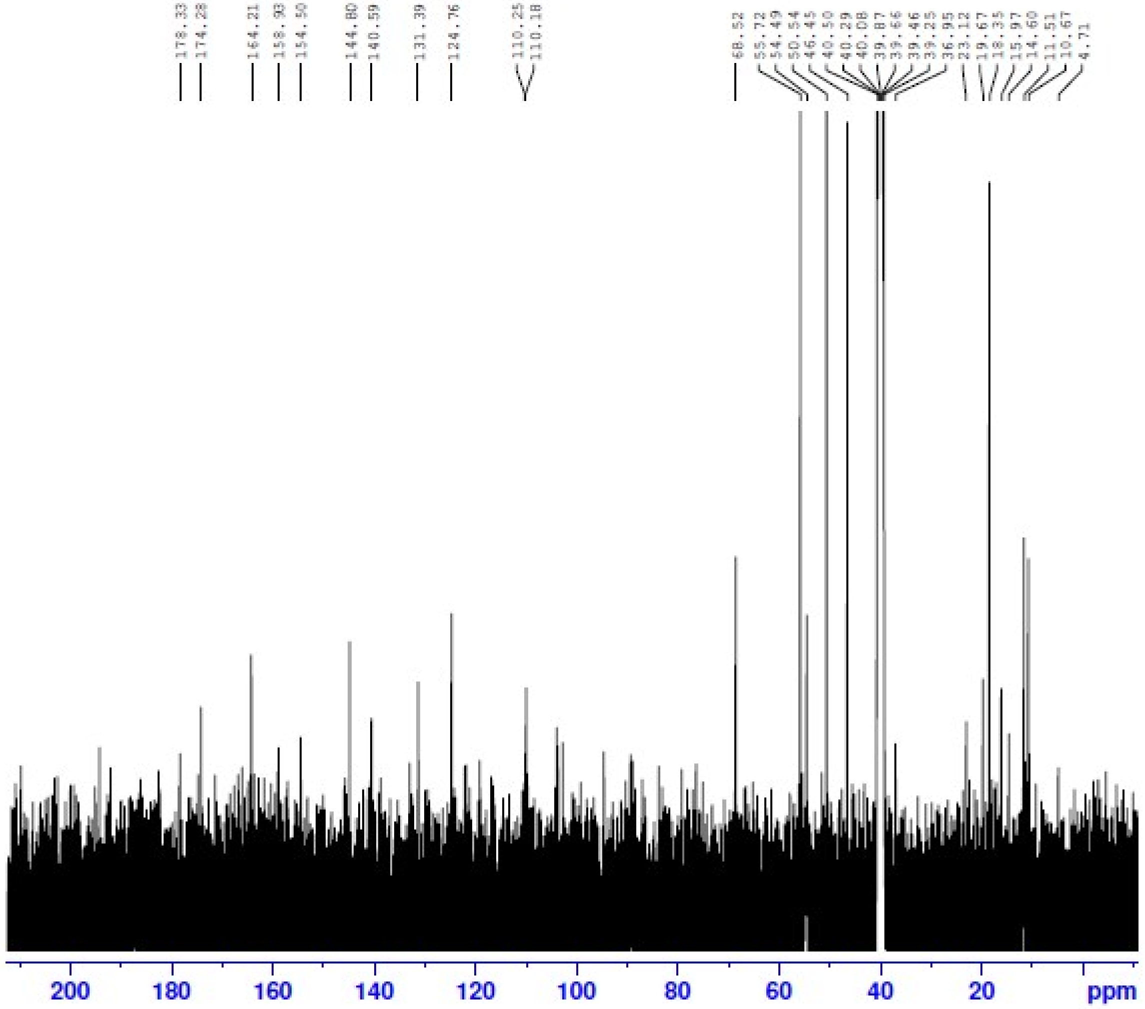

BRUKER

Current Data Parameters
NAME amira-youssef-KAA-red
EXPNO 2
PROCNO 1

F2 - Acquisition Parameters
Date_ 20220209
Time 6.09
INSTRUM spect
PROBHD 5 mm PABBO BB/
PULPROG zgpg30
TD 65536
SOLVENT DMSO
NS 15000
DS 4
SWH 24038.461 Hz
FIDRES 0.366798 Hz
AQ 1.3631488 sec
RG 205.37
DW 20.800 usec
DE 6.50 usec
TE 299.9 K
D1 2.00000000 sec
D11 0.03000000 sec
TD0 1

======== CHANNEL f1 ========
SFO1 100.6278588 MHz
NUC1 13C
P1 10.00 usec
PLW1 47.00000000 W

======== CHANNEL f2 ========
SFO2 400.1516006 MHz
NUC2 1H
CPDPRG[2 waltz16
PCPD2 90.00 usec
PLW2 18.00000000 W
PLW12 0.34722000 W
PLW13 0.28125000 W

F2 - Processing parameters
SI 32768
SF 100.6177975 MHz
WDW EM
SSB 0
LB 1.00 Hz
GB 0
PC 1.40

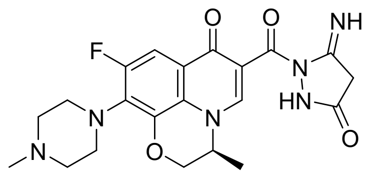
O
O
NH
F
N
N
HN
N
O
O

7 IR

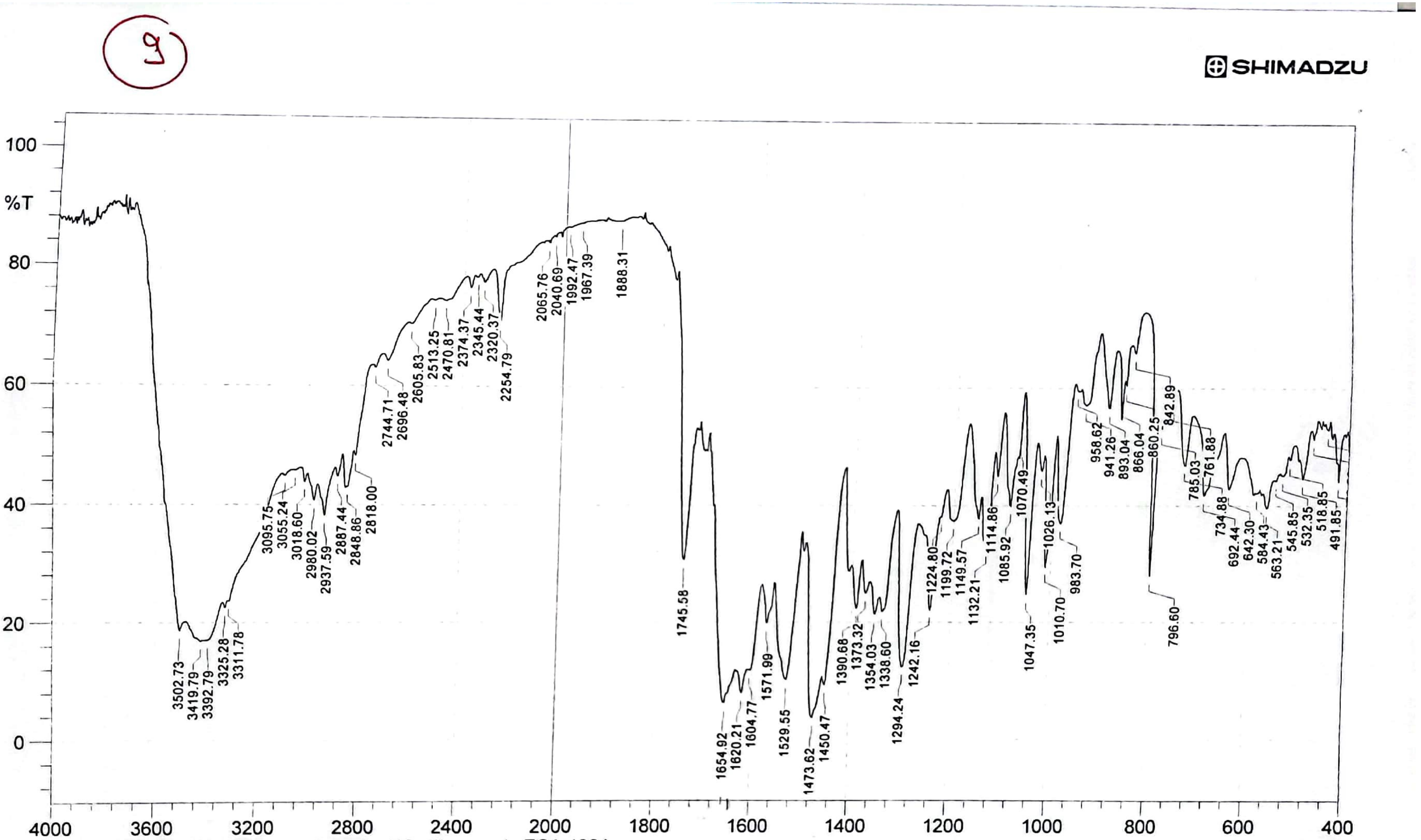
9
SHIMADZU
%T
100
80
60
40
20
0
4000
3600
3200
2800
2400
2000
1800
1600
1400
1200
1000
800
600
400
3502.73
3419.79
3392.79
3325.28
3311.78
3095.75
3055.24
3018.60
2980.02
2937.59
2887.44
2848.86
2818.00
2744.71
2696.48
2605.83
2513.25
2470.81
2374.37
2345.44
2320.37
2254.79
2065.76
2040.69
1992.47
1967.39
1888.31
1745.58
1654.92
1620.21
1604.77
1571.99
1529.55
1473.62
1450.47
1390.68
1373.32
1354.03
1338.60
1294.24
1242.16
1224.80
1199.72
1149.57
1132.21
1114.86
1085.92
1070.49
1047.35
1026.13
1010.70
983.70
958.62
941.26
893.04
866.04
860.25
842.89
796.60
785.03
761.88
734.88
692.44
642.30
584.43
563.21
545.85
532.35
518.85
491.85

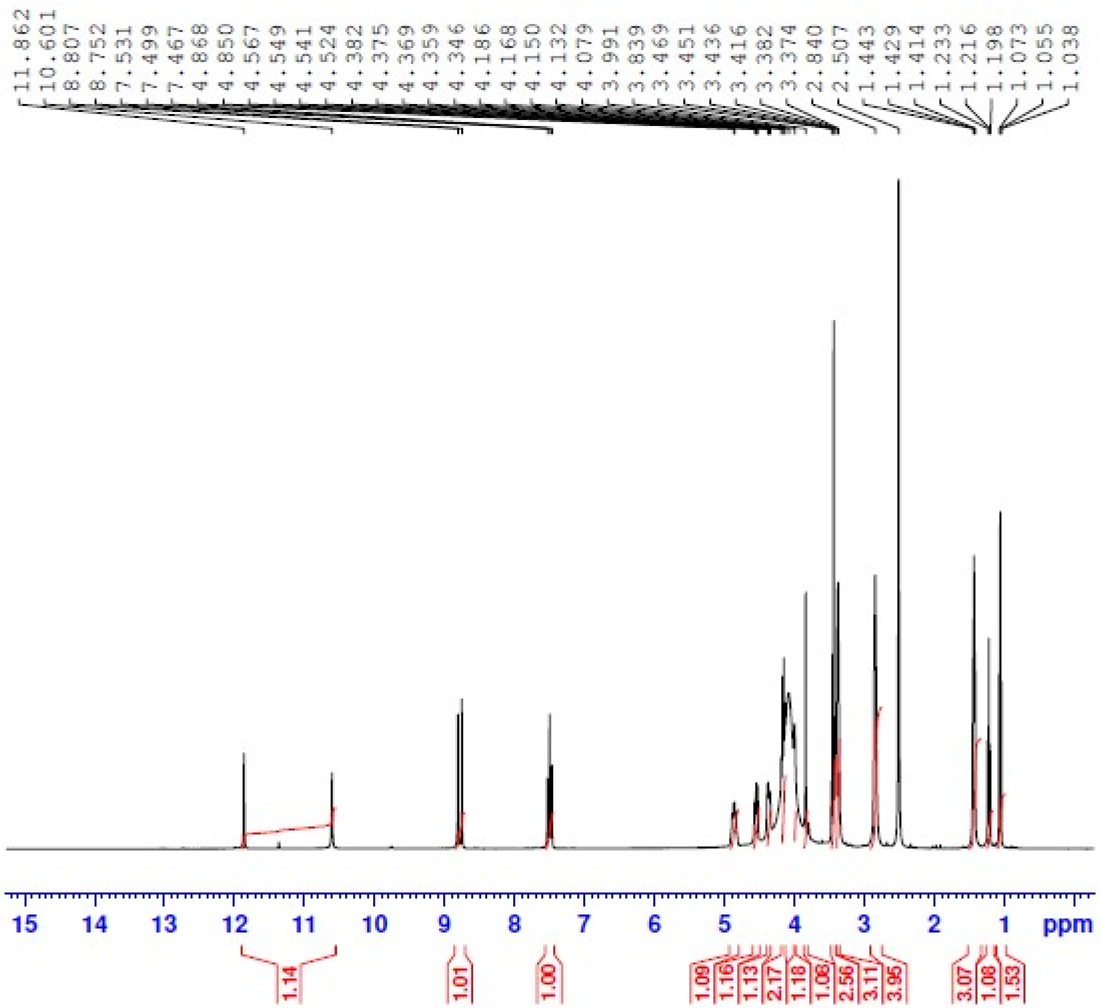

Current Data Parameters
NAME amira-youssef-EcyA
EXPNO 1
PROCNO 1

F2 - Acquisition Parameters
Date_ 20220426
Time 10.38
INSTRUM spect
PROBHD 5 mm PABBO BB/
PULPROG zg30
TD 65536
SOLVENT DMSO
NS 97
DS 2
SWH 8012.820 Hz
FIDRES 0.122266 Hz
AQ 4.0894465 sec
RG 205.37
DW 62.400 usec
DE 6.50 usec
TE 300.0 K
D1 1.00000000 sec
TD0 1

======== CHANNEL f1 ========
SFO1 400.1524711 MHz
NUC1 1H
P1 12.00 usec
PLW1 18.00000000 W

F2 - Processing parameters
SI 65536
SF 400.1500000 MHz
WDW EM
SSB 0
LB 0.30 Hz
GB 0
PC 1.00

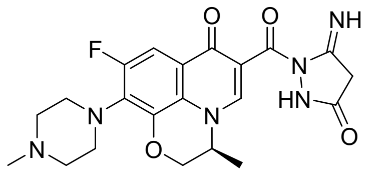

7 NMR

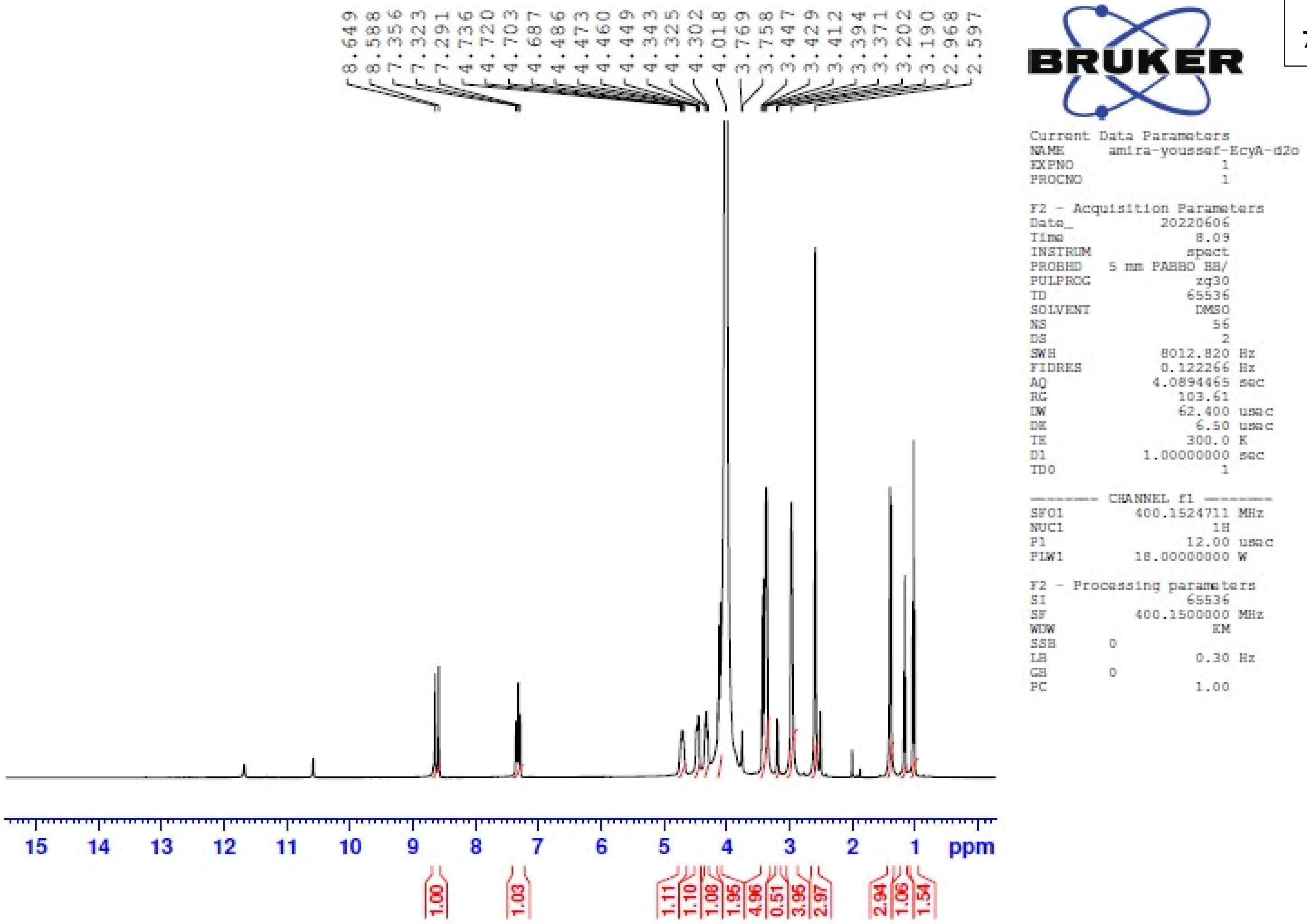
8.649
8.588
7.356
7.323
7.291
4.736
4.720
4.703
4.687
4.486
4.473
4.460
4.449
4.343
4.325
4.302
4.018
3.769
3.758
3.447
3.429
3.412
3.394
3.371
3.202
3.190
2.968
2.597
BRUKER
Current Data Parameters
NAME amira-youssef-EcyA-d2o
EXPNO 1
PROCNO 1
F2 - Acquisition Parameters
Date_ 20220606
Time 8.09
INSTRUM spect
PROBHD 5 mm PABBO BB/
PULPROG zg30
TD 65536
SOLVENT DMSO
NS 56
DS 2
SWH 8012.820 Hz
FIDRES 0.122266 Hz
AQ 4.0894465 sec
RG 103.61
DW 62.400 usec
DE 6.50 usec
TE 300.0 K
D1 1.00000000 sec
TD0 1
======== CHANNEL f1 ========
SFO1 400.1524711 MHz
NUC1 1H
P1 12.00 usec
PLW1 18.00000000 W
F2 - Processing parameters
SI 65536
SF 400.1500000 MHz
WDW EM
SSB 0
LB 0.30 Hz
GB 0
PC 1.00
15 14 13 12 11 10 9 8 7 6 5 4 3 2 1 ppm
1.00
1.03
1.11
1.10
1.08
1.95
4.96
0.51
3.95
2.97
2.94
1.06
1.54

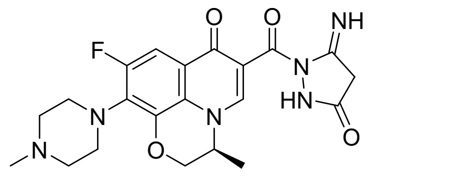
F
O
O
NH
N
N
HN
N
O
N
O

7 D$_2$O

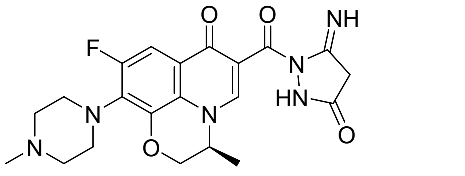

F
O
O
NH
N
N
N
HN
O
N
O

7 ^{13}C

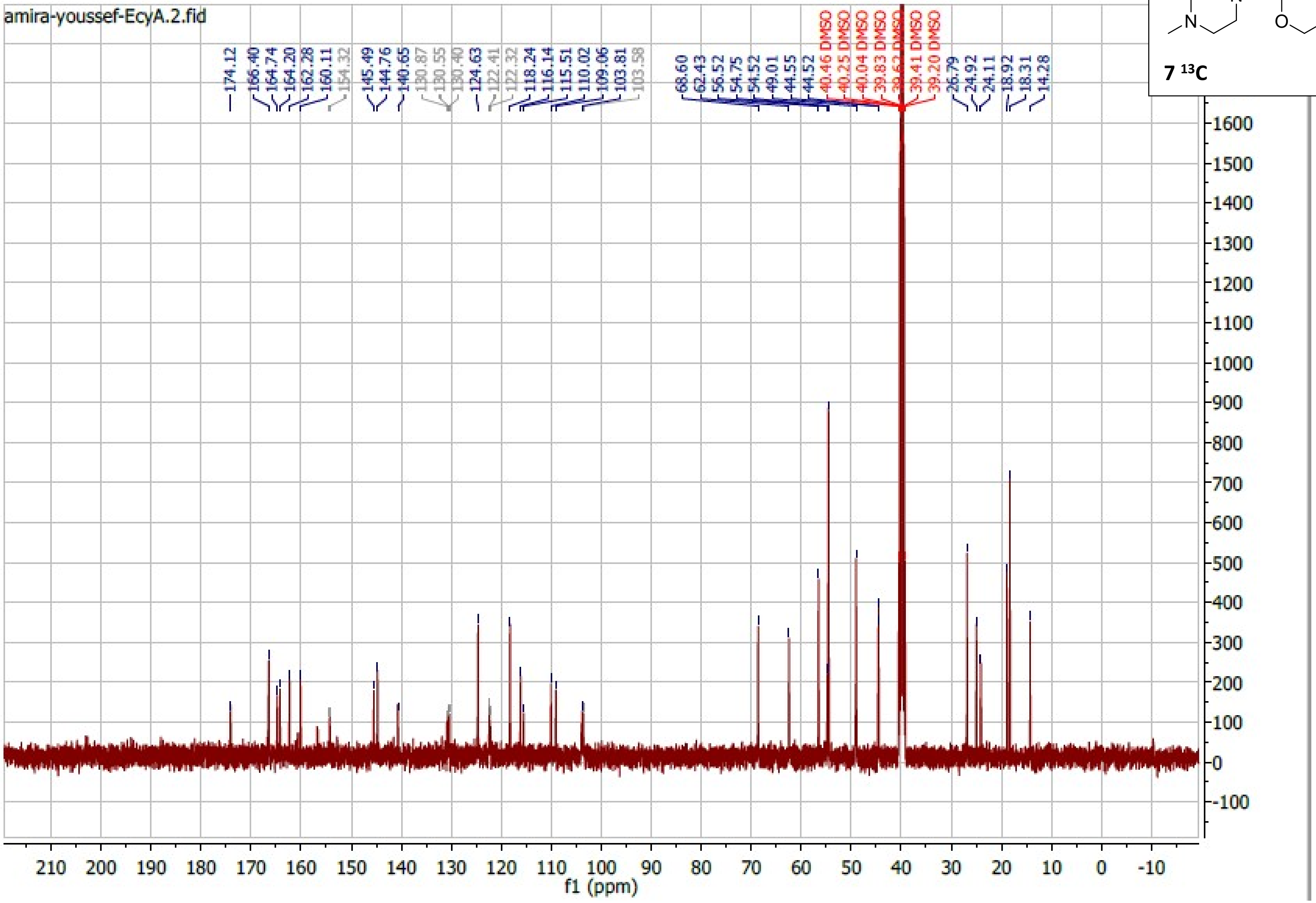

amira-youssef-EcyA.2.fid
174.12
166.40
164.74
164.20
162.28
160.11
154.32
145.49
144.76
140.65
130.87
130.55
130.40
124.63
122.41
122.32
118.24
116.14
115.51
110.02
109.06
103.81
103.58
68.60
62.43
56.52
54.75
54.52
49.01
44.55
44.52
40.46 DMSO
40.25 DMSO
40.04 DMSO
39.83 DMSO
39.62 DMSO
39.41 DMSO
39.20 DMSO
26.79
24.92
24.11
18.92
18.31
14.28
210 200 190 180 170 160 150 140 130 120 110 100 90 80 70 60 50 40 30 20 10 0 -10
f1 (ppm)
1600 1500 1400 1300 1200 1100 1000 900 800 700 600 500 400 300 200 100 0 -100

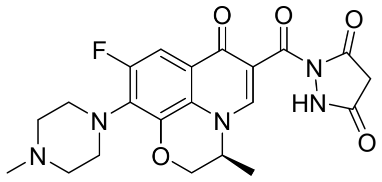
F
N
O
N
O
O
N
HN
O
O

8 IR

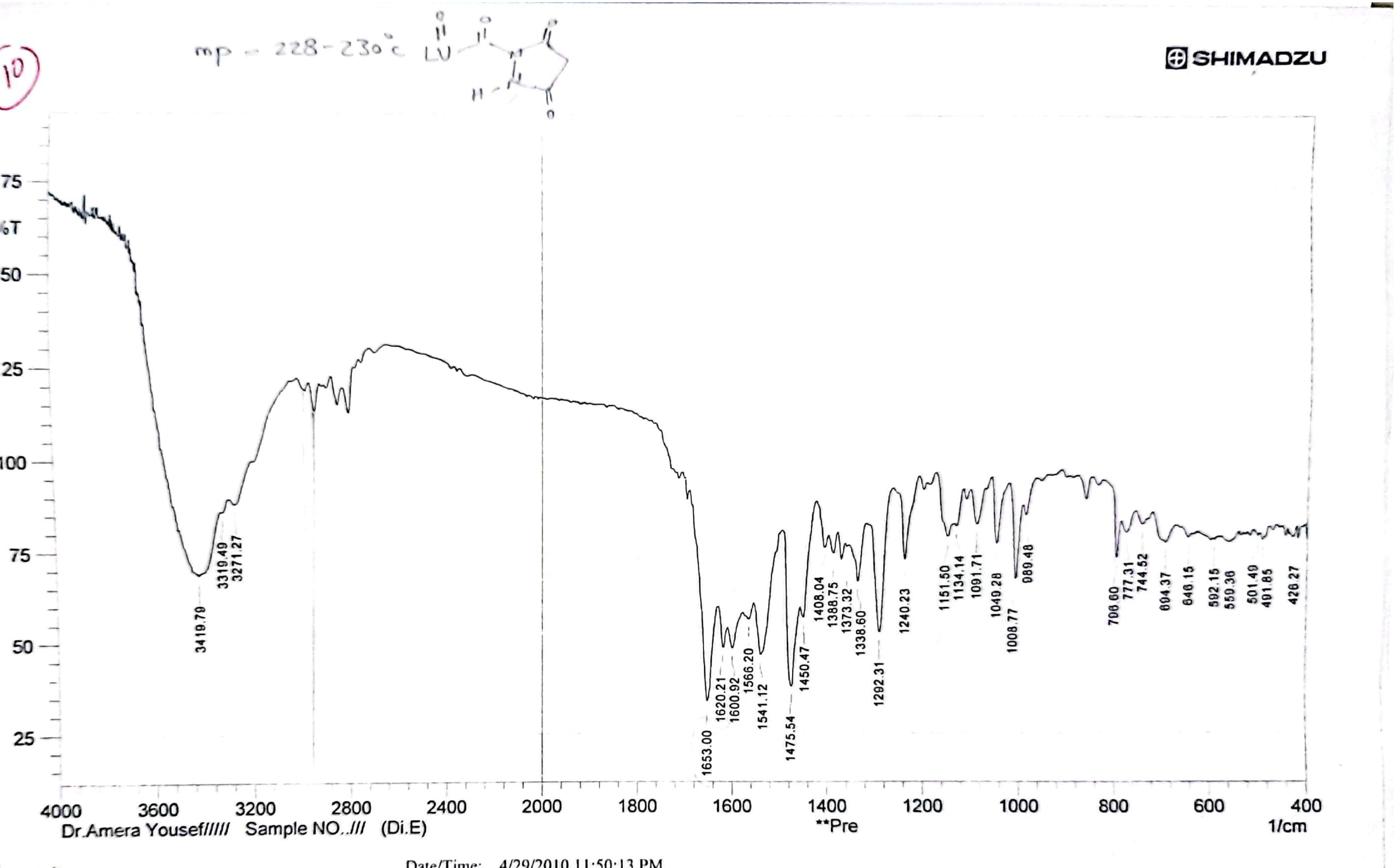
mp - 228-230°c
SHIMADZU
%T
75
50
25
100
75
50
25
3419.70
3319.49
3271.27
1653.00
1620.21
1600.92
1566.20
1541.12
1475.54
1450.47
1408.04
1388.75
1373.32
1338.60
1292.31
1240.23
1151.50
1134.14
1091.71
1049.28
1008.77
989.48
796.60
777.31
744.52
694.37
640.15
592.15
559.36
501.49
491.85
420.27
4000
3600
3200
2800
2400
2000
1800
1600
1400
1200
1000
800
600
400
1/cm
Dr.Amera Yousef////// Sample NO../// (Di.E)
**Pre
Date/Time: 4/29/2010 11:50:13 PM

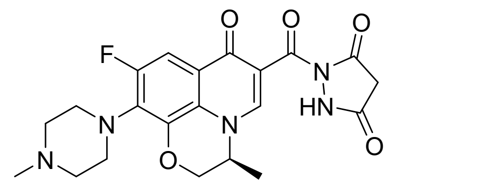

8 NMR

Amira Youssef_H_Malon

Microanalytical Unit - FOPCU - NMR laboratory
www.pharma.cu.edu.eg dir-mau.fopcu@pharma.cu.edu.eg

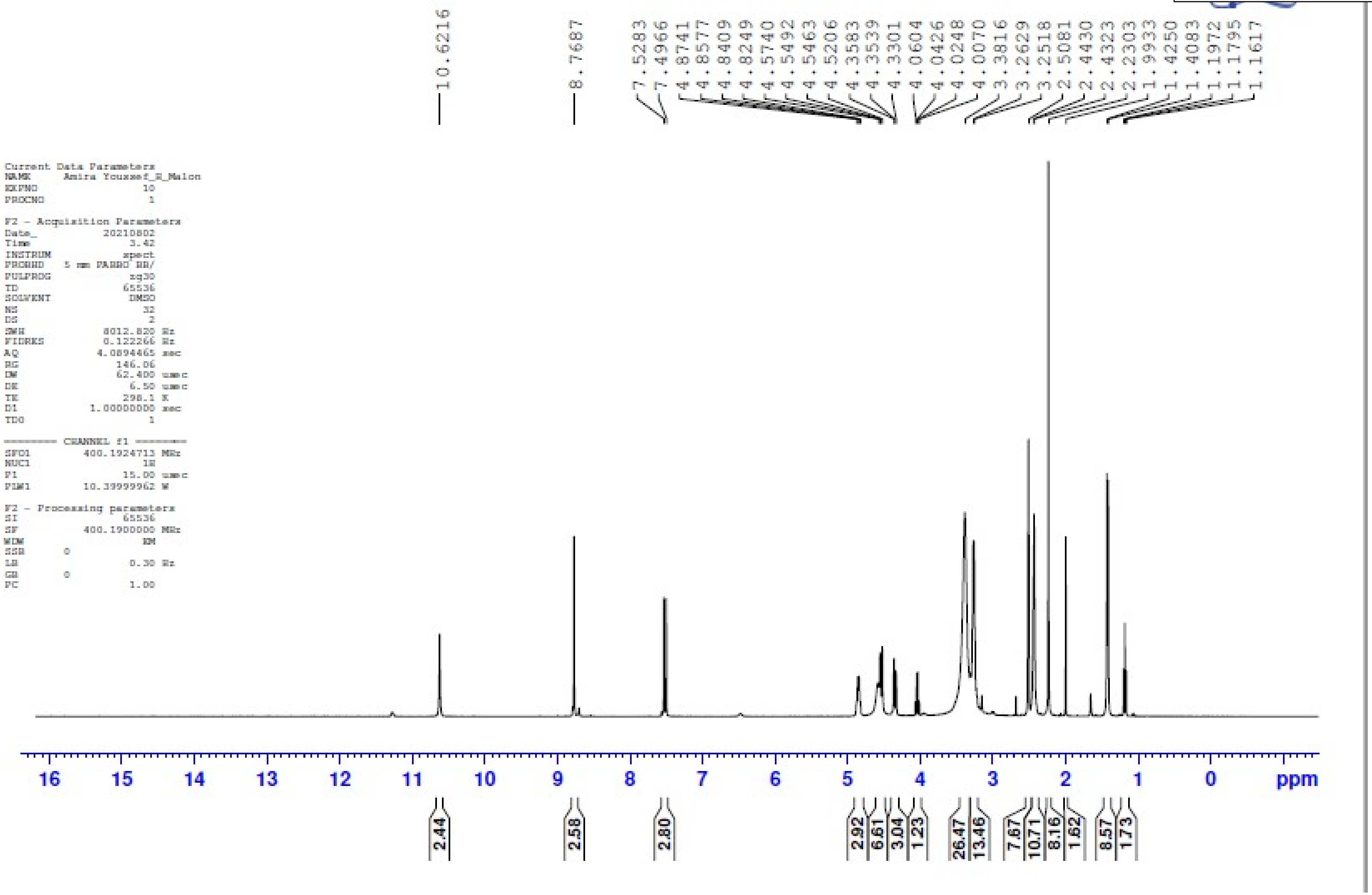

Amira Youssef_H_malon_D2O

Microanalytical Unit - FOPCU - NMR laboratory
www.pharma.cu.edu.eg dir-mau.fopcu@pharma.cu.edu.eg

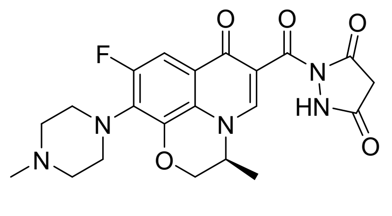

8 D_2O

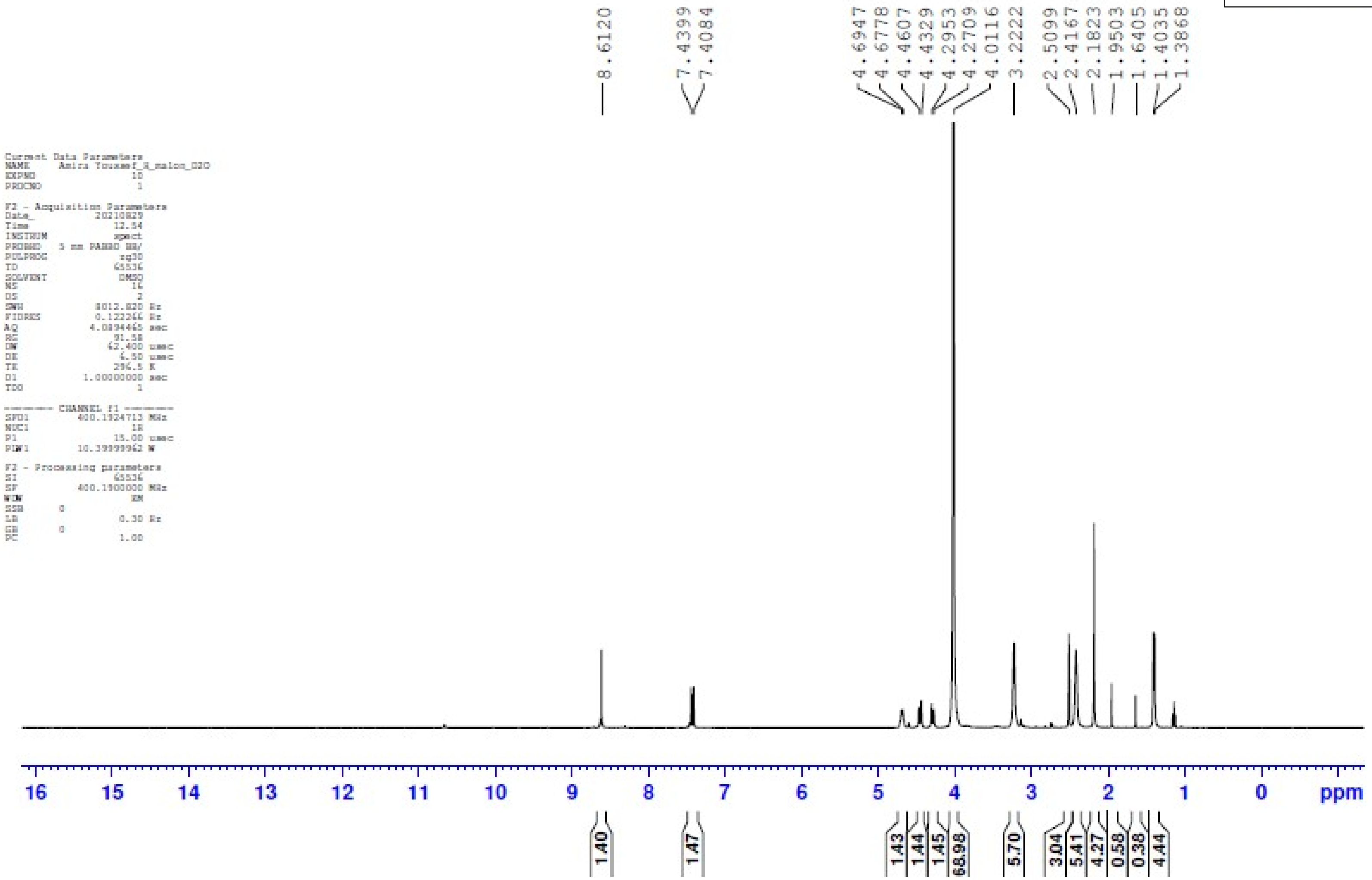

Amira Youssef_C_malon

Microanalytical Unit - FOPCU - NMR laboratory
www.pharma.cu.edu.eg dir-mau.fopcu@pharma.cu.edu.eg

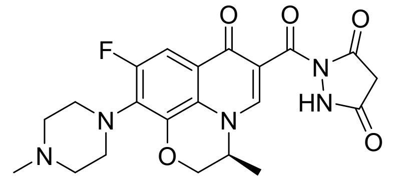

8 ^{13}C

```
Current Data Parameters
NAME     Amira Youssef_C_malon
EXPNO                  10
PROCNO                  1

F2 - Acquisition Parameters
Date_            20210826
Time                20.17
INSTRUM             spect
PROBHD   5 mm PABBO BB/
PULPROG           zgpg30
TD                  65536
SOLVENT              DMSO
NS                   1500
DS                      4
SWH             24038.461 Hz
FIDRES           0.366798 Hz
AQ              1.3631488 sec
RG                 202.37
DW                 20.800 usec
DE                   6.50 usec
TE                  298.0 K
D1             2.00000000 sec
D11            0.03000000 sec
TD0                     1

======== CHANNEL f1 ========
SFO1         100.6379178 MHz
NUC1                 13C
P1                  10.00 usec
PLW1          45.00000000 W

======== CHANNEL f2 ========
SFO2         400.1916008 MHz
NUC2                  1H
CPDPRG[2            waltz16
```

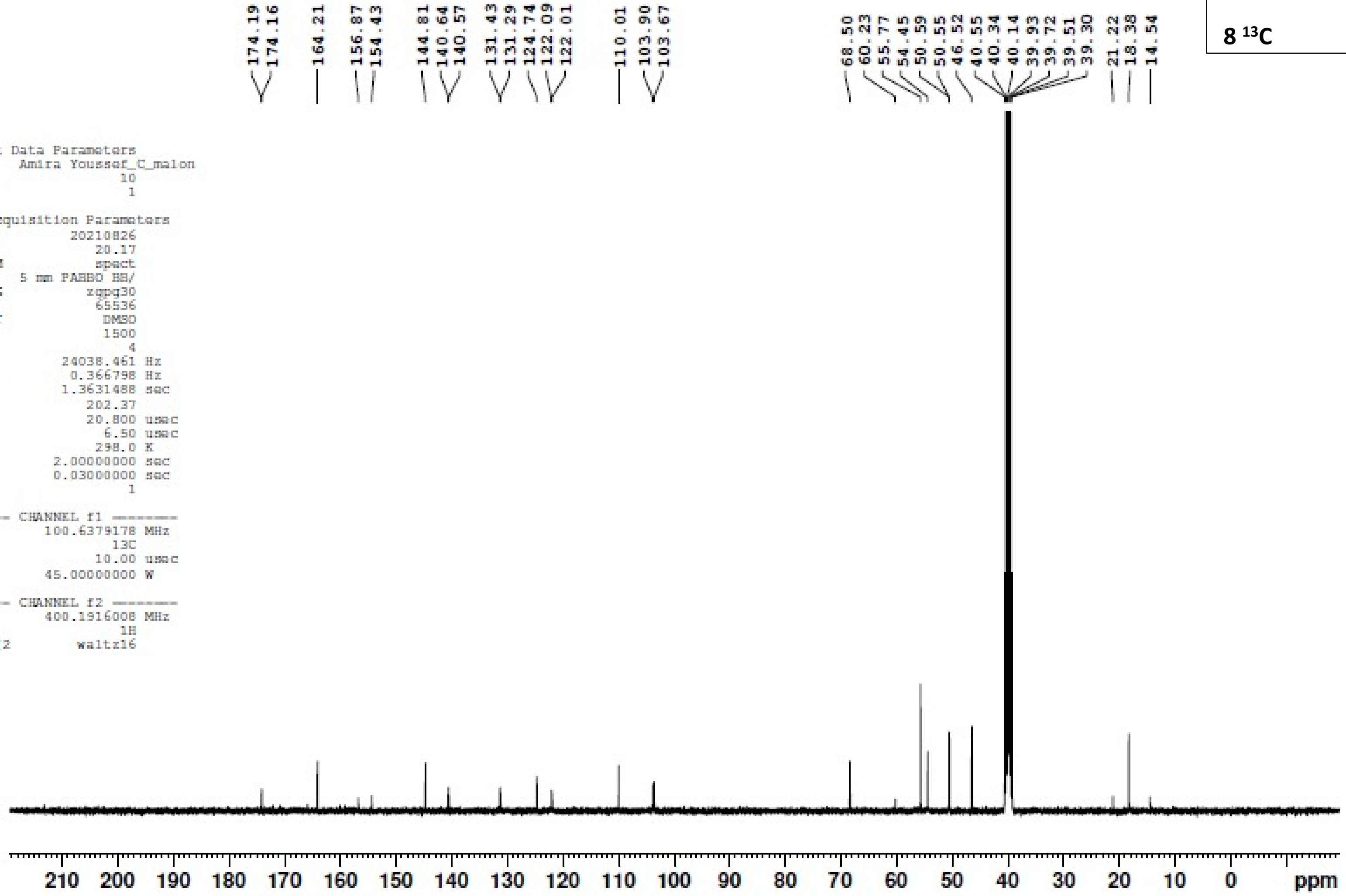

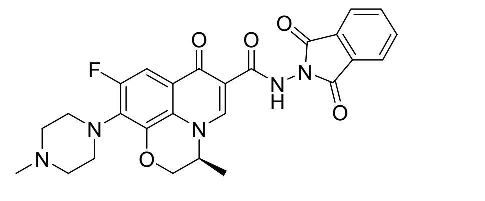

F
N
O
N
O
O
N
H
N
O
O

9 IR

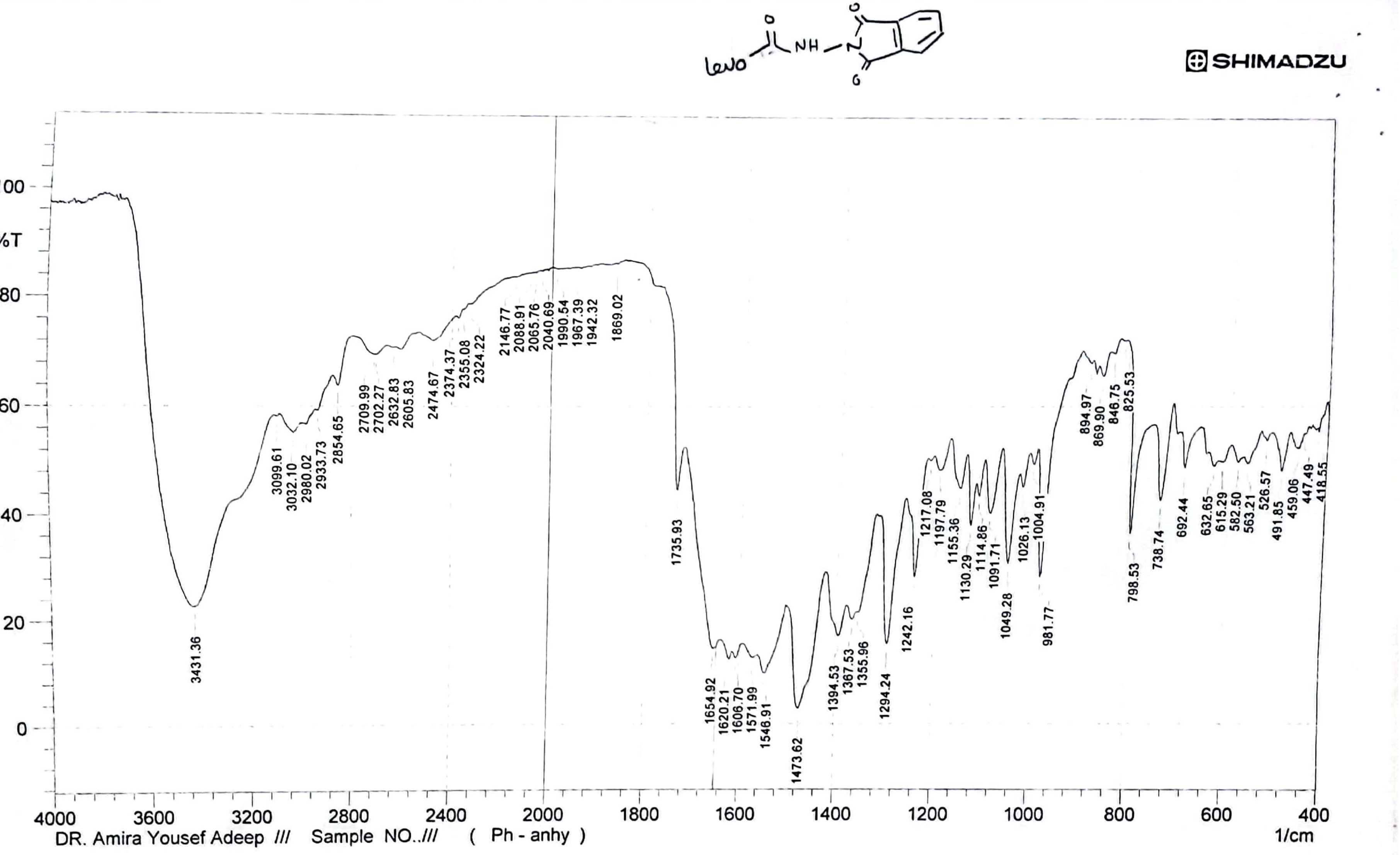

%T
100
80
60
40
20
0
3431.36
3099.61
3032.10
2980.02
2933.73
2854.65
2709.99
2702.27
2632.83
2605.83
2474.67
2374.37
2355.08
2324.22
2146.77
2088.91
2065.76
2040.69
1990.54
1967.39
1942.32
1869.02
1735.93
1654.92
1620.21
1606.70
1571.99
1548.91
1473.62
1394.53
1367.53
1355.96
1294.24
1242.16
1217.08
1197.79
1155.36
1130.29
1114.86
1091.71
1049.28
1026.13
1004.91
981.77
894.97
869.90
846.75
825.53
798.53
738.74
692.44
632.65
615.29
582.50
563.21
526.57
491.85
459.06
447.49
418.55
4000
3600
3200
2800
2400
2000
1800
1600
1400
1200
1000
800
600
400
1/cm
DR. Amira Yousef Adeep /// Sample NO../// (Ph - anhy)

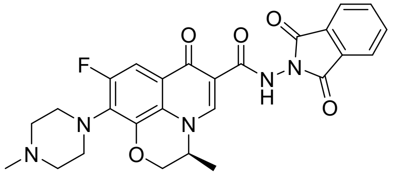

9 NMR

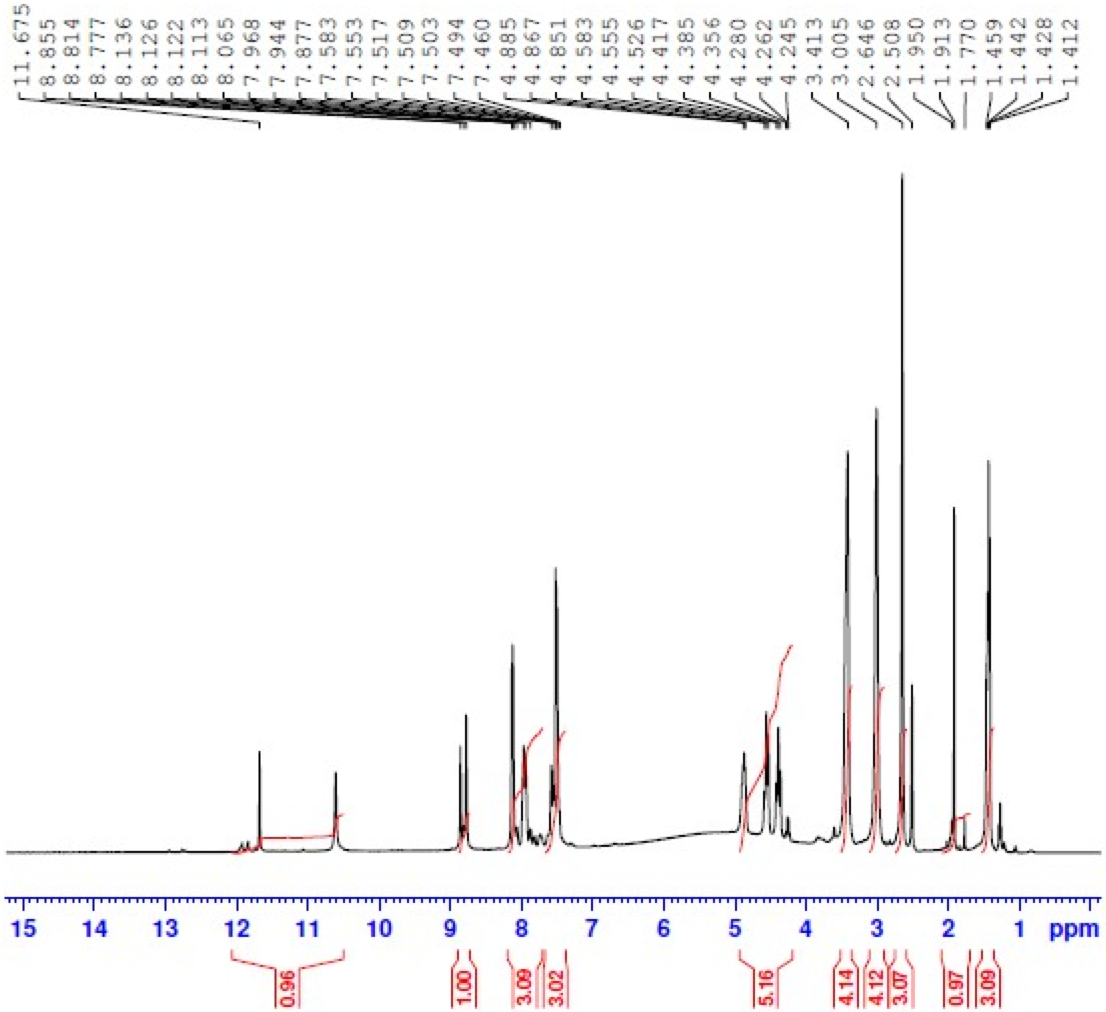


```
Current Data Parameters
NAME        amira-youssef-Ph-anh
EXPNO                  1
PROCNO                 1

F2 - Acquisition Parameters
Date_           20220426
Time               10.27
INSTRUM            spect
PROBHD    5 mm PABBO BB/
PULPROG             zg30
TD                 65536
SOLVENT             DMSO
NS                    52
DS                     2
SWH             8012.820 Hz
FIDRES          0.122266 Hz
AQ             4.0894465 sec
RG                205.37
DW                62.400 usec
DE                  6.50 usec
TE                 300.0 K
D1            1.00000000 sec
TD0                    1

======== CHANNEL f1 ========
SFO1         400.1524711 MHz
NUC1                  1H
P1                 12.00 usec
PLW1         18.00000000 W

F2 - Processing parameters
SI                 65536
SF           400.1500000 MHz
WDW                   EM
SSB      0
LB                  0.30 Hz
GB       0
PC                  1.00
```

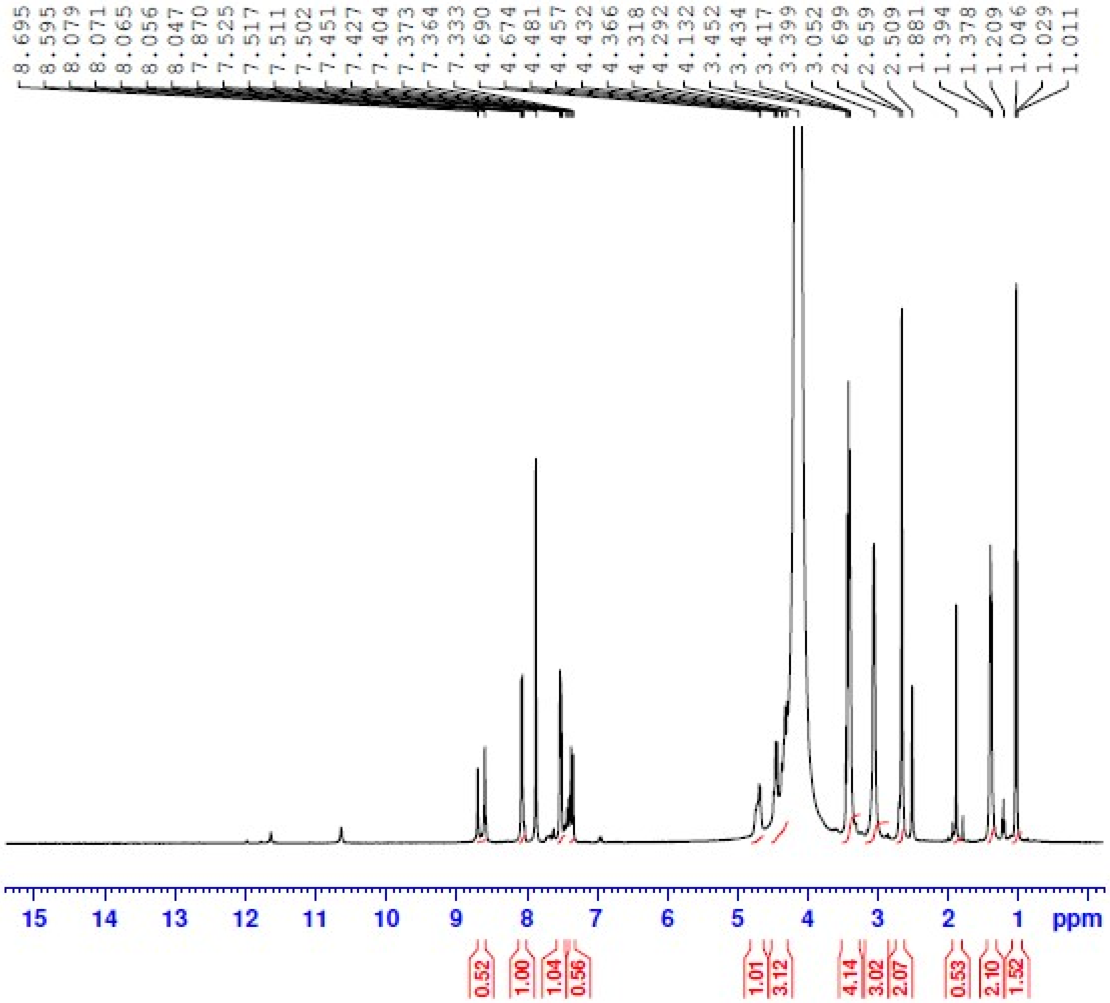

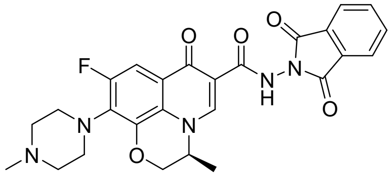

9 D_2O

```
Current Data Parameters
NAME      amira-youssef-Ph-anh-d2o
EXPNO                    1
PROCNO                   1

F2 - Acquisition Parameters
Date_             20220606
Time                  8.02
INSTRUM              spect
PROBHD   5 mm PABBO BB/
PULPROG               zg30
TD                   65536
SOLVENT               DMSO
NS                      41
DS                       2
SWH            8012.820 Hz
FIDRES         0.122266 Hz
AQ           4.0894465 sec
RG                  103.61
DW              62.400 usec
DE                6.50 usec
TE                 300.0 K
D1          1.00000000 sec
TD0                      1

======= CHANNEL f1 =======
SFO1       400.1524711 MHz
NUC1                    1H
P1               12.00 usec
PLW1       18.00000000 W

F2 - Processing parameters
SI                   65536
SF         400.1500000 MHz
WDW                     EM
SSB      0
LB                0.30 Hz
GB       0
PC                    1.00
```

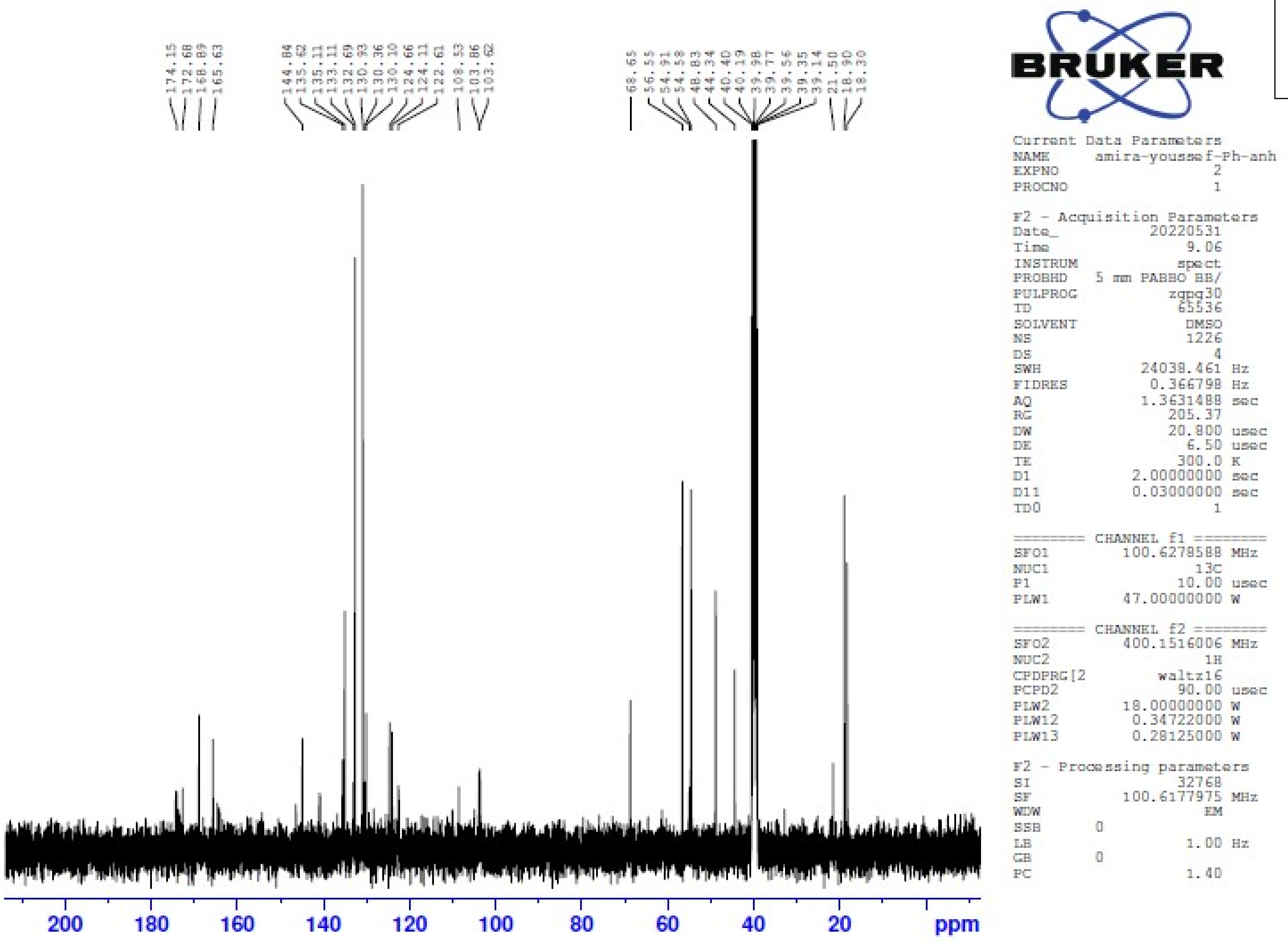

174.15
172.68
168.89
165.63
144.84
135.62
135.11
133.11
132.69
130.93
130.36
130.30
124.66
124.11
122.61
108.53
103.86
103.62
68.65
56.55
54.91
54.58
48.83
44.34
40.40
40.19
39.98
39.77
39.56
39.35
39.14
21.50
18.90
18.30
200 180 160 140 120 100 80 60 40 20 ppm
BRUKER
Current Data Parameters
NAME amira-youssef-Ph-anh
EXPNO 2
PROCNO 1
F2 - Acquisition Parameters
Date_ 20220531
Time 9.06
INSTRUM spect
PROBHD 5 mm PABBO BB/
PULPROG zgpg30
TD 65536
SOLVENT DMSO
NS 1226
DS 4
SWH 24038.461 Hz
FIDRES 0.366798 Hz
AQ 1.3631488 sec
RG 205.37
DW 20.800 usec
DE 6.50 usec
TE 300.0 K
D1 2.00000000 sec
D11 0.03000000 sec
TD0 1
======== CHANNEL f1 ========
SFO1 100.6278588 MHz
NUC1 13C
P1 10.00 usec
PLW1 47.00000000 W
======== CHANNEL f2 ========
SFO2 400.1516006 MHz
NUC2 1H
CPDPRG[2 waltz16
PCPD2 90.00 usec
PLW2 18.00000000 W
PLW12 0.34722000 W
PLW13 0.28125000 W
F2 - Processing parameters
SI 32768
SF 100.6177975 MHz
WDW EM
SSB 0
LB 1.00 Hz
GB 0
PC 1.40

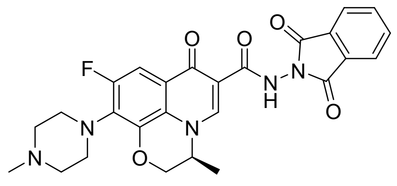

F
O
O
O
O
N
N
N
H
N
N
O

9 ^{13}C

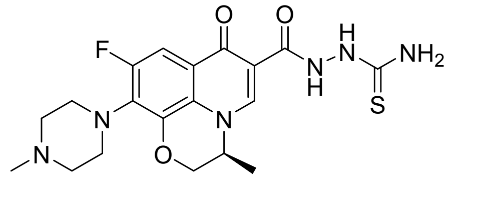

10 IR

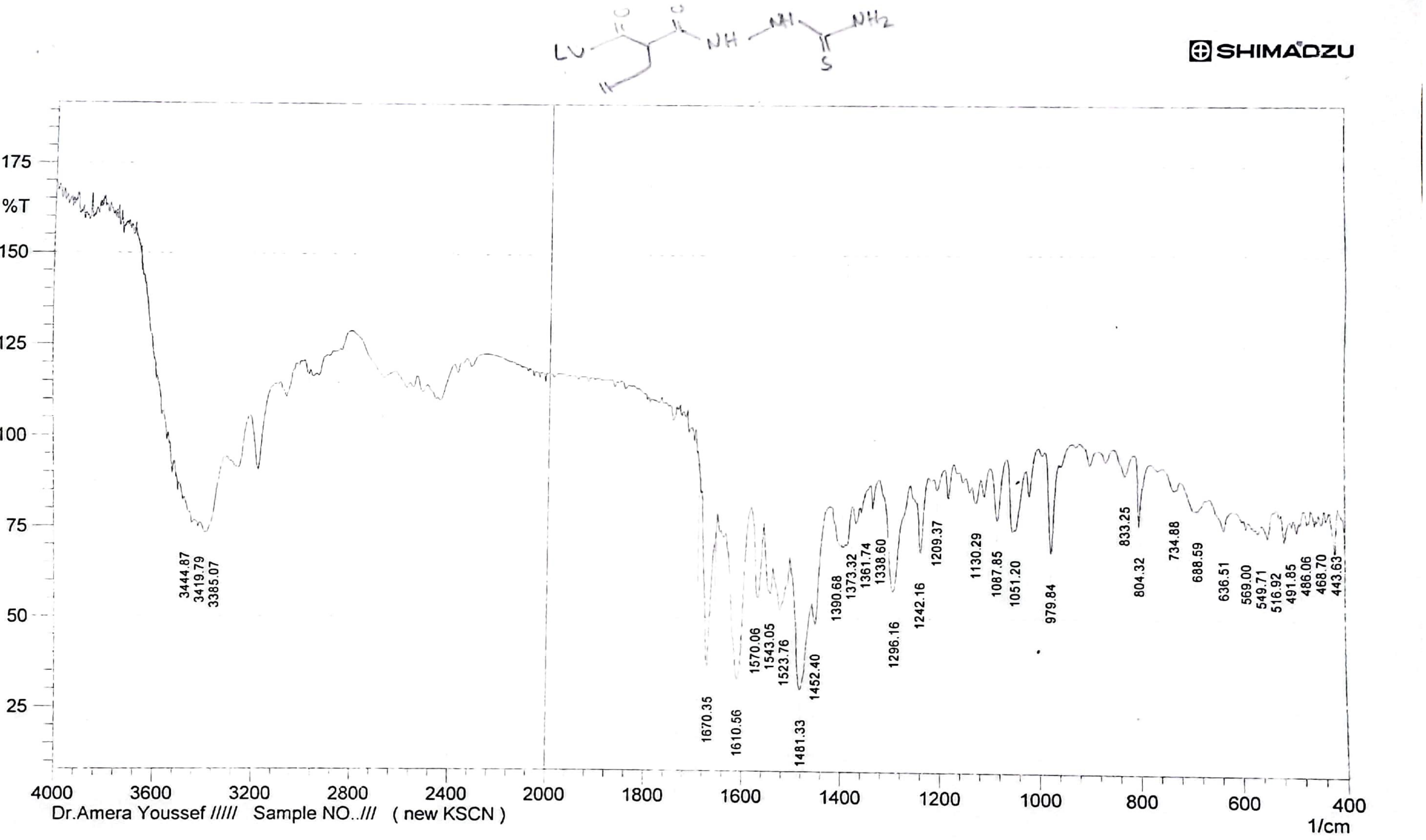

mment: Date/Time: 4/30/2010 4:19:57 AM

Amira Youssef_H_new-KSCN

Microanalytical Unit - FOPCU - NMR laboratory
www.pharma.cu.edu.eg dir-mau.fopcu@pharma.cu.edu.eg

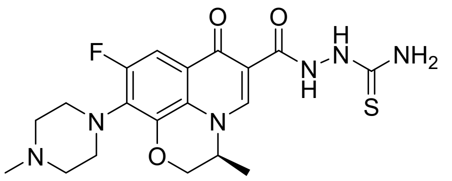

10 NMR

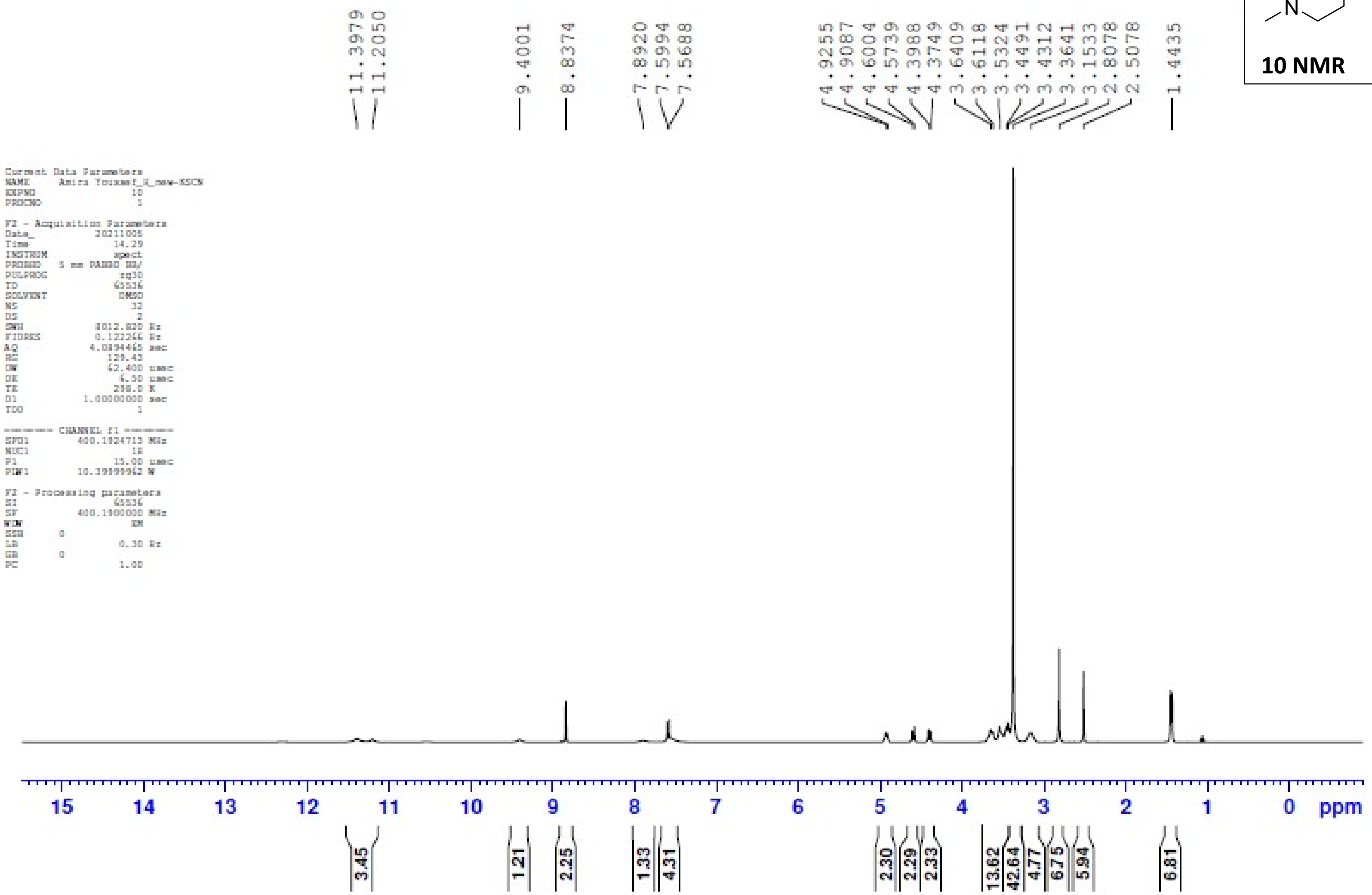

Amira Youssef_H_newKSCN_D2O

Microanalytical Unit - FOPCU - NMR laboratory
www.pharma.cu.edu.eg dir-mau.fopcu@pharma.cu.edu.eg

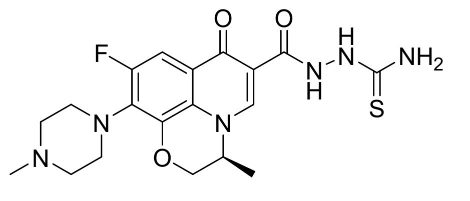

10 D_2O

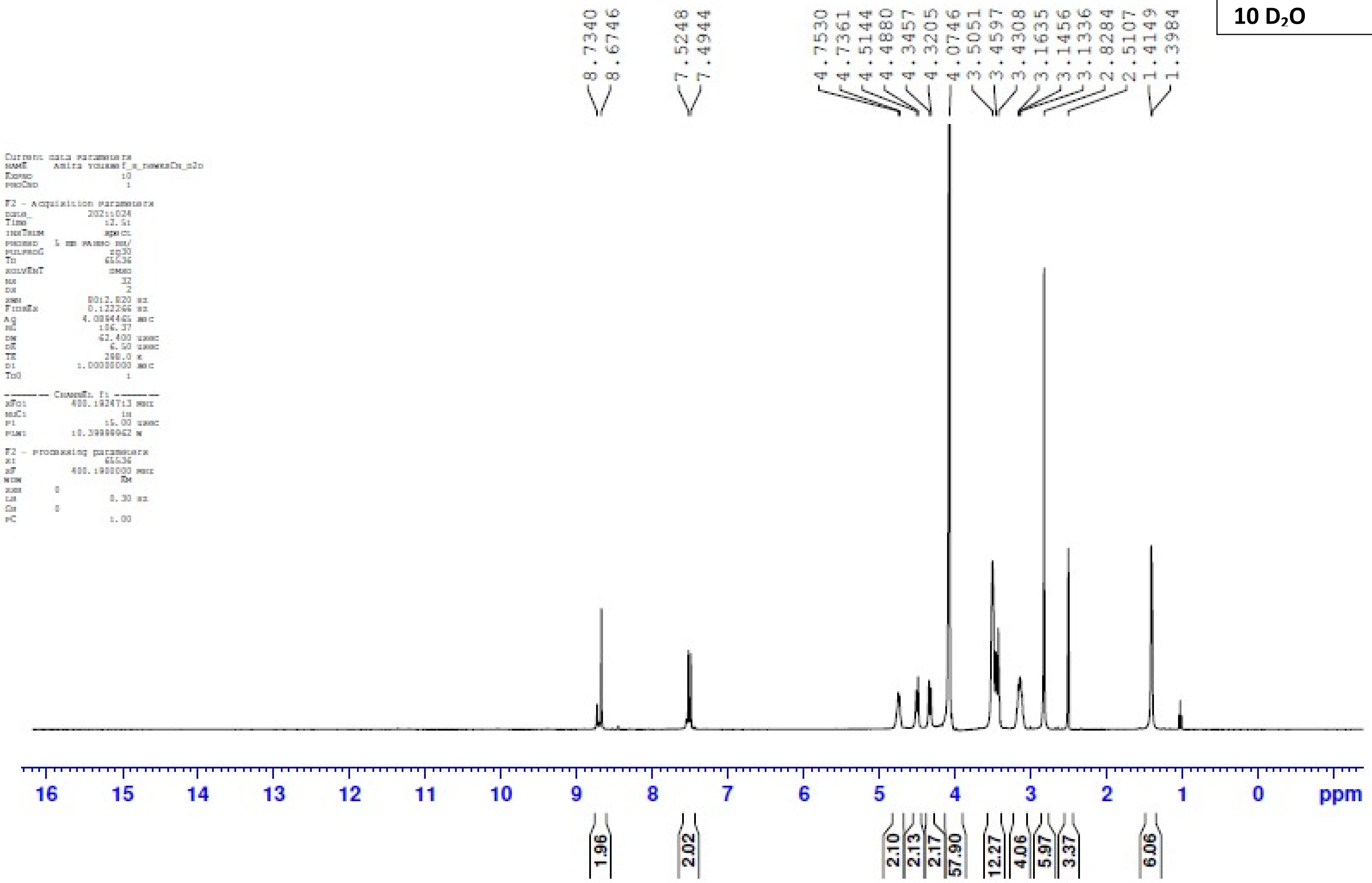

Amira Youssef_C_newKSCN

Microanalytical Unit - FOPCU - NMR laboratory
www.pharma.cu.edu.eg dir-mau.fopcu@pharma.cu.edu.eg

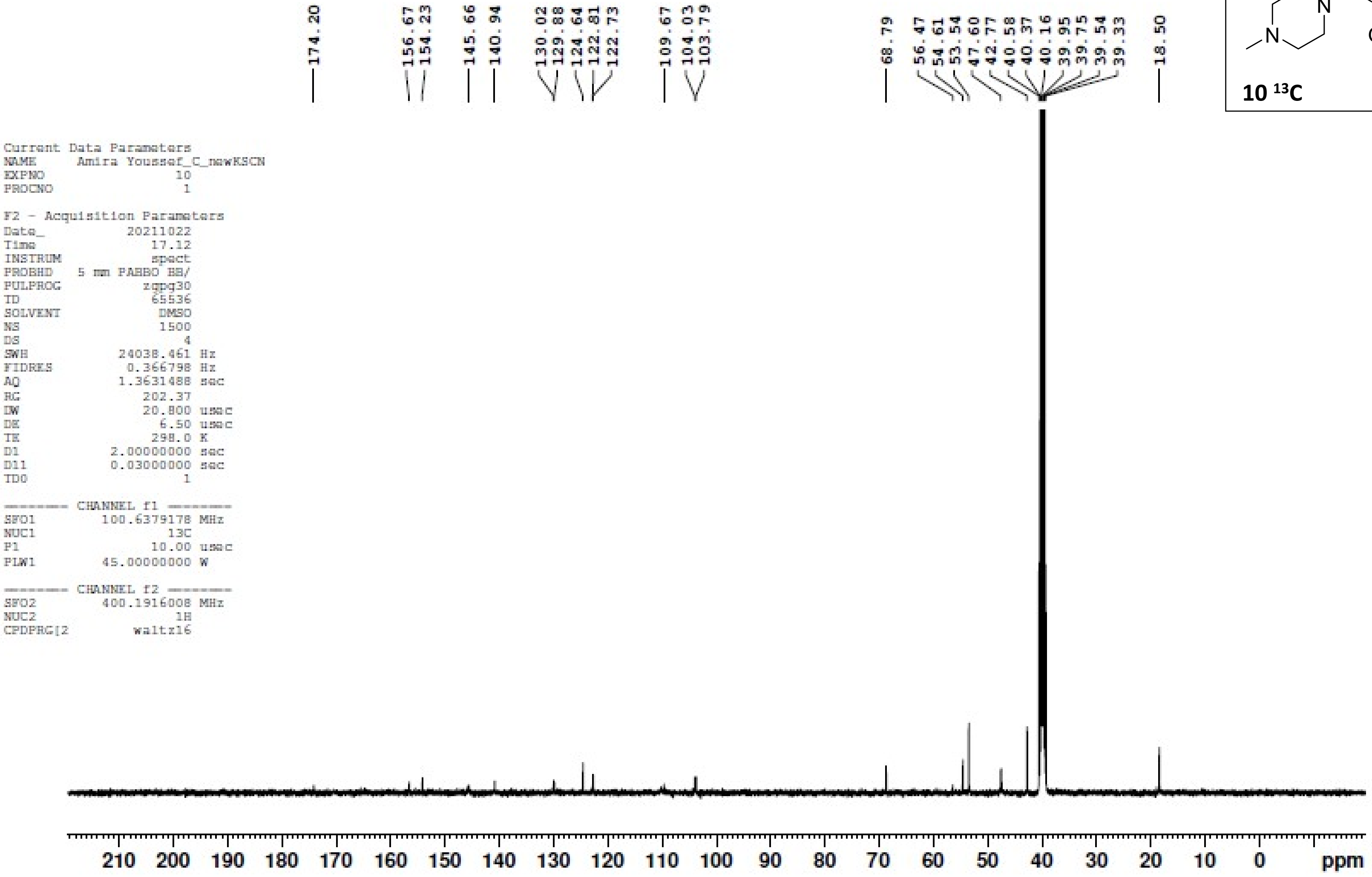

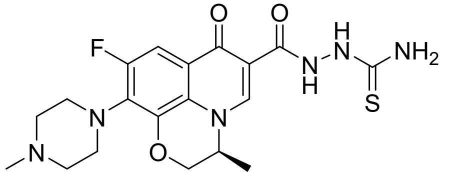

10 ^{13}C

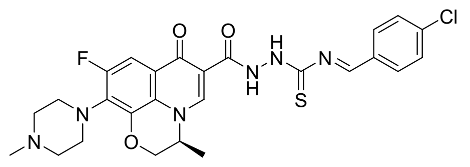

11a IR

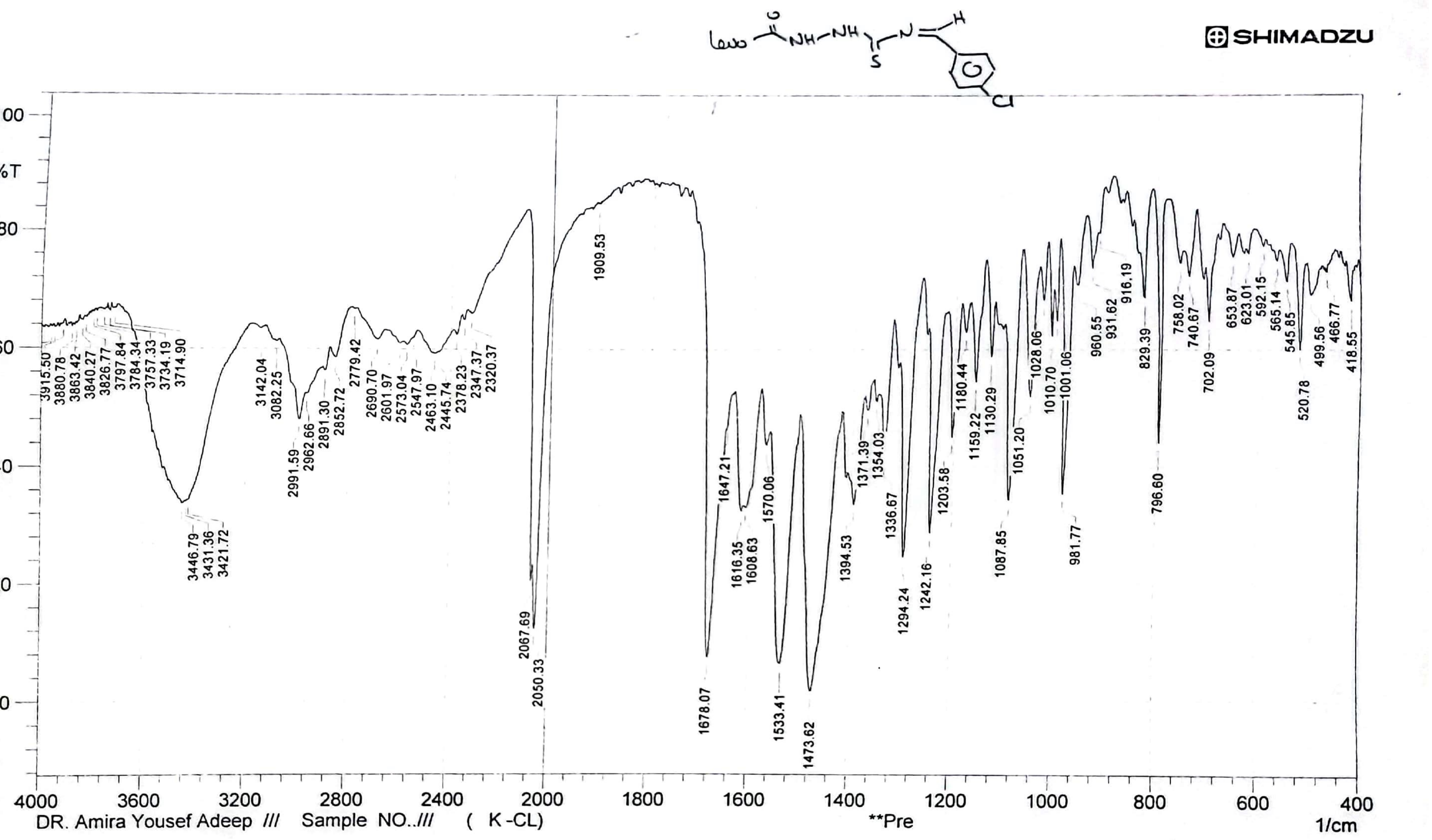
SHIMADZU
%T
3915.50
3880.78
3863.42
3840.27
3826.77
3797.84
3784.34
3757.33
3734.19
3714.90
3446.79
3431.36
3421.72
3142.04
3082.25
2991.59
2962.66
2891.30
2852.72
2779.42
2690.70
2601.97
2573.04
2547.97
2463.10
2445.74
2378.23
2347.37
2320.37
2067.69
2050.33
1909.53
1678.07
1647.21
1616.35
1608.63
1570.06
1533.41
1473.62
1394.53
1371.39
1354.03
1336.67
1294.24
1242.16
1203.58
1180.44
1159.22
1130.29
1087.85
1051.20
1028.06
1010.70
1001.06
981.77
960.55
931.62
916.19
829.39
796.60
758.02
740.67
702.09
653.87
623.01
592.15
565.14
545.85
520.78
499.56
466.77
418.55
4000 3600 3200 2800 2400 2000 1800 1600 1400 1200 1000 800 600 400
1/cm
DR. Amira Yousef Adeep /// Sample NO../// (K -CL)
**Pre

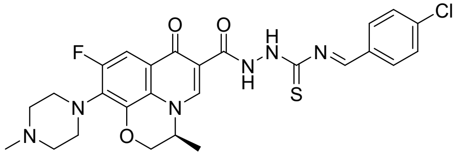

11a NMR

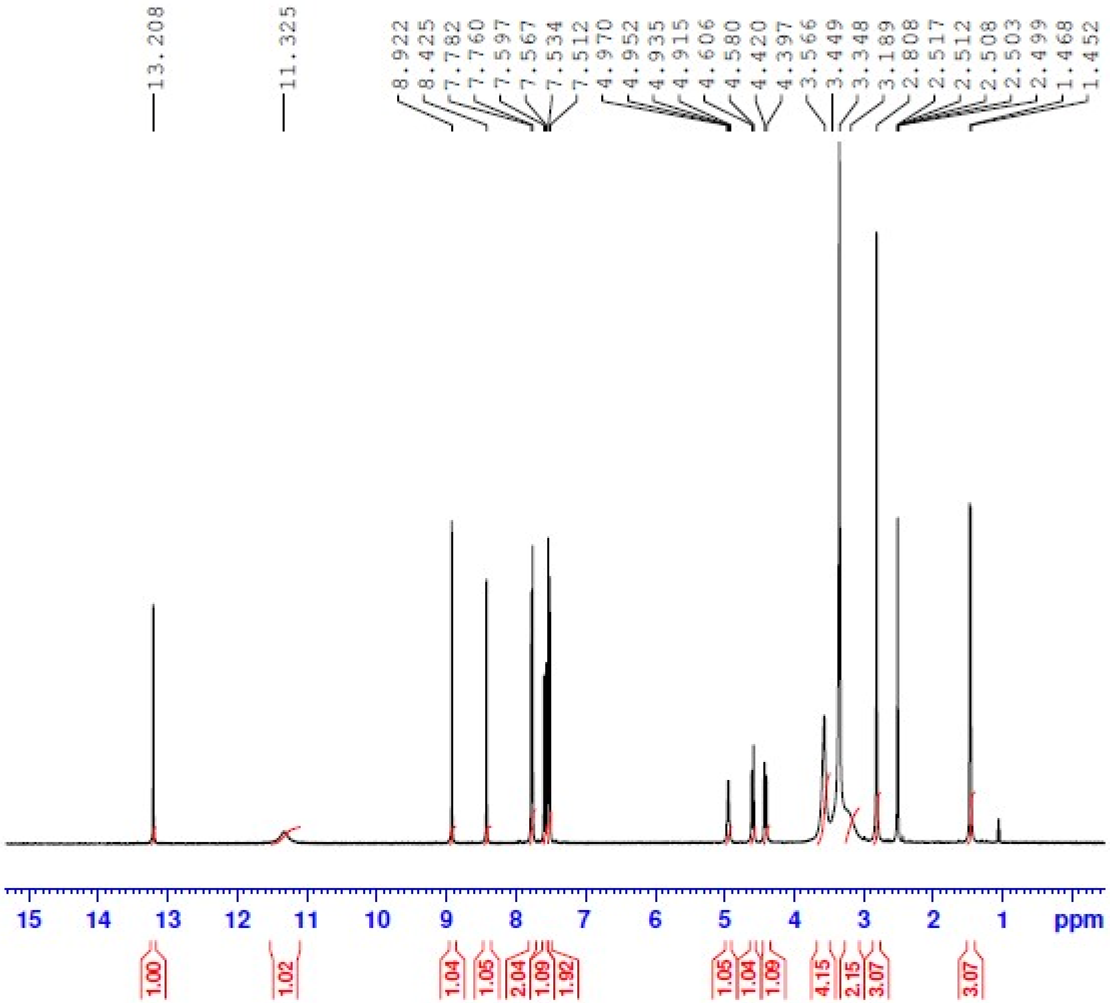


```
Current Data Parameters
NAME      amira-youssef-KCl
EXPNO                     1
PROCNO                    1

F2 - Acquisition Parameters
Date_              20220426
Time                  10.21
INSTRUM               spect
PROBHD   5 mm PABBO BB/
PULPROG                zg30
TD                    65536
SOLVENT                DMSO
NS                       20
DS                        2
SWH               8012.820 Hz
FIDRES            0.122266 Hz
AQ               4.0894465 sec
RG                   205.37
DW                   62.400 usec
DE                     6.50 usec
TE                    300.0 K
D1               1.00000000 sec
TD0                       1

======== CHANNEL f1 ========
SFO1         400.1524711 MHz
NUC1                     1H
P1                    12.00 usec
PLW1           18.00000000 W

F2 - Processing parameters
SI                    65536
SF           400.1500000 MHz
WDW                      EM
SSB      0
LB                     0.30 Hz
GB       0
PC                     1.00
```

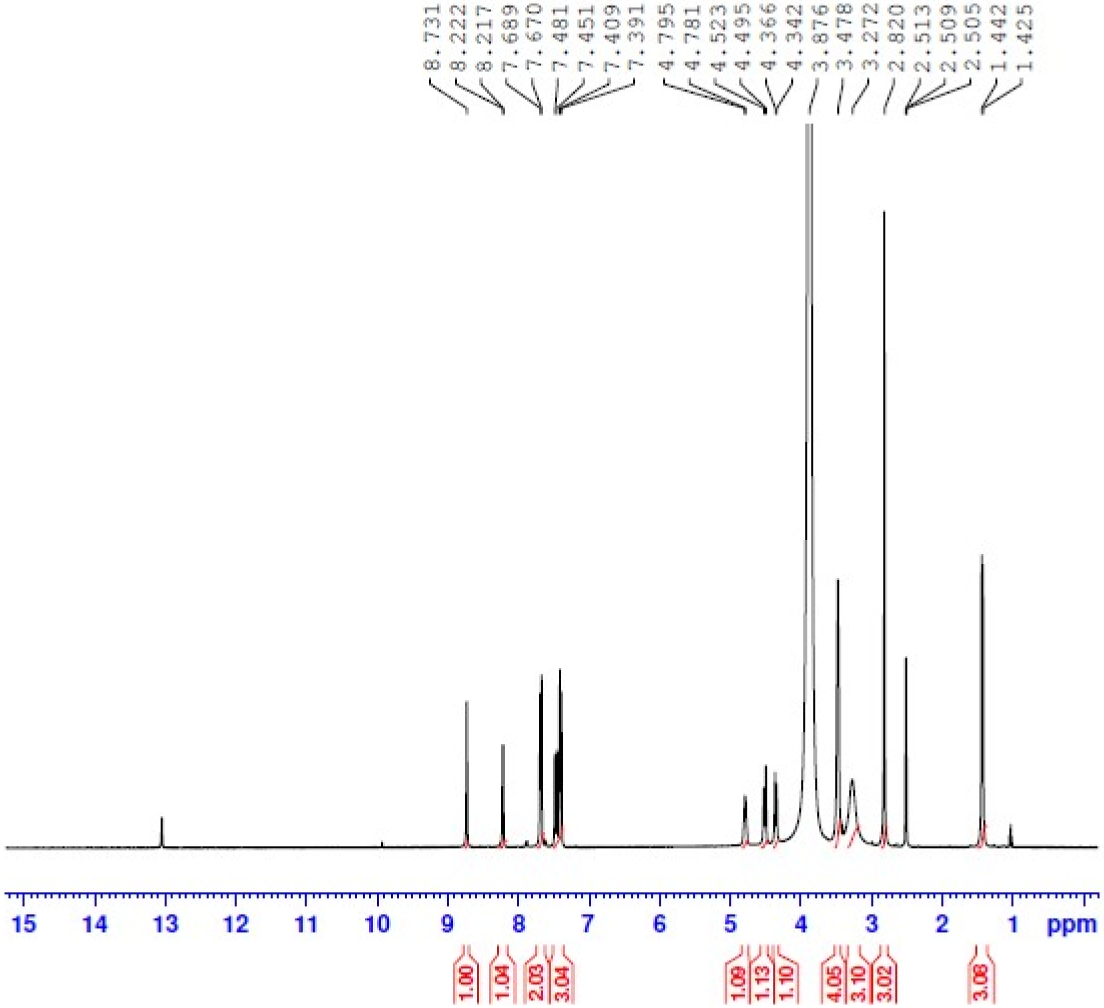


```
Current Data Parameters
NAME      amira-youssef-KC1-d2o
EXPNO                 1
PROCNO                1

F2 - Acquisition Parameters
Date_          20220606
Time               7.56
INSTRUM           spect
PROBHD   5 mm PABBO BB/
PULPROG            zg30
TD                65536
SOLVENT            DMSO
NS                   29
DS                    2
SWH            8012.820 Hz
FIDRES         0.122266 Hz
AQ            4.0894465 sec
RG               164.32
DW               62.400 usec
DE                 6.50 usec
TE                300.0 K
D1           1.00000000 sec
TD0                   1

======== CHANNEL f1 ========
SFO1        400.1524711 MHz
NUC1                 1H
P1                12.00 usec
PLW1        18.00000000 W

F2 - Processing parameters
SI                65536
SF          400.1500000 MHz
WDW                  EM
SSB      0
LB                 0.30 Hz
GB       0
PC                 1.00
```

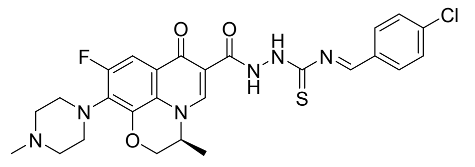

11a D_2O

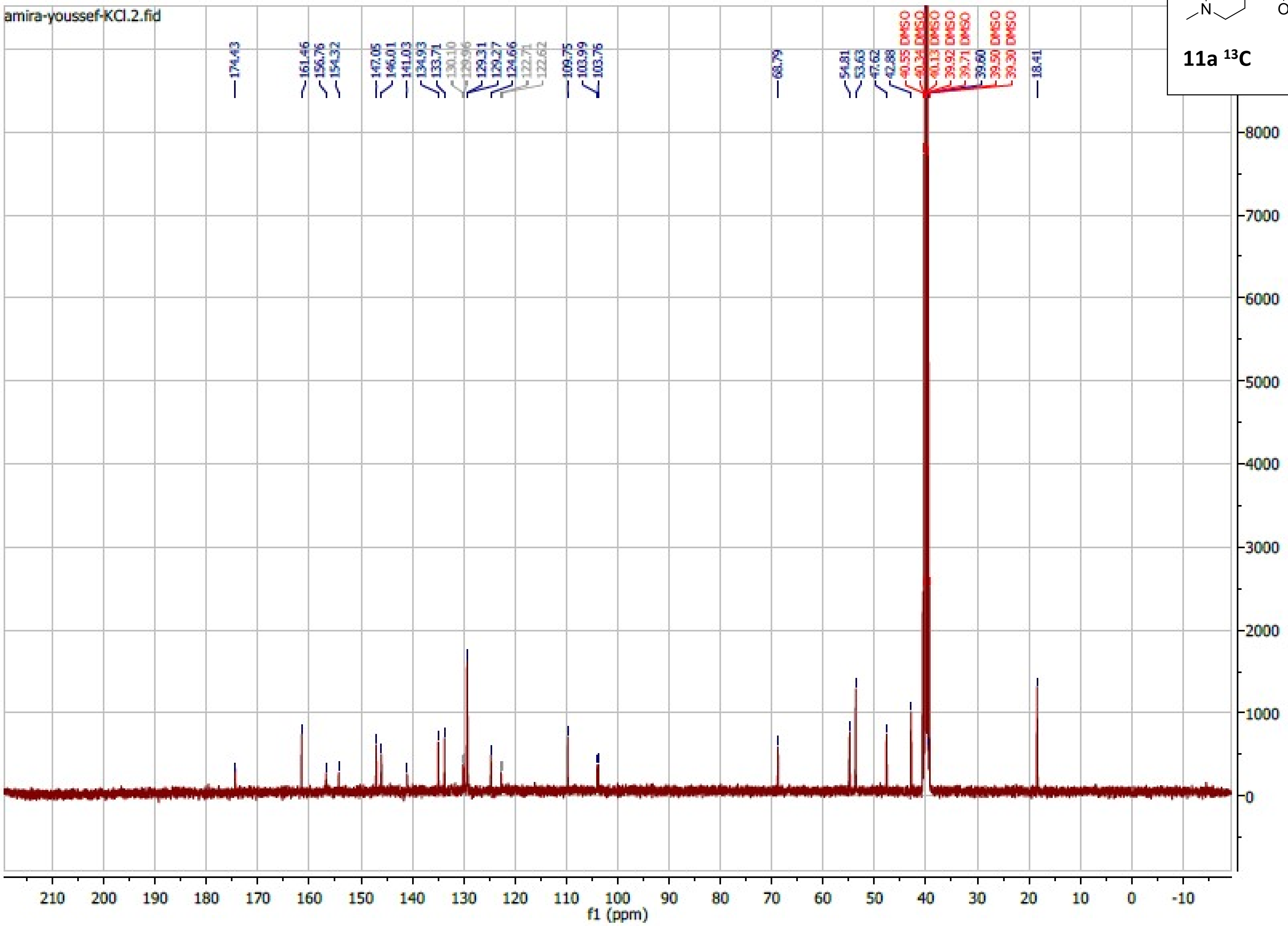

amira-youssef-KCl.2.fid
174.43
161.46
156.76
154.32
147.05
146.01
141.03
134.93
133.71
130.10
129.96
129.31
129.27
124.66
122.71
122.62
109.75
103.99
103.76
68.79
54.81
53.63
47.62
42.88
40.55 DMSO
40.34 DMSO
40.13 DMSO
39.92 DMSO
39.71 DMSO
39.60
39.50 DMSO
39.30 DMSO
18.41
f1 (ppm)

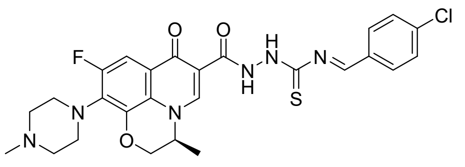

F
O
O
H
N
N
H
N
S
Cl
N
N
O
N

11a ^{13}C

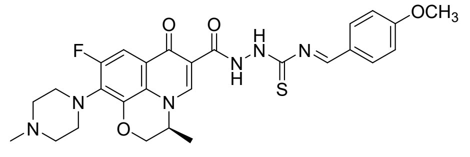

11b IR

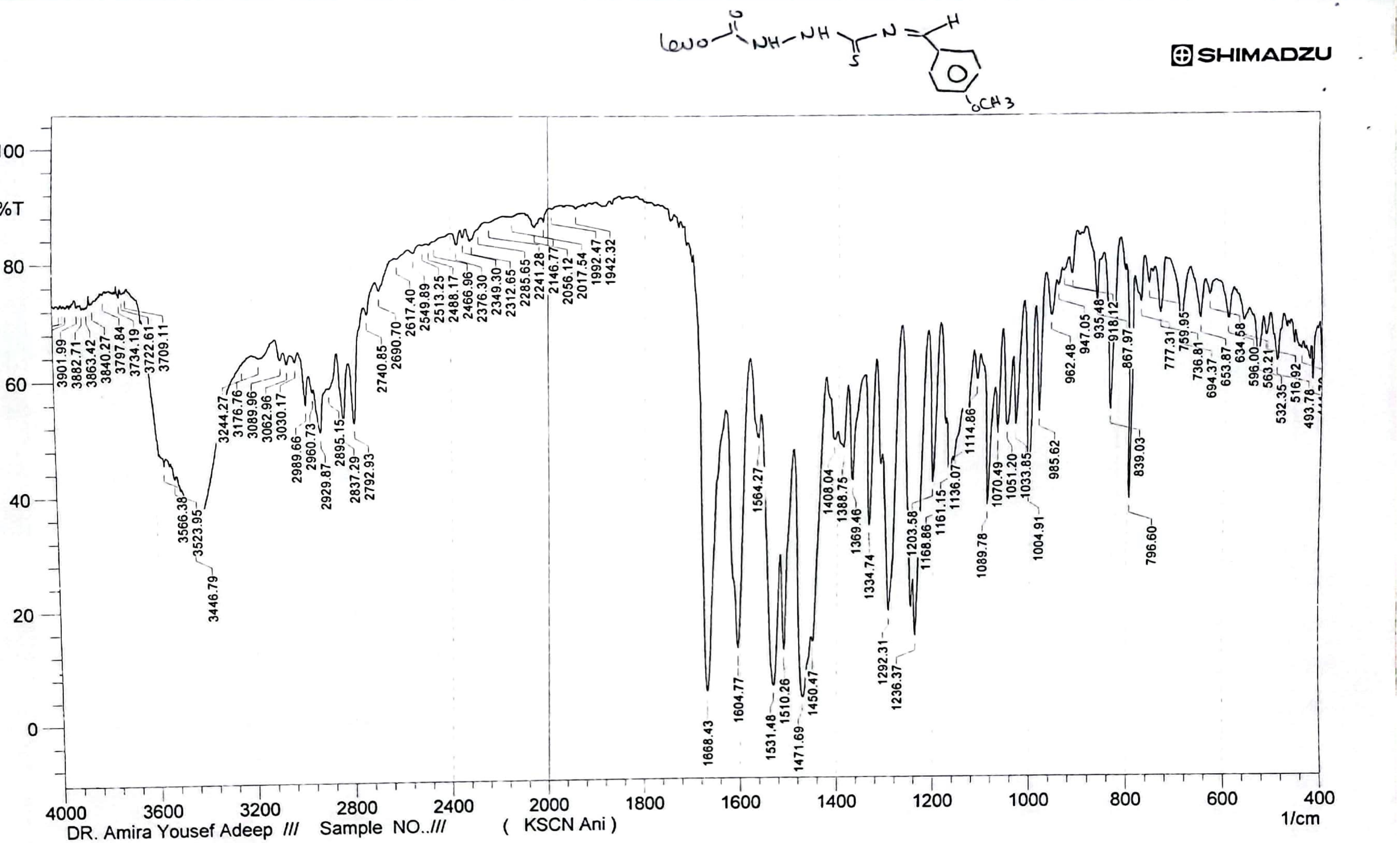

DR. Amira Yousef Adeep /// Sample NO../// (KSCN Ani)

Comment; Date/Time; 3/14/2022 12:40:49 PM

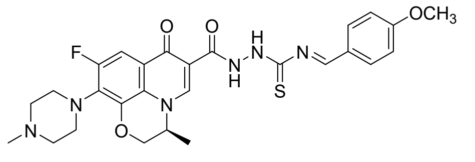

11b NMR

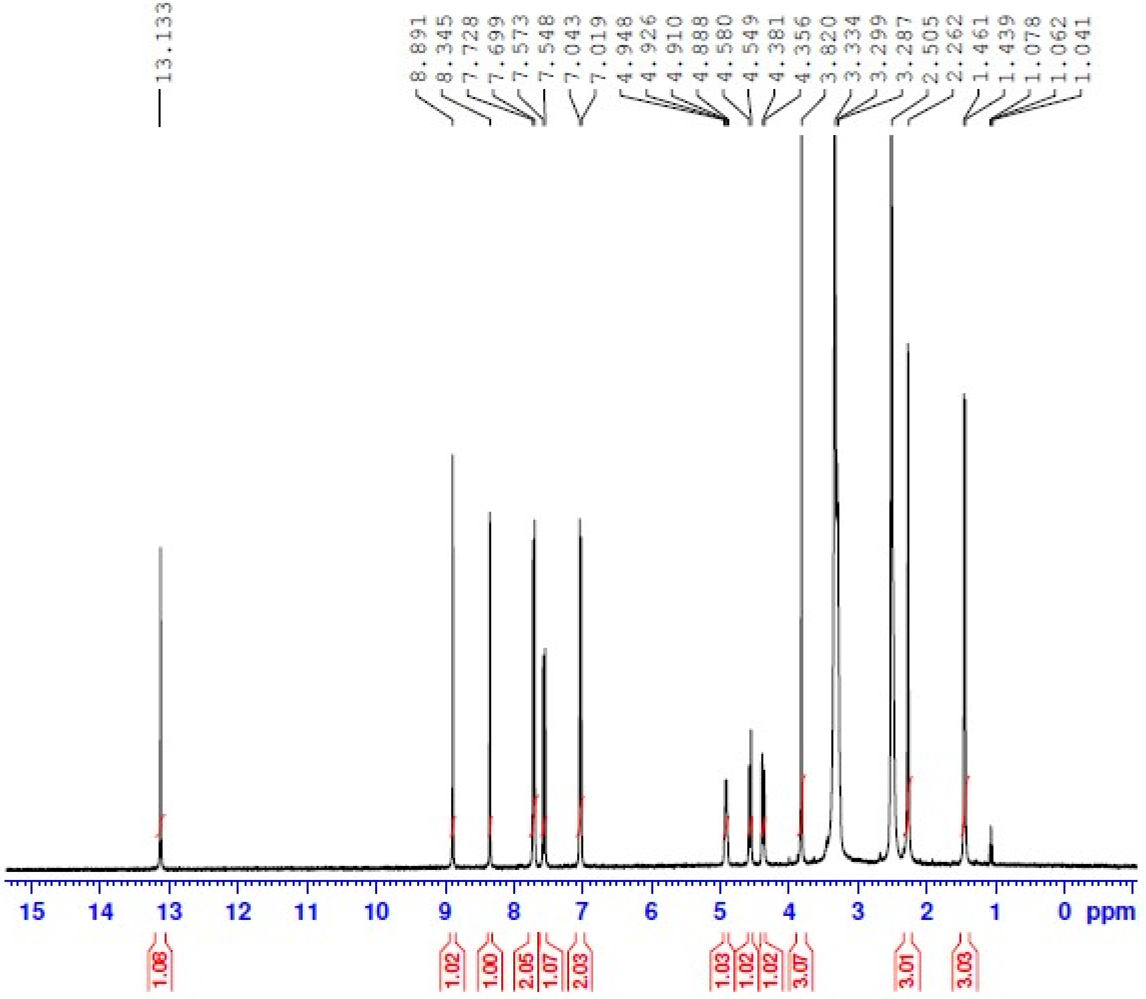

Current Data Parameters
NAME abdelrahman-ziada-K-Ani
EXPNO 1
PROCNO 1

F2 - Acquisition Parameters
Date_ 20220228
Time 14.29
INSTRUM spect
PROBHD 5 mm PABBO BB/
PULPROG zg30
TD 65536
SOLVENT DMSO
NS 60
DS 2
SWH 8012.820 Hz
FIDRES 0.122266 Hz
AQ 4.0894465 sec
RG 205.37
DW 62.400 usec
DE 6.50 usec
TE 300.0 K
D1 1.00000000 sec
TD0 1

======== CHANNEL f1 ========
SFO1 400.1524711 MHz
NUC1 1H
P1 12.00 usec
PLW1 18.00000000 W

F2 - Processing parameters
SI 65536
SF 400.1500000 MHz
WDW EM
SSB 0
LB 0.30 Hz
GB 0
PC 1.00

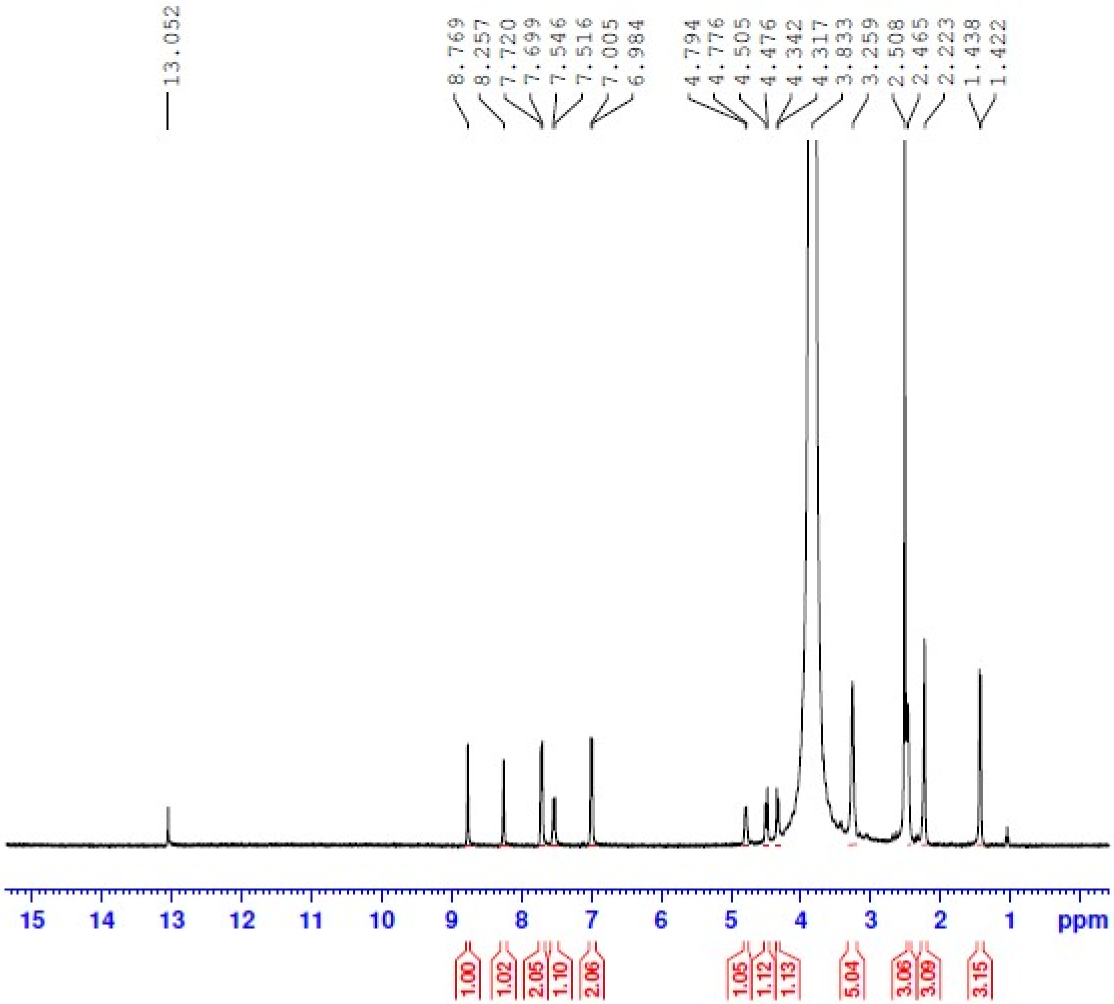


```
Current Data Parameters
NAME      amira-youssef-K-Ani-d2o
EXPNO                    1
PROCNO                   1

F2 - Acquisition Parameters
Date_             20220324
Time                  9.08
INSTRUM              spect
PROBHD   5 mm PABBO BB/
PULPROG               zg30
TD                   65536
SOLVENT               DMSO
NS                      68
DS                       2
SWH              8012.820 Hz
FIDRES           0.122266 Hz
AQ              4.0894465 sec
RG                  126.31
DW                  62.400 usec
DE                    6.50 usec
TE                   300.0 K
D1              1.00000000 sec
TD0                      1

======== CHANNEL f1 ========
SFO1         400.1524711 MHz
NUC1                    1H
P1                   12.00 usec
PLW1           18.00000000 W

F2 - Processing parameters
SI                   65536
SF           400.1500000 MHz
WDW                     EM
SSB      0
LB                    0.30 Hz
GB       0
PC                    1.00
```

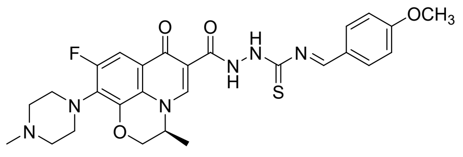

11b D_2O

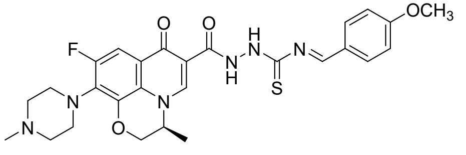

11b ^{13}C

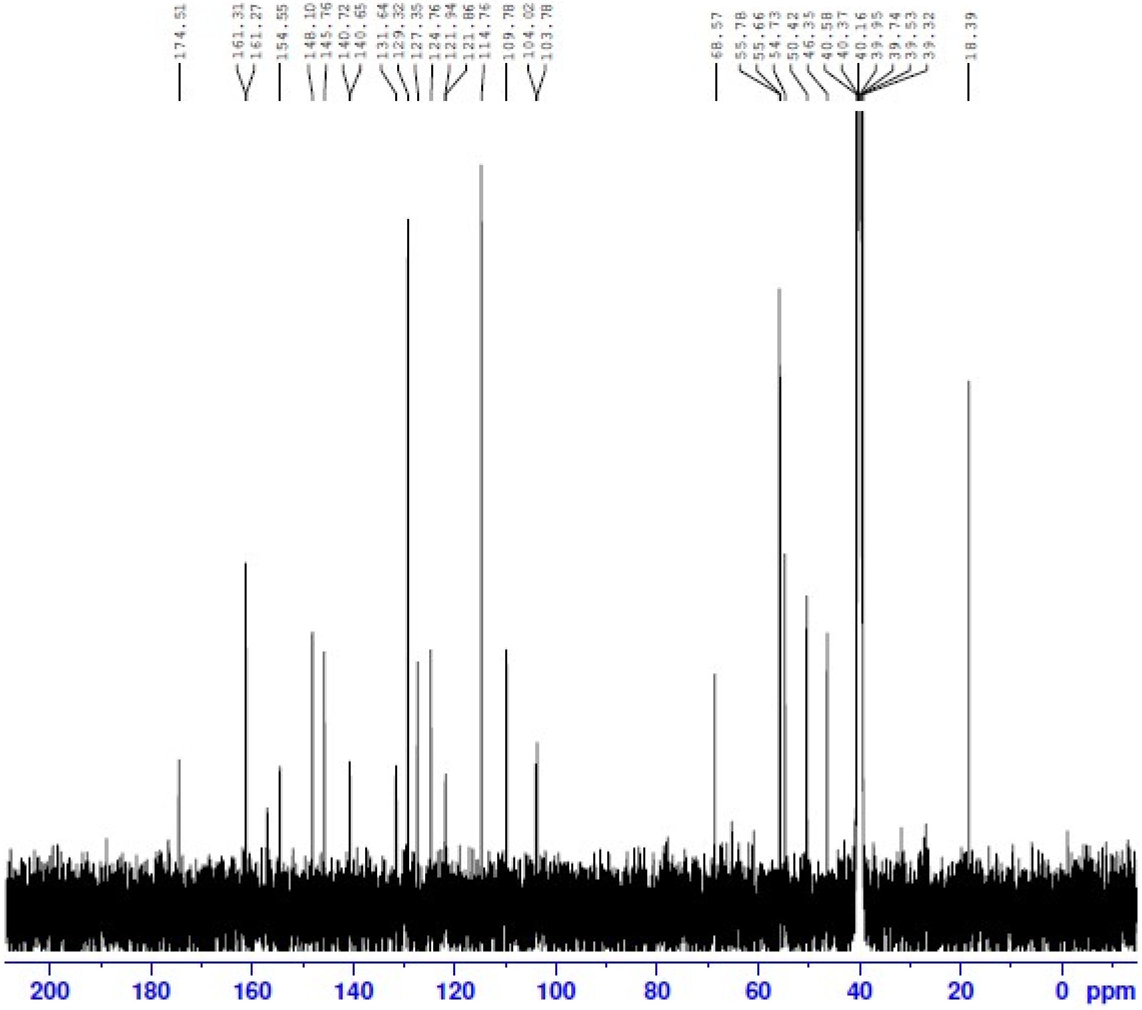

Current Data Parameters
NAME amira-youssef-kiani
EXPNO 2
PROCNO 1

F2 - Acquisition Parameters
Date_ 20220801
Time 7.54
INSTRUM spect
PROBHD 5 mm PABBO BB/
PULPROG zgpg30
TD 65536
SOLVENT DMSO
NS 14823
DS 4
SWH 24038.461 Hz
FIDRES 0.366798 Hz
AQ 1.3631488 sec
RG 205.37
DW 20.800 usec
DE 6.50 usec
TE 300.0 K
D1 2.00000000 sec
D11 0.03000000 sec
TD0 1

======== CHANNEL f1 ========
SFO1 100.6278588 MHz
NUC1 13C
P1 10.00 usec
PLW1 47.00000000 W

======== CHANNEL f2 ========
SFO2 400.1516006 MHz
NUC2 1H
CPDPRG[2 waltz16
PCPD2 90.00 usec
PLW2 18.00000000 W
PLW12 0.34722000 W
PLW13 0.28125000 W

F2 - Processing parameters
SI 32768
SF 100.6177975 MHz
WDW EM
SSB 0
LB 1.00 Hz
GB 0
PC 1.40

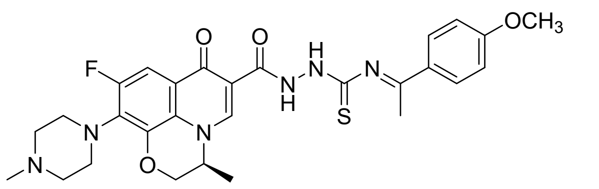

F
O
O
H
N
N
H
N
S
N
O
OCH3

12a IR

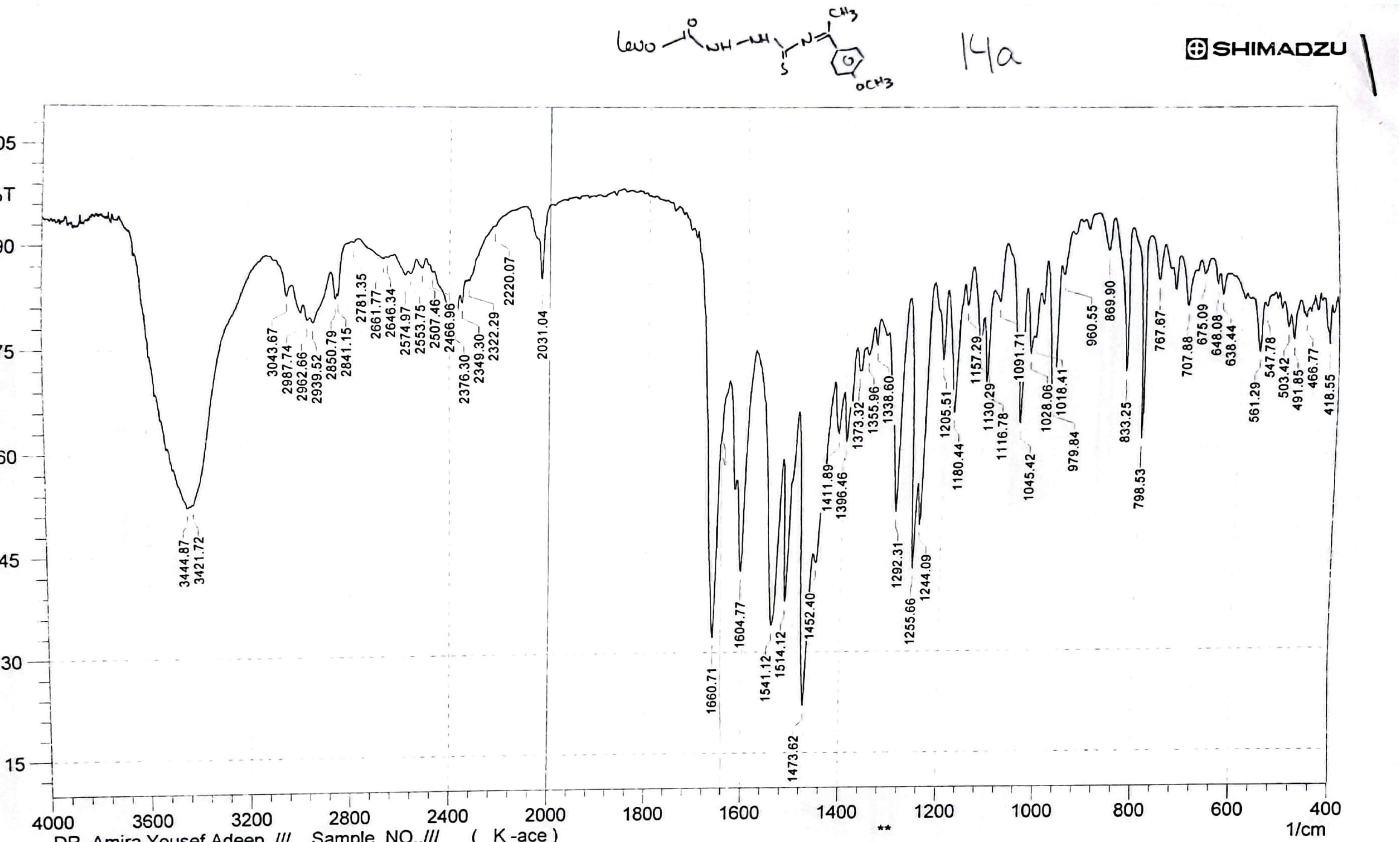

14a
SHIMADZU
%T
3444.87
3421.72
3043.67
2987.74
2962.66
2939.52
2850.79
2841.15
2781.35
2661.77
2646.34
2574.97
2553.75
2507.46
2466.96
2376.30
2349.30
2322.29
2220.07
2031.04
1660.71
1604.77
1541.12
1514.12
1473.62
1452.40
1411.89
1396.46
1373.32
1355.96
1338.60
1292.31
1255.66
1244.09
1205.51
1180.44
1157.29
1130.29
1116.78
1091.71
1045.42
1028.06
1018.41
979.84
960.55
869.90
833.25
798.53
767.67
707.88
675.09
648.08
638.44
561.29
547.78
503.42
491.85
466.77
418.55
4000
3600
3200
2800
2400
2000
1800
1600
1400
1200
1000
800
600
400
1/cm

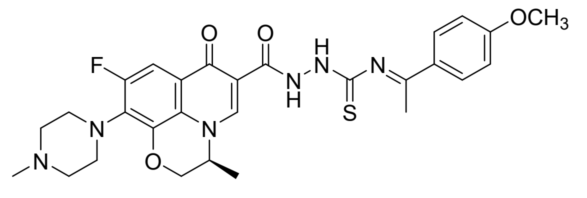

12a NMR

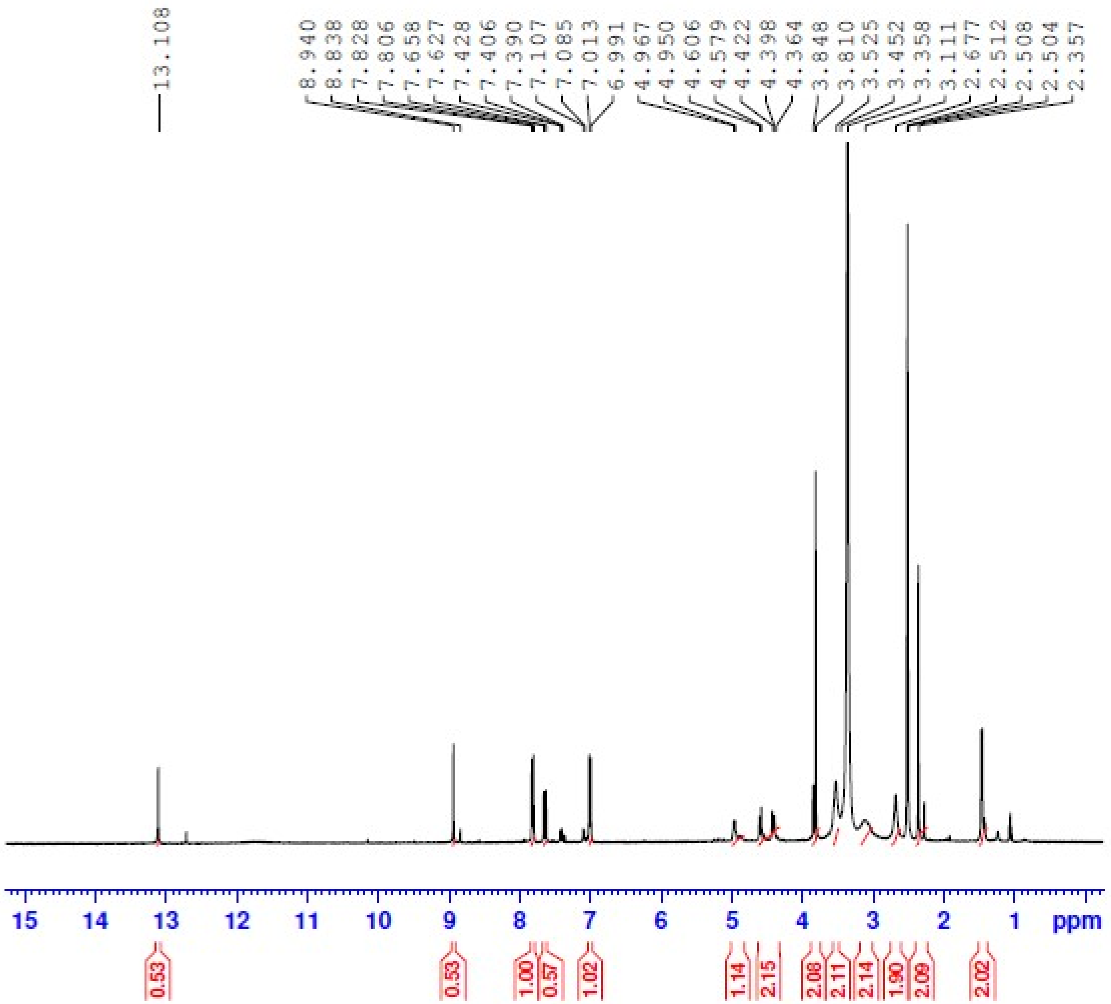

Current Data Parameters
NAME amira-youssef-K-ace
EXPNO 1
PROCNO 1

F2 - Acquisition Parameters
Date_ 20220426
Time 10.49
INSTRUM spect
PROBHD 5 mm PABBO BB/
PULPROG zg30
TD 65536
SOLVENT DMSO
NS 102
DS 2
SWH 8012.820 Hz
FIDRES 0.122266 Hz
AQ 4.0894465 sec
RG 205.37
DW 62.400 usec
DE 6.50 usec
TE 300.0 K
D1 1.00000000 sec
TD0 1

======== CHANNEL f1 ========
SFO1 400.1524711 MHz
NUC1 1H
P1 12.00 usec
PLW1 18.00000000 W

F2 - Processing parameters
SI 65536
SF 400.1500000 MHz
WDW EM
SSB 0
LB 0.30 Hz
GB 0
PC 1.00

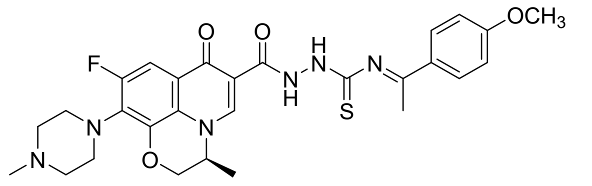
F
O
O
H
N
N
H
S
N
OCH3
N
N
O
N

12a D_2O

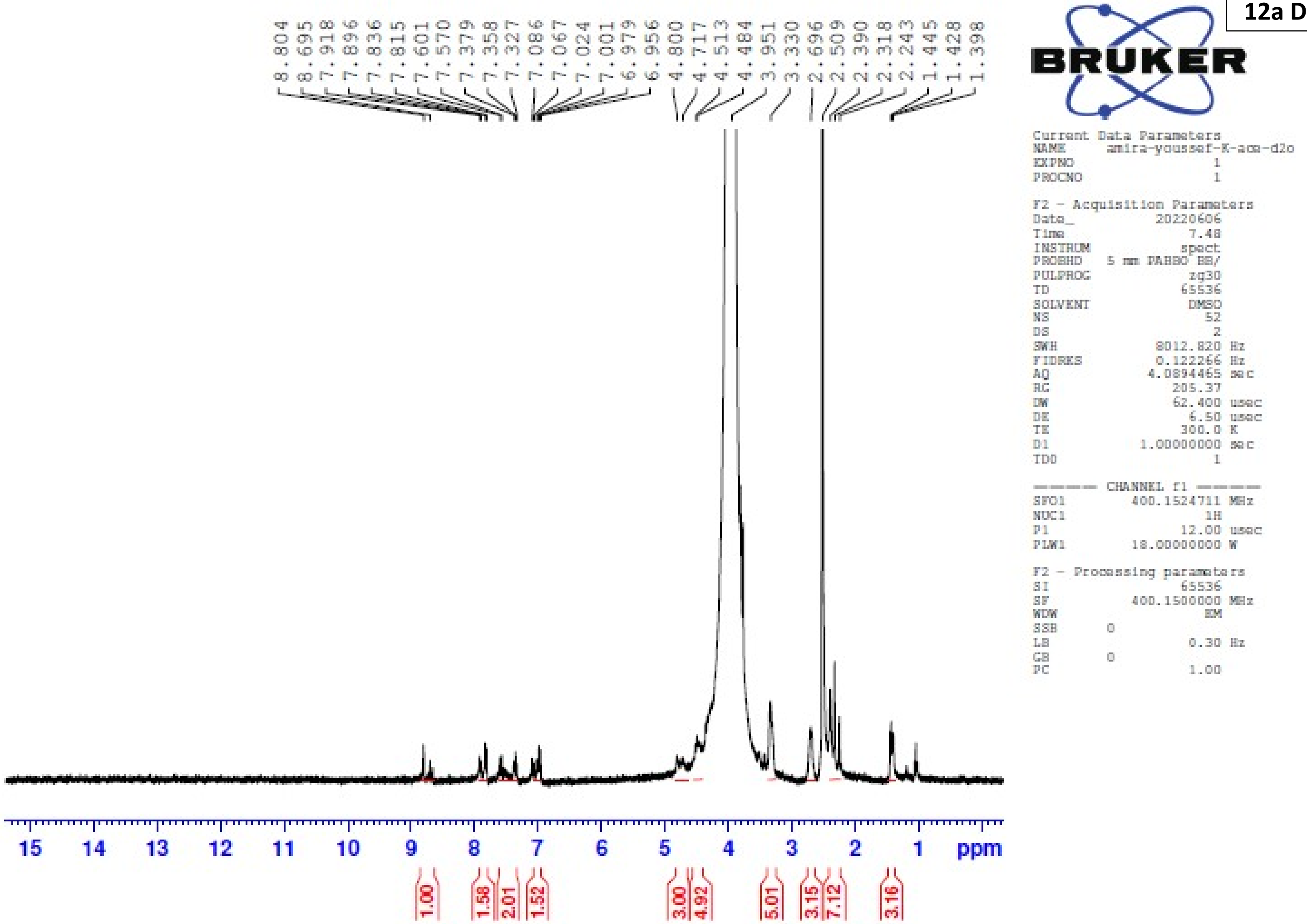
8.804
8.695
7.918
7.896
7.836
7.815
7.601
7.570
7.379
7.358
7.327
7.086
7.067
7.024
7.001
6.979
6.956
4.800
4.717
4.513
4.484
3.951
3.330
2.696
2.509
2.390
2.318
2.243
1.445
1.428
1.398
15 14 13 12 11 10 9 8 7 6 5 4 3 2 1 ppm
1.00
1.58
2.01
1.52
3.00
4.92
5.01
3.15
7.12
3.16
BRUKER
Current Data Parameters
NAME amira-youssef-K-aca-d2o
EXPNO 1
PROCNO 1
F2 - Acquisition Parameters
Date_ 20220606
Time 7.48
INSTRUM spect
PROBHD 5 mm PABBO BB/
PULPROG zg30
TD 65536
SOLVENT DMSO
NS 52
DS 2
SWH 8012.820 Hz
FIDRES 0.122266 Hz
AQ 4.0894465 sec
RG 205.37
DW 62.400 usec
DE 6.50 usec
TE 300.0 K
D1 1.00000000 sec
TD0 1
======== CHANNEL f1 ========
SFO1 400.1524711 MHz
NUC1 1H
P1 12.00 usec
PLW1 18.00000000 W
F2 - Processing parameters
SI 65536
SF 400.1500000 MHz
WDW EM
SSB 0
LB 0.30 Hz
GB 0
PC 1.00

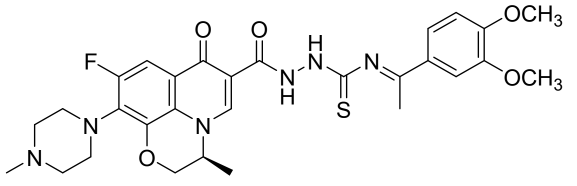
OCH3
OCH3
F
S

12b IR

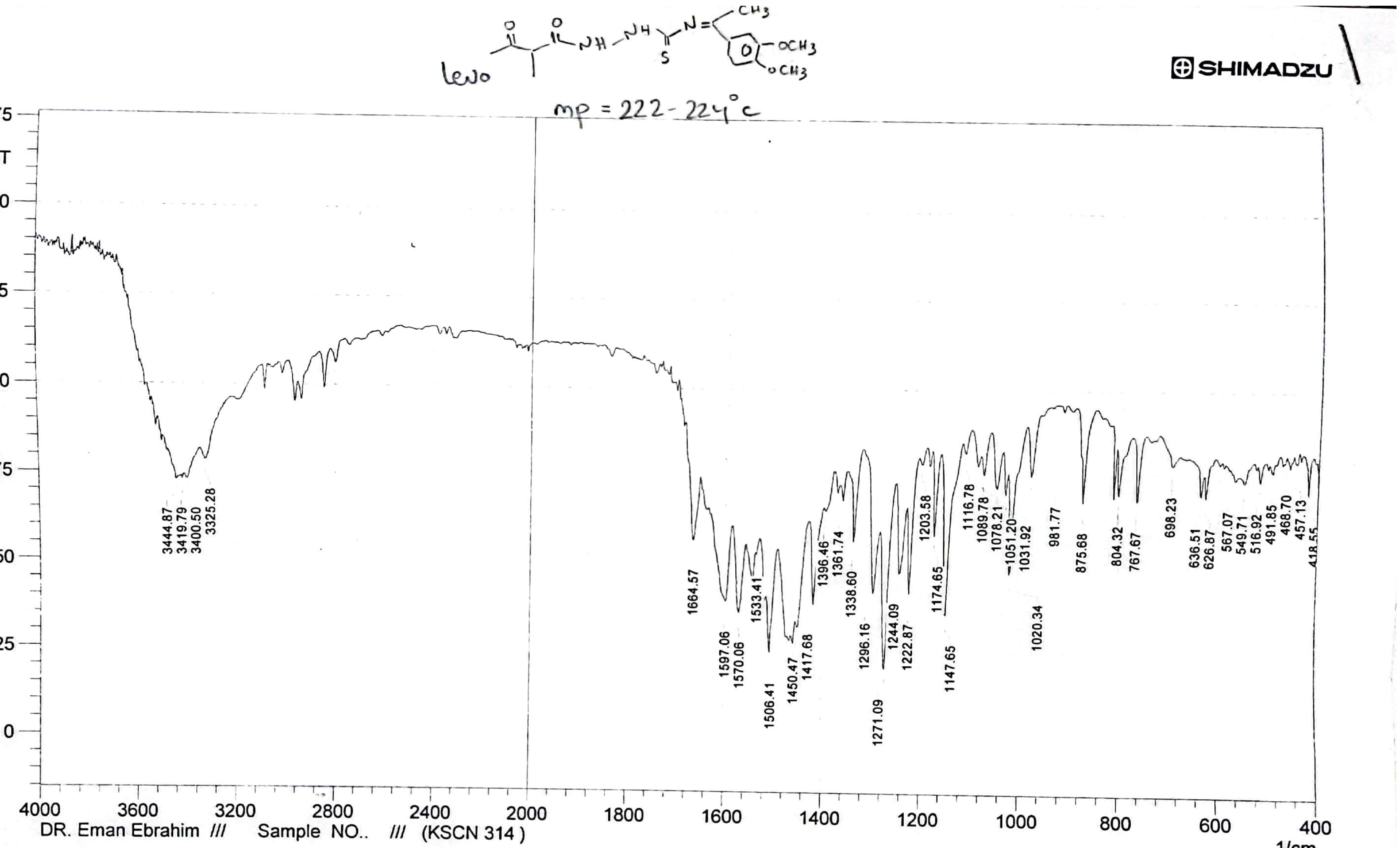
levo
mp = 222-224°c
SHIMADZU
3444.87
3419.79
3400.50
3325.28
1664.57
1597.06
1570.06
1533.41
1506.41
1450.47
1417.68
1396.46
1361.74
1338.60
1296.16
1271.09
1244.09
1222.87
1203.58
1174.65
1147.65
1116.78
1089.78
1078.21
1051.20
1031.92
1020.34
981.77
875.68
804.32
767.67
698.23
636.51
626.87
567.07
549.71
516.92
491.85
468.70
457.13
4000
3600
3200
2800
2400
2000
1800
1600
1400
1200
1000
800
600
400
DR. Eman Ebrahim /// Sample NO.. /// (KSCN 314)

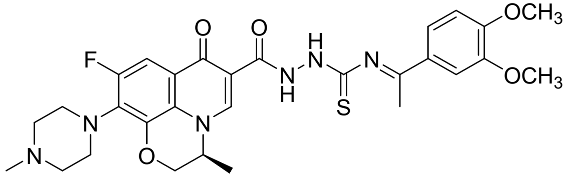

12b NMR

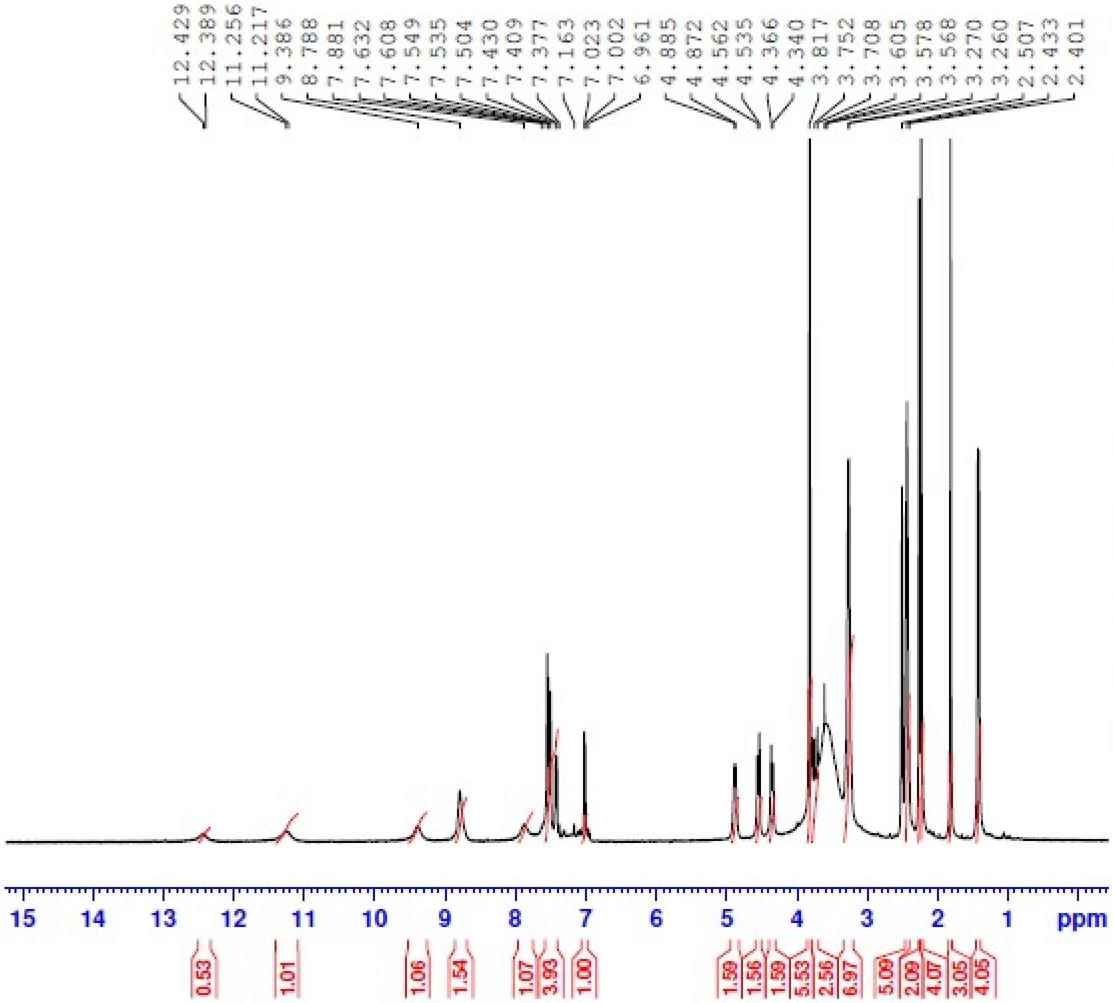


```
Current Data Parameters
NAME      amira-youssef-KSCN
EXPNO                    1
PROCNO                   1

F2 - Acquisition Parameters
Date_             20211125
Time                 14.46
INSTRUM              spect
PROBHD   5 mm PABBO BB/
PULPROG               zg30
TD                   65536
SOLVENT               DMSO
NS                      40
DS                       2
SWH             8012.820 Hz
FIDRES          0.122266 Hz
AQ             4.0894465 sec
RG                  205.37
DW                  62.400 usec
DE                    6.50 usec
TE                   300.0 K
D1            1.00000000 sec
TD0                      1

======== CHANNEL f1 ========
SFO1         400.1524711 MHz
NUC1                    1H
P1                   12.00 usec
PLW1         18.00000000 W

F2 - Processing parameters
SI                   65536
SF           400.1500000 MHz
WDW                     EM
SSB       0
LB                    0.30 Hz
GB        0
PC                    1.00
```

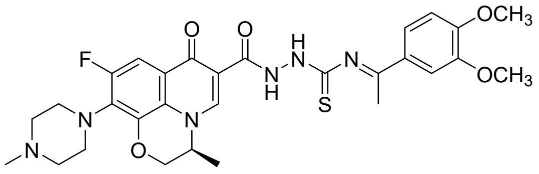

12b D_2O

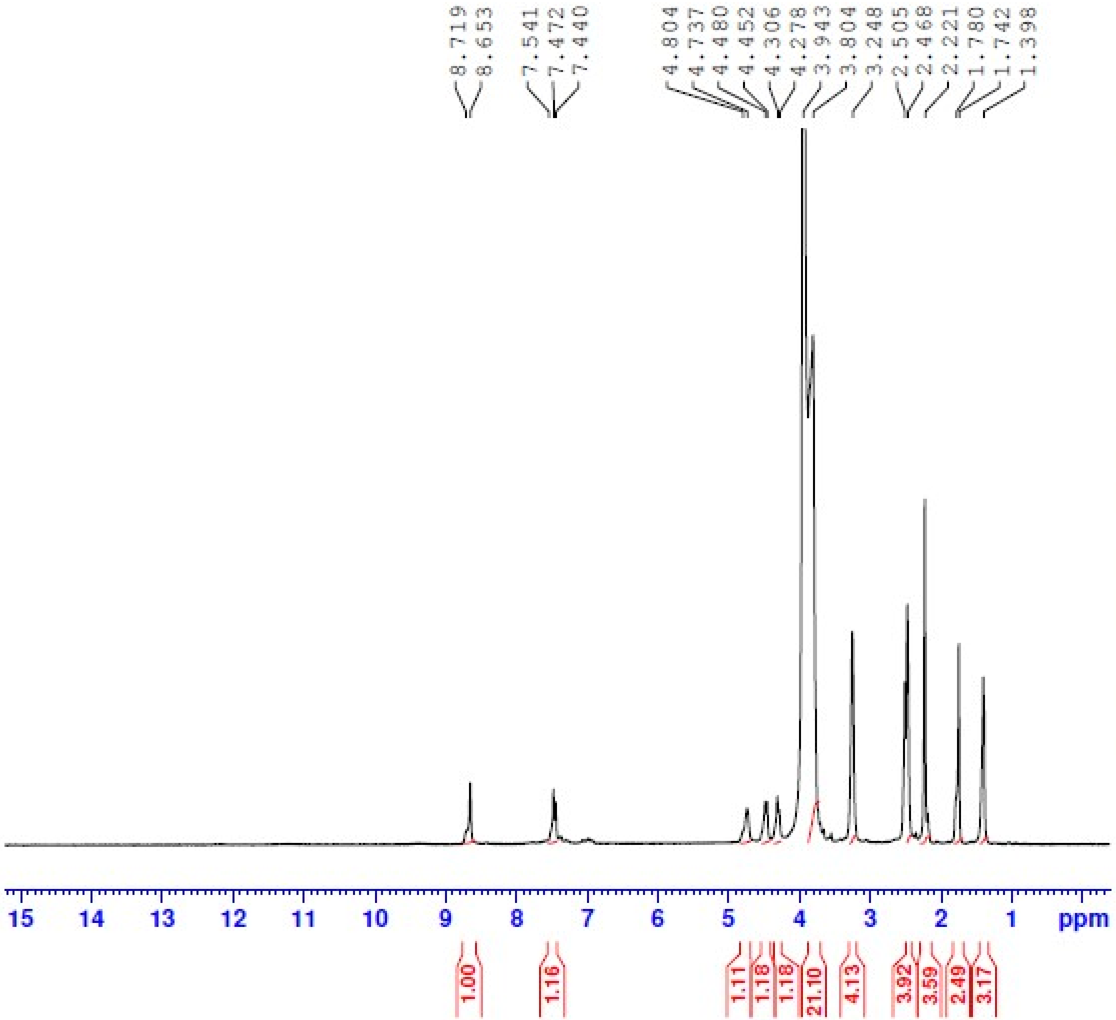

Current Data Parameters
NAME amira-youssef-KSCN-d2o
EXPNO 1
PROCNO 1

F2 - Acquisition Parameters
Date_ 20211212
Time 16.02
INSTRUM spect
PROBHD 5 mm PABBO BB/
PULPROG zg30
TD 65536
SOLVENT DMSO
NS 58
DS 2
SWH 8012.820 Hz
FIDRES 0.122266 Hz
AQ 4.0894465 sec
RG 31.47
DW 62.400 usec
DE 6.50 usec
TE 300.0 K
D1 1.00000000 sec
TD0 1

======== CHANNEL f1 ========
SFO1 400.1524711 MHz
NUC1 1H
P1 12.00 usec
PLW1 18.00000000 W

F2 - Processing parameters
SI 65536
SF 400.1500000 MHz
WDW EM
SSB 0
LB 0.30 Hz
GB 0
PC 1.00

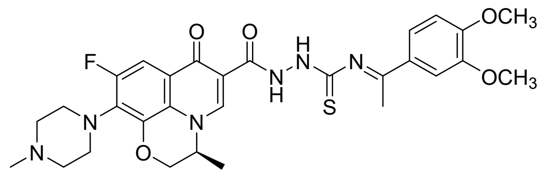

12b ^{13}C

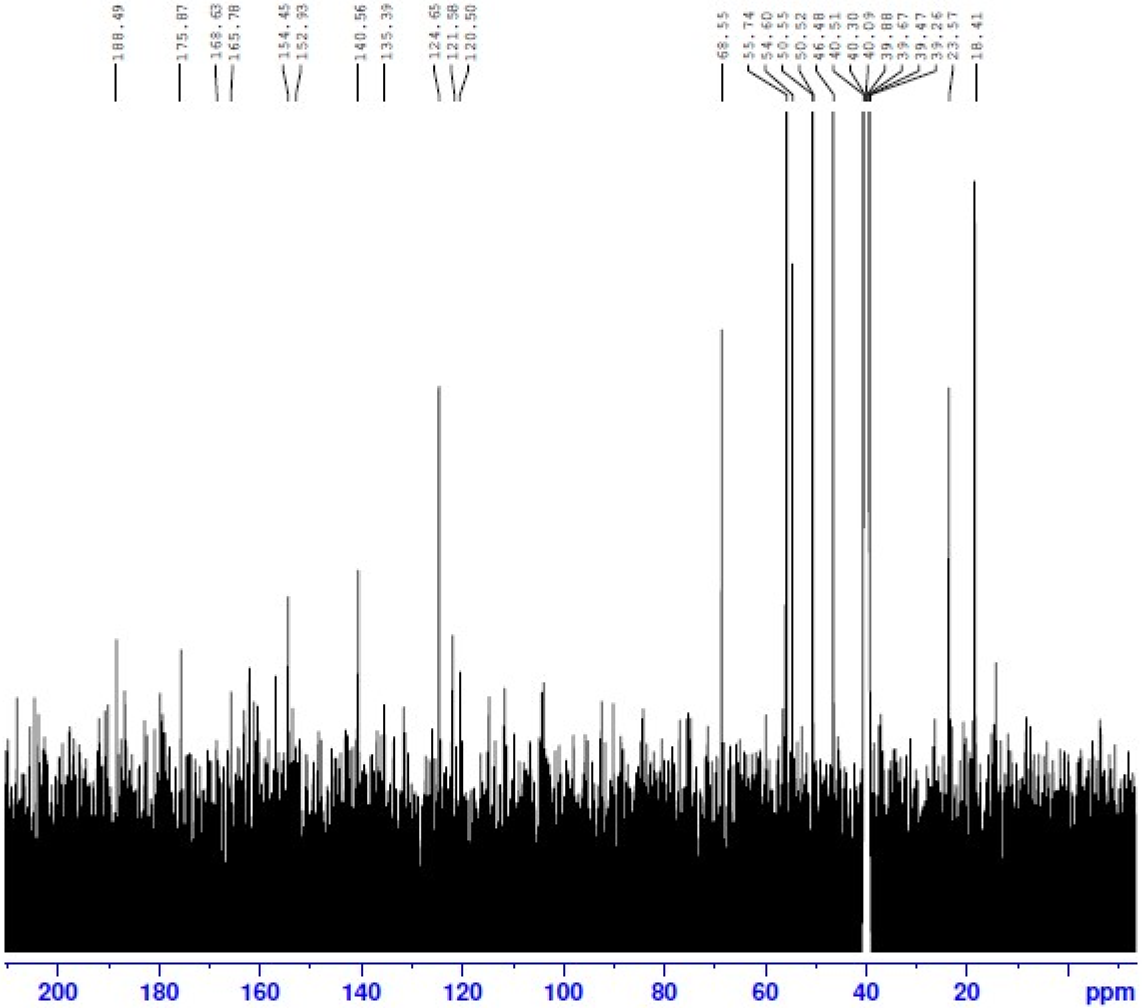


```
Current Data Parameters
NAME       amira-youssef-KSCN
EXPNO                       2
PROCNO                      1

F2 - Acquisition Parameters
Date_              20211212
Time                  15.52
INSTRUM               spect
PROBHD   5 mm PABBO BB/
PULPROG             zgpg30
TD                    65536
SOLVENT                DMSO
NS                     2000
DS                        4
SWH              24038.461 Hz
FIDRES            0.366798 Hz
AQ               1.3631488 sec
RG                  205.37
DW                  20.800 usec
DE                    6.50 usec
TE                   300.0 K
D1              2.00000000 sec
D11             0.03000000 sec
TD0                       1

======== CHANNEL f1 ========
SFO1           100.6278588 MHz
NUC1                    13C
P1                    10.00 usec
PLW1            47.00000000 W

======== CHANNEL f2 ========
SFO2           400.1516006 MHz
NUC2                     1H
CPDPRG[2            waltz16
PCPD2                 90.00 usec
PLW2            18.00000000 W
PLW12            0.34722000 W
PLW13            0.28125000 W

F2 - Processing parameters
SI                    32768
SF              100.6177975 MHz
WDW                      EM
SSB      0
LB                     1.00 Hz
GB       0
PC                     1.40
```

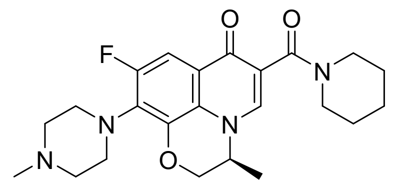

13a IR

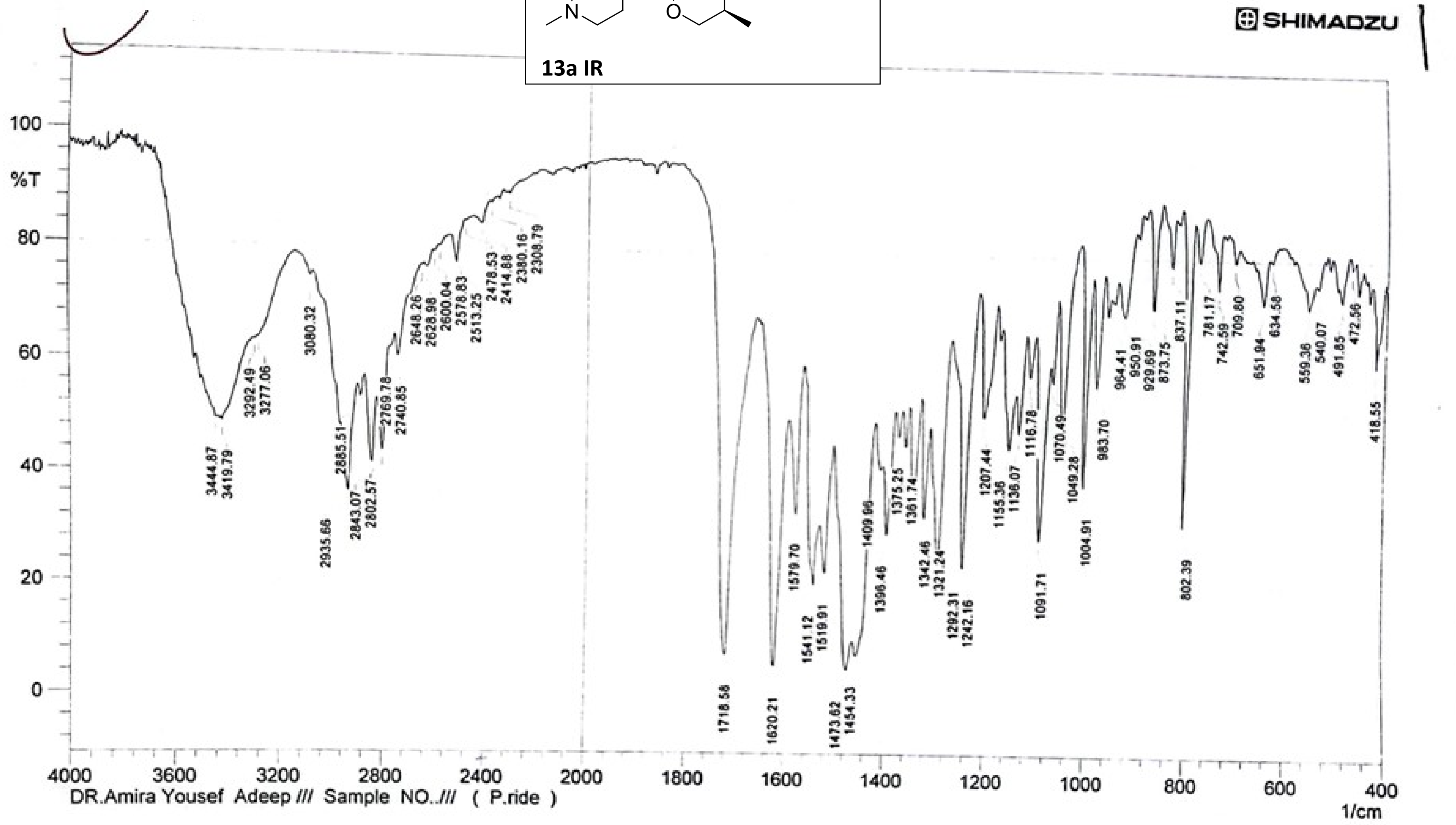

Comment;
DR.Amira Yousef Adeep /// Sample NO../// (P.ride
)
**Prepared By : Dr.Ahmed A.EL-Wakeel , Ph.D.

Date/Time; 10/4/2022 11:45:12 AM
No. of Scans; 10
Resolution; 4 [1/cm]
Apodization; Happ-Genzel
User; Heinrich

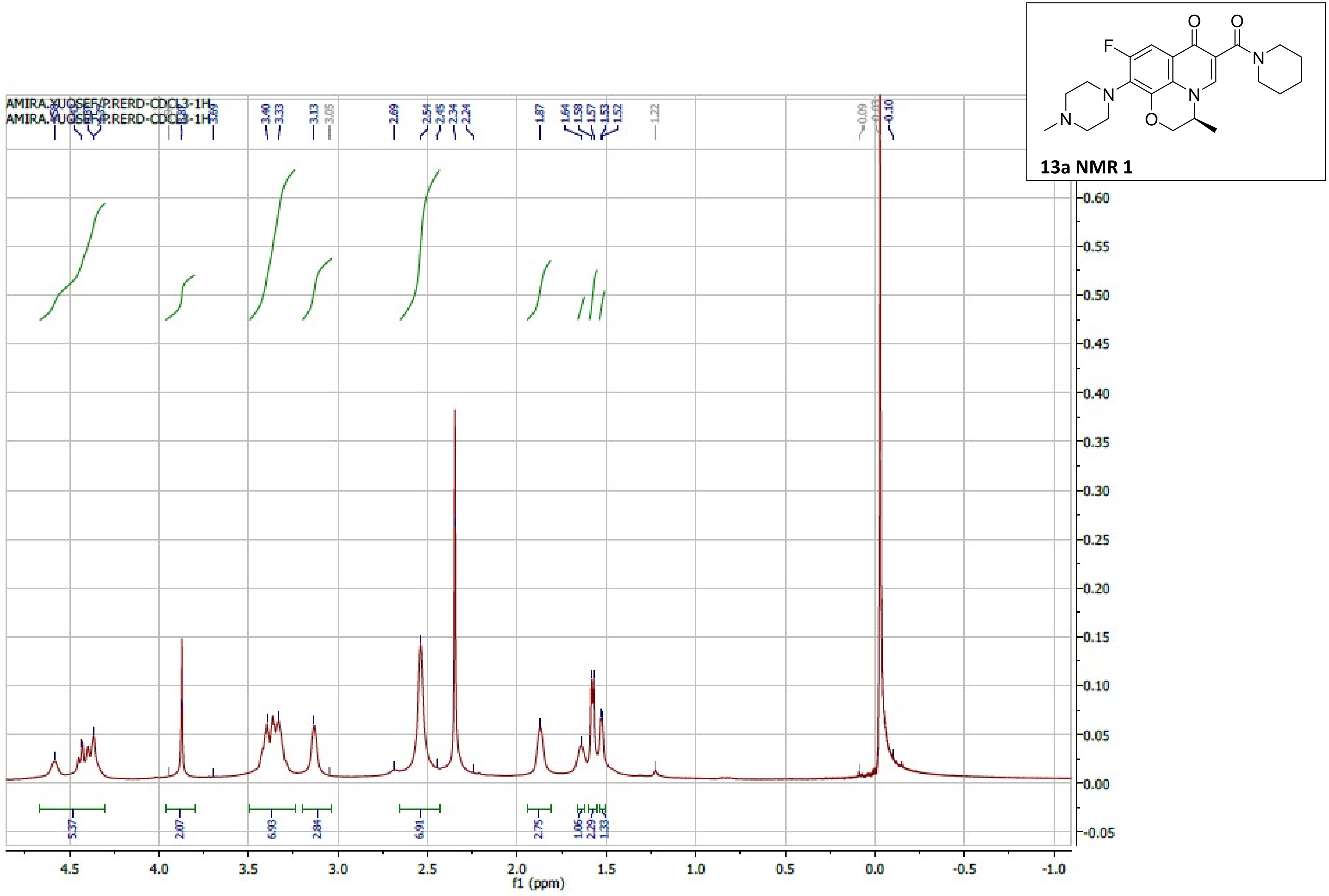

13a NMR 1

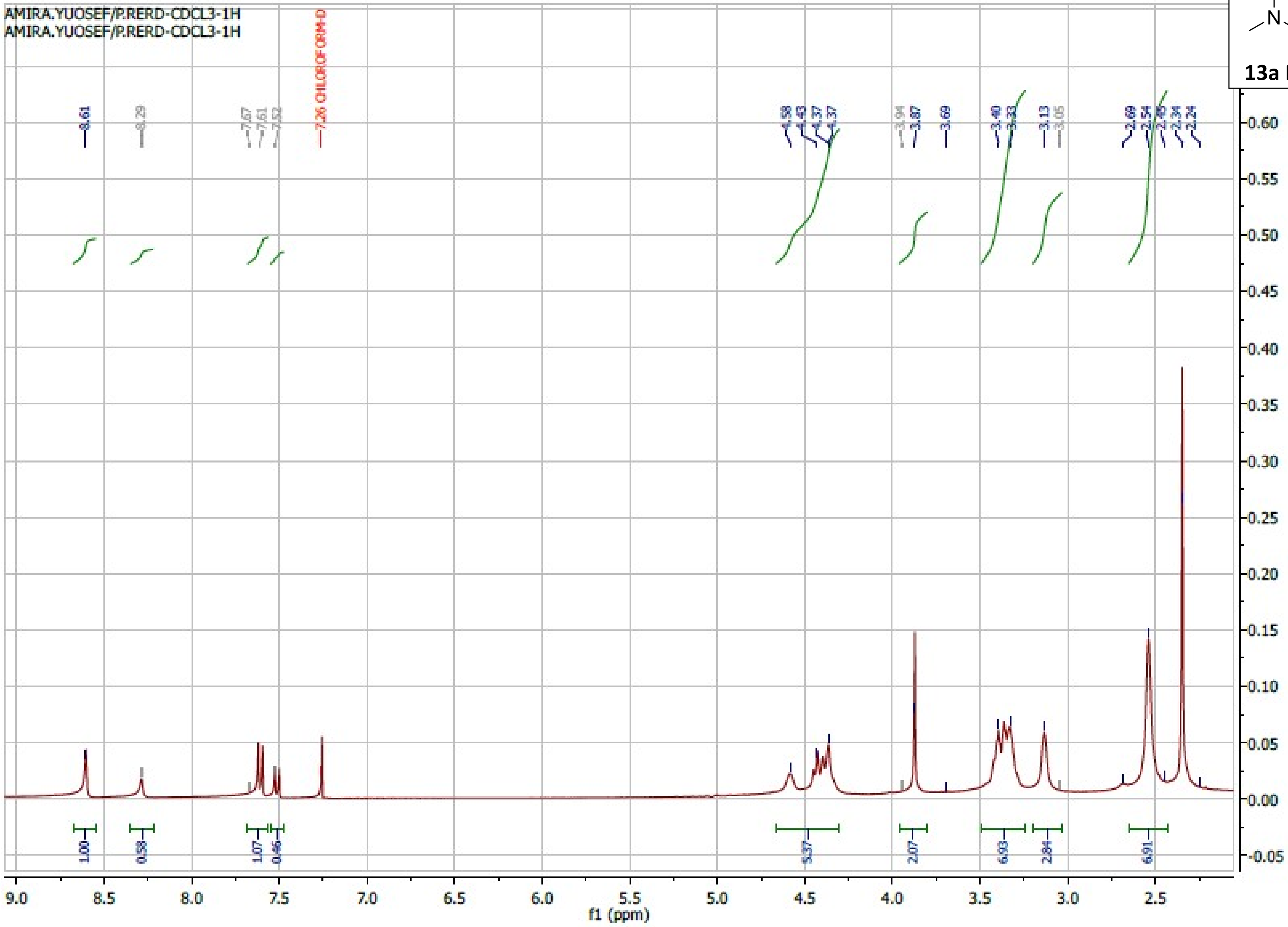
AMIRA.YUOSEF/P.RERD-CDCL3-1H
AMIRA.YUOSEF/P.RERD-CDCL3-1H
8.61
8.29
7.67
7.61
7.52
7.26 CHLOROFORM-D
4.58
4.43
4.37
4.37
3.94
3.87
3.69
3.40
3.33
3.13
3.05
2.69
2.54
2.45
2.34
2.24
1.00
0.58
1.07
0.46
5.37
2.07
6.93
2.84
6.91
f1 (ppm)

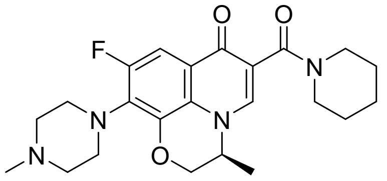
F
O
O
N
N
N
N
O

13a NMR 2

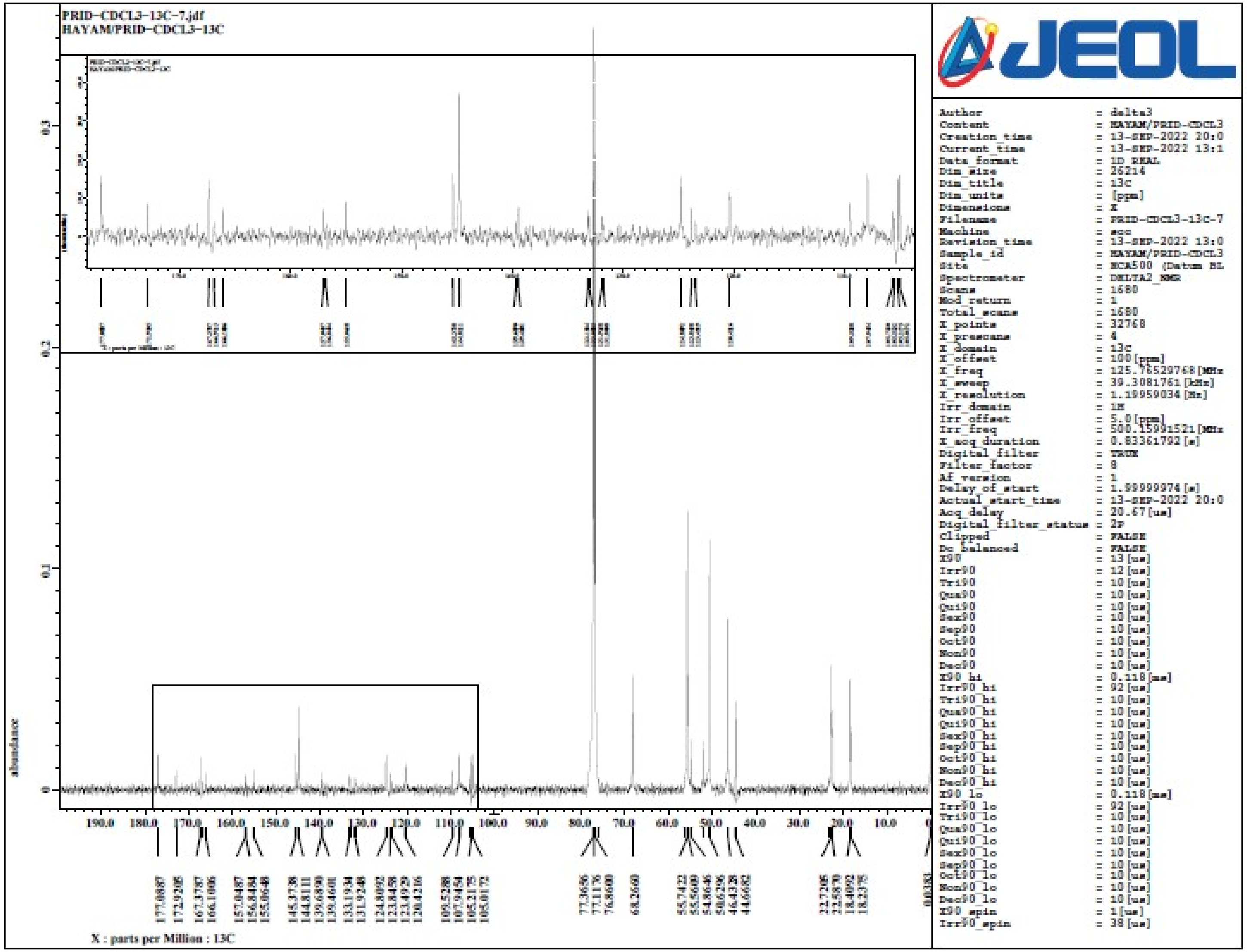
PRID-CDCL3-13C-7.jdf
HAYAM/PRID-CDCL3-13C
abundance
X : parts per Million : 13C
177.0887
172.9205
167.3787
166.1006
157.0487
156.8484
155.0648
145.3738
144.8111
139.6890
139.4601
133.1934
131.9248
124.8092
123.8458
123.4929
120.4216
109.5288
107.9454
105.2175
105.0172
77.3656
77.1176
76.8600
68.2660
55.7422
55.5609
54.8646
50.6296
46.4328
44.6682
22.7205
22.5870
18.4092
18.2375
0.0183
JEOL
Author = delta3
Content = HAYAM/PRID-CDCL3
Creation_time = 13-SEP-2022 20:0
Current_time = 13-SEP-2022 13:1
Data_format = 1D REAL
Dim_size = 26214
Dim_title = 13C
Dim_units = [ppm]
Dimensions = X
Filename = PRID-CDCL3-13C-7
Machine = ecc
Revision_time = 13-SEP-2022 13:0
Sample_id = HAYAM/PRID-CDCL3
Site = ECA500 (Datum BL
Spectrometer = DELTA2_NMR
Scans = 1680
Mod_return = 1
Total_scans = 1680
X_points = 32768
X_prescans = 4
X_domain = 13C
X_offset = 100[ppm]
X_freq = 125.76529768[MHz
X_sweep = 39.3081761[kHz]
X_resolution = 1.19959034[Hz]
Irr_domain = 1H
Irr_offset = 5.0[ppm]
Irr_freq = 500.15991521[MHz
X_acq_duration = 0.83361792[s]
Digital_filter = TRUE
Filter_factor = 8
Af_version = 1
Delay_of_start = 1.99999974[s]
Actual_start_time = 13-SEP-2022 20:0
Acq_delay = 20.67[us]
Digital_filter_status = 2P
Clipped = FALSE
Dc_balanced = FALSE

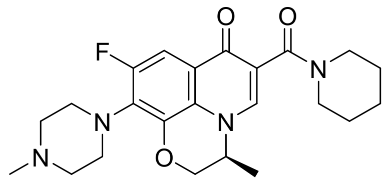
F
O
O
N
N
N
O
N

13a ^{13}C

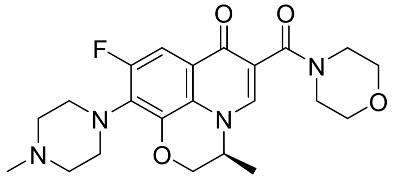
F
N
O

13b IR

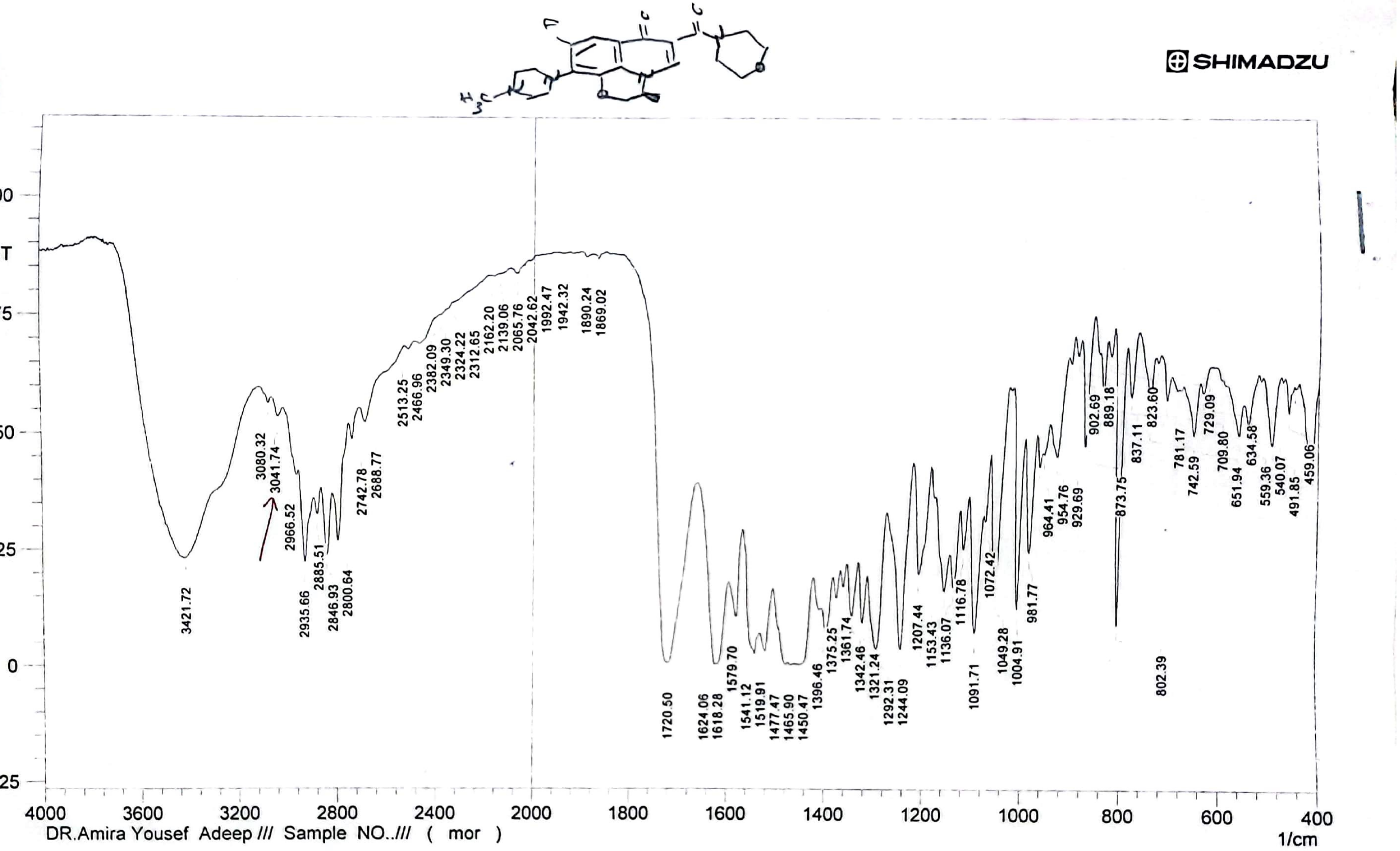
SHIMADZU
%T
3421.72
3080.32
3041.74
2966.52
2935.66
2885.51
2846.93
2800.64
2742.78
2688.77
2513.25
2466.96
2382.09
2349.30
2324.22
2312.65
2162.20
2139.06
2065.76
2042.62
1992.47
1942.32
1890.24
1869.02
1720.50
1624.06
1618.28
1579.70
1541.12
1519.91
1477.47
1465.90
1450.47
1396.46
1375.25
1361.74
1342.46
1321.24
1292.31
1244.09
1207.44
1153.43
1136.07
1116.78
1091.71
1072.42
1049.28
1004.91
981.77
964.41
954.76
929.69
902.69
889.18
873.75
837.11
823.60
802.39
781.17
742.59
729.09
709.80
651.94
634.58
559.36
540.07
491.85
459.06
4000
3600
3200
2800
2400
2000
1800
1600
1400
1200
1000
800
600
400
1/cm
DR.Amira Yousef Adeep /// Sample NO../// (mor)

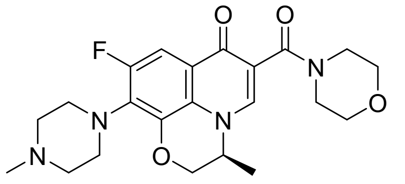
F
O
O
N
N
O
N
N
O

13b NMR

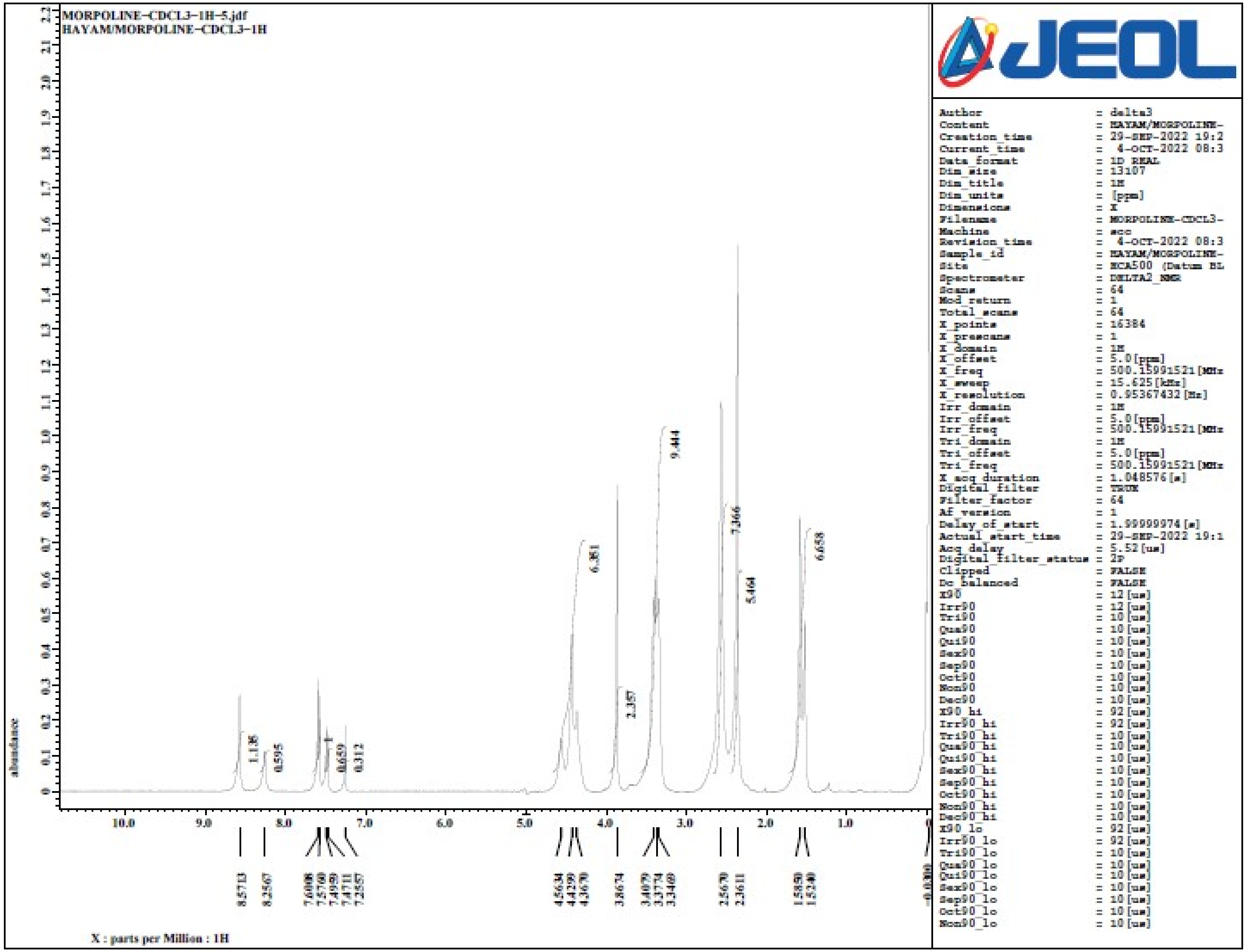
MORPOLINE-CDCL3-1H-5.jdf
HAYAM/MORPOLINE-CDCL3-1H
abundance
X : parts per Million : 1H
10.0 9.0 8.0 7.0 6.0 5.0 4.0 3.0 2.0 1.0
1.136 0.595 1 0.659 0.312 6.381 2.357 9.444 7.366 5.464 6.658
8.5713 8.2567 7.6008 7.5760 7.4959 7.4711 7.2557 4.5634 4.4299 4.3670 3.8674 3.4079 3.3774 3.3469 2.5670 2.3611 1.5850 1.5340
JEOL
Author = delta3
Content = HAYAM/MORPOLINE-
Creation_time = 29-SEP-2022 19:2
Current_time = 4-OCT-2022 08:3
Data_format = 1D REAL
Dim_size = 13107
Dim_title = 1H
Dim_units = [ppm]
Dimensions = X
Filename = MORPOLINE-CDCL3-
Machine = eco
Revision_time = 4-OCT-2022 08:3
Sample_id = HAYAM/MORPOLINE-
Site = ECA500 (Datum BL
Spectrometer = DELTA2_NMR
Scans = 64
Mod_return = 1
Total_scans = 64
X_points = 16384
X_prescans = 1
X_domain = 1H
X_offset = 5.0[ppm]
X_freq = 500.15991521[MHz
X_sweep = 15.625[kHz]
X_resolution = 0.95367432[Hz]
Irr_domain = 1H
Irr_offset = 5.0[ppm]
Irr_freq = 500.15991521[MHz
Tri_domain = 1H
Tri_offset = 5.0[ppm]
Tri_freq = 500.15991521[MHz
X_acq_duration = 1.048576[s]
Digital_filter = TRUE
Filter_factor = 64
Af_version = 1
Delay_of_start = 1.99999974[s]
Actual_start_time = 29-SEP-2022 19:1
Acq_delay = 5.52[us]
Digital_filter_status = 2P
Clipped = FALSE
Dc_balanced = FALSE
X90 = 12[us]
Irr90 = 12[us]
Tri90 = 10[us]
Qua90 = 10[us]
Qui90 = 10[us]
Sex90 = 10[us]
Sep90 = 10[us]
Oct90 = 10[us]
Non90 = 10[us]
Dec90 = 10[us]
X90_hi = 92[us]
Irr90_hi = 92[us]
Tri90_hi = 10[us]
Qua90_hi = 10[us]
Qui90_hi = 10[us]
Sex90_hi = 10[us]
Sep90_hi = 10[us]
Oct90_hi = 10[us]
Non90_hi = 10[us]
Dec90_hi = 10[us]
X90_lo = 92[us]
Irr90_lo = 92[us]
Tri90_lo = 10[us]
Qua90_lo = 10[us]
Qui90_lo = 10[us]
Sex90_lo = 10[us]
Sep90_lo = 10[us]
Oct90_lo = 10[us]
Non90_lo = 10[us]

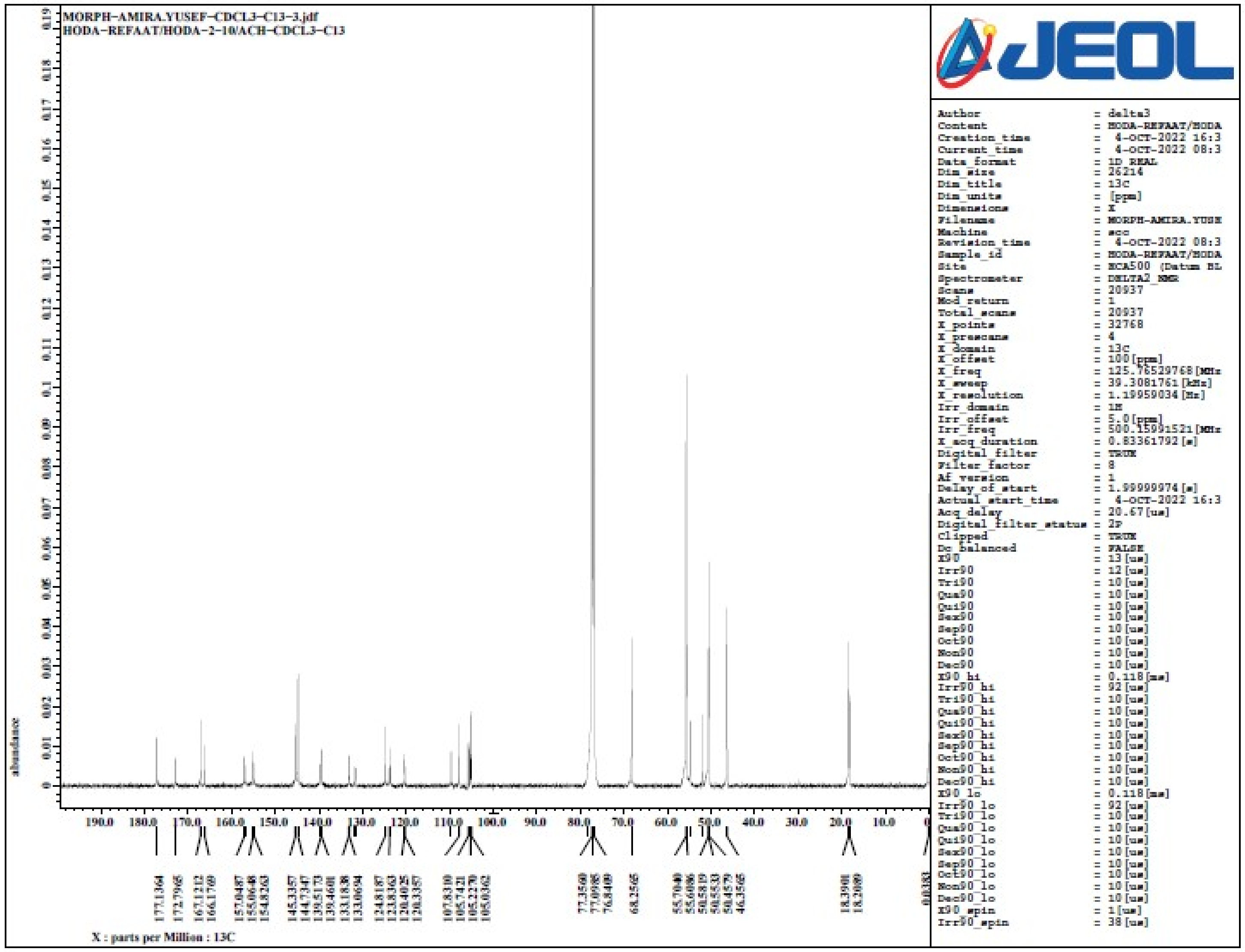

Author	=	delta3
Content	=	HODA-REFAAT/HODA
Creation_time	=	4-OCT-2022 16:3
Current_time	=	4-OCT-2022 08:3
Data_format	=	1D REAL
Dim_size	=	26214
Dim_title	=	13C
Dim_units	=	[ppm]
Dimensions	=	X
Filename	=	MORPH-AMIRA.YUSE
Machine	=	ecc
Revision_time	=	4-OCT-2022 08:3
Sample_id	=	HODA-REFAAT/HODA
Site	=	ECA500 (Datum BL
Spectrometer	=	DELTA2_NMR
Scans	=	20937
Mod_return	=	1
Total_scans	=	20937
X_points	=	32768
X_prescans	=	4
X_domain	=	13C
X_offset	=	100[ppm]
X_freq	=	125.76529768[MHz
X_sweep	=	39.3081761[kHz]
X_resolution	=	1.19959034[Hz]
Irr_domain	=	1H
Irr_offset	=	5.0[ppm]
Irr_freq	=	500.15991521[MHz
X_acq_duration	=	0.83361792[s]
Digital_filter	=	TRUE
Filter_factor	=	8
Af_version	=	1
Delay_of_start	=	1.99999974[s]
Actual_start_time	=	4-OCT-2022 16:3
Acq_delay	=	20.67[us]
Digital_filter_status	=	2P
Clipped	=	TRUE
Dc_balanced	=	FALSE
X90	=	13[us]
Irr90	=	12[us]
Tri90	=	10[us]
Qua90	=	10[us]
Qui90	=	10[us]
Sex90	=	10[us]
Sep90	=	10[us]
Oct90	=	10[us]
Non90	=	10[us]
Dec90	=	10[us]
X90_hi	=	0.118[ms]
Irr90_hi	=	92[us]
Tri90_hi	=	10[us]
Qua90_hi	=	10[us]
Qui90_hi	=	10[us]
Sex90_hi	=	10[us]
Sep90_hi	=	10[us]
Oct90_hi	=	10[us]
Non90_hi	=	10[us]
Dec90_hi	=	10[us]
X90_lo	=	0.118[ms]
Irr90_lo	=	92[us]
Tri90_lo	=	10[us]
Qua90_lo	=	10[us]
Qui90_lo	=	10[us]
Sex90_lo	=	10[us]
Sep90_lo	=	10[us]
Oct90_lo	=	10[us]
Non90_lo	=	10[us]
Dec90_lo	=	10[us]
X90_spin	=	1[us]
Irr90_spin	=	38[us]

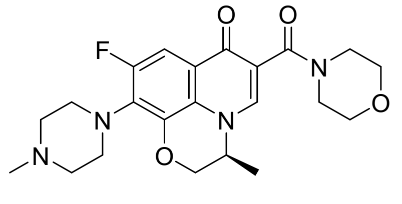

13b ^{13}C

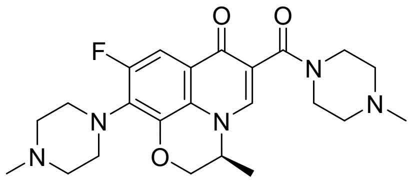

13c IR

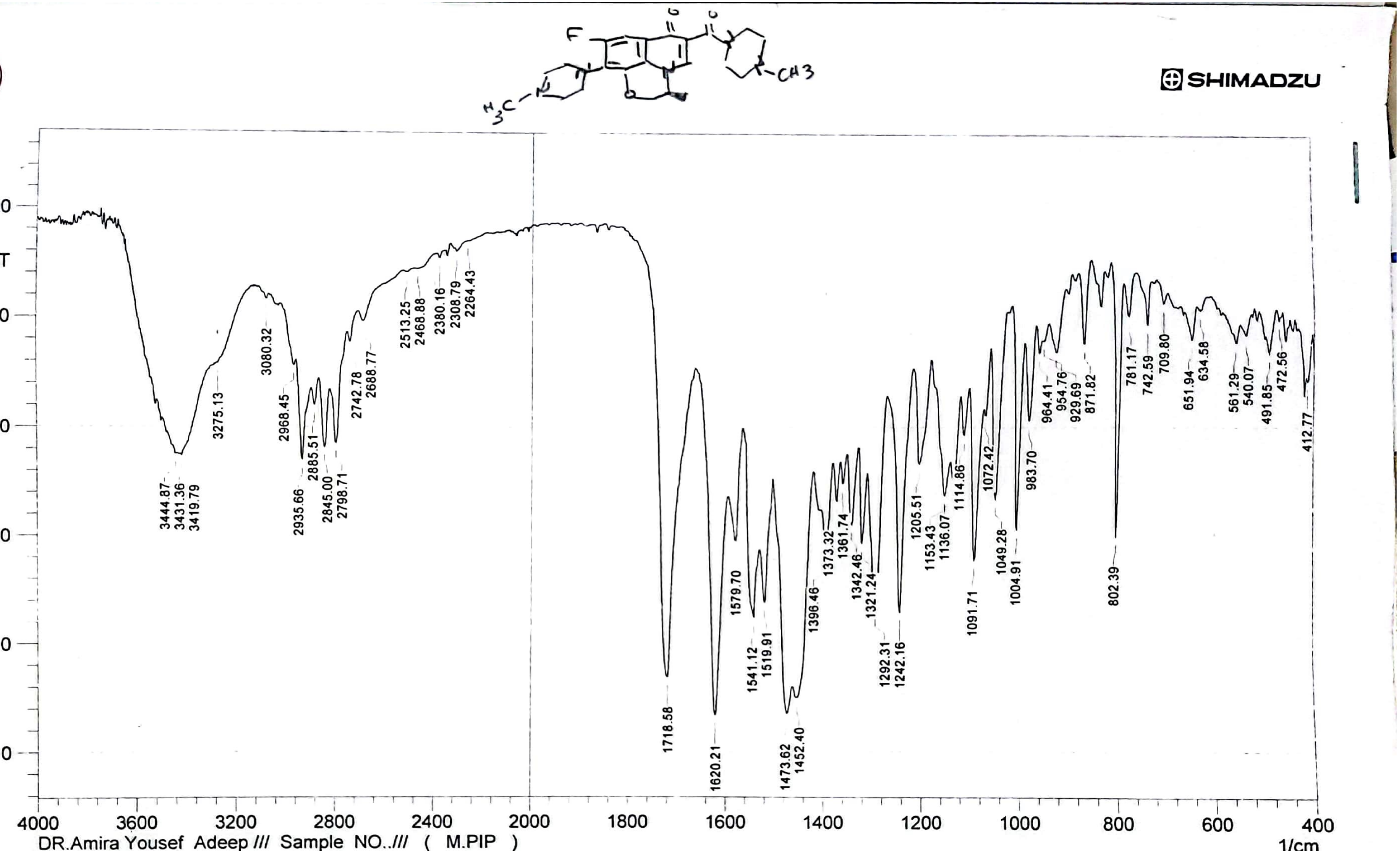
SHIMADZU
3444.87
3431.36
3419.79
3275.13
3080.32
2968.45
2935.66
2885.51
2845.00
2798.71
2742.78
2688.77
2513.25
2468.88
2380.16
2308.79
2264.43
1718.58
1620.21
1579.70
1541.12
1519.91
1473.62
1452.40
1396.46
1373.32
1361.74
1342.46
1321.24
1292.31
1242.16
1205.51
1153.43
1136.07
1114.86
1091.71
1072.42
1049.28
1004.91
983.70
964.41
954.76
929.69
871.82
802.39
781.17
742.59
709.80
651.94
634.58
561.29
540.07
491.85
472.56
412.77
4000
3600
3200
2800
2400
2000
1800
1600
1400
1200
1000
800
600
400
1/cm
DR.Amira Yousef Adeep /// Sample NO../// (M.PIP)

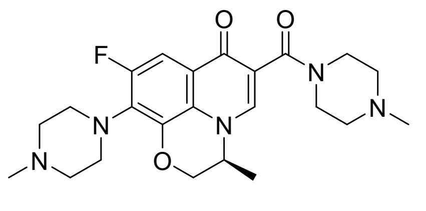
F
O
O
N
N
N
O
N
N

13c NMR

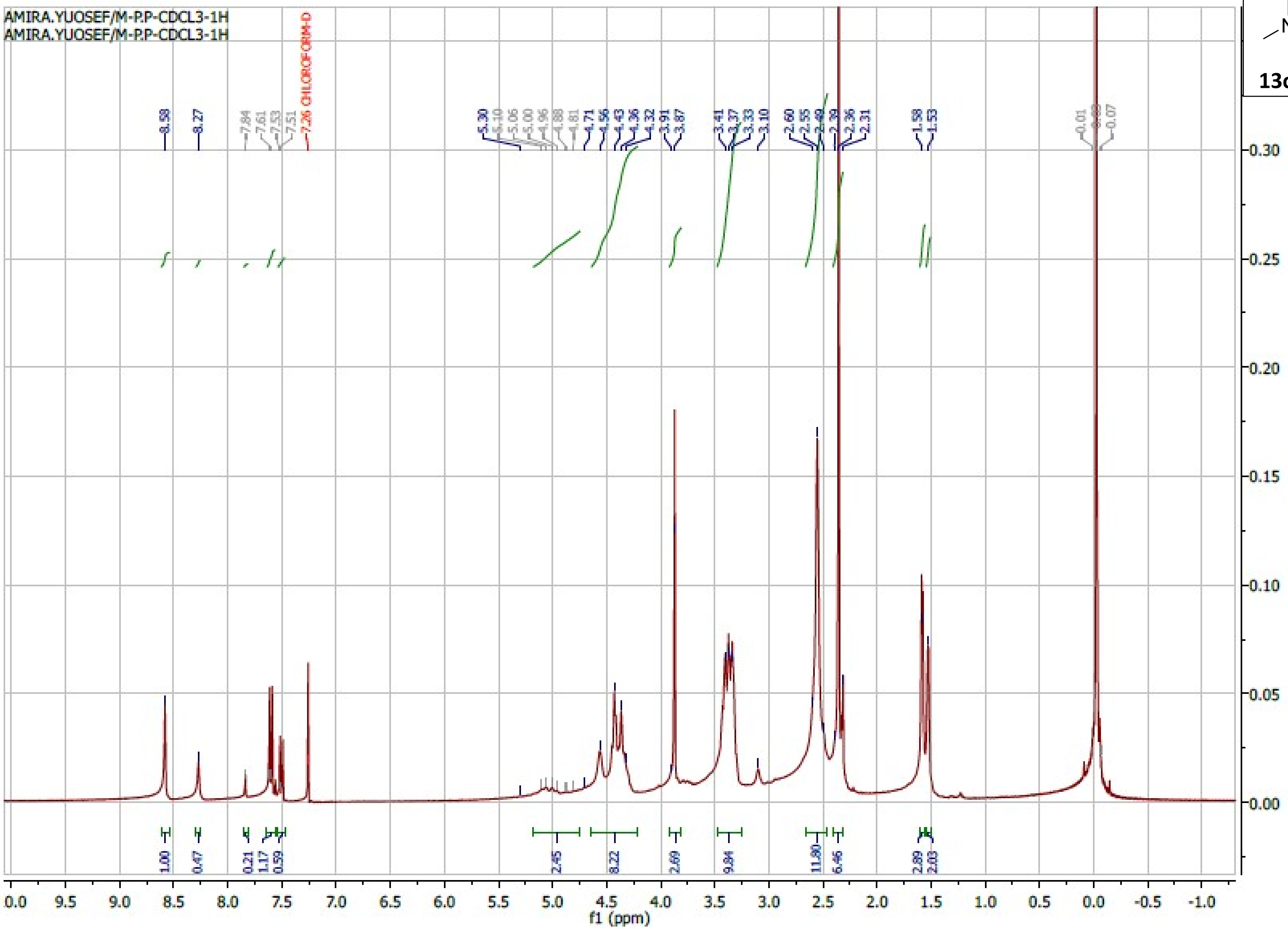
AMIRA.YUOSEF/M-P.P-CDCL3-1H
AMIRA.YUOSEF/M-P.P-CDCL3-1H
7.26 CHLOROFORM-D
8.58
8.27
7.84
7.61
7.53
7.51
5.30
5.10
5.06
5.00
4.96
4.88
4.81
4.71
4.56
4.43
4.36
4.32
3.91
3.87
3.41
3.37
3.33
3.10
2.60
2.55
2.49
2.39
2.36
2.31
1.58
1.53
0.01
0.00
-0.07
1.00
0.47
0.21
1.17
0.59
2.45
8.22
2.69
9.84
11.80
6.46
2.89
2.03
f1 (ppm)

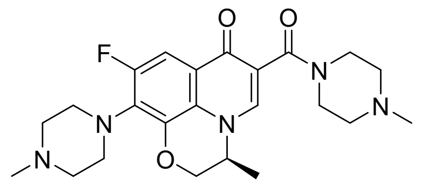

13c ^{13}C

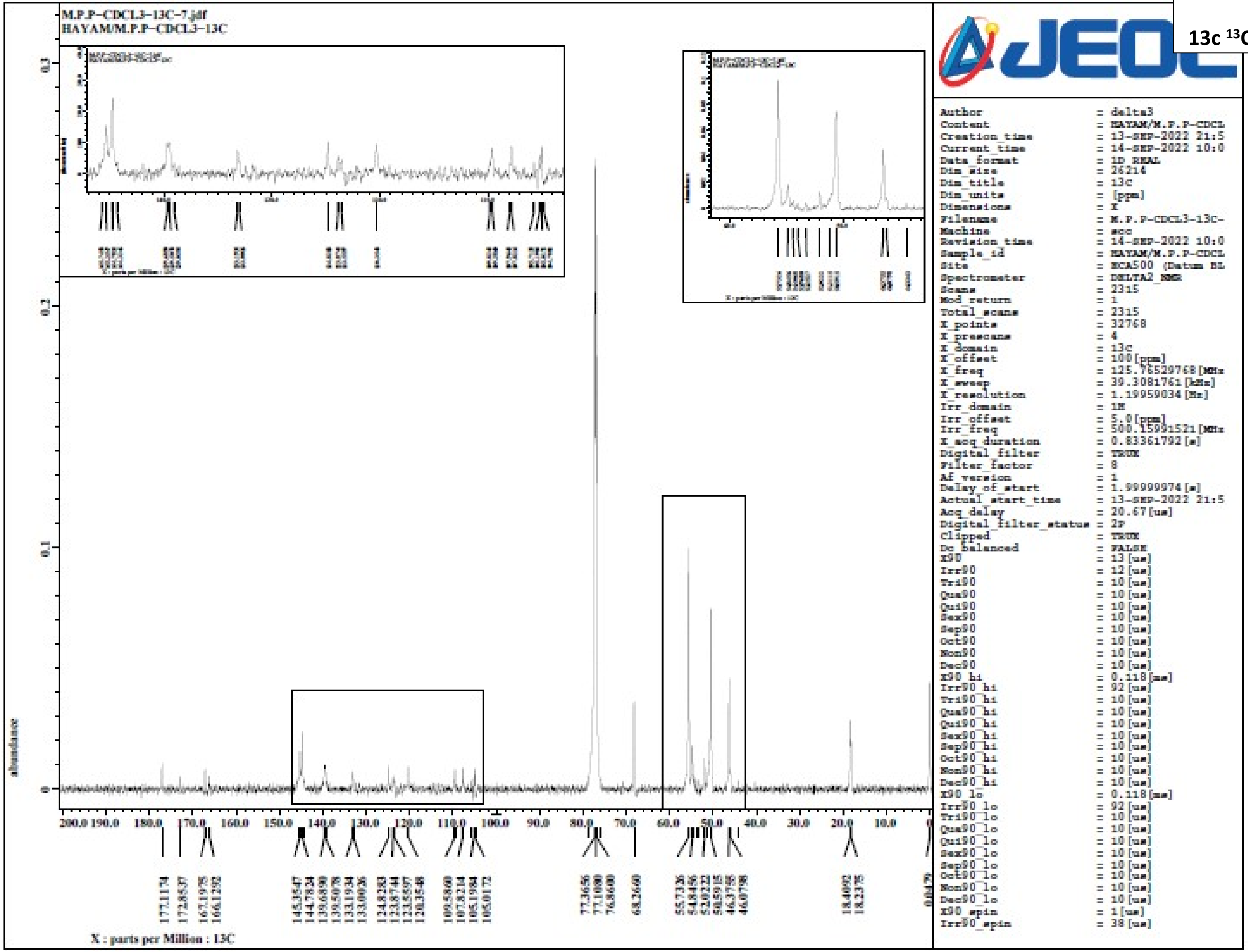
M.P.P-CDCL3-13C-7.jdf
HAYAM/M.P.P-CDCL3-13C
abundance
X : parts per Million : 13C
177.1174
172.8537
167.1975
166.1292
145.3547
144.7824
139.6890
139.5078
133.1934
133.0036
124.8283
123.8744
123.5597
120.3548
109.5860
107.8214
105.1984
105.0172
77.3656
77.1080
76.8600
68.2660
55.7336
54.8456
52.0232
50.5915
46.3758
46.0798
18.4092
18.2375
0.0479
Author = delta3
Content = HAYAM/M.P.P-CDCL
Creation_time = 13-SEP-2022 21:5
Current_time = 14-SEP-2022 10:0
Data_format = 1D REAL
Dim_size = 26214
Dim_title = 13C
Dim_units = [ppm]
Dimensions = X
Filename = M.P.P-CDCL3-13C-
Machine = eca
Revision_time = 14-SEP-2022 10:0
Sample_id = HAYAM/M.P.P-CDCL
Site = ECA500 (Datum BL
Spectrometer = DELTA2_NMR
Scans = 2315
Mod_return = 1
Total_scans = 2315
X_points = 32768
X_prescans = 4
X_domain = 13C
X_offset = 100[ppm]
X_freq = 125.76529768[MHz
X_sweep = 39.3081761[kHz]
X_resolution = 1.19959034[Hz]
Irr_domain = 1H
Irr_offset = 5.0[ppm]
Irr_freq = 500.15991521[MHz
X_acq_duration = 0.83361792[s]
Digital_filter = TRUE
Filter_factor = 8
Af_version = 1
Delay_of_start = 1.99999974[s]
Actual_start_time = 13-SEP-2022 21:5
Acq_delay = 20.67[us]
Digital_filter_status = 2P
Clipped = TRUE
Dc_balanced = FALSE
X90 = 13[us]
Irr90 = 12[us]
Tri90 = 10[us]
Qua90 = 10[us]
Qui90 = 10[us]
Sex90 = 10[us]
Sep90 = 10[us]
Oct90 = 10[us]
Non90 = 10[us]
Dec90 = 10[us]
X90_hi = 0.118[ms]
Irr90_hi = 92[us]
Tri90_hi = 10[us]
Qua90_hi = 10[us]
Qui90_hi = 10[us]
Sex90_hi = 10[us]
Sep90_hi = 10[us]
Oct90_hi = 10[us]
Non90_hi = 10[us]
Dec90_hi = 10[us]
X90_lo = 0.118[ms]
Irr90_lo = 92[us]
Tri90_lo = 10[us]
Qua90_lo = 10[us]
Qui90_lo = 10[us]
Sex90_lo = 10[us]
Sep90_lo = 10[us]
Oct90_lo = 10[us]
Non90_lo = 10[us]
Dec90_lo = 10[us]
X90_spin = 1[us]
Irr90_spin = 38[us]

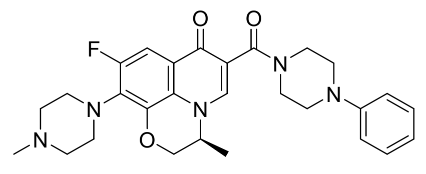

13d IR

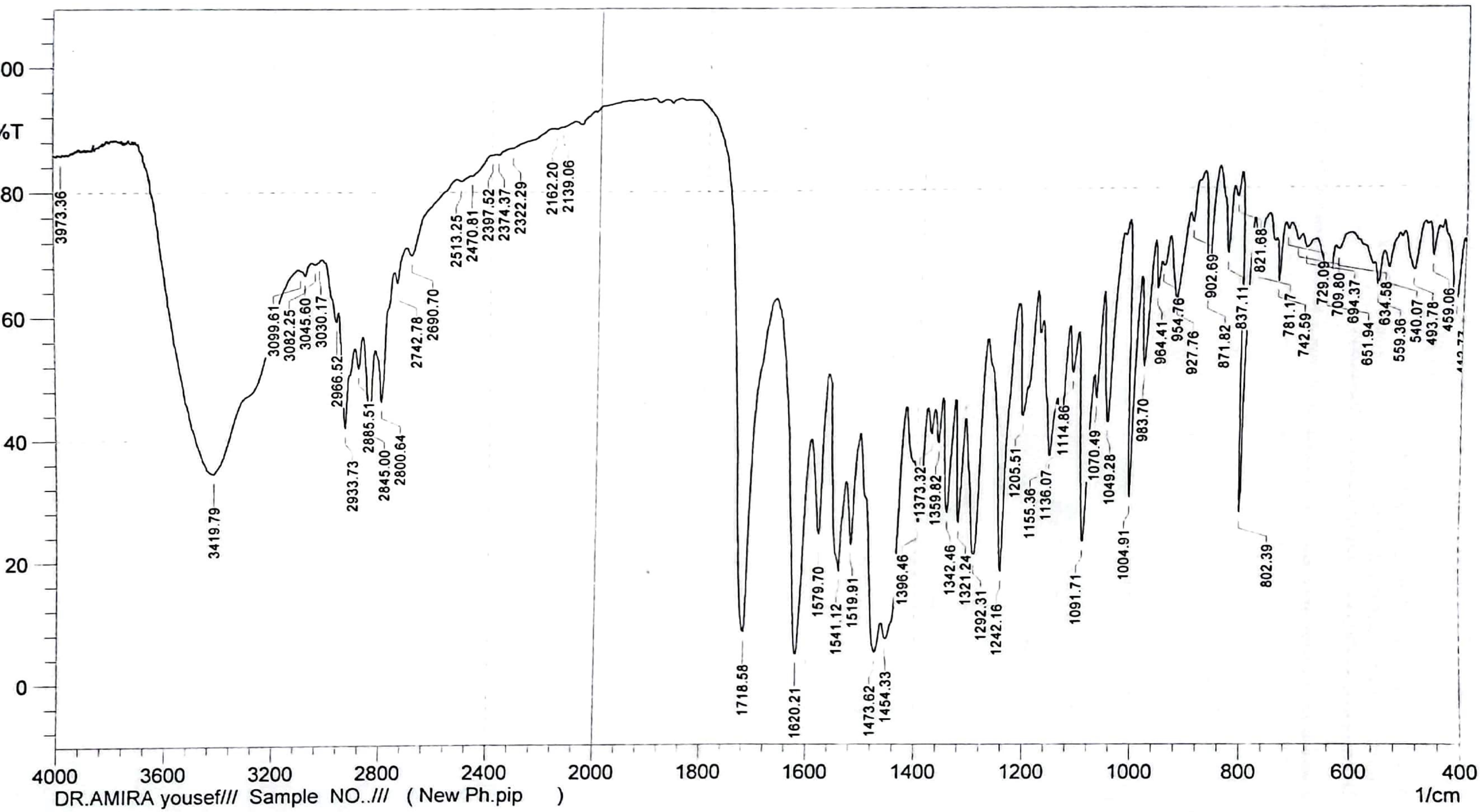
SHIMADZU
%T
00
80
60
40
20
0
3973.36
3419.79
3099.61
3082.25
3045.60
3030.17
2966.52
2933.73
2885.51
2845.00
2800.64
2742.78
2690.70
2513.25
2470.81
2397.52
2374.37
2322.29
2162.20
2139.06
1718.58
1620.21
1579.70
1541.12
1519.91
1473.62
1454.33
1396.46
1373.32
1359.82
1342.46
1321.24
1292.31
1242.16
1205.51
1155.36
1136.07
1114.86
1091.71
1070.49
1049.28
1004.91
983.70
964.41
954.76
927.76
902.69
871.82
837.11
821.68
802.39
781.17
742.59
729.09
709.80
694.37
651.94
634.58
559.36
540.07
493.78
459.06
4000
3600
3200
2800
2400
2000
1800
1600
1400
1200
1000
800
600
400
1/cm
DR.AMIRA yousef/// Sample NO../// (New Ph.pip)

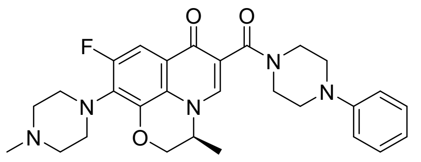

13d NMR

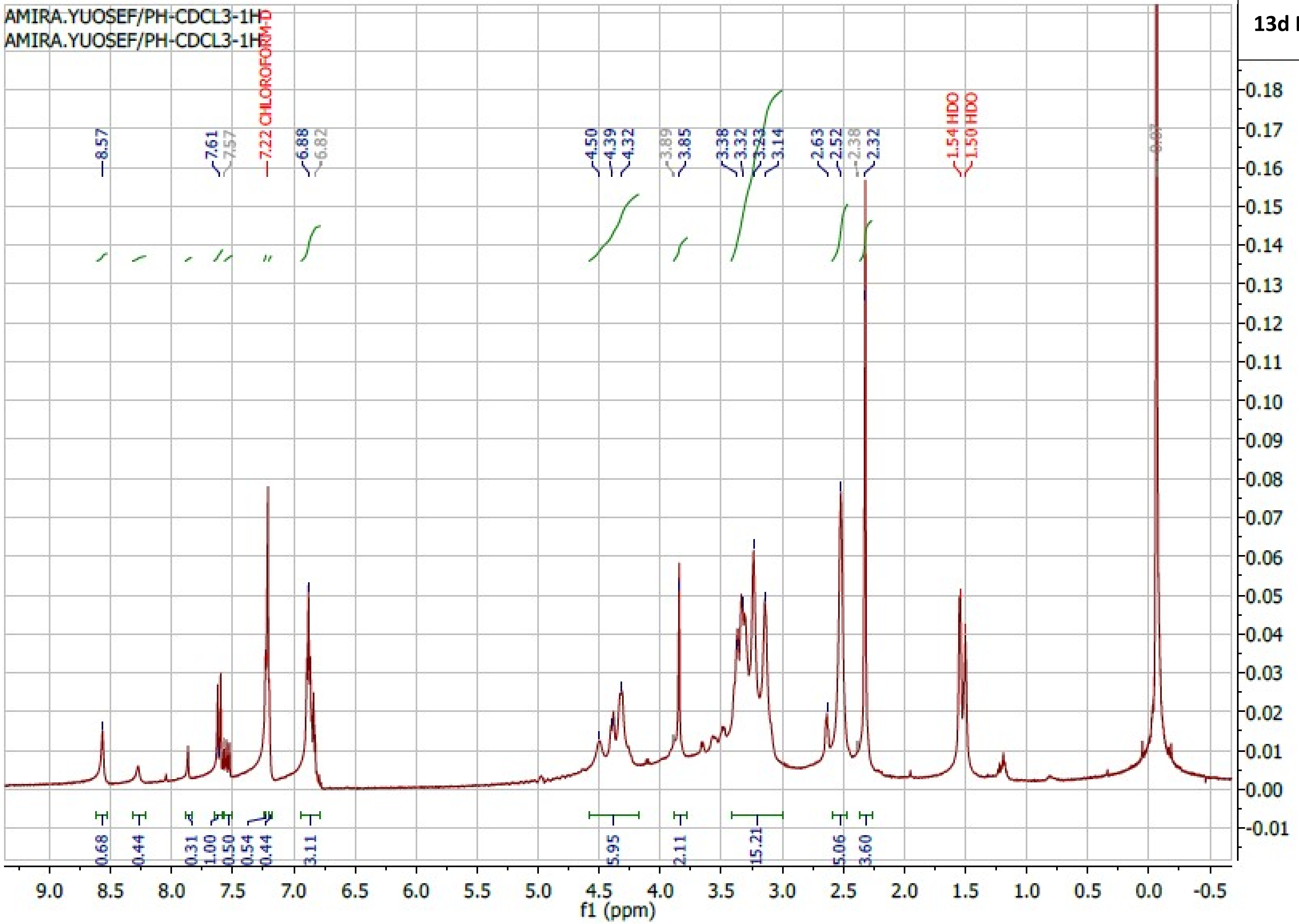
AMIRA.YUOSEF/PH-CDCL3-1H
AMIRA.YUOSEF/PH-CDCL3-1H
8.57
7.61
7.57
7.22 CHLOROFORM-D
6.88
6.82
4.50
4.39
4.32
3.89
3.85
3.38
3.32
3.23
3.14
2.63
2.52
2.38
2.32
1.54 HDO
1.50 HDO
0.68
0.44
0.31
1.00
0.50
0.54
0.44
3.11
5.95
2.11
15.21
5.06
3.60
f1 (ppm)

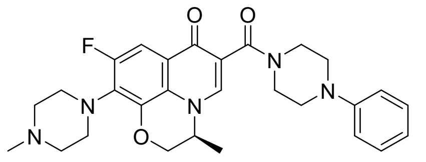

O
O
F
N
N
N
N
N
O

13d ^{13}C

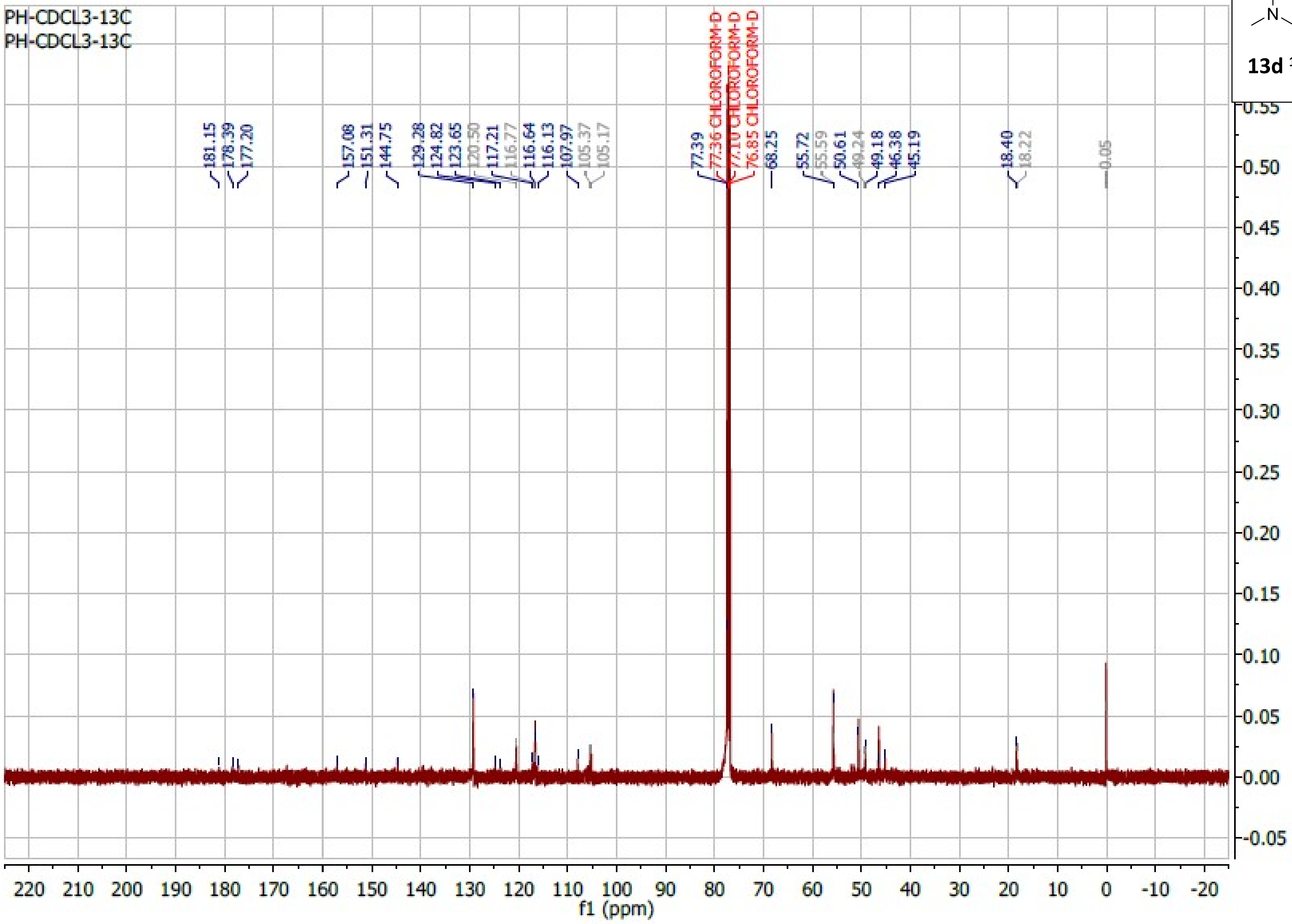

PH-CDCL3-13C
PH-CDCL3-13C
181.15
178.39
177.20
157.08
151.31
144.75
129.28
124.82
123.65
120.50
117.21
116.77
116.64
116.13
107.97
105.37
105.17
77.39
77.36 CHLOROFORM-D
77.10 CHLOROFORM-D
76.85 CHLOROFORM-D
68.25
55.72
55.59
50.61
49.24
49.18
46.38
45.19
18.40
18.22
0.05
0.55
0.50
0.45
0.40
0.35
0.30
0.25
0.20
0.15
0.10
0.05
0.00
-0.05
220 210 200 190 180 170 160 150 140 130 120 110 100 90 80 70 60 50 40 30 20 10 0 -10 -20
f1 (ppm)

Al-Azhar University
The Regional Center for Mycology and Biotechnology

Requester Data:

Name: Dr. Amira Youssef Adib
Authority: Faculty of Pharmacy,
Misr University for Science and Technology University

Sample Data:

Sixteen samples had been submitted for elemental analysis.

Analysis Report:

Sample Code	C%	H%	N%
3a	60.47	5.28	14.32
3b	62.59	5.35	14.81
3c	63.04	5.91	14.47
4a	61.73	5.12	16.80
4b	53.72	4.39	14.67
4c	57.80	4.63	15.73
5	59.41	6.12	14.50
6	59.80	6.13	16.03
7	57.23	5.40	19.17
8	57.09	5.14	15.83
9	61.52	4.95	14.09
10	52.79	5.61	19.12
11a	56.30	4.87	15.26
11b	58.92	5.46	15.39
12a	59.47	5.62	15.04
12b	58.50	5.69	14.31

INVESTIGATOR DIRECTOR

جامعة الأزهر
المركز الإقليمي للفطريات وتطبيقاتها

Al-Azhar University Campus - Nasr City, Cairo, Egypt.
Tel: 0202 22620373 Fax : 0202 22620373
E.mail:rcmb@azhar.edu.eg
Website: http://www.azhar.edu.eg.htm * http://www.azhar.edu.eg/pages/fungi_center.htm
Facebook : RCMB AZHAR P.O. box mail : 11751 Nasr City Cairo, Egypt.

Al-Azhar University

The Regional Center for Mycology and Biotechnology

Requester Data:

Name: Dr. Amira Youssef

Authority: Misr University for Science and Technology University

Sample Data:

Four samples had been submitted for elemental analysis.

Analysis Report:

Sample Code	C%	H%	N%
**13a**	64.70	6.95	13.31
**13b**	61.60	6.46	13.29
**13c**	62.58	7.10	16.05
**13d**	66.41	6.52	14.11

INVESTIGATOR **DIRECTOR**

جامعة الأزهر
المركز الإقليمي للفطريات وتطبيقاتها

Al-Azhar University Campus - Nasr City, Cairo, Egypt.
Tel: 0202 22620373 Fax : 0202 22620373
E.mail:rcmb[illegible]azhar.edu.eg
Website: http://www.azhar.edu.eg.htm * http://www.azhar.edu.eg/pages/fungi_[illegible].htm
Facebook : RCMB AZHAR P.O. box mail : 11751 Nasr City Cairo, Egypt

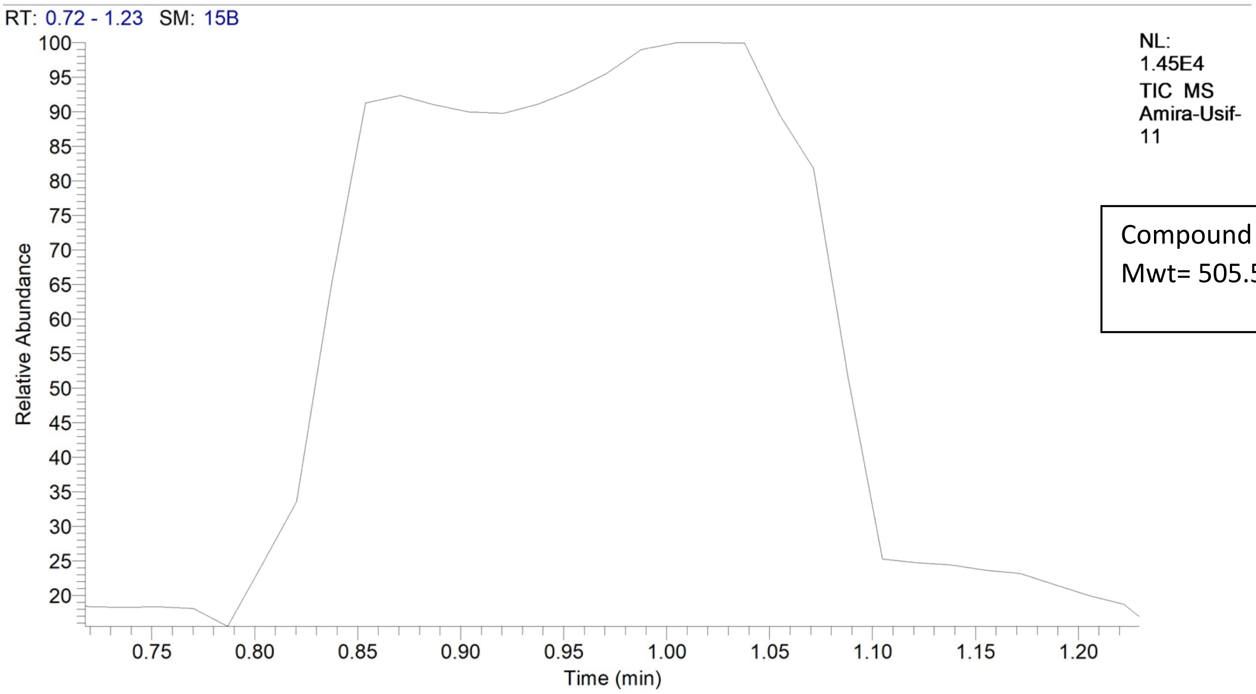

Compound **9**
Mwt= 505.51

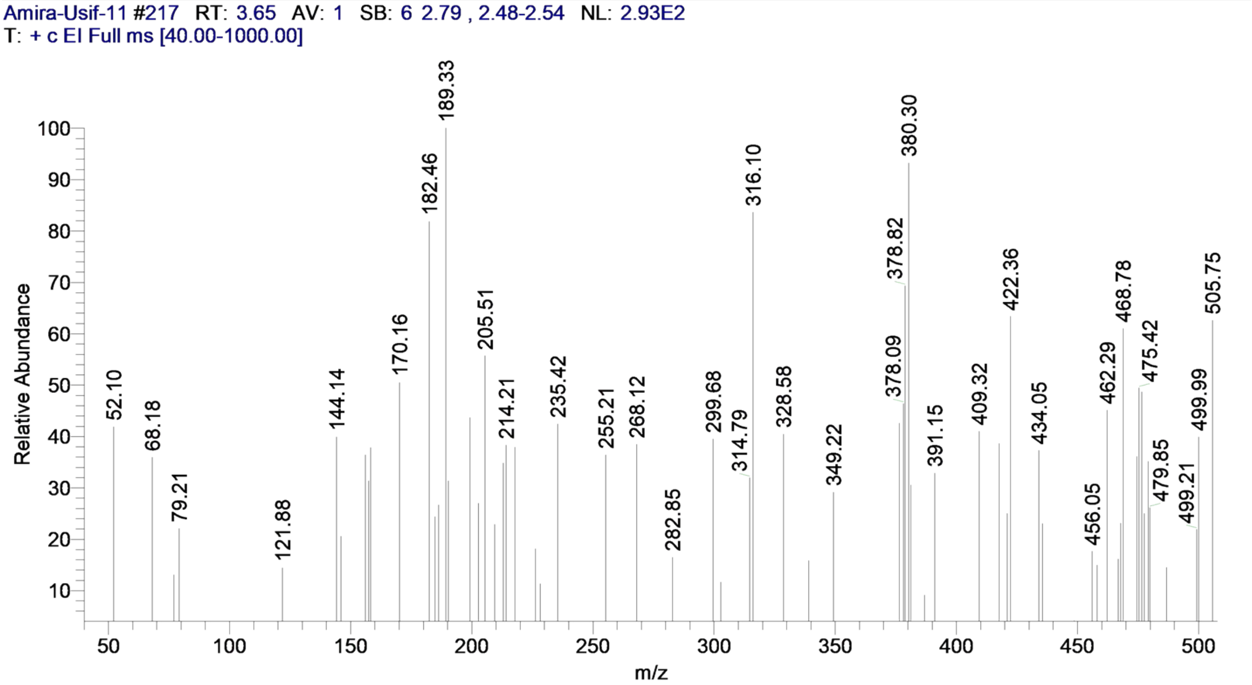

m/z	Intensity	Relative
52.10	122.8	41.96
68.18	105.3	35.98
77.08	38.3	13.08
79.21	64.8	22.13
121.88	42.3	14.44

144.14	117.0	39.97
145.93	60.4	20.65
156.11	106.7	36.45
157.34	91.9	31.39
158.34	110.7	37.81
170.16	147.9	50.52
182.46	239.6	81.87
184.84	71.3	24.37
186.34	78.0	26.65
189.33	292.6	100.00
190.40	91.9	31.39
199.20	127.9	43.71
202.82	78.9	26.97
205.51	163.1	55.72
209.48	67.1	22.92
213.05	102.0	34.85
214.21	112.3	38.36
217.77	111.1	37.98
226.28	53.1	18.13
228.21	33.4	11.41
235.42	124.4	42.51
255.21	106.8	36.49
268.12	112.7	38.50
282.85	48.3	16.51
299.68	115.6	39.51
302.77	34.2	11.70
314.79	93.6	31.98
316.10	244.8	83.64
328.58	118.6	40.52
339.12	46.5	15.88
349.22	85.4	29.18

376.41	124.7	42.60
378.09	136.0	46.47
378.82	203.0	69.35
380.30	272.9	93.26
381.20	89.5	30.57
386.94	26.8	9.16
391.15	96.3	32.89
409.32	120.0	41.02
417.60	113.2	38.68
421.03	73.2	25.03
422.36	185.5	63.39
434.05	109.2	37.31
435.62	67.6	23.08
448.73	12.0	4.10
456.05	51.7	17.66
458.05	44.0	15.04
462.29	132.1	45.15
466.86	47.2	16.13
467.79	67.7	23.14
468.78	178.7	61.06
474.51	105.8	36.17
475.42	144.8	49.48
476.46	142.5	48.70
477.46	73.3	25.06
479.27	102.9	35.17
479.85	76.5	26.15
486.70	42.6	14.55
499.21	64.1	21.91
499.99	116.8	39.91
505.75	183.2	62.61

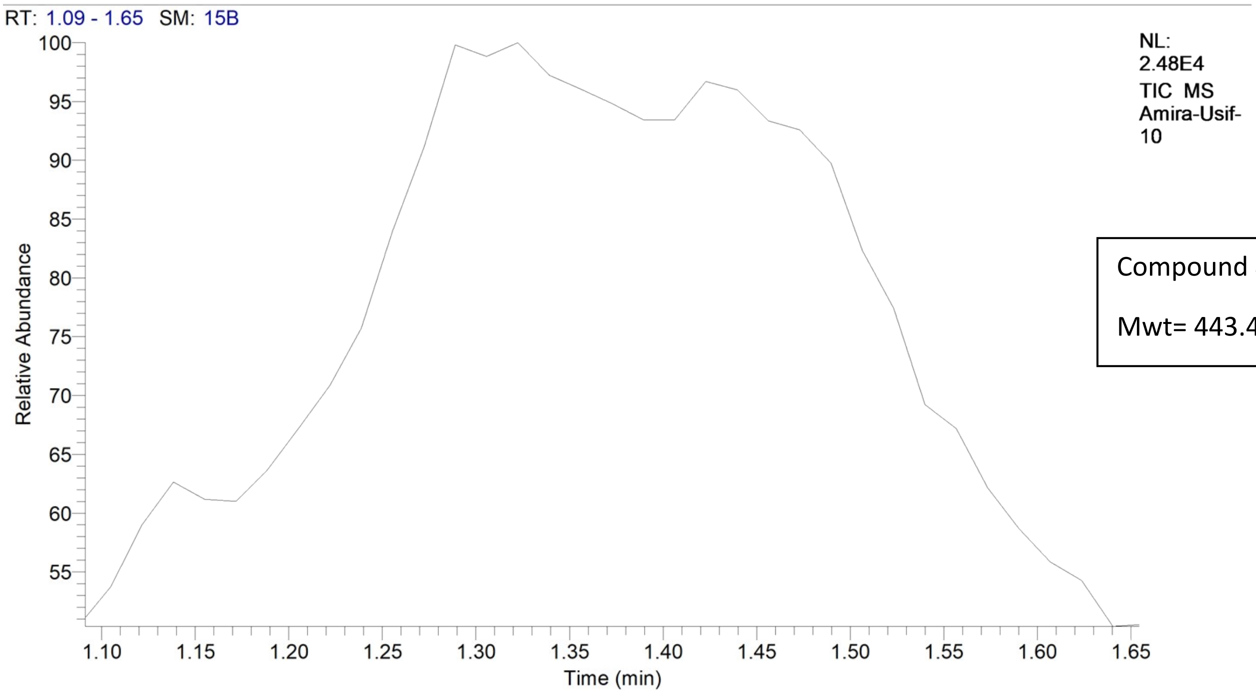

Compound **8**

Mwt= 443.44

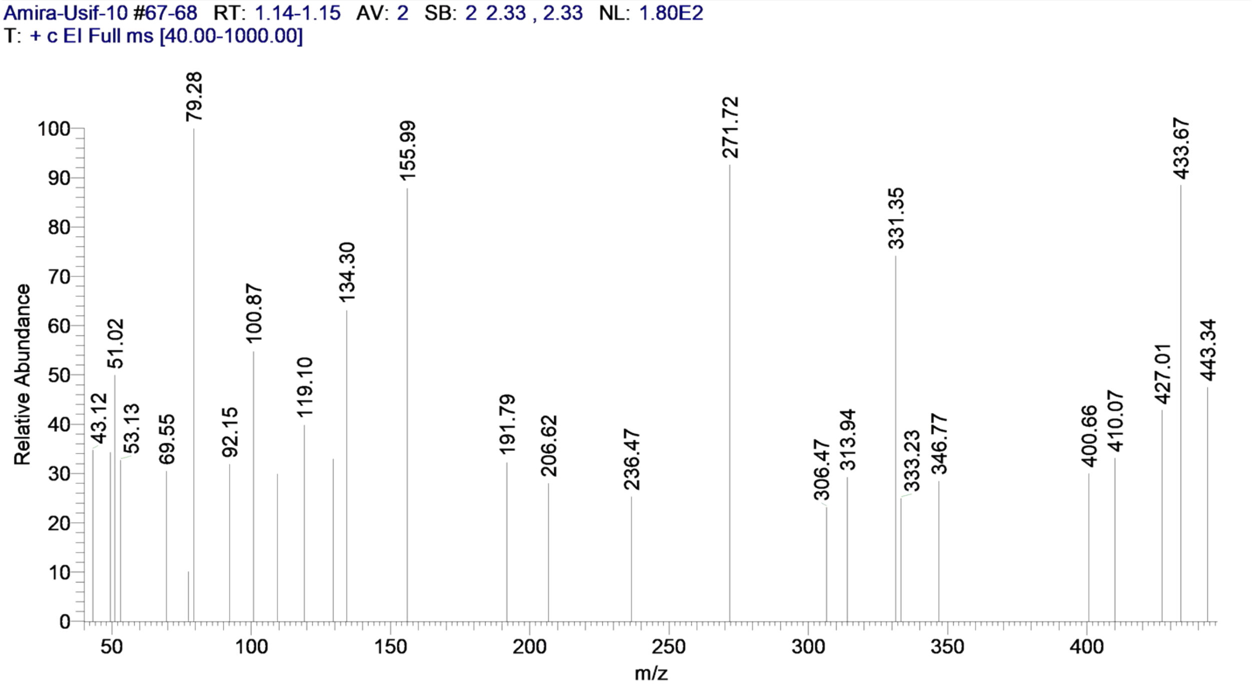

m/z	Intensity	Relative
43.12	62.6	34.82
49.36	61.7	34.33
51.02	89.8	49.94
53.13	58.8	32.70
69.55	54.9	30.55

77.52	18.2	10.12
79.28	179.8	100.00
92.15	57.5	31.96
100.87	98.5	54.76
109.41	53.9	29.96
119.10	71.5	39.78
129.42	59.3	33.00
134.30	113.5	63.11
155.99	157.9	87.80
191.79	58.0	32.26
206.62	50.4	28.03
236.47	45.5	25.32
271.72	166.7	92.70
306.47	41.7	23.17
313.94	52.7	29.29
331.35	133.3	74.16
333.23	44.9	24.99
346.77	51.2	28.48
400.66	54.0	30.03
410.07	59.6	33.15
427.01	77.0	42.83
433.67	159.1	88.47
443.34	85.4	47.50

HPLC purity for compound 5

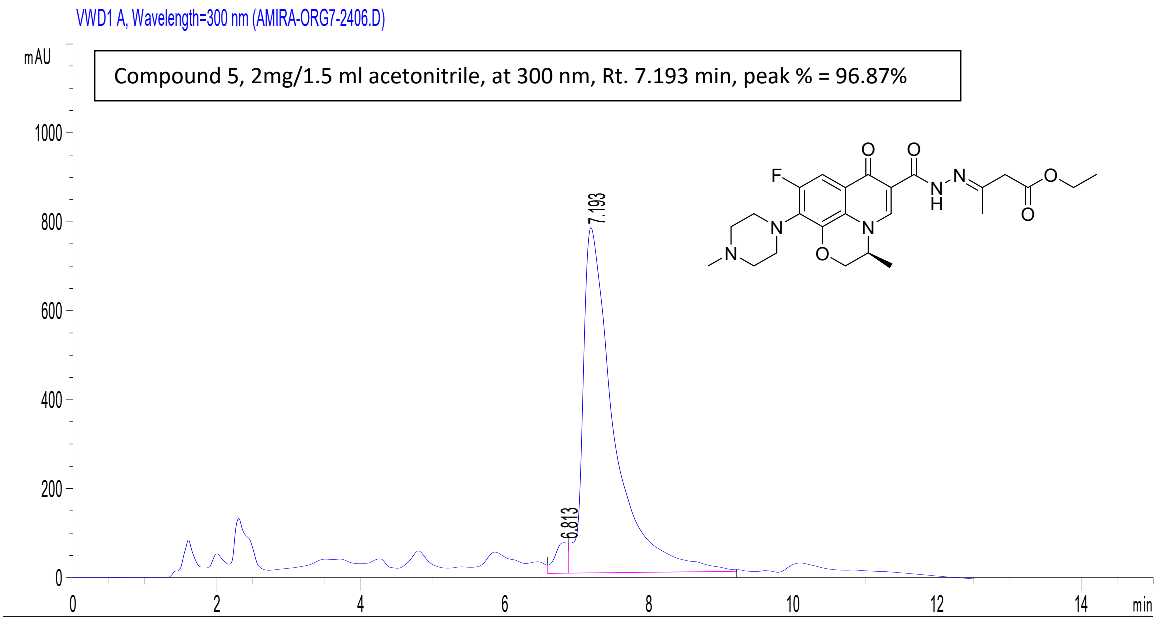

Supplement: RA-014-D4RA03975K-s001 [file RA-014-D4RA03975K-s001.pdf]
